# Proximal tubular FHL2, a novel downstream target of hypoxia inducible factor 1, is a protector against ischemic acute kidney injury

**DOI:** 10.1007/s00018-024-05289-x

**Published:** 2024-05-30

**Authors:** Yan Wang, Ziwei Kuang, Xueqi Xing, Yumei Qiu, Jie Zhang, Dandan Shao, Jiaxin Huang, Chunsun Dai, Weichun He

**Affiliations:** Center for Kidney Disease, Second Affiliated Hospital, Nanjing Medical University, 262 North Zhongshan Road, Nanjing, Jiangsu 210003 China

**Keywords:** FHL2, Hypoxia inducible factor 1α, Β-catenin, Ischemia-reperfusion injury, Proximal tubular cells

## Abstract

Four-and-a-half LIM domains protein 2 (FHL2) is an adaptor protein that may interact with hypoxia inducible factor 1α (HIF-1α) or β-catenin, two pivotal protective signaling in acute kidney injury (AKI). However, little is known about the regulation and function of FHL2 during AKI. We found that FHL2 was induced in renal tubular cells in patients with acute tubular necrosis and mice model of ischemia-reperfusion injury (IRI). In cultured renal proximal tubular cells (PTCs), hypoxia induced FHL2 expression and promoted the binding of HIF-1 to FHL2 promoter. Compared with control littermates, mice with PTC-specific deletion of FHL2 gene displayed worse renal function, more severe morphologic lesion, more tubular cell death and less cell proliferation, accompanying by downregulation of AQP1 and Na, K-ATPase after IRI. Consistently, loss of FHL2 in PTCs restricted activation of HIF-1 and β-catenin signaling simultaneously, leading to attenuation of glycolysis, upregulation of apoptosis-related proteins and downregulation of proliferation-related proteins during IRI. In vitro, knockdown of FHL2 suppressed hypoxia-induced activation of HIF-1α and β-catenin signaling pathways. Overexpression of FHL2 induced physical interactions between FHL2 and HIF-1α, β-catenin, GSK-3β or p300, and the combination of these interactions favored the stabilization and nuclear translocation of HIF-1α and β-catenin, enhancing their mediated gene transcription. Collectively, these findings identify FHL2 as a direct downstream target gene of HIF-1 signaling and demonstrate that FHL2 could play a critical role in protecting against ischemic AKI by promoting the activation of HIF-1 and β-catenin signaling through the interactions with its multiple protein partners.

**Supplementary Information:**

The online version contains supplementary material available at 10.1007/s00018-024-05289-x.

## Introduction

Acute kidney injury (AKI) attracts widespread attention due to its rising morbidity and high mortality [1]. Despite growing understanding in the pathogenesis of AKI, strategies for effective intervention are limited. Renal ischemia-reperfusion injury (IRI) is the leading cause of AKI, commonly related to several clinical settings including cardiovascular surgery, sepsis, and organ transplantation [2]. Molecular pathways that mediate the injuries are being extensively investigated for potential intervention targets [3, 4]. In this regard, hypoxia inducible factor 1 (HIF-1) and β-catenin signaling have been intensively studied and shown to be critical for renal protection during IRI.

HIF-1 is a master transcription factor for cellular adaptations to hypoxia, and one of its principle functions is regulation of metabolism. Activated HIF-1 during IRI mediates metabolic reprogramming from oxidative phosphorylation to glycolysis via its target genes encoding key enzymes including hexokinase 2 (HK2), pyruvate kinase M2 (PKM2), lactate dehydrogenase A (LDHA), pyruvate dehydrogenase kinase 1 (PDK1), etc., which leads to an increase in lactate production and a decrease in mitochondrial production of reactive oxygen species [5, 6]. Comparably, β-catenin is essential in governing various biological processes, especially injury repair and tissue homeostasis. During IRI, activated β-catenin exerts anti-apoptotic and pro-survival actions through multiple intracellular cascades involving Akt, p53, caspase 3, cyclin D1, etc [7, 8].

Activity of HIF-1 or β-catenin is tightly regulated by post-translational modification (PTM). The hydroxylation of HIF-1α by prolyl hydroxylase and subsequent proteasomal degradation is prevented by hypoxia, resulting in its stabilization and translocation into the nucleus, where it dimerizes with HIF-1β to form active transcriptional complex [9, 10]. Likewise, upon Wnts occurs, the phosphorylation and subsequent degradation of β-catenin is impeded, leading to its stabilization and translocation into the nucleus, where it combines with T-cell factor/lymphoid enhancer-binding factor to trigger transcription of Wnt target genes [11, 12]. In addition, glycogen synthetic kinase 3 (GSK-3) that phosphorylates β-catenin can also phosphorylate HIF-1α, making it susceptible to target by proteasome [13]. Furthermore, p300 enhances the transactivation of both signaling by acetylation of HIF-1α and β-catenin [14, 15].

Four-and-a-half LIM domains protein 2 (FHL2) belongs to LIM-domain only protein family [16]. As an adapter protein, its main function is believed to modulate activity of relevant intracellular signaling by combining with protein partners and making them accessible to PTM or their stability affected [17]. Interaction of FHL2 with HIF-1α or β-catenin has been reported, and the effect of FHL2 on the activity of HIF-1 [18–20] or β-catenin [21–23] signaling was not consistent across cell types. Our previous studies have shown that the upregulation of FHL2 in obstructive nephropathy is implicated in the pathogenesis of renal fibrosis through enhancing β-catenin activity [24, 25]. However, the regulation of FHL2 expression by hypoxia remains to be determined, and little is known about its effects on the activity of HIF-1 or β-catenin during IRI. Furthermore, whether FHL2 plays any regulatory role in ischemic AKI is completely unknown.

Here, we investigated the expression, regulation and function of FHL2 in ischemic AKI and its effects on the activity of HIF-1 and β-catenin signaling in vivo and in vitro. Our findings identify FHL2 as a direct downstream target gene of HIF-1 and demonstrate that FHL2 is essential for tubule protection after IRI, mainly owing to its pivotal role in mediating the activation of HIF-1 and β-catenin signaling.

## Results

### FHL2 expression is induced in tubular cells during AKI and activation of HIF-1α by hypoxia mediates FHL2 induction

**Fig. 1:**
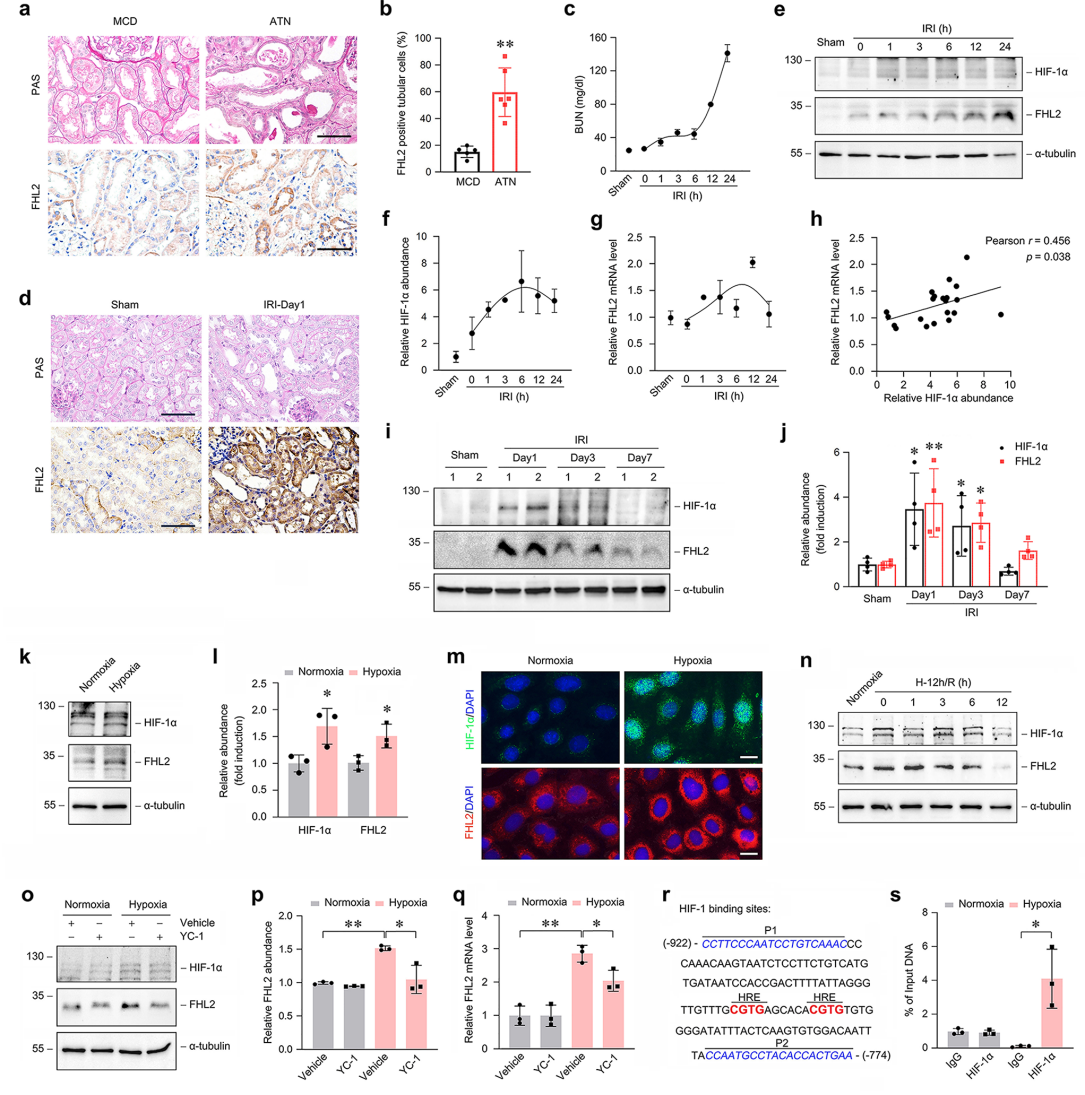
FHL2 expression is induced in tubular cells during ischemic AKI and activation of HIF-1α by hypoxia mediates FHL2 induction. **a, b** Representative micrographs with periodic acid–Schiff (PAS) staining and immunohistochemical staining for FHL2 in the kidney sections from patients with acute tubular necrosis (ATN), and the kidney sections from patients with minimal change disease (MCD) served as controls. Scale bar, 50 μm. Quantitative determination of FHL2 expression in the kidney sections with ATN and MCD is presented in **b**. **P < 0.01 (*n* = 5–6). **c** Dynamic changes of blood urea nitrogen (BUN) in mice within 24 h after IRI (*n* = 3). **d** Representative micrographs with PAS and immunohistochemical staining for FHL2 in mice kidneys at 1 d after IRI. Scale bar, 50 μm. **e, f** Western blot analyses show the induction of both HIF-1α and FHL2 in mice kidneys at different time points within 24 h after IRI compared with sham controls. Quantitative analysis data of HIF-1α protein is presented in **f** (*n* = 3). **g** Graphic presentation shows the relative mRNA levels of FHL2 determined by real-time RT-PCR analyses in mice kidneys at different time points within 24 h after IRI compared with sham controls (*n* = 3). **h** Linear regression analysis shows a close correlation between HIF-1α abundance and FHL2 mRNA level (arbitrary units). The Pearson correlation coefficient (*r*) is shown. **i, j** Representative Western blot shows the changes of HIF-1α and FHL2 abundance in mice kidneys at 1, 3, and 7 d after IRI compared with sham controls. Numbers (1 and 2) indicate each individual animal in a given group. Quantitative analysis data is presented in **j**. *P < 0.05, **P < 0.01 (*n* = 4). Representative blots of three (**e**) or two (**i**) experiments, and one (**e**) or two (**i**) individual animals in each experiment. Data are mean ± SD of three (**c, f, g**) or four (**j**) individual animals. **k-s** NRK-52E cells were used. **k-m** Western blot analyses **k, l** and immunofluorescence staining **m** show the changes of HIF-1α and FHL2 protein in cells after 12 h of hypoxia compared with normoxia controls. Quantitative analysis data of HIF-1α and FHL2 protein is presented in **l**. *P < 0.05 (*n* = 3). **n** Western blot analyses show the abundance of HIF-1α and FHL2 protein at different time points of re-oxygenation after 12 h of hypoxia compared with normoxia controls. H, hypoxia; R, re-oxygenation. **o, p** Western blot analyses show the induction of FHL2 protein by hypoxia was abolished following the inhibition of HIF-1α by YC-1 (10 µM). Quantitative analysis data is presented in **p**. *P < 0.05, **P < 0.01 (*n* = 3). **q** Real-time RT-PCR analysis shows the induction of FHL2 mRNA by hypoxia was abolished by YC-1 (10 µM). *P < 0.05, **P < 0.01 (*n* = 3). **r, s** Chromatin immunoprecipitation (ChIP) assay reveals that hypoxia promotes HIF-1α binding to the hypoxia response elements (HREs) in the FHL2 promoter. Partial sequence of rat FHL2 gene promoter region is presented in **r**. Bold red letters indicate two HREs, and P1 and P2 indicate the primer pair encompassing these HREs. Quantitative ChIP data is presented in **s**. *P < 0.05 (*n* = 3). Representative blots of two (**n**) or three (**k, o**) independent experiments. Data are mean ± SD of three (**l, p, q, s**) independent experiments

To establish the potential correlation between FHL2 regulation and AKI, we first observed the expression and localization of FHL2 in renal biopsy specimens from six AKI patients with acute tubular necrosis (ATN), while biopsy specimens from five minimal change disease (MCD) patients with minor tubular damage were used as controls. We found that FHL2 was expressed at quite low levels in the tubules in MCD, whereas its expression was largely induced in ATN (Fig. 1a, b).

Since ischemic hypoxia is a common cause of AKI, we next examined the correlation between FHL2 expression and HIF-1α activation in a mouse model of IRI. AKI was confirmed by rapidly elevated levels of blood urea nitrogen (BUN) (Fig. 1c) and pathological changes in the kidneys (Fig. 1d) after IRI. Western blot analyses showed that FHL2 protein expression was upregulated in a time dependent manner following the increase in abundance of HIF-1α after IRI (Fig. 1e, f). Immunostaining exhibited that FHL2 expression was mainly induced in tubular cells in mice after IRI (Fig. 1d). FHL2 mRNA level was also induced, which was determined by quantitative real-time RT-PCR (Fig. 1g). Linear regression showed a positive correlation between FHL2 mRNA level and HIF-1α abundance (Fig. 1h). At 1, 3 and 7 days after IRI, the expression of FHL2 reduced gradually following the decrease in HIF-1α abundance and approached the baseline (Fig. 1i, j).

We next investigated FHL2 regulation by hypoxia in rat proximal tubular cells (PTCs). Western blot analyses showed that FHL2 expression was induced by hypoxia with an increase in the abundance of HIF-1α (Fig. 1k, l). Immunostaining displayed that FHL2 expression was upregulated in both cytosol and nuclei following nuclear translocation of HIF-1α after hypoxia compared with normoxic conditions (Fig. 1m). The hypoxia-induced increase in the abundance of HIF-1α was terminated by reoxygenation, and the abundance of HIF-1α gradually returned to the baseline under normoxic conditions. The change pattern of FHL2 expression was similar to that of HIF-1α abundance (Fig. 1n). Then we used YC-1, a specific HIF-1α inhibitor [26], to determine any involvement of HIF-1α activation in FHL2 regulation. Western blot and quantitative determination revealed a 1.5-fold induction of FHL2 expression at 12 h of hypoxia, which was almost blocked by YC-1 (Fig. 1o, p). Similar result was obtained when FHL2 mRNA was detected (Fig. 1q).

To elucidate the mechanism underlying HIF-1α regulation of FHL2 expression, we analyzed the structure of rat FHL2 gene promoter [27]. Bioinformatics analysis revealed two putative hypoxia response elements (HREs) in the proximal promoter region of rat FHL2 gene (Fig. 1r). We examined the physical and functional interaction between HIF-1 and its cognate HREs in FHL2 promoter by using an in situ chromatin immunoprecipitation (ChIP) assay. We found that activation of HIF-1α by hypoxia promoted HIF-1 binding to at least one of the HREs (Fig. 1s). Therefore, HIF-1α activation is able to facilitate HIF-1 binding to one or two of the HREs, leading to FHL2 transcription.

### Proximal tubule-specific ablation of FHL2 aggravates AKI in mouse IRI model

**Fig. 2:**
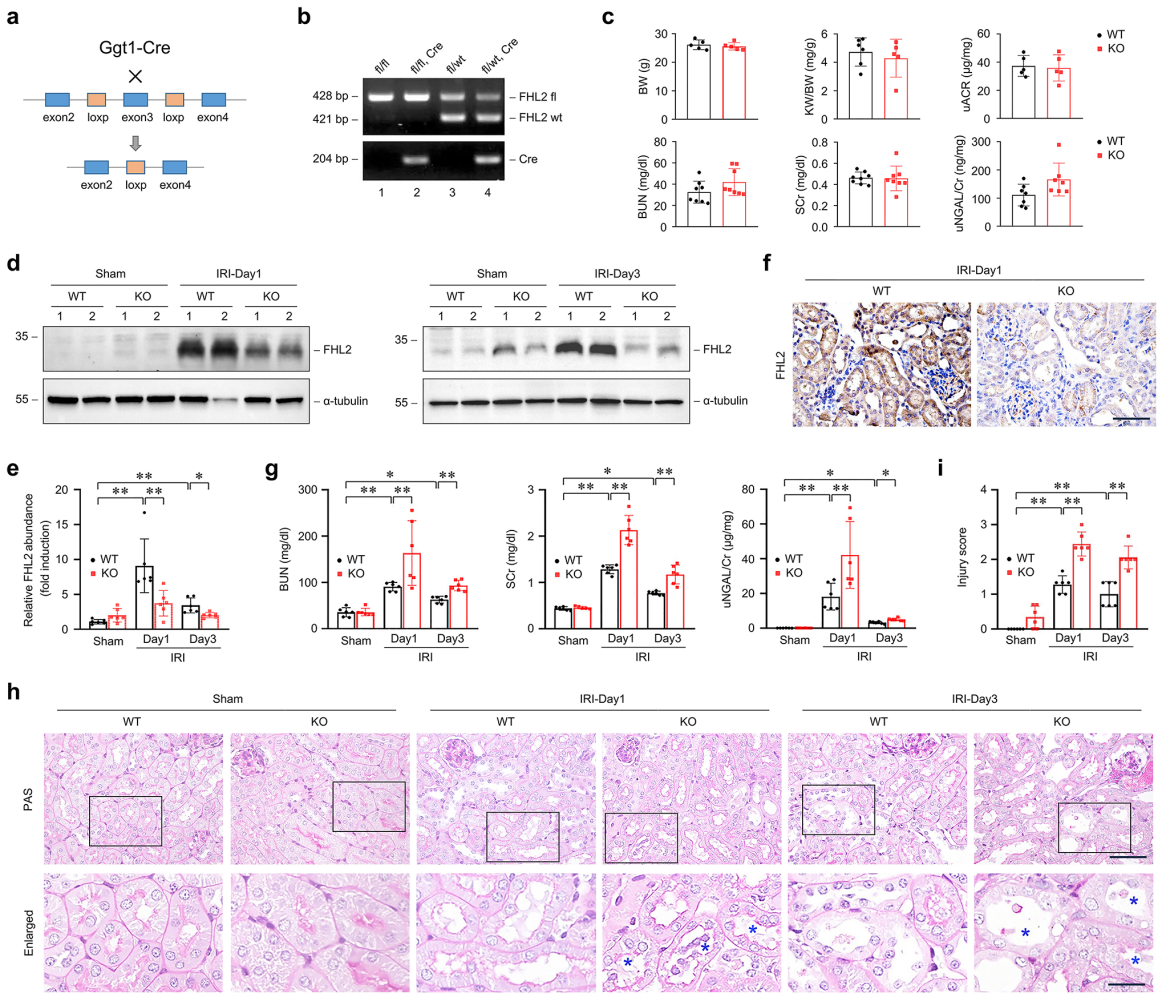
Proximal tubule-specific ablation of FHL2 aggravates AKI induced by IRI in mice. **a** Schematic diagram illustrates the strategy for generating conditional knockout mice with proximal tubular cell-specific ablation of FHL2 by using Cre-LoxP system. Blue boxes indicate the exons of FHL2 gene. Orange boxes denote LoxP site. **b** Genotyping the mice by PCR analysis of genomic DNA. Lane 3 shows genotyping of the wildtype (WT) control mice used in this study (genotype: FHL2^fl/wt^), and lane 2 denotes genotyping of the tubule-specific FHL2 knockout (KO) mice (genotype: FHL2^fl/fl, Cre^). **c** There was no difference in body weight (BW), ratio of kidney weight to body weight (KW/BW), blood urea nitrogen (BUN), serum creatinine (SCr), urinary albumin-creatinine ratio (uACR) and urine NGAL-creatinine ratio (uNGAL/Cr) between KO and WT mice at 8 w after birth (*n* = 5–8). **d, e** Western blot analyses show FHL2 abundance in the kidney lysates from different groups as indicated. Numbers (1 and 2) indicate each individual animal in a given group **d**. Quantitative analysis data is presented in **e**. *P < 0.05, **P < 0.01 (*n* = 6). **f** Representative micrographs with immunohistochemical staining for FHL2 in the kidneys from WT and KO mice at 1 d after IRI. Scale bar, 50 μm. **g** Graphic presentation of BUN, SCr and uNGAL/Cr in WT and KO mice at 1 and 3 d after IRI. *P < 0.05, **P < 0.01 (*n* = 6). **h, i** Morphological injury assessed in the periodic acid-Schiff (PAS)-stained kidney sections in WT and KO mice. Representative micrographs of the kidneys at 1 and 3 d after IRI and sham controls **h** and quantitative assessment of injury **i** are presented. Blue asterisks in the enlarged box areas denote injured tubules. Scale bar, 50 μm, 20 μm (Enlarged). **P < 0.01 (*n* = 6). Representative blots of three (**d**) experiments, and two individual animals in each experiment. Data are mean ± SD of six (**e, g, i**) or five to eight (**c**) individual animals. NGAL, neutrophil gelatinase-associated lipocalin

To determine the potential regulatory role of FHL2 in AKI, we generated a mouse model with PTC-specific deletion of FHL2 gene by the Cre-LoxP system. The breeding strategy for the generation is shown in Fig. 2a. Mice with PTC-specific knockout (KO) of FHL2 gene were named KO mice in this study (Fig. 2b, lane 2), whereas the age- and gender-matched FHL2-floxed littermates served as wildtype (WT) controls were named WT mice (Fig. 2b, lane 3). All mice were born with expected Mendelian frequency. There was no significant difference in body weight, kidney/body weight ratio, BUN and serum creatinine (SCr) level, urinary albumin to creatinine ratio and neutrophil gelatinase-associated lipocalin (NGAL) to creatinine ratio (NGAL/Cr) between KO and WT mice at 8 weeks after birth (Fig. 2c). Thus KO mice have normal phenotypes in physiological state within 8 weeks after birth.

Western blot analyses of whole kidney lysates showed that the abundance of FHL2 protein in sham kidneys was no difference between WT and KO mice, which reasonably owing to the very low level of FHL2 expression in renal tubules under physiological conditions, while after IRI, FHL2 protein expression in the kidneys from KO mice was remarkably lower than that from WT littermates (Fig. 2d, e). Immunostaining exhibited that the number of FHL2-positive tubular cells in the kidneys from KO mice was significantly lower than that in WT littermates after IRI (Fig. 2f). Then we investigated the effect of FHL2 deficiency in PTCs on renal damage induced by IRI. As shown in Fig. 2g, the levels of BUN and SCr and urinary NGAL/Cr were markedly higher in KO mice than those in WT littermates after IRI. Consistently, compared with WT littermates, the kidneys from KO mice after IRI displayed more severe morphological lesion, featured by more severe tubular dilatation, loss of brush border, tubular cell depletion, and cellular debris and cast formation in the lumen (Fig. 2h, enlarged box, blue stars). Quantitative assessment of kidney morphological injury between WT and KO mice at 1 and 3 days after IRI is presented in Fig. 2i. These data suggest that loss of endogenous FHL2 exacerbates acute renal impairment after IRI.

To explore the mechanism underlying the protective role of endogenous FHL2 in AKI, we further examined the effect of FHL2 deficiency in PTCs on apoptosis and proliferation during IRI.

**Fig. 3:**
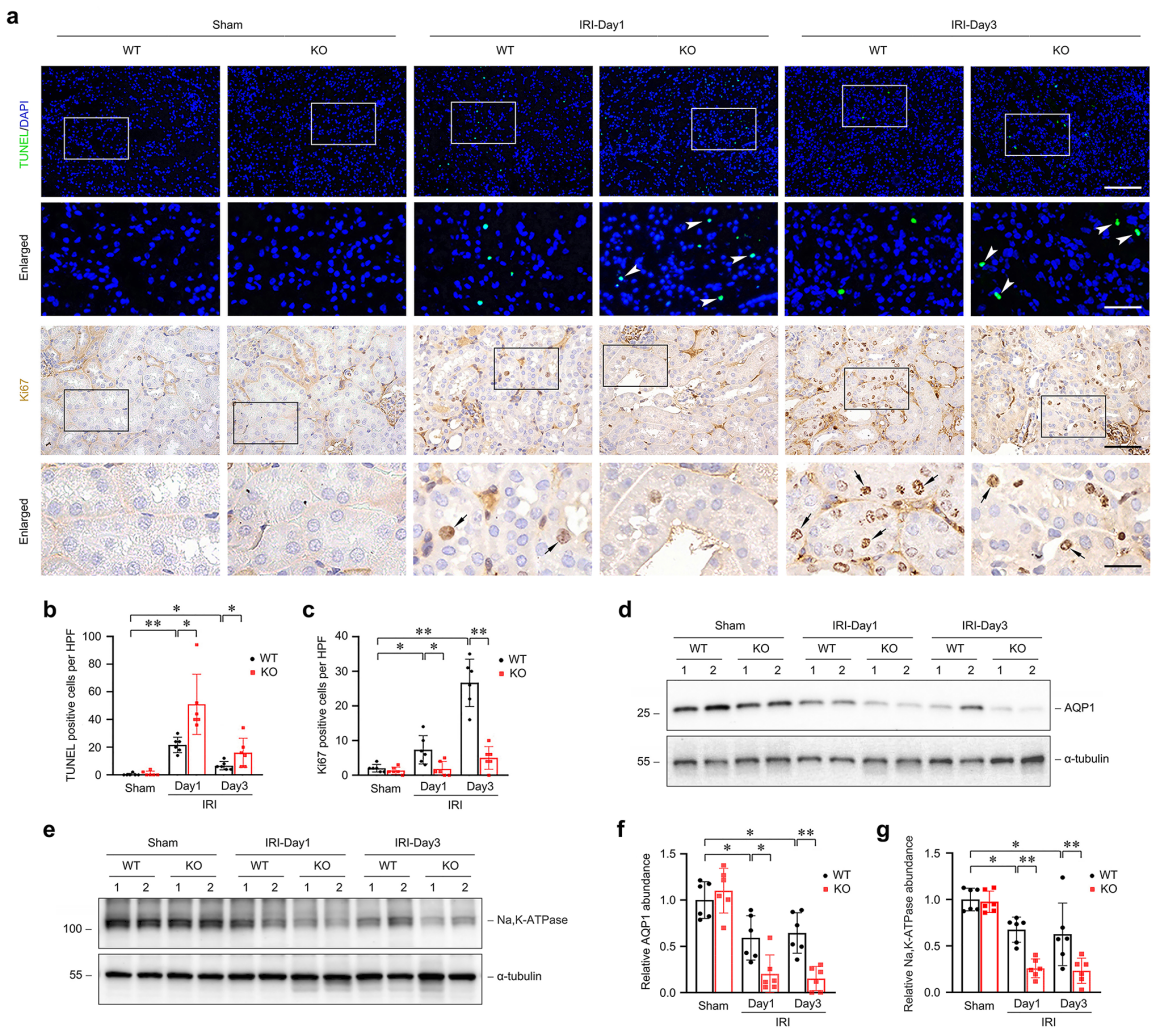
Specific deletion of FHL2 in proximal tubules aggravates acute kidney injury in mice after IRI. **a-c** Specific deletion of FHL2 in proximal tubules promotes apoptosis and suppresses proliferation after renal IRI. **a** Representative micrographs show apoptotic cell death detected by TUNEL staining and cell proliferation detected by immunohistochemical staining for Ki67. In the enlarged box areas, white arrowheads indicate apoptotic cells, whereas black arrows indicate proliferative cells. Scale bar, 50 μm, 20 μm (Enlarged). **b, c** Quantitative determination of apoptotic and proliferative cells in the kidneys from WT and KO mice at 1 and 3 d after IRI compared with sham controls, respectively. Data are presented as numbers of TUNEL-positive **b** and Ki67-positive **c** cells per high power field (HPF). *P < 0.05, **P < 0.01 (*n* = 6). **d-g** Proximal tubule-specific deletion of FHL2 aggravates the downregulation of AQP1 and Na, K-ATPase protein after renal IRI. Representative western blot analyses present the expression of AQP1 **d** and Na, K-ATPase **e** in the kidneys from WT and KO mice at 1 and 3 d after IRI compared with sham controls, respectively. Numbers (1 and 2) indicate each individual animal in a given group. Graphic presentations show the relative abundance of AQP1 **f** and Na, K-ATPase **g** in different groups as indicated. *P < 0.05, **P < 0.01 (*n* = 6). Representative blots of three (**d, e**) experiments and two individual animals in each experiment. Data are mean ± SD of six (**b, c, f, g**) individual animals. TUNEL, terminal deoxynucleotidyl transferase-mediated dUTP nick-end labeling

As shown in Fig. 3a, terminal deoxynucleotidyl transferase-mediated dUTP nick-end labeling (TUNEL) staining revealed considerable apoptosis in the kidneys from WT mice at 1 day after IRI, and then the frequency of apoptosis reduced at 3 days after IRI but remained higher than the baseline in sham controls. The frequency of apoptosis in the kidneys from KO mice was significantly higher than that in WT littermates after IRI under the same conditions (Fig. 3a, enlarged box, white arrowheads). Conversely, immunostaining for Ki67, a marker for proliferating cells in the late G1 to M phases, identified a remarkable increase in the number of Ki67-positive cells in the kidneys from WT mice at 1 and 3 days after IRI, whereas the number of Ki67-positive cells in the kidneys from KO mice was markedly less than that in WT littermates after IRI (Fig. 3a, enlarged box, black arrows). Quantitative data on apoptotic cells (Fig. 3b) and proliferating cells (Fig. 3c) in the kidneys from WT and KO mice are presented, respectively. These results suggest that proximal tubule-specific ablation of FHL2 aggravates kidney injury by promoting apoptosis and suppressing proliferation.

To further determine the effect of FHL2 deficiency in PTCs on the severity of renal lesion after IRI, we investigated the expression of aquaporin (AQP) 1 and Na, K-ATPase, two membrane proteins essential for the functions of tubules [28–30]. AQP1 is one of water-transporting proteins and Na, K-ATPase is an ion channel using energy derived from ATP. Western blot analyses showed that AQP1 and Na, K-ATPase protein levels were reduced in the kidneys from WT mice after IRI compared with sham controls, and their levels in the kidneys from KO mice were distinctly lower than those from WT littermates after IRI (Fig. 3d-g). These data further confirm that proximal tubule’s FHL2 deficiency exacerbates renal damage after IRI.

### Elimination of endogenous FHL2 restrains HIF-1 activation and glucose metabolic switch after IRI

**Fig. 4:**
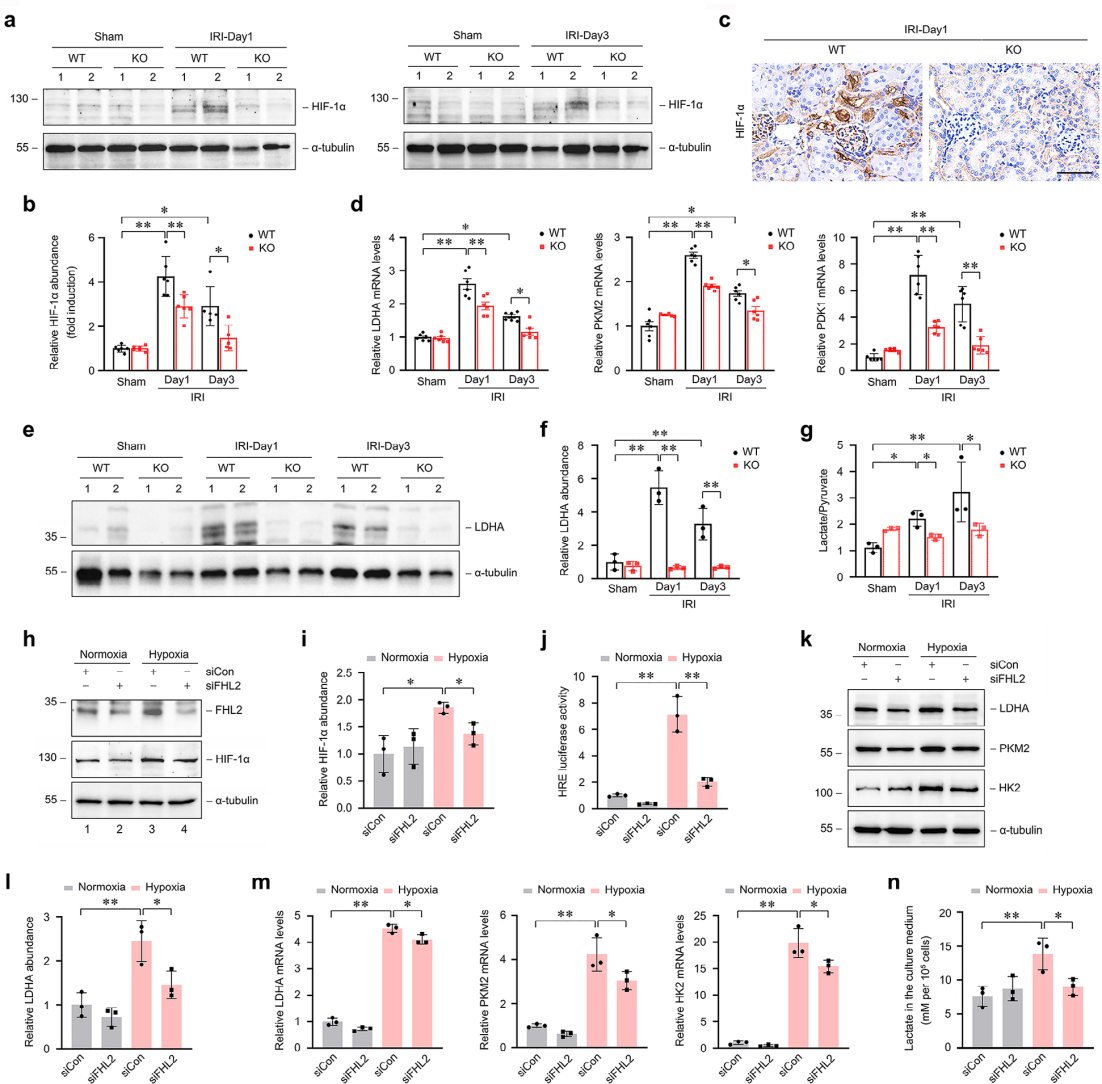
Specific deletion of FHL2 in proximal tubules restrains HIF-1 activation and glucose metabolic switch during IRI. **a, b** Western blot analyses show the abundance of HIF-1α in the kidneys from WT and KO mice at 1 and 3 d after IRI and sham controls. Numbers (1 and 2) indicate each individual animal in a given group **a**. Quantitative analysis data is presented in **b**. *P < 0.05, **P < 0.01 (*n* = 6). **c** Representative micrographs show the immunohistochemical staining for HIF-1α in the kidneys from WT and KO mice at 1 d after IRI. Scale bar, 50 μm. **d** Graphic presentations show the relative mRNA levels of LDHA, PKM2 and PDK1 determined by real-time RT-PCR analyses in the kidneys from various groups as indicated. *P < 0.05, **P < 0.01 (*n* = 6). **e, f** Western blot analyses show the abundance of LDHA in the kidneys from WT and KO mice at 1 and 3 d after IRI. Numbers (1 and 2) indicate each individual animal in a given group **e**. Quantitative analysis data is presented in **f**. **P < 0.01 (*n* = 3). **g** Graphic presentation shows the ratio of lactate to pyruvate level in kidney tissue lysate from various groups as indicated. *P < 0.05, **P < 0.01 (*n* = 3). Representative blots of three (**a, e**) experiments and two individual animals in each experiment. Data are mean ± SD of six (**b, d**) or three (**f, g**) individual animals. **h, i, k-n** NRK-52E cells were transfected with either control (siCon) or FHL2 (siFHL2) siRNA and then treated with or without hypoxia for 12 h. **h, i** Western blot analyses show the abundance of FHL2 and HIF-1α. Quantitative analysis data for HIF-1α is presented in **i**. *P < 0.05 (*n* = 3). **j** Cells were co-transfected with HRE reporter plasmid (pGL4.42) and either siCon or siFHL2 and then treated with or without hypoxia for 12 h. Relative luciferase activities are reported. **P < 0.01 (*n* = 3). **k, l** Western blot analyses show the abundance of LDHA, PKM2 and HK2. Quantitative analysis data of LDHA is presented in **l**. *P < 0.05, **P < 0.01 (*n* = 3). **m** Graphic presentations show the relative mRNA levels of LDHA, PKM2 and HK2 determined by real-time RT-PCR analyses. *P < 0.05, **P < 0.01 (*n* = 3). **n** Graphic presentation shows lactate levels in the cell culture supernatant. *P < 0.05, **P < 0.01 (*n* = 3). Representative blots of three (**h, k**-LDHA) or two (**k**-PKM2, HK2) independent experiments. Data are mean ± SD of three (**i, j, l-n**) independent experiments. LDHA, lactate dehydrogenase A; PKM2, pyruvate kinase M2; HK2, hexokinase 2; PDK1, pyruvate dehydrogenase kinase 1

Since HIF-1α is a potential interactor of FHL2, to determine whether FHL2 is involved in activation of HIF-1 signaling, we first compared HIF-1α protein levels in the kidneys between KO and WT mice. Western blot analyses showed that HIF-1α protein level in the kidneys from KO mice was much lower than that in WT littermates at 1 and 3 days after IRI, respectively (Fig. 4a, b). Immunostaining displayed less HIF-1α-positive tubules in the kidneys from KO mice than that from WT littermates after IRI (Fig. 4c). We then compared the induction of HIF-1 target genes that are key enzymes involved in HIF-1-dependent metabolic switching in response to hypoxia, in the kidneys between KO and WT mice after IRI. Quantitative real-time RT-PCR determined that mRNA levels of LDHA, PKM2 and PDK1 in the kidneys from KO mice were significantly lower than that from WT littermates after IRI under the same conditions, respectively (Fig. 4d). Western blotting analyses showed markedly lower levels of LDHA protein in the kidneys from KO mice than that in WT littermates after IRI (Fig. 4e, f). Since HIF-1 governs metabolic switch from oxidative to glycolysis, we next compared lactate levels in the kidneys between KO and WT mice after IRI. As shown in Fig. 4g, the ratio of lactate to pyruvate increased in the kidneys from WT mice after IRI in a time dependent pattern compared to sham controls, while the ratio was almost unchanged in the kidneys from KO mice after IRI compared to sham controls, and accordingly, it in the kidneys from KO mice was significantly lower than that from WT mice after IRI under identical conditions.

For further exploring whether the upregulation of FHL2 by hypoxia is important and necessary in regulating HIF-1 signaling activity, we investigated the effect of knocking down FHL2 via transfection with specific siRNA on HIF-1α activation induced by hypoxia in PTCs. Western blot analyses showed that downregulation of FHL2 had no significant effect on HIF-1α protein level under normoxic conditions, but hindered hypoxia-induced HIF-1α stabilization (Fig. 4h, i). Knockdown of FHL2 expression also restrained hypoxia-stimulated HIF-1-mediated transcriptional activation in a HRE luciferase report system (Fig. 4j). Furthermore, downregulation of FHL2 attenuated upregulation of LDHA, PKM2 and HK2 protein expression induced by hypoxia (Fig. 4k). Quantitative determination of LDHA protein is presented in Fig. 4l. Similar results were obtained when levels of LDHA, PKM2 and HK2 mRNA were detected (Fig. 4m). The increase in lactate production induced by hypoxia was hampered by knockdown of FHL2 (Fig. 4n). Hence, these data suggest that FHL2 plays an important role in mediating HIF-1 signaling activation and the switch of glycose metabolism in PTCs under hypoxic conditions.

### Ablation of endogenous FHL2 impedes β-catenin activation and is involved in promoting apoptosis and inhibiting proliferation after IRI

**Fig. 5:**
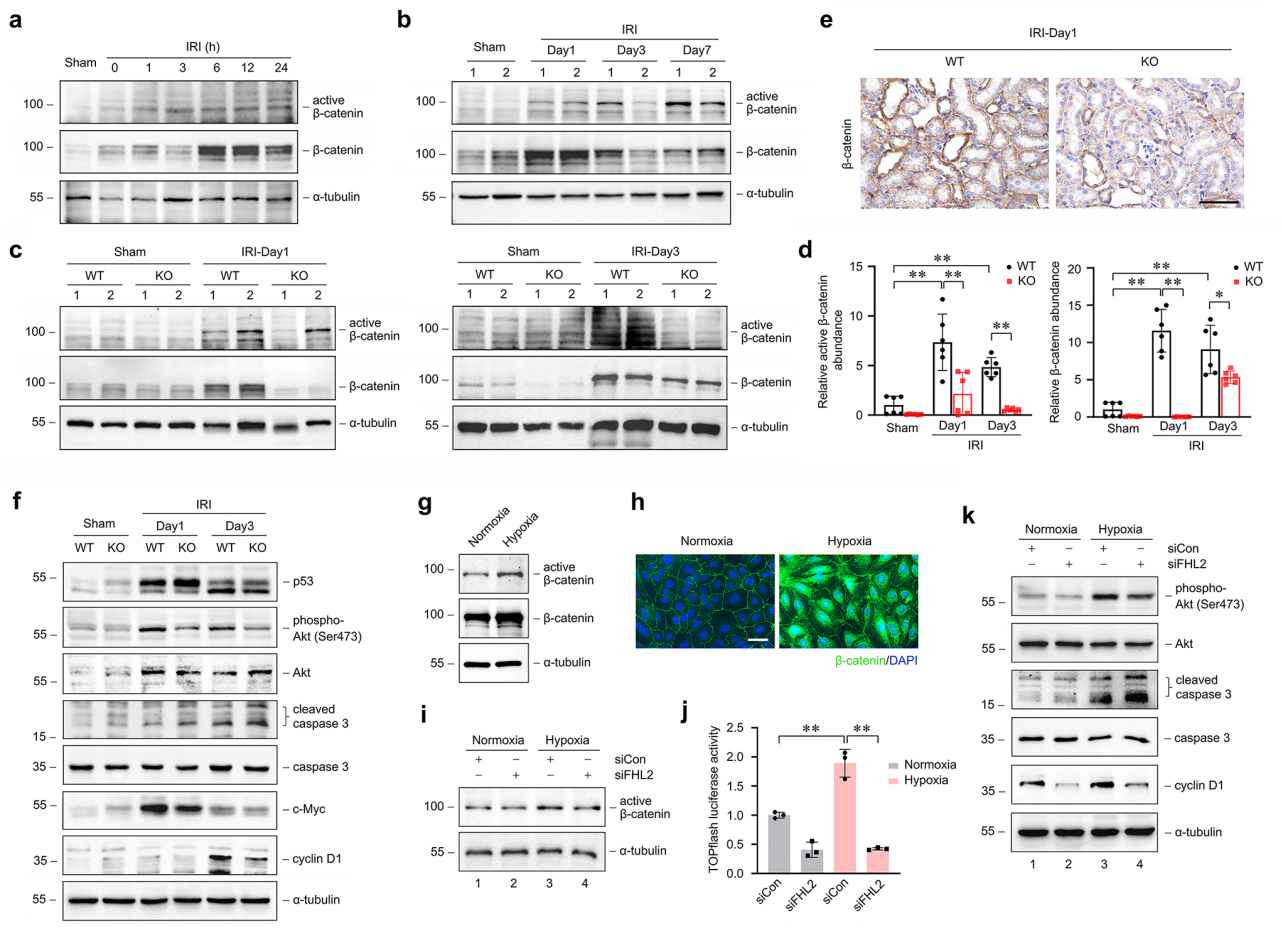
Proximal tubule-specific ablation of FHL2 impedes β-catenin activation and is involved in promoting apoptosis and inhibiting proliferation during IRI. **a, b** Western blot analyses show the induction of renal active β-catenin and total β-catenin at different time points after IRI. **c, d** Western blot analyses show the abundance of active β-catenin and total β-catenin in the kidneys from WT and KO mice at 1 and 3 d after IRI and sham controls. Numbers (1 and 2) indicate each individual animal in a given group **b, c**. Quantitative analysis data is presented in **d**. *P < 0.05, **P < 0.01 (*n* = 6). **e** Representative micrographs show the immunohistochemical staining for β-catenin in the kidneys from WT and KO mice at 1 d after IRI. Scale bar, 50 μm. **f** Western blot analyses show the abundance of p53, phosphorylated Akt (Ser473), cleaved caspase3, c-Myc and cyclin D1 in the kidneys from various groups as indicated. Representative blots of two (**a, f**) or three (**b, c**) experiments, and one (**a**) or two (**b, c**) individual animals, or one kidney preparation from a pool of two animals (**f**) in each experiment. Data are mean ± SD of six (**d**) individual animals. **g-k** NRK-52E cells were used. **g** Western blot analyses show the abundance of active β-catenin and total β-catenin in cells at 12 h of hypoxia compared with normoxia control. **h** Representative micrograph shows the immunofluorescence staining for β-catenin in cells at 12 h of hypoxia compared with normoxia control. **i, k** Cells were transfected with either control (siCon) or FHL2 (siFHL2) siRNA and then treated with or without hypoxia for 12 h. **i** Western blot analyses show the abundance of active β-catenin. **j** Cells were co-transfected with TOPflash reporter plasmid and either siCon or siFHL2 and then treated with or without hypoxia for 12 h. Relative luciferase activities are reported. **P < 0.01 (*n* = 3). **k** Western blot analyses show the abundance of phosphorylated Akt (Ser473), cleaved caspase 3 and cyclin D1. Representative blots of two (**g**) or one (**i, k**) independent experiments. Data are mean ± SD of three (**j**) independent experiments

Our previous studies has shown that FHL2 could modulate β-catenin activity in a mouse model of obstructive nephropathy [24, 25]. Since renal activation of β-catenin has been demonstrated to be protective in AKI, we reasoned that the aggravation of kidney injury during IRI due to PTC-specific deletion of FHL2 is partially associated with inhibition of β-catenin activity. Similar to previous study [8, 31], β-catenin signaling was dramatically activated, as illustrated by a time-dependent increase in the level of active β-catenin (dephosphorylated on Ser37/Thr41) and total β-catenin protein in kidneys after IRI (Fig. 5a, b). However, protein levels of both active β-catenin and total β-catenin in the kidneys from KO mice were significantly lower than that from WT littermates at 1 and 3 days after IRI, respectively (Fig. 5c, d). Immunostaining exhibited that β-catenin-positive tubules in the kidneys from KO mice was much less than that from WT littermates after IRI (Fig. 5e). Because the cytoprotective effect of β-catenin is attributed to inhibition of proapoptotic genes and activation of prosurvival genes, which has been indicated by cumulative reported data [7, 32], and the data in Fig. 3a-c have suggested that loss of FHL2 in PTCs aggravated apoptosis and hindered proliferation after IRI, we further examined several downstream targets of β-catenin associated with modulating apoptotic and proliferative pathways. We found that in the kidneys from KO mice after IRI, the levels of p53 and cleaved caspase 3 were higher than those from WT littermates, whereas the levels of phosphorylated Akt (Ser473), cyclin D1 and c-Myc were lower than those under identical conditions (Fig. 5f).

Then, we investigated the effect of FHL2 on hypoxia-induced activation of β-catenin signaling pathway in vitro. Hypoxia-induced upregulation and nuclear translocation of β-catenin are shown in Fig. 5g, h. Knockdown of FHL2 expression suppressed hypoxia-induced upregulation of active β-catenin (Fig. 5i) and β-catenin-mediated transcription (Fig. 5j). Accordingly, downregulation of FHL2 restrained hypoxia-induced upregulation of phosphorylated Akt (Ser473) and cyclin D1 and promoted hypoxia-induced upregulation of cleaved caspase 3 (Fig. 5k). In addition, PI staining assay showed that downregulation of FHL2 significantly promote hypoxia-induced cell death (Supplemental Fig. 1a, b). These data suggest that proximal tubular FHL2 plays an essential part in modulating β-catenin signaling activity and contributes to ameliorate apoptosis and enhance cell proliferation after IRI.

### Overexpression of FHL2 protects PTCs against hypoxia-induced injury through activation of HIF-1 and β-catenin

**Fig. 6:**
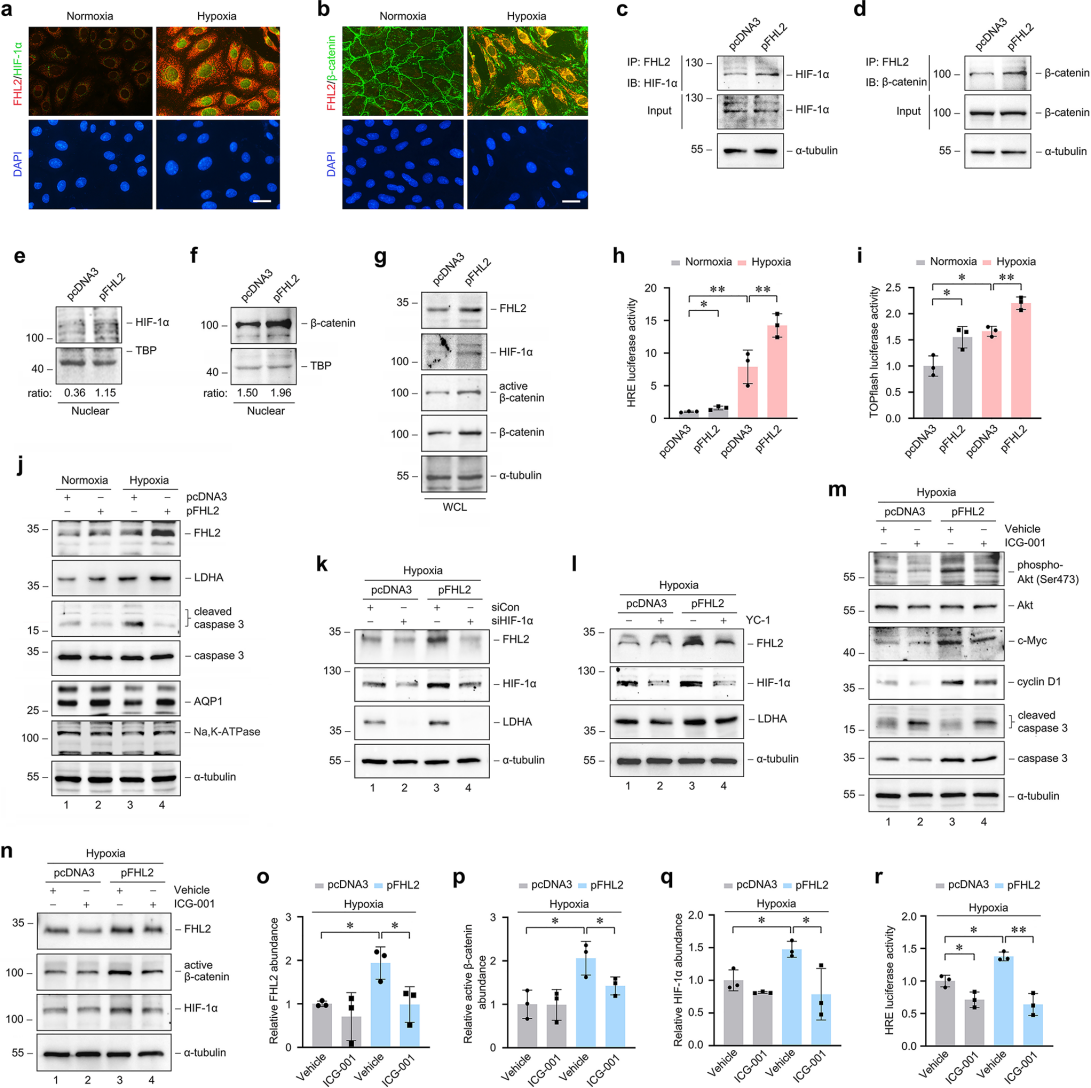
Overexpression of FHL2 protects proximal tubular cells against hypoxia-induced injury by activation of HIF-1 and β-catenin signaling in vitro. NRK-52E cells were used. **a, b** Representative micrographs of immunofluorescence staining exhibit the co-localization of FHL2 with HIF-1α **a** or β‐catenin **b** in cells at 12 h of hypoxia compared with nomorxia controls. Scale bar, 25 μm. **c, d** Co-immunoprecipitation (IP) reveals that ectopic expression of FHL2 induced FHL2/HIF-1α **c** and FHL2/β-catenin **d** complex formation. Cells were transiently transfected with FHL2 expression vector (pFHL2) or empty vector (pcDNA3) for 6 h, and the cell lysate was immunoprecipitated with antibody against FHL2, followed by immunoblotting (IB) for HIF-1α **c** or β-catenin **d**, respectively. **e-g** Ectopic expression of FHL2 induced the stabilization **g** and nuclear translocation of HIF-1α **e** and β-catenin **f**. After 48 h of transfection with pFHL2 or pcDNA3, nuclear protein preparation **e, f** and whole cell lysate (WCL) **g** were immunoblotted with antibodies as indicated. The ratio of HIF-1α **e** or β-catenin **f** per control protein (TBP as nuclear protein) is shown. **h, i** Cells were co-transfected with HRE **h** or TOPflash **i** reporter plasmid and either pFHL2 or pcDNA3 and then treated with or without hypoxia for 12 h. Relative luciferase activities are reported. *P < 0.05, **P < 0.01 (*n* = 3). **j** Cells were transiently transfected with either pFHL2 or pcDNA3 for 6 h and then treated with or without hypoxia for 12 h. Western blot analyses show the abundance of FHL2, LDHA, cleaved caspase 3, AQP1 and Na, K-ATPase. **k** Cells were co-transfected with pFHL2 or pcDNA3 and control (siCon) or HIF-1α (siHIF-1α) siRNA, followed by treating with hypoxia for 12 h. **l** Cells were transiently transfected with pFHL2 or pcDNA3, pretreated with or without YC-1 for 30 min, and then treated with hypoxia for 12 h. **m, n** Cells were transiently transfected with pFHL2 or pcDNA3, pretreated with or without ICG-001 for 30 min, and then treated with hypoxia for 12 h. **m** Western blot analyses show the abundance of phosphorylated Akt (Ser473), c-Myc, cyclin D1 and cleaved caspase 3. **n** Representative western blot shows the abundance of FHL2, active β-catenin and HIF-1α. **o-q** Quantitative analyses data of FHL2 **o**, active β-catenin **p** and HIF-1α **q** are presented. *P < 0.05, **P < 0.01 (*n* = 3). **r** Cells were co-transfected with HRE reporter plasmid and either pFHL2 or pcDNA3, pretreated with or without ICG-001 for 30 min, and then treated with hypoxia for 12 h. Relative luciferase activity is reported. *P < 0.05, **P < 0.01 (*n* = 3). Representative blots of one (**c-g**), two or one (**j-m**) or three (**n**) independent experiments. Data are mean ± SD of three (**h, i, o-r**) independent experiments

To provide direct evidence that links proximal tubular loss of FHL2 to renal damage during IRI, We further explored the mechanism by which FHL2 affects HIF-1 and β-catenin in vitro. Immunostaining revealed an increase in co-localization of FHL2 with HIF-1α (Fig. 6a) and β-catenin (Fig. 6b) in PTCs under hypoxic conditions. To avoid other effects of hypoxia, we examined the effect of ectopic expression of FHL2 on HIF-1 and β-catenin. Overexpression of FHL2 not only induced FHL2 to interact with HIF-1α and β-catenin respectively, as shown by increased FHL2/HIF-1α (Fig. 6c) and FHL2/β-catenin (Fig. 6d) complex formation, but also increased HIF-1α (Fig. 6e) and β-catenin (Fig. 6f) nuclear translocation. Compared with the cells transfected with empty vector pcDNA3, the total abundance of HIF-1α and β-catenin did not change significantly in the cells transfected with FHL2 expression vector for 6 h (Fig. 6c, d), but increased in the cells transfected for 48 h (Fig. 6g). These data imply that the increased FHL2 protein contributes to the stabilization and accumulation of HIF-1α and β-catenin via interacting with them respectively.

Then we investigated the effects of overexpressing FHL2 in PTCs under hypoxic conditions. Luciferase-reporter containing HRE responded not only to hypoxia but also to exogenous FHL2, and a combination of hypoxia and exogenous FHL2 further increased the luciferase-reporter activity (Fig. 6h). Similar result was obtained when TOPflash luciferase-reporter activity was detected (Fig. 6i). Accordingly, western blot analyses showed that overexpression of FHL2 further increased hypoxia-induced upregulation of LDHA (Fig. 6j). More importantly, exogenous FHL2 substantially abolished hypoxia-stimulated upregulation of cleaved caspase 3 (Fig. 6j). PI staining assay showed that exogenous FHL2 largely decrease hypoxia-induced cell death (Supplemental Fig. 1c, d). In addition, downregulation of AQP1 and Na, K-ATPase caused by hypoxia was largely restored by exogenous FHL2 (Fig. 6j). Thus, overexpression of FHL2 in PTCs could further enhance activation of HIF-1 and β-catenin signaling and alleviate cell damage under hypoxic conditions.

We also assessed whether the protective role of FHL2 depended on its regulation of HIF-1 and β-catenin activity. We found that the further upregulation of LDHA induced by exogenous FHL2 under hypoxic conditions was suppressed by knockdown (Fig. 6k, lane 4 *versus* 3) or inhibition (Fig. 6l, lane 4 *versus* 3) of HIF-1α. Similarly, the further upregulation of phosphorylated Akt (Ser473), c-Myc and cyclin D1 induced by exogenous FHL2 under hypoxic conditions was partially restrained by ICG-001, a specific inhibitor of β-catenin [25, 33] (Fig. 6m, lane 4 *versus* 3). Meanwhile, exogenous FHL2 inhibited hypoxia-induced upregulation of cleaved caspase 3 (Fig. 6m, lane 3 *versus* 1), which was restricted by ICG-001 (Fig. 6m, lane 4 *versus* 3). These data suggest that the protection effect of FHL2 depends, at least in part, on its regulation of both signaling in PTCs.

Since FHL2 is identified as a downstream target gene of HIF-1 in present study (Fig. 1) while β-catenin could enhance HIF-1-mediated transcription [26], it is speculated that the expression of FHL2 is also dependent on the activation of β-catenin signaling. We found that the further upregulation of FHL2, HIF-1α and active β-catenin by exogenous FHL2 under hypoxic conditions could be inhibited by ICG-001 (Fig. 6n, lane 4 *versus* 3; 6o-q). Because ICG-001 functions in the nucleus and does not by itself affect the abundance of active β-catenin, the inhibitory effect of ICG-001 on the upregulation of HIF-1α and active β-catenin by exogenous FHL2 should be due to its inhibitory effect on the HIF-1-induced expression of FHL2. These data further emphasize that FHL2 is essential for stabilization of both HIF-1α and β-catenin. Furthermore, the induction of HRE luciferase-reporter activity by exogenous FHL2 under hypoxic conditions was impeded by ICG-001 (Fig. 6r). Altogether, it seems clear that FHL2 involves in the mutual facilitation of the two signaling.

### FHL2 may regulate HIF-1 and β-catenin signaling simultaneously through interacting with GSK-3β in cytoplasm and p300 in nuclei

**Fig. 7:**
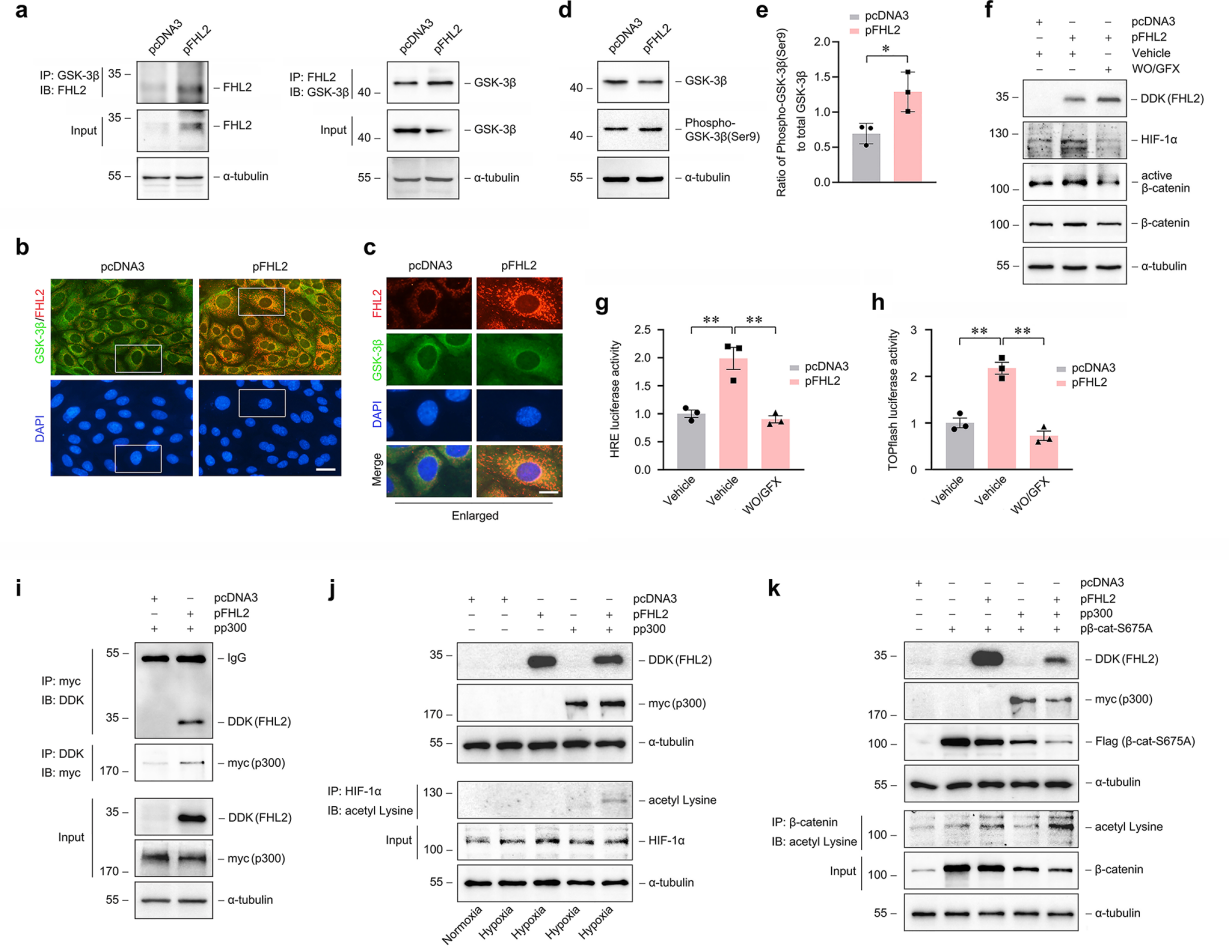
FHL2 may regulate HIF-1 and β-catenin signaling simultaneously through interacting with GSK-3β in the cytoplasm and p300 in the nucleus. **a-h** NRK-52E cells were used. **a-e** Cells were transiently transfected with FHL2 expression vector (pFHL2) or empty vector (pcDNA3) for 6 h. **a** Co-immunoprecipitation (IP) assay reveals that ectopic expression of FHL2 induced FHL2/GSK-3β complex formation. The cell lysate was immunoprecipitated with antibody against GSK-3β or FHL2, followed by immunoblotting (IB) for FHL2 or GSK-3β, respectively. **b, c** Representative micrographs of immunofluorescence staining display the co-localization of FHL2 with GSK-3β. The enlarged box areas from **b** are presented in **c**. Scale bar in b, 25 μm, in c, 15 μm. **d, e** Ectopic expression of FHL2 led to the inhibition of GSK-3β. **d** Representative western blot shows the abundance of total GSK-3β and phosphorylated GSK-3β (Ser9). The ratio of phosphorylated GSK-3β (Ser9) to total GSK-3β protein is presented in **e**. *P < 0.05 (*n* = 3). **f** Western blot analyses show the stabilization of HIF-1α and β-catenin induced by ectopic expression of FHL2 was blocked by GSK-3β activation. Cells were transiently transfected with pFHL2 or pcDNA3 for 6 h, followed by incubation with or without WO/GFX (10µM/10µM) for 12 h. **g, h** Cells were co-transfected with HRE **g** or TOPflash **h** reporter plasmid and either pFHL2 or pcDNA3, followed by incubation with or without WO/GFX (10µM/10µM) for 12 h. Relative luciferase activities are reported. **P < 0.01 (*n* = 3). **i-k** HEK293 cells were used. **i** Co-immunoprecipitation (IP) assay shows that ectopic expression of FHL2 induced FHL2/p300 complex formation. Cells were transiently co-transfected with myc-tagged p300 expression vector (pp300) and either pFHL2 (DDK-tagged) or pcDNA3. The cell lysate was immunoprecipitated with antibody against myc or DDK, followed by immunoblotting (IB) for DDK or myc, respectively. **j, k** IP assay reveals that ectopic expression of FHL2 increased the acetylation of HIF-1α **j** and β‐catenin **k** by p300. **j** Cells were transiently transfected with pcDNA3, pFHL2, pp300 or pFHL2 plus pp300 as indicated, and then treated with hypoxia for 12 h. The cell lysate was immunoprecipitated with antibody against HIF-1α, followed by IB for acetylated Lysine. **k** Cells were transiently co-transfected with active β‐catenin expression vector (pβ‐cat-S675A, Flag-tagged) and pcDNA3, pFHL2, pp300 or pFHL2 plus pp300 as indicated. The cell lysate was immunoprecipitated with antibody against β‐catenin, followed by IB for acetylated Lysine. Representative blots of one (**a, i-k**), two or one (**f**) or three (**d**) independent experiments. Data are mean ± SD of three (**e, g, h**) independent experiments

With our data showing that FHL2 is involved in both signaling, we sought to explore additional regulatory mechanisms. Since GSK-3 could downregulate stability of HIF-1α and β-catenin via targeting them to proteasome by phosphorylation [14, 15], we examined the possibility that FHL2 regulates GSK-3 activity in PTCs. As shown in Fig. 7a-e, overexpression of FHL2 not only induced FHL2 to interact with GSK-3β, as shown by increased FHL2/GSK-3β complex formation (Fig. 7a), and increased co-localization of FHL2 with GSK-3β in cytoplasm displayed by immunostaining (Fig. 7b, c), but also led to an increase in the ratio of phosphorylated (Ser9) GSK-3β to total GSK-3β (Fig. 7d, e), implying an inhibition of GSK-3 activity. Furthermore, the upregulation of HIF-1α and active β-catenin by exogenous FHL2 was largely hindered by WO/GFX, an indirect GSK-3β activator [34] (Fig. 7f). The increase in HRE (Fig. 7g) and TOPflash (Fig. 7h) luciferase-reporter activity induced by exogenous FHL2 was also markedly inhibited by WO/GFX.

As a component of HIF-1α or β-catenin transcriptional complex, p300 positively regulates their mediated transcription through lysine acetylation [14, 15], and FHL2 could enhance β-catenin acetylation by p300 in COS-7 cells [22]. We next investigated the effect of FHL2 on acetylation of HIF-1α or β-catenin by p300 in HEK293 cells. Overexpression of FHL2 induced FHL2 to interact with p300, which was confirmed by increased FHL2/p300 complex formation (Fig. 7i). Interestingly, overexpression of either p300 or FHL2 was not sufficient to obviously increase the acetylation of HIF-1α under hypoxic conditions, whereas overexpression of both p300 and FHL2 substantially increased HIF-1α acetylation (Fig. 7j). Meanwhile, overexpression of both p300 and FHL2 significantly increased the acetylation of β-catenin compared to overexpression of p300 or FHL2 alone (Fig. 7k). Together, FHL2 could regulate HIF-1 and β-catenin simultaneously through interacting with GSK-3β and p300 respectively.

## Discussion

By constructing conditional KO mice with specific deletion of FHL2 in renal PTCs, this study is the first attempt to explore the regulatory role of FHL2 in ischemic AKI. The results demonstrate that the increased FHL2 expression during IRI is renoprotective, because FHL2 deficiency in PTCs leads to more severe renal damage after IRI (Figs. 2g-l and 3). Mechanistically, the ablation of FHL2 in PTCs simultaneously hindered the activation of two important protective signaling, HIF-1 (Fig. 4a-g) and β-catenin (Fig. 5c-f), after IRI. These findings suggest that the induction of FHL2 in renal PTCs is a defense response to attempt to prevent devastating damage after IRI. In vitro data that knockdown of FHL2 inhibited the activation of HIF-1 (Fig. 4h-n) and β-catenin (Fig. 5i-k) signaling further verify that hypoxia-induced activation of both signaling is partly FHL2-dependent.

One of the novel and interesting findings in this study is that hypoxia-induced FHL2 upregulation in PTCs is primarily controlled by HIF-1 signaling. Several data support this conclusion. First, FHL2 expression was upregulated in ischemic AKI and was closely correlated with HIF-1α abundance in mice kidneys during IRI. Second, FHL2 expression was induced by hypoxia in PTCs in vitro, whereas attenuating HIF-1 signaling either by re-oxygenation or YC-1 inhibited FHL2 induction. Third, hypoxia facilitated HIF-1 binding to the HREs in the FHL2 gene promoter. In this context, it is clear that FHL2 is a transcriptional target gene of HIF-1 in PTCs. Given the protective role of HIF-1 signaling in AKI [35, 36], the present study has identified an important protective mechanism by which HIF-1 exerts its actions via evoking FHL2 expression.

Upregulated FHL2 is present throughout the cells during hypoxia (Figs. 1m and 6a and b) and could interact with its protein partners in all intracellular compartments. In this study, proteins that could interact with FHL2 include HIF-1α, β-catenin, GSK-3β, and p300. Since HIF-1α, β-catenin and p300 have been reported as interactors of FHL2 [17, 19, 22, 23], the finding that GSK-3β is a potential protein partner of FHL2 is new. Overexpression of FHL2 could promote HIF-1-mediated transcription in a hypoxia-independent manner (Figs. 6h and 7g), however, this effect was almost completely suppressed by WO/GFX (Fig. 7g), suggesting that the contribution of FHL2 to HIF-1α stability is mainly through inhibiting the phosphorylation of HIF-1α by GSK-3β. The interactions of FHL2 with HIF-1α and FHL2 with GSK-3β may be all involved in this inhibitory effect. Similarly, FHL2 enhances β-catenin stability also through inhibiting the phosphorylation of β-catenin by GSK-3β, and the interactions of FHL2 with β-catenin and FHL2 with GSK-3β may be all involved in this inhibition. Based on current literature search, this is the first report on the interaction of FHL2 with GSK-3β, although the details of molecular domains in the interaction remain to be revealed by further experiments.

**Fig. 8:**
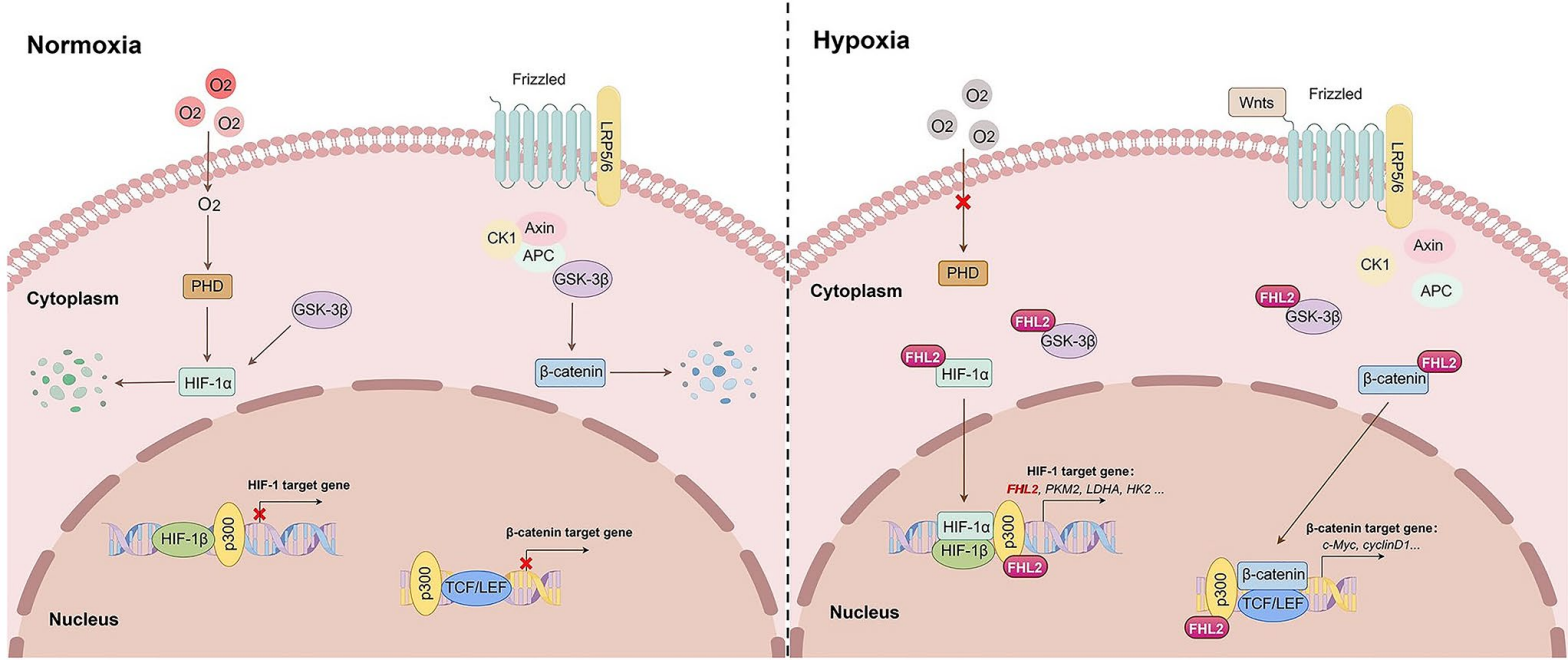
Molecular mechanism of FHL2-mediated renal protection in ischemic AKI. In the proximal tubular cells in an ischemic AKI setting, hypoxia prevents HIF-1α from hydroxylation and degradation while hypoxia-induced upregulation of Wnts prevents β-catenin from phosphorylation and degradation, which allow HIF-1α and β‐catenin stabilization and translocation into nucleus, where HIF-1α dimerizes with HIF-1β while β‐catenin combines with TCF/LEF1, to drive respective target genes transcription for exert protective effects. As a direct downstream target gene of HIF-1, FHL2 is induced under hypoxic condition. Upregulated FHL2 may interact with HIF-1α, β‐catenin, GSK-3β or p300, respectively, which contributes to the stabilization and nuclear translocation of HIF-1α and β‐catenin and promotes HIF-1- and β‐catenin-mediated transcriptional activation, in order to exert a renoprotective effect in ischemic AKI

HIF-1 and β-catenin are mutual reinforced. Hypoxia incited activation of β-catenin (Figs. 5 and 6), in line with previous study [31], whereas β-catenin could enhance HIF-1-mediated transcription [26]. In this study, the upregulation of FHL2, HIF-1α and active β-catenin induced by overexpression of FHL2 could be inhibited by ICG-001, a small molecule that specifically disrupts β-catenin-mediated transcription [33] (Fig. 6o-q), suggesting that FHL2 serves as a mediator strengthening interaction between the two signaling. Although we cannot rule out that other proteins may interact with FHL2, it appears that the increased FHL2 coordinates interactions with multiple proteins mentioned above and is involved in promoting the activation of HIF-1 and β-catenin, ultimately exporting protective effects (Fig. 8).

One of findings worth pointing out is that ectopic expression of FHL2 seems more effective in preventing cell damage caused by hypoxia. Deficiency of FHL2 in PTCs aggravated hypoxia-induced apoptosis (Figs. 3a and b and 5f; Supplemental Fig. 1a, b), indicating the upregulation of endogenous FHL2 is important for alleviating cell death. Nevertheless, the abundance of cleaved caspase 3 was still markedly increased in PTCs under hypoxic conditions (Fig. 5k, lane 3 *versus* 1; 6j, lane 3 *versus* 1), which implies that apoptosis could not be substantially prevented despite the upregulation of endogenous FHL2 and the activation of two protective signaling. Surprisingly, ectopic expression of FHL2 almost blocked the induction of cleaved caspase 3 by hypoxia (Fig. 6j, lane 4 *versus* 3; 6 m, lane 3 *versus* 1) and largely decreased hypoxia-induced cell death (Supplemental Fig. 1c, d), suggesting that exogenous FHL2 produces additional cellular protective effects. Whether exogenous FHL2 can alleviate ischemic AKI remains to be verified by further in vivo experiments.

It must be proposed that sustained activation of either HIF-1 [37–39] or β-catenin [31, 40, 41] can give rise to tubular epithelial-to-mesenchymal transition (EMT) and renal fibrosis. Our previous study showed that upregulation of FHL2 promotes TGF-β1-induced tubular EMT through regulating β-catenin signaling [24]. In this study, the upregulated FHL2 was reduced with the reduced HIF-1α after re-oxygenation (Fig. 1i, j, n), which should be a self-protection mechanism of cells. Given the regulatory effect of FHL2 on activities of both signaling, inappropriate prolonged overexpression of FHL2 may impel the conversion of AKI to chronic kidney disease (CKD). In this regard, FHL2 is also expected to become an effective tool to manipulate two signaling simultaneously, avoiding their persistent activation after AKI and preventing the conversion to CKD.

The major limitation in the current study should be noted. We did not explore the full picture of the proteins with which FHL2 interacts under hypoxia conditions. This may have led to the omission of any more important part of FHL2’s potential role. Since FHL2 may interact with a variety of intracellular protein partners, it may affect other signaling pathways that also play important regulatory roles in IRI, such as REDD1 (regulated in development and DNA damage response 1) or AMPK (adenosine monophosphate-activated protein kinase), both of which function as central mediators of the cellular response to energetic stress including hypoxia. In addition, ectopic expression of FHL2 almost blocked the induction of cleaved caspase 3 by hypoxia and reduced cell death, suggesting the potential actions of FHL2 on the ubiquitin-proteasome system and the highly activated autophagy system in hypoxia. Whether FHL2 can interact with proteins in the signaling pathway mediated by REDD1 or AMPK, or in the ubiquitin-proteasome or autophagy system, remains to be revealed by further experiments. Besides, we should acknowledge that the novelty of the current study is limited since the interaction of FHL2 with HIF-1α or FHL2 with β-catenin has been described before.

In summary, this study represents the first exposition that FHL2, a downstream target gene of HIF-1, is induced in ischemic AKI, and the upregulation of endogenous FHL2 is a protective response to renal damage. The concomitant inhibition of HIF-1 and β-catenin owing to loss of FHL2 in PTCs underscores that FHL2 contributes to the activation of both protective signaling. Therefore, further studies on the role and mechanisms of FHL2 in different stages are warranted to provide new clues for designing rational intervention strategies for AKI.

## Materials and methods

### Mice genotyping and ischemic AKI model

Homozygous FHL2-floxed mice (C57BL/6J background) were ordered from Cyagen Biosciences (Suzhou, China) [25]. Transgenic mice (Strain#:012841, C57BL/6 N background) that expressed Cre recombinase under the control of rat *Ggt1* promoter (Ggt1-Cre) were purchased from Jackson Laboratory (Bar Harbor, ME, USA). By mating FHL2-floxed mice with Ggt1-Cre transgenic mice, conditional knockout (KO) mice that FHL2 gene was specifically deleted in renal cortical PTCs (FHL2^fl/fl^, Cre^+/–^) were generated, and the same gender wildtype mice (FHL2^fl/wt^, Cre^−/−^) from the same litters were used as wildtype (WT) control littermates. Genotyping was performed by a routine PCR protocol using DNA extracted from the mouse tail. The primers used for genotyping were as follows: Ggt1-Cre transgene, sense: 5’-CAG CCT GCT CTA ACG GTT TC-3’ and anti-sense: 5’-CAG GTT CTT GCG AAC CTC AT-3’; and FHL2 genotyping, sense: 5’-AGA AAA GGA ATG CCA GCA ATT CC-3’ and anti-sense: 5’-ACA TAG TAT CAT TGC GTA TAG TTC TTA ACC CA-3’. Male KO and WT mice aged 8 weeks were made AKI model. And at 1 and 3 d after renal IRI, mice were sacrificed and the samples of blood, urine and kidneys were harvested for various analyses.

Male C57BL/6J mice weighing 22–25 g were obtained from Nanjing Medical University Experimental Animal Center. AKI was induced by renal IRI using an established protocol [31]. Briefly, under general anesthesia, a midline abdominal incision was made and bilateral renal pedicles were clipped for 25 min using micro-aneurysm clamps. After removal of the clamps, reperfusion of the kidneys was visually confirmed, then the incision was closed. During the ischemic period, body temperature was maintained between 36 °C and 37 °C using a temperature-controlled system. Mice were sacrificed and the blood and kidneys were harvested at different time points after IRI.

Three sets of animal experiments were performed. Sham-operated mice were used as normal controls. In the first set, groups of mice (*n* = 3) were sacrificed at 0, 1, 3, 6, 12, and 24 h after IRI. In the second set, groups of mice (*n* = 4) were sacrificed at 1, 3, and 7 d after IRI. In the third set, groups of KO mice and WT control littermates (*n* = 6) were sacrificed at 1 and 3 d after IRI.

### Cell culture and treatment

Rat renal tubular proximal epithelial cells (NRK-52E) and human embryonic kidney 293 cells (HEK293) were purchased from the American Type Culture Collection. Cells were cultured in DMEM/F12 medium supplemented with 5% fetal bovine serum (Invitrogen, USA). To establish a model of hypoxia, cells were cultured in an airtight anoxia jar in which a hypoxic environment was maintained using a MGC AnaeroPack system (Mitsubishi Gas Chemical Corporation, Tokyo, Japan) [42]. Specifically, AnaeroPack served as an O_2_ absorber and CO_2_ generator. The sachet of Anaeropack absorbed O_2_ and simultaneously produce approximately 16% or more CO_2_, creating an anaerobic atmosphere. The concentration of O_2_ changed to 0% after about 2 h, and when the concentration of O_2_ is less than 0.1%, the color of the oxygen indicator changed from pink to purple. Cells were incubated under anaerobic conditions for 12 h and then transferred to normal atmosphere for different periods of time.

In some experiments, cells were pretreated with 10µM ICG-001 (A8217, APExBIO, USA), 10µM YC-1 (Y102, Sigma-Aldrich, USA), or a combination (WO/GFX) of 10µM Wortmannin (HY-10197, MCE, USA) and 10µM GF-109203X (HY-13867, MCE) [34, 43] for 30 min, followed by other treatment. ICG-001 is a small molecular peptide that can mimetically compete with β-catenin for binding to CBP, which restrains β-catenin/CBP complex formation, leading to inhibition of β-catenin target genes expression. YC-1 inhibits HIF-1α through inhibiting the translation activity of HIF-1α mRNA, promoting the degradation of HIF-1α and inhibiting the transcriptional activity of HIF-1α. Wortmannin (WO) is a PI3K inhibitor and GF-109203X (GFX) is a PKC inhibitor. Since PI3K/Akt is the upstream target and negatively regulates GSK-3β pathway and activating PKC also inhibits GSK-3β, WO/GFX can serve as an activator of GSK-3β.

Transient transfection of DDK-tagged FHL2 (RR207061, OriGene, USA), Flag-tagged β-cat-S675A (ZT087, Fenghui Biotechnology, China), myc-tagged p300 (79381-2, Gnenchem, China) expression vector, or empty vector pcDNA3 (Invitrogen) was performed using Lipofectamine 3000 reagent (Invitrogen, USA). Transfection of FHL2, HIF-1α or control siRNA (Integrated Biotech Solutions, China) was performed using Lipofectamine 2000 reagent. The sequences of siRNA oligo were as follows: FHL2, sense 5’-GGC AAG AAG UAC AUU CUA AAG-3’ and anti-sense 5’-UUA GAA UGU ACU UCU UGC CAU-3’; HIF-1α, sense 5’-GUU ACG AUU GUG AAG UUA AUG-3’ and anti-sense 5’-UUA ACU UCA CAA UCG UAA CUG-3’; negative control, sense 5’-UUC UCC GAA CGU GUC ACG UTT-3’ and anti-sense 5’-ACG UGA CAC GUU CGG AGA ATT-3’.

### Western blot analysis

Kidney tissue homogenate and cell lysates were prepared by using the routine procedures, and Western blot analysis of protein expression was performed as described previously [44]. The primary antibodies were as follows: anti-HIF-1α (1:500 dilution; ab1, Abcam, USA), anti-FHL2 (1:1000; ab12306, Abcam), anti-active-β-catenin (1:500; 05-665, Millipore, USA), anti-β-catenin (1:1000; 610154, BD Bioscience, USA), anti-Na, K-ATPase (1:1000; ab76020, Abcam), anti-AQP1 (1:1000; AB2219, Millipore), anti-HK2 (1:1000; NBP2-02272, Novus, USA), anti-PKM2 (1:1000; 4053, Cell Signaling Technology (CST), USA), anti-LDHA (1:1000; 3558, CST), anti-p-AKT (1:1000; 4060, CST), anti-AKT (1:1000; 9272, CST), anti-p53 (1:1000; 2524, CST), anti-cyclin D1 (1:1000; 55506, CST), anti-c-Myc (1:1000; ab32072, Abcam), anti-cleaved caspase 3 (1:500; 9664, CST), anti-caspase3 (1:1000; 9662, CST), anti-GSK-3β (1:1000; 9315, CST), anti-p-GSK-3β (1:1000; 9323, CST), anti-acetyl lysine (1:1000; ab22550, Abcam), anti-myc (1:1000; 2276, CST), anti-DDK (1:1000; TA50011, Origene), anti-TBP (1:1000; ab51841, Abcam) and anti-α-tubulin (1:2000; T9026, Sigma-Aldrich). Secondary antibody were anti-Rabbit IgG, HRP conjugate (1:5000; A0545, Sigma- Aldrich) and anti-Mouse IgG, HRP conjugate (1:5000; AP308P, Millipore). Each sample of kidney tissue was loaded with 50 µg and each sample of cells was loaded with 20 µg.

### RNA isolation and real-time RT-PCR

Total RNA was extracted using TRIzol RNA isolation system (Invitrogen), and the first strand of cDNA was synthesized using Reverse transcription kit (Toyobo, Japan). Real-time PCR was performed using ABI PRISM 7300 Sequence Detection system (Applied Biosystems, USA). The sequences of primer pairs are shown in Supplemental Table 1.

### Histology and immunohistochemical staining

Kidney samples were fixed with 10% neutral formalin overnight and then were paraffin-embedded. Sections of 3 μm thickness were prepared for PAS staining according to the established procedures [44]. Immunohistochemical staining of sections was performed by utilizing Vector M.O.M. immunodetection kit (Vector Laboratories, USA), and the primary antibodies used as follows: anti-FHL2 (1:200 dilution; ab12306, Abcam), anti-HIF-1α (1:100; ab1, Abcam), anti-β-catenin (1:100; 610154, BD Bioscience), or Ki67 (1:200; 1882, CST). Nonimmune normal control IgG was used to replace the primary antibody as negative control, and no staining occurred. The representative pictures of negative control staining are given in the Supplemental Fig. 2. Slides were captured on a Nikon Eclipse 80i microscope connected to a digital camera (DS-Ri1, Nikon). The severity of tubular injury was assessed by tubular necrosis, loss of brush border, cast formation, and tubular dilatation. The injury was scored as: 0, ≤ 10% of the injury area stained; 1, 11–25% of stained; 2, 26–50% of stained; 3, 51–75% of stained; and 4, > 75% of stained. At least ten microscope fields (400×) were randomly selected from each section, and an average injury score for each sample was calculated [45]. The number of FHL2-positive and Ki67-positive cells from ten randomly selected fields under microscope (400×) for each sample was counted, and an average number of positive cells for each sample was calculated [44].

### Immunofluorescence staining

Immunofluorescence staining was carried out by using a routine procedure [45]. Cells attached on coverslips were incubated with specific primary antibodies: anti-HIF-1α (1:50 dilution), anti-FHL2 (1:50), anti-β-catenin (D10A8) (1:50; 8480, CST), or anti-GSK-3β (1:50; 9315, CST) followed by incubation with tetramethyl rhodamine- or FITC-conjugated secondary antibody. Cell nuclei were counterstained with 4’,6-diamidino-2-phenylindole-HCl. Stained cells were mounted and viewed with a Nikon Eclipse 80i Epi-fluorescence microscope attached a digital camera (DS-Ri1, Nikon). Nonimmune normal control IgG was used to replace the primary antibody as negative control, and no staining occurred. The representative pictures of negative control staining are given in the Supplemental Fig. 2.

### TUNEL staining

For determination of apoptosis in the kidney tissue, terminal deoxynucleotidyl transferase-mediated dUTP nick-end labeling (TUNEL) staining was carried out by utilizing Apoptosis Detection System (G3250, Promega) according to the manufacturer’s instructions. The TUNEL-positive cells were semi-quantitative analyzed.

### Coimmunoprecipitation assay

Cells were lysed with lysis buffer (Beyotime, Shanghai, China) containing 1% protease inhibitor cocktail and 1% phosphatase inhibitor cocktail I and II (Sigma-Aldrich) on ice. After preclearing with normal IgG, cell lysates were incubated with anti-β-catenin (8480, CST), anti-HIF-1α (ab1, Abcam), anti-GSK-3β (9315, CST), anti-FHL2 (sc-13409, Santa Cruz), anti-myc (2276, CST), or anti-DDK (TA50011, Origene) at 4 °C overnight, followed by precipitation with protein A/G Plus-agarose for 4 h. The precipitated complexes were analyzed by Western blotting with specific antibodies.

### Chromatin immunoprecipitation (ChIP)

ChIP assay was performed by using a ChIP assay kit (26157, Fisher Scientific). Anti-HIF-1α (14179, CST) was used to detect HIF-1 that interacts with the putative HREs in rat FHL2 gene promoter. ChIP samples were used as templates for real-time PCR. Primer sets P1/P2 encompass the region of FHL2 promoter containing two putative HREs, and the sequences of primers are given in Fig. 1r.

### Nuclear and cytoplasmic fractionation

Nuclear protein was extracted using NEPER Nuclear and Cytoplasmic Extraction Reagents (SJ252790, Fisher Scientific) on the basis of the protocols provided by the manufacturer.

### Transfection and luciferase assay

Cells were transfected with either HRE (pGL4.42, Promega) or TOPflash (Millipore) reporter plasmid, and an internal control reporter plasmid Renilla reniformis luciferase driven under thymidine kinase promoter (pRL-TK) was co-transfected for normalizing transfection efficiency. Luciferase assay was performed using a dual luciferase assay system kit (E1910, Promega).

### Serum creatinine, BUN, urinary albumin and NGAL assay

Serum creatinine, BUN, urinary albumin and NGAL level was determined using QuantiChrom Urea (DIUR-500) and Creatinine (DICT-500) assay kit (BioAssay Systems, USA), Exocell Albuwell M kit (NC9182134, Fisher Scientific) and Quantikine Elisa kit (MLCN20, R&D Systems), respectively.

### Lactate and pyruvate assay

Lactate and pyruvate concentration was measured using Lactate (K607, BioVision, USA) and Pyruvate assay kit (K609, BioVision).

### Propidium iodide (PI) staining assay

PI staining was employed to determine dead cells (E-CK-A161, Elabscience, China) according to the manufacturer’s instruction. Cells were stained with PI staining at 37 °C in the dark for 30 min. Fluorescence microscopy was used to verify the dead cells.

### Statistical analysis

Quantitative data were presented as mean ± standard. Statistical analysis of the data was performed using SigmaStat software (Jandel Scientific Software). The unpaired Student’s test was used to compare two groups. ANOVA was used to compare more than two groups. *P* ˂ 0.05 was considered to represent a significant difference.

### Electronic supplementary material

Below is the link to the electronic supplementary material.

Supplementary Material 1

Supplementary Material 2

Supplementary Material 3

Supplementary Material 4

Supplementary Material 5

## Data Availability

All data necessary for confirming the conclusions are included in this article. The datasets generated and/or analyzed during the current study are available from the author upon reasonable request.
