# Supplementary figures and images for: Proximal tubular FHL2, a novel downstream target of hypoxia inducible factor 1, is a protector against ischemic acute kidney injury
Source: Cell Mol Life Sci. 2024 May 30;81(1):244. doi: 10.1007/s00018-024-05289-x (PMC11139843; doi:10.1007/s00018-024-05289-x)

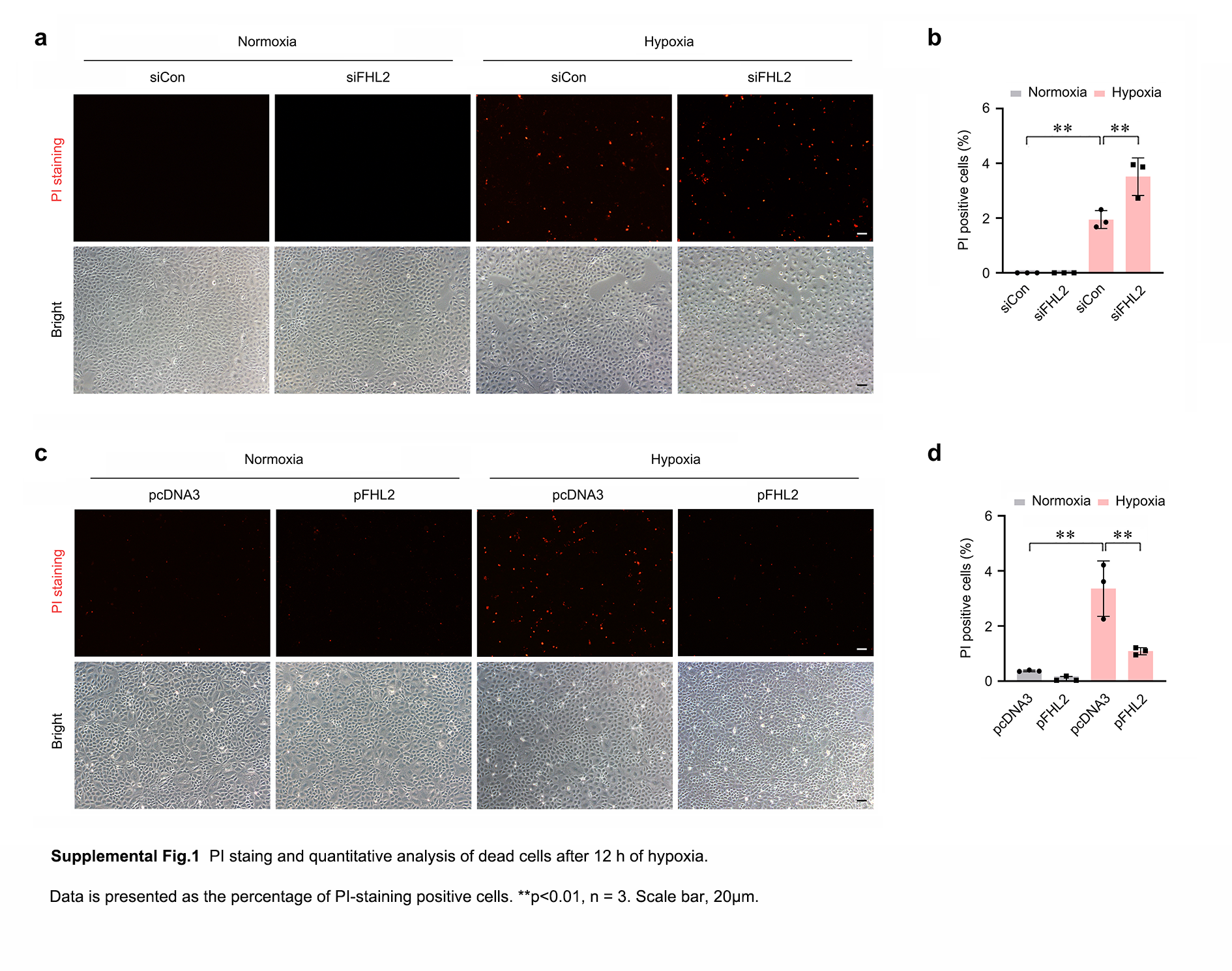

Supplement: Supplementary file 1 — Supplementary Material 1 [file 18_2024_5289_MOESM1_ESM.tif]

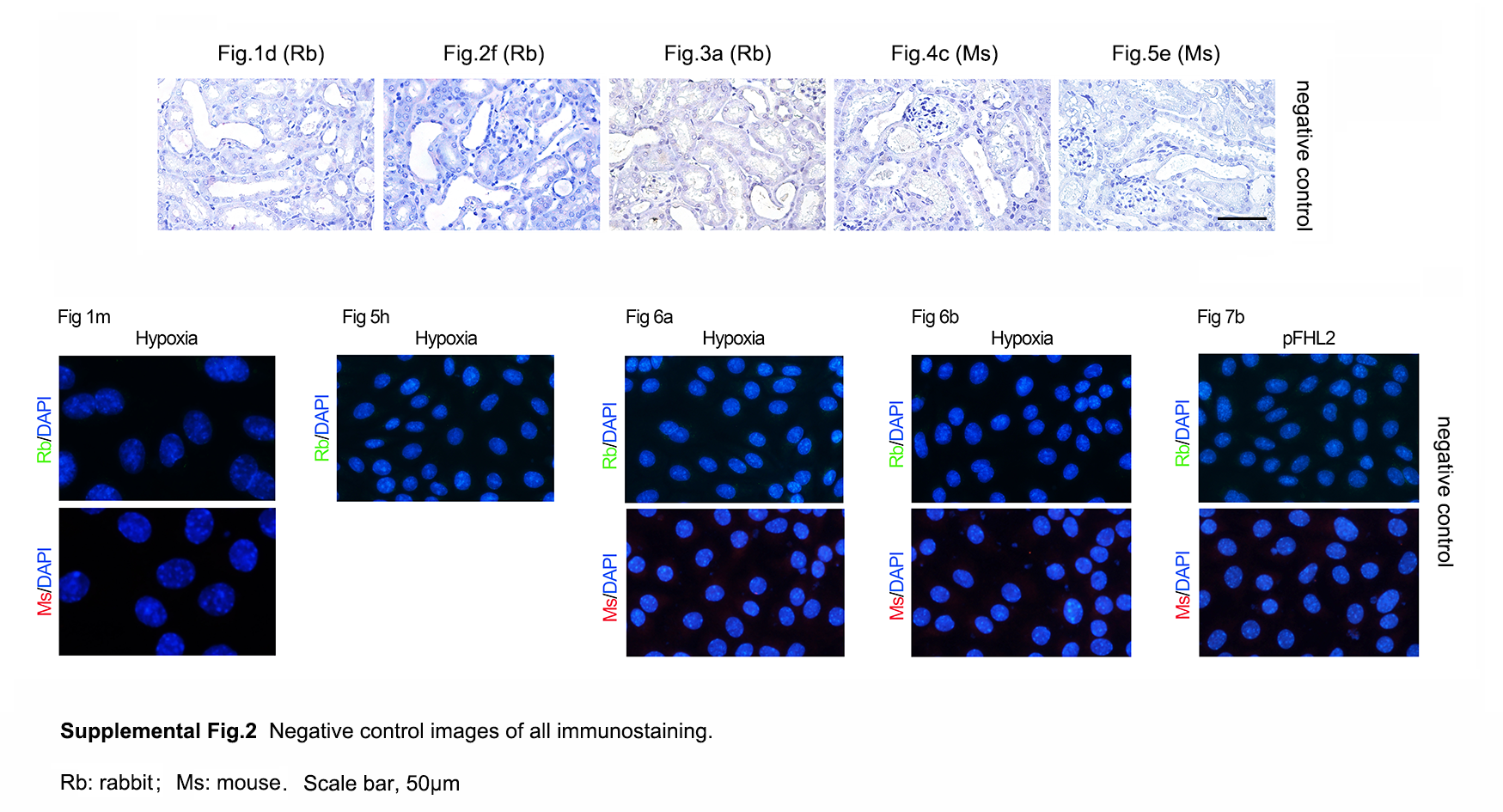

Supplement: Supplementary file 2 — Supplementary Material 2 [file 18_2024_5289_MOESM2_ESM.tif]

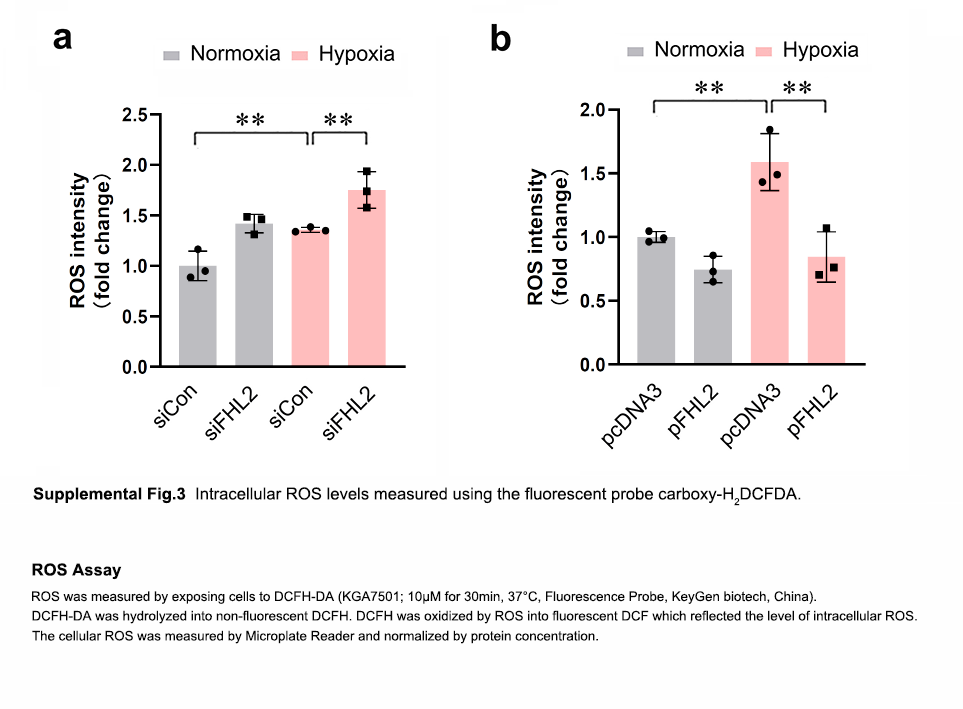

Supplement: Supplementary file 3 — Supplementary Material 3 [file 18_2024_5289_MOESM3_ESM.tif]
