# Supplementary material for: Proximal tubular FHL2, a novel downstream target of hypoxia inducible factor 1, is a protector against ischemic acute kidney injury
Source: Cell Mol Life Sci. 2024 May 30;81(1):244. doi: 10.1007/s00018-024-05289-x (PMC11139843; doi:10.1007/s00018-024-05289-x)
Supplement: Supplementary file 4 — Supplementary Material 4 [file 18_2024_5289_MOESM4_ESM.pdf]

Supplemental Table 1. The sequence of primer pairs for real-time RT-PCR, the expected size of the amplicon, the annealing temperature, and Genbank accession number.

Gene	Forward	Reverse	Size of amplicon	Annealing temperature	Genbank accession number
mouse *FHL2*	5’- CACCGACTGCTATTCCAACG -3’	5’- GTGAAGCGTTGCCCAGATAG -3’	142 bp	51℃	NM_010212
mouse *LDHA*	5’- TGTCTCCAGCAAAGACTACTGT -3’	5’- GACTGTACTTGACAATGTTGGGA -3’	155 bp	60℃	NM_001136069
mouse *PKM2*	5’- GTCTGGAGAAACAGCCAAGG -3’	5’- CGGAGTTCCTCGAATAGCTG -3’	114 bp	58℃	NM_001253883
mouse *PDK1*	5’- GGACTTCGGGTCAGTGAATGC -3’	5’- CTGAGAAGATTGTCGGGGA -3’	120 bp	60℃	NM_172665
mouse *β-actin*	5’- GGCTGTATTCCCCTCCATCG -3’	5’- CCAGTTGGTAACAATGCCATGT -3’	154 bp	60℃	NM_007393
rat *FHL2*	5’- GGACATCTGAAGGCGACACA-3’	5’- GCAAATCCCAGCTCTCCCTT-3’	360 bp	50℃	NM_031677
rat *LDHA*	5’- TTGGTCCAGCGAAACGTGAA-3’	5’- CTTCCAAGCCACGTAGGTCAAGA-3’	123 bp	60℃	NM_017025
rat *PKM2*	5’- ACCTGGGCATTGAGATTCCG-3’	5’- TCGCGCAAGCTCTTCAAACA-3’	314 bp	60℃	NM_053297
rat *HK2*	5’- GATGGAATCGAGAAGGCCTA-3’	5’- GTTTCTTGTAGACGGAGCCA-3’	220 bp	57℃	NM_012735
rat *β-actin*	5’-CGTAAAGACCTCTATGCCAACA-3’	5’-GGAGGAGCAATGATCTTGATCT-3’	131 bp	58℃	NM_031144

FHL2, four-and-a-half LIM domain protein 2; LDHA, lactate dehydrogenase A; PKM2, pyruvate kinase M2; PDK1, pyruvate dehydrogenase kinase 1; HK2, hexokinase 2.

The absence of non-specific results has been verified by blasting the primers. The validations of each pair of primers in PRIMER BLAST are as follows:

Mouse *FHL2*

Primer pair 1

	Sequence (5'->3')	Length	Tm	GC%	Self complementarity	Self 3' complementarity
**Forward primer**	ATGACTGAACGCTTTGACTGC	21	59.47	47.62	4.00	2.00
**Reverse primer**	CGATGGGTGTTCCACACTCC	20	60.67	60.00	5.00	5.00

Products on target templates

>NM_001412598.1 Mus musculus four and a half LIM domains 2 (Fhl2), transcript variant 3, mRNA

```
product length = 142
Forward primer  1    ATGACTGAACGCTTTGACTGC  21
Template        91   .....................  111

Reverse primer  1    CGATGGGTGTTCCACACTCC  20
Template        232  ....................  213
```

>NM_010212.5 Mus musculus four and a half LIM domains 2 (Fhl2), transcript variant 1, mRNA

```
product length = 142
Forward primer  1    ATGACTGAACGCTTTGACTGC  21
Template        257  .....................  277

Reverse primer  1    CGATGGGTGTTCCACACTCC  20
Template        398  ....................  379
```

>NM_001289533.2 Mus musculus four and a half LIM domains 2 (Fhl2), transcript variant 2, mRNA

```
product length = 142
Forward primer  1    ATGACTGAACGCTTTGACTGC  21
Template        135  .....................  155

Reverse primer  1    CGATGGGTGTTCCACACTCC  20
Template        276  ....................  257
```

Mouse *LDHA*

Primer pair 1

	Sequence (5'->3')	Length	Tm	GC%	Self complementarity	Self 3' complementarity
**Forward primer**	TGTCTCCAGCAAAGACTACTGT	22	59.03	45.45	4.00	3.00
**Reverse primer**	GACTGTACTTGACAATGTTGGGA	23	58.61	43.48	5.00	2.00

Products on target templates

>XM_030242176.1 PREDICTED: Mus musculus lactate dehydrogenase A (Ldha), transcript variant X1, mRNA

```
product length = 155
Forward primer  1     TGTCTCCAGCAAAGACTACTGT  22
Template        3781  ......................  3802

Reverse primer  1     GACTGTACTTGACAATGTTGGGA  23
Template        3935  .......................  3913
```

>NM_001136069.2 Mus musculus lactate dehydrogenase A (Ldha), transcript variant 2, mRNA

```
product length = 155
Forward primer  1    TGTCTCCAGCAAAGACTACTGT  22
Template        562  ......................  583

Reverse primer  1    GACTGTACTTGACAATGTTGGGA  23
Template        716  .......................  694
```

>NM_010699.2 Mus musculus lactate dehydrogenase A (Ldha), transcript variant 1, mRNA

```
product length = 155
Forward primer  1    TGTCTCCAGCAAAGACTACTGT  22
Template        369  ......................  390

Reverse primer  1    GACTGTACTTGACAATGTTGGGA  23
Template        523  .......................  501
```

Mouse *PKM2*

Primer pair 1

	Sequence (5'->3')	Length	Tm	GC%	Self complementarity	Self 3' complementarity
**Forward primer**	GTCTGGAGAAACAGCCAAGG	20	58.47	55.00	4.00	2.00
**Reverse primer**	CGGAGTTCCTCGAATAGCTG	20	57.87	55.00	4.00	2.00

Products on target templates

>XM_006510856.3 PREDICTED: Mus musculus pyruvate kinase, muscle (Pkm), transcript variant X6, mRNA

```
product length = 114
Forward primer  1     GTCTGGAGAAACAGCCAAGG  20
Template        4575  ....................  4594

Reverse primer  1     CGGAGTTCCTCGAATAGCTG  20
Template        4688  ....................  4669
```

>XM_036154691.1 PREDICTED: Mus musculus pyruvate kinase, muscle (Pkm), transcript variant X3, mRNA

```
product length = 114
Forward primer  1     GTCTGGAGAAACAGCCAAGG  20
Template        2747  ....................  2766

Reverse primer  1     CGGAGTTCCTCGAATAGCTG  20
Template        2860  ....................  2841
```

>XM_036154690.1 PREDICTED: Mus musculus pyruvate kinase, muscle (Pkm), transcript variant X2, mRNA

```
product length = 114
Forward primer  1     GTCTGGAGAAACAGCCAAGG  20
Template        3099  ....................  3118

Reverse primer  1     CGGAGTTCCTCGAATAGCTG  20
Template        3212  ....................  3193
```

>NM_001378868.1 Mus musculus pyruvate kinase, muscle (Pkm), transcript variant 5, mRNA

```
product length = 114
Forward primer  1     GTCTGGAGAAACAGCCAAGG  20
Template        1287  ....................  1306

Reverse primer  1     CGGAGTTCCTCGAATAGCTG  20
Template        1400  ....................  1381
```

>NM_011099.4 Mus musculus pyruvate kinase, muscle (Pkm), transcript variant 2, mRNA

```
product length = 114
Forward primer  1     GTCTGGAGAAACAGCCAAGG  20
Template        1170  ....................  1189

Reverse primer  1     CGGAGTTCCTCGAATAGCTG  20
Template        1283  ....................  1264
```

>NM_001378869.1 Mus musculus pyruvate kinase, muscle (Pkm), transcript variant 6, mRNA

```
product length = 114
Forward primer  1     GTCTGGAGAAACAGCCAAGG  20
Template        1167  ....................  1186

Reverse primer  1     CGGAGTTCCTCGAATAGCTG  20
Template        1280  ....................  1261
```

>NM_001378870.1 Mus musculus pyruvate kinase, muscle (Pkm), transcript variant 7, mRNA

```
product length = 114
Forward primer  1     GTCTGGAGAAACAGCCAAGG  20
Template        1140  ....................  1159

Reverse primer  1     CGGAGTTCCTCGAATAGCTG  20
Template        1253  ....................  1234
```

>XM_011242674.2 PREDICTED: Mus musculus pyruvate kinase, muscle (Pkm), transcript variant X1, mRNA

```
product length = 114
Forward primer  1     GTCTGGAGAAACAGCCAAGG  20
Template        2786  ....................  2805

Reverse primer  1     CGGAGTTCCTCGAATAGCTG  20
Template        2899  ....................  2880
```

>XM_006510855.1 PREDICTED: Mus musculus pyruvate kinase, muscle (Pkm), transcript variant X4, mRNA

```
product length = 114
Forward primer  1     GTCTGGAGAAACAGCCAAGG  20
Template        1324  ....................  1343

Reverse primer  1     CGGAGTTCCTCGAATAGCTG  20
Template        1437  ....................  1418
```

Mouse *PDK1*

Primer pair 1

	Sequence (5'->3')	Length	Tm	GC%	Self complementarity	Self 3' complementarity
**Forward primer**	GGACTTCGGGTCAGTGAATGC	21	61.27	57.14	5.00	2.00
**Reverse primer**	CTGAGAAGATTGTCGGGGA	19	56.14	52.63	4.00	1.00

Products on target templates

>XM_011239444.3 PREDICTED: Mus musculus pyruvate dehydrogenase kinase, isoenzyme 1 (Pdk1), transcript variant X1, mRNA

```
product length = 120
Forward primer  1    GGACTTCGGGTCAGTGAATGC  21
Template        505  .....................  525

Reverse primer  1    CTGAGAAGATTGTCGGGGA  19
Template        624  ...................  606
```

>NM_172665.5 Mus musculus pyruvate dehydrogenase kinase, isoenzyme 1 (Pdk1), transcript variant 1, mRNA; nuclear gene for mitochondrial product

```
product length = 120
Forward primer  1    GGACTTCGGGTCAGTGAATGC  21
Template        335  .....................  355

Reverse primer  1    CTGAGAAGATTGTCGGGGA  19
Template        454  ...................  436
```

>XM_011239446.4 PREDICTED: Mus musculus pyruvate dehydrogenase kinase, isoenzyme 1 (Pdk1), transcript variant X3, mRNA

```
product length = 120
Forward primer  1    GGACTTCGGGTCAGTGAATGC  21
Template        239  AT.GAC...............  259

Reverse primer  1    CTGAGAAGATTGTCGGGGA  19
Template        358  ...................  340
```

>NM_001360002.1 Mus musculus pyruvate dehydrogenase kinase, isoenzyme 1 (Pdk1), transcript variant 2, mRNA; nuclear gene for mitochondrial product

```
product length = 120
Forward primer  1    GGACTTCGGGTCAGTGAATGC  21
Template        337  AT.GAC...............  357

Reverse primer  1    CTGAGAAGATTGTCGGGGA  19
Template        456  ...................  438
```

Mouse *β-actin*

Primer pair 1

	Sequence (5'->3')	Length	Tm	GC%	Self complementarity	Self 3' complementarity
**Forward primer**	CGTAAAGACCTCTATGCCAACA	22	58.14	45.45	4.00	1.00
**Reverse primer**	GGAGGAGCAATGATCTTGATCT	22	57.65	45.45	8.00	6.00

Products on target templates

>NM_007393.5 Mus musculus actin, beta (Actb), mRNA

```
product length = 131
Forward primer  1     CGTAAAGACCTCTATGCCAACA  22
Template        977   ......................  998

Reverse primer  1     GGAGGAGCAATGATCTTGATCT  22
Template        1107  ......................  1086
```

>NM_177093.3 Mus musculus leucine rich repeat containing 58 (Lrrc58), mRNA

```
product length = 131
Forward primer  1     CGTAAAGACCTCTATGCCAACA  22
Template        3111  .ACC....T.............  3132

Reverse primer  1     GGAGGAGCAATGATCTTGATCT  22
Template        3241  ......................  3220
```

>NM_009608.4 Mus musculus actin, alpha, cardiac muscle 1 (Actc1), mRNA

```
product length = 131
Forward primer  1     CGTAAAGACCTCTATGCCAACA  22
Template        1017  ..C........G..........  1038

Reverse primer  1     GGAGGAGCAATGATCTTGATCT  22
Template        1147  ..G........A..........  1126
```

>NM_001313923.1 Mus musculus actin, gamma, cytoplasmic 1 (Actg1), transcript variant 2, mRNA

```
product length = 131
Forward primer  1     CGTAAAGACCTCTATGCCAACA  22
Template        990   ..C........G........T.  1011

Reverse primer  1     GGAGGAGCAATGATCTTGATCT  22
Template        1120  ..G..............A....  1099
```

>NM_009609.3 Mus musculus actin, gamma, cytoplasmic 1 (Actg1), transcript variant 1, mRNA

```
product length = 131
Forward primer  1     CGTAAAGACCTCTATGCCAACA  22
Template        945   ..C........G........T.  966

Reverse primer  1     GGAGGAGCAATGATCTTGATCT  22
Template        1075  ..G..............A....  1054
```

>NM_007392.3 Mus musculus actin alpha 2, smooth muscle, aorta (Acta2), mRNA

```
product length = 131
Forward primer  1     CGTAAAGACCTCTATGCCAACA  22
Template        981   A.G..G..T........T....  1002

Reverse primer  1     GGAGGAGCAATGATCTTGATCT  22
Template        1111  .....G................  1090
```

>NM_001272041.1 Mus musculus actin alpha 1, skeletal muscle (Acta1), transcript variant 1, mRNA

```
product length = 131
Forward primer  1     CGTAAAGACCTCTATGCCAACA  22
Template        1060  A.G..G.....G..........  1081

Reverse primer  1     GGAGGAGCAATGATCTTGATCT  22
Template        1190  ..G..G..G.............  1169
```

>NM_009606.3 Mus musculus actin alpha 1, skeletal muscle (Acta1), transcript variant 2, mRNA

```
product length = 131
Forward primer  1     CGTAAAGACCTCTATGCCAACA  22
Template        970   A.G..G.....G..........  991

Reverse primer  1     GGAGGAGCAATGATCTTGATCT  22
Template        1100  ..G..G..G.............  1079
```

Rat *FHL2*

Primer pair 1

	Sequence (5'->3')	Length	Tm	GC%	Self complementarity	Self 3' complementarity
**Forward primer**	GGACATCTGAAGGCGACACA	20	60.04	55.00	3.00	1.00
**Reverse primer**	GCAAATCCCAGCTCTCCCTT	20	60.03	55.00	4.00	0.00

Products on target templates

>XM_039084158.2 PREDICTED: Rattus norvegicus four and a half LIM domains 2 (Fhl2), transcript variant X1, mRNA

```
product length = 360
Forward primer  1     GGACATCTGAAGGCGACACA  20
Template        993   ....................  1012

Reverse primer  1     GCAAATCCCAGCTCTCCCTT  20
Template        1352  ....................  1333
```

>NM_031677.2 Rattus norvegicus four and a half LIM domains 2 (Fhl2), transcript variant 1, mRNA

```
product length = 360
Forward primer  1     GGACATCTGAAGGCGACACA  20
Template        966   ....................  985

Reverse primer  1     GCAAATCCCAGCTCTCCCTT  20
Template        1325  ....................  1306
```

>NM_001412599.1 Rattus norvegicus four and a half LIM domains 2 (Fhl2), transcript variant 2, mRNA

```
product length = 360
Forward primer  1     GGACATCTGAAGGCGACACA  20
Template        1093  ....................  1112

Reverse primer  1     GCAAATCCCAGCTCTCCCTT  20
Template        1452  ....................  1433
```

Rat *LDHA*

Primer pair 1

	Sequence (5'->3')	Length	Tm	GC%	Self complementarity	Self 3' complementarity
**Forward primer**	TTGGTCCAGCGAAACGTGAA	20	60.46	50.00	5.00	1.00
**Reverse primer**	CTTCCAAGCCACGTAGGTCAAGA	23	62.43	52.17	4.00	2.00

Products on target templates

>XM_039082293.2 PREDICTED: Rattus norvegicus lactate dehydrogenase A like 1 (Ldhal1), mRNA

```
product length = 123
Forward primer  1    TTGGTCCAGCGAAACGTGAA  20
Template        431  ....................  450

Reverse primer  1    CTTCCAAGCCACGTAGGTCAAGA  23
Template        553  .......................  531
```

>XM_006229232.5 PREDICTED: Rattus norvegicus lactate dehydrogenase A (Ldha), transcript variant X1, mRNA

```
product length = 123
Forward primer  1    TTGGTCCAGCGAAACGTGAA  20
Template        693  ....................  712

Reverse primer  1    CTTCCAAGCCACGTAGGTCAAGA  23
Template        815  .......................  793
```

>NM_017025.2 Rattus norvegicus lactate dehydrogenase A (Ldha), mRNA

```
product length = 123
Forward primer  1    TTGGTCCAGCGAAACGTGAA  20
Template        426  ....................  445

Reverse primer  1    CTTCCAAGCCACGTAGGTCAAGA  23
Template        548  .......................  526
```

Rat *PKM2*

Primer pair 1

	Sequence (5'->3')	Length	Tm	GC%	Self complementarity	Self 3' complementarity
**Forward primer**	ACCTGGGCATTGAGATTCCG	20	60.11	55.00	3.00	2.00
**Reverse primer**	TCGCGCAAGCTCTTCAAACA	20	61.16	50.00	4.00	2.00

Products on target templates

>XM_063264962.1 PREDICTED: Rattus norvegicus pyruvate kinase M1/2 (Pkm), transcript variant X11, mRNA

```
product length = 314
Forward primer  1     ACCTGGGCATTGAGATTCCG  20
Template        949   ....................  968

Reverse primer  1     TCGCGCAAGCTCTTCAAACA  20
Template        1262  ....................  1243
```

>XM_063264961.1 PREDICTED: Rattus norvegicus pyruvate kinase M1/2 (Pkm), transcript variant X10, mRNA

```
product length = 314
Forward primer  1     ACCTGGGCATTGAGATTCCG  20
Template        969   ....................  988

Reverse primer  1     TCGCGCAAGCTCTTCAAACA  20
Template        1282  ....................  1263
```

>XM_006243190.4 PREDICTED: Rattus norvegicus pyruvate kinase M1/2 (Pkm), transcript variant X8, mRNA

```
product length = 314
Forward primer  1     ACCTGGGCATTGAGATTCCG  20
Template        1174  ....................  1193

Reverse primer  1     TCGCGCAAGCTCTTCAAACA  20
Template        1487  ....................  1468
```

>XM_063264957.1 PREDICTED: Rattus norvegicus pyruvate kinase M1/2 (Pkm), transcript variant X5, mRNA

```
product length = 314
Forward primer  1     ACCTGGGCATTGAGATTCCG  20
Template        1258  ....................  1277

Reverse primer  1     TCGCGCAAGCTCTTCAAACA  20
Template        1571  ....................  1552
```

>XM_063264956.1 PREDICTED: Rattus norvegicus pyruvate kinase M1/2 (Pkm), transcript variant X4, mRNA

```
product length = 314
Forward primer  1     ACCTGGGCATTGAGATTCCG  20
Template        1012  ....................  1031

Reverse primer  1     TCGCGCAAGCTCTTCAAACA  20
Template        1325  ....................  1306
```

>XM_063264955.1 PREDICTED: Rattus norvegicus pyruvate kinase M1/2 (Pkm), transcript variant X3, mRNA

```
product length = 314
Forward primer  1     ACCTGGGCATTGAGATTCCG  20
Template        1012  ....................  1031

Reverse primer  1     TCGCGCAAGCTCTTCAAACA  20
Template        1325  ....................  1306
```

>XM_063264953.1 PREDICTED: Rattus norvegicus pyruvate kinase M1/2 (Pkm), transcript variant X1, mRNA

```
product length = 314
Forward primer  1     ACCTGGGCATTGAGATTCCG  20
Template        1375  ....................  1394

Reverse primer  1     TCGCGCAAGCTCTTCAAACA  20
Template        1688  ....................  1669
```

>NM_053297.2 Rattus norvegicus pyruvate kinase M1/2 (Pkm), mRNA

```
product length = 314
Forward primer  1     ACCTGGGCATTGAGATTCCG  20
Template        969   ....................  988

Reverse primer  1     TCGCGCAAGCTCTTCAAACA  20
Template        1282  ....................  1263
```

Rat *HK2*

Primer pair 1

	Sequence (5'->3')	Length	Tm	GC%	Self complementarity	Self 3' complementarity
**Forward primer**	GATGGAATCGAGAAGGCCTA	20	56.50	50.00	6.00	4.00
**Reverse primer**	GTTTCTTGTAGACGGAGCCA	20	57.55	50.00	3.00	0.00

Products on target templates

>NM_012735.2 Rattus norvegicus hexokinase 2 (Hk2), mRNA

```
product length = 220
Forward primer  1     GATGGAATCGAGAAGGCCTA  20
Template        1235  ....................  1254

Reverse primer  1     GTTTCTTGTAGACGGAGCCA  20
Template        1454  ....................  1435
```

Rat *β-actin*

Primer pair 1

	Sequence (5'->3')	Length	Tm	GC%	Self complementarity	Self 3' complementarity
**Forward primer**	CGTAAAGACCTCTATGCCAACA	22	58.14	45.45	4.00	1.00
**Reverse primer**	GGAGGAGCAATGATCTTGATCT	22	57.65	45.45	8.00	6.00

Products on target templates

>NM_031144.3 Rattus norvegicus actin, beta (Actb), mRNA

```
product length = 131
Forward primer  1    CGTAAAGACCTCTATGCCAACA  22
Template        946  ......................  967

Reverse primer  1     GGAGGAGCAATGATCTTGATCT  22
Template        1076  ......................  1055
```
