# Supplementary material for: Proximal tubular FHL2, a novel downstream target of hypoxia inducible factor 1, is a protector against ischemic acute kidney injury
Source: Cell Mol Life Sci. 2024 May 30;81(1):244. doi: 10.1007/s00018-024-05289-x (PMC11139843; doi:10.1007/s00018-024-05289-x)
Supplement: Supplementary file 5 — Supplementary Material 5 [file 18_2024_5289_MOESM5_ESM.pdf]

Figure 1e

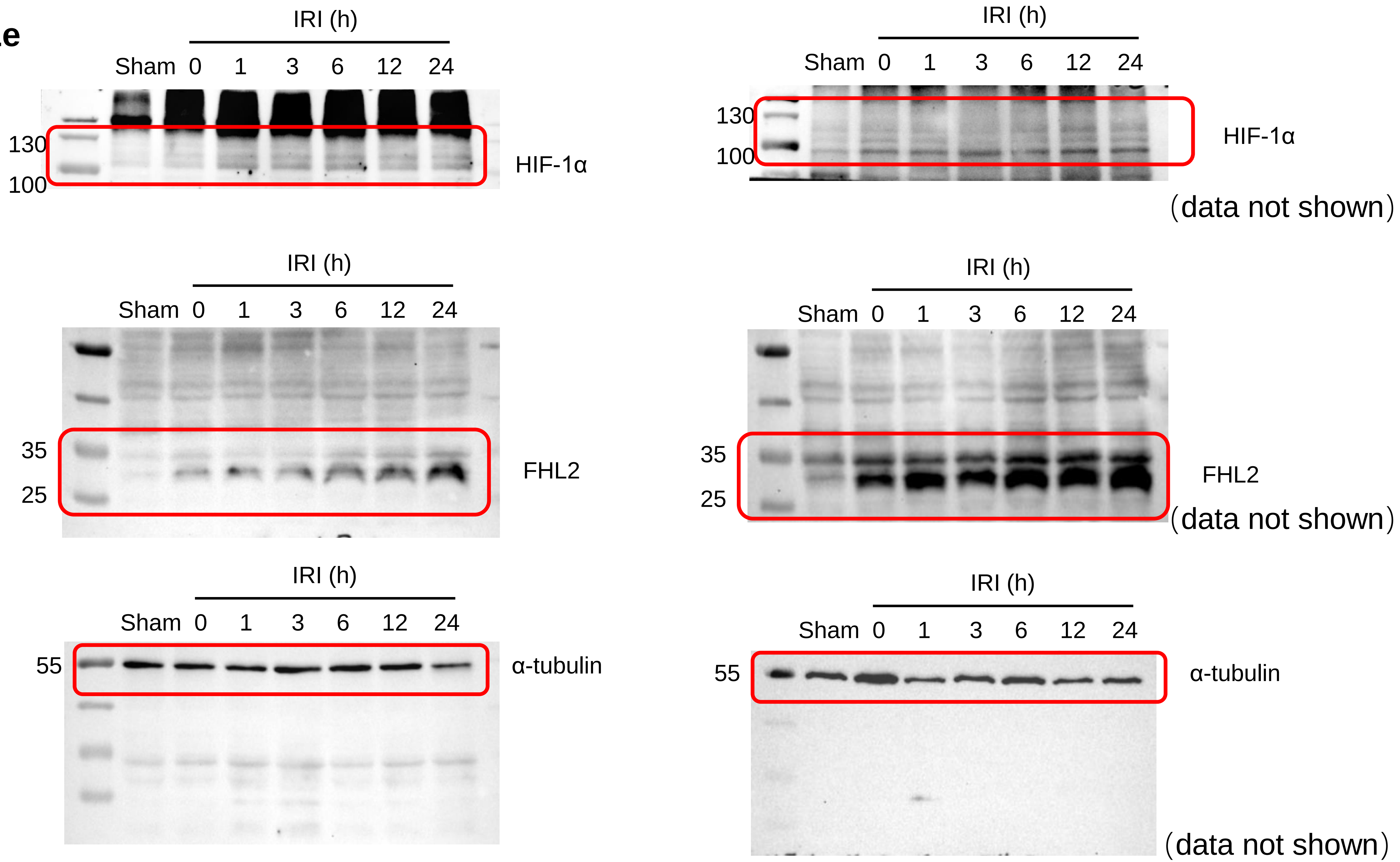

IRI (h)
Sham 0 1 3 6 12 24
130
100
HIF-1α
IRI (h)
Sham 0 1 3 6 12 24
130
100
HIF-1α
(data not shown)
IRI (h)
Sham 0 1 3 6 12 24
35
25
FHL2
IRI (h)
Sham 0 1 3 6 12 24
35
25
FHL2
(data not shown)
IRI (h)
Sham 0 1 3 6 12 24
55
α-tubulin
IRI (h)
Sham 0 1 3 6 12 24
55
α-tubulin
(data not shown)

Figure 1i

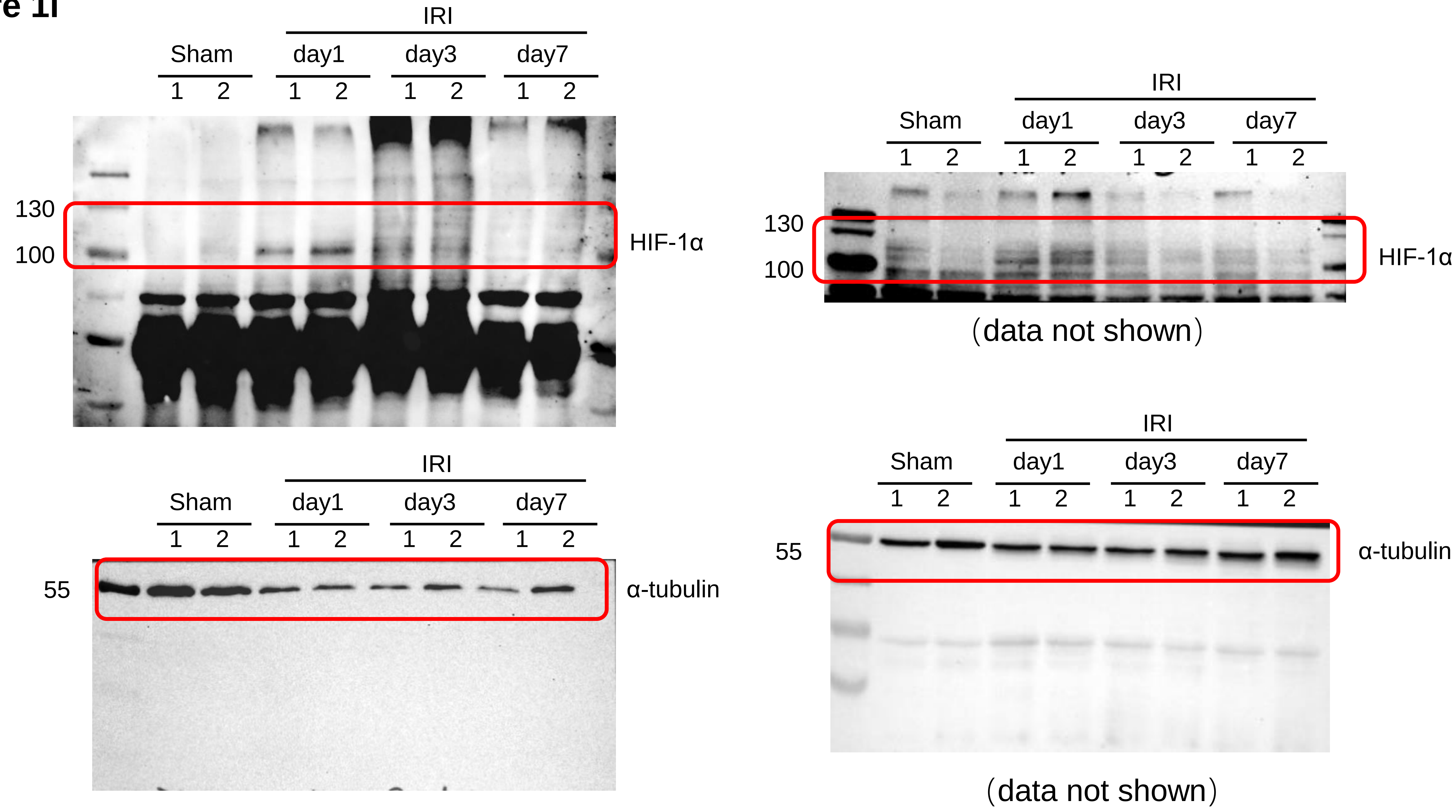

IRI
Sham
day1
day3
day7
1 2 1 2 1 2 1 2
130
100
HIF-1α
55
α-tubulin
(data not shown)

Figure 1i

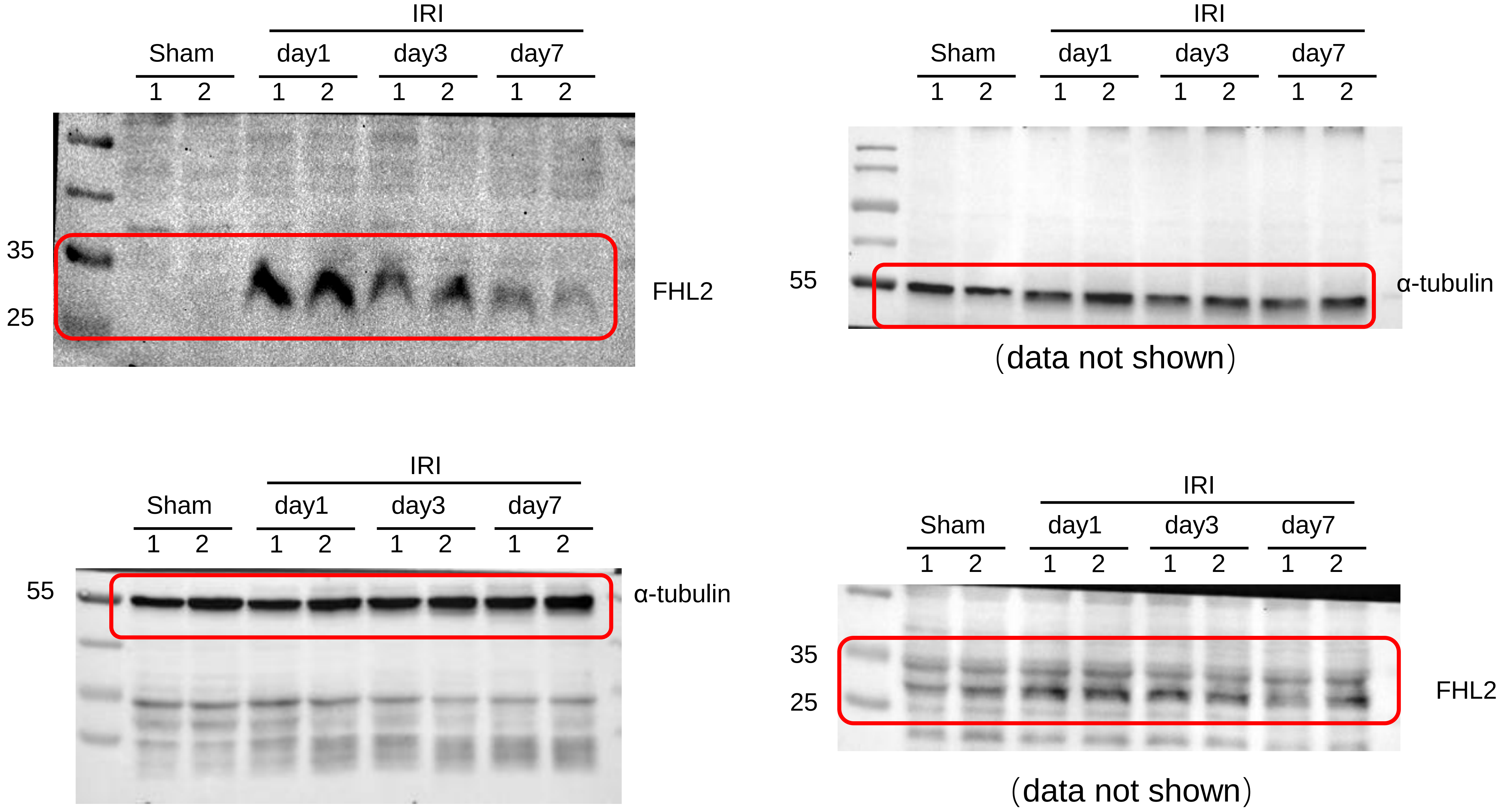

IRI
Sham
day1
day3
day7
1 2 1 2 1 2 1 2
35
25
FHL2
IRI
Sham
day1
day3
day7
1 2 1 2 1 2 1 2
55
α-tubulin
(data not shown)
IRI
Sham
day1
day3
day7
1 2 1 2 1 2 1 2
55
α-tubulin
IRI
Sham
day1
day3
day7
1 2 1 2 1 2 1 2
35
25
FHL2
(data not shown)

Figure 1k

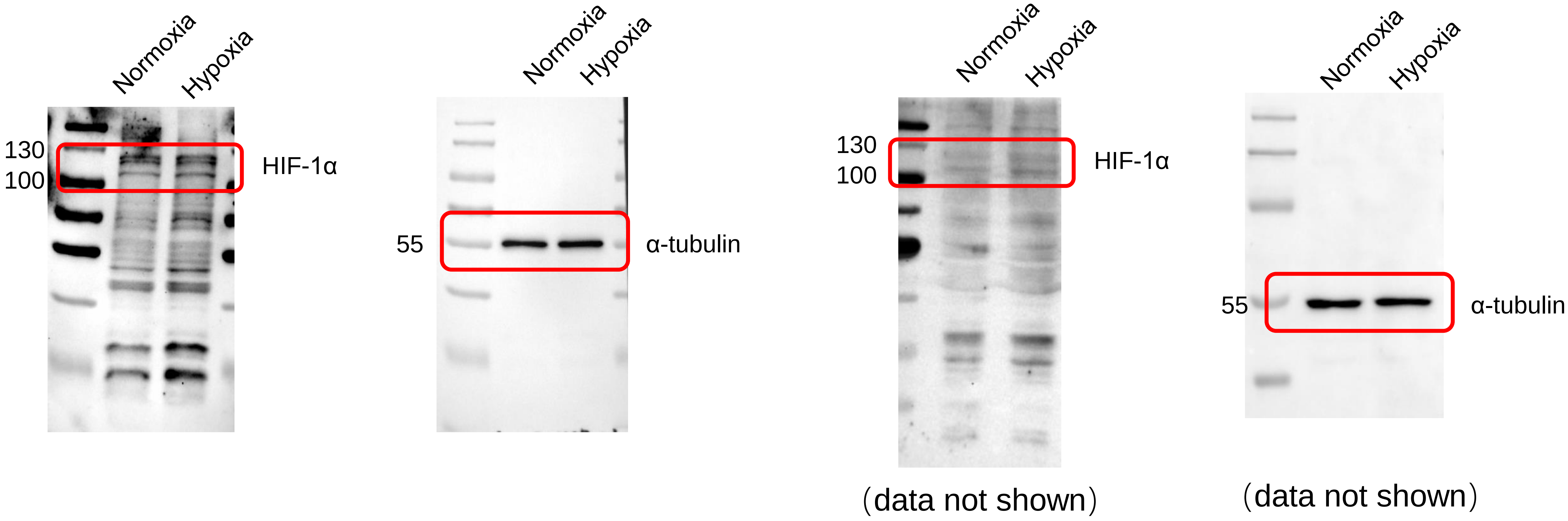

Normoxia
Hypoxia
130
100
HIF-1α
Normoxia
Hypoxia
55
α-tubulin
Normoxia
Hypoxia
130
100
HIF-1α
(data not shown)
Normoxia
Hypoxia
55
α-tubulin
(data not shown)

Figure 1k

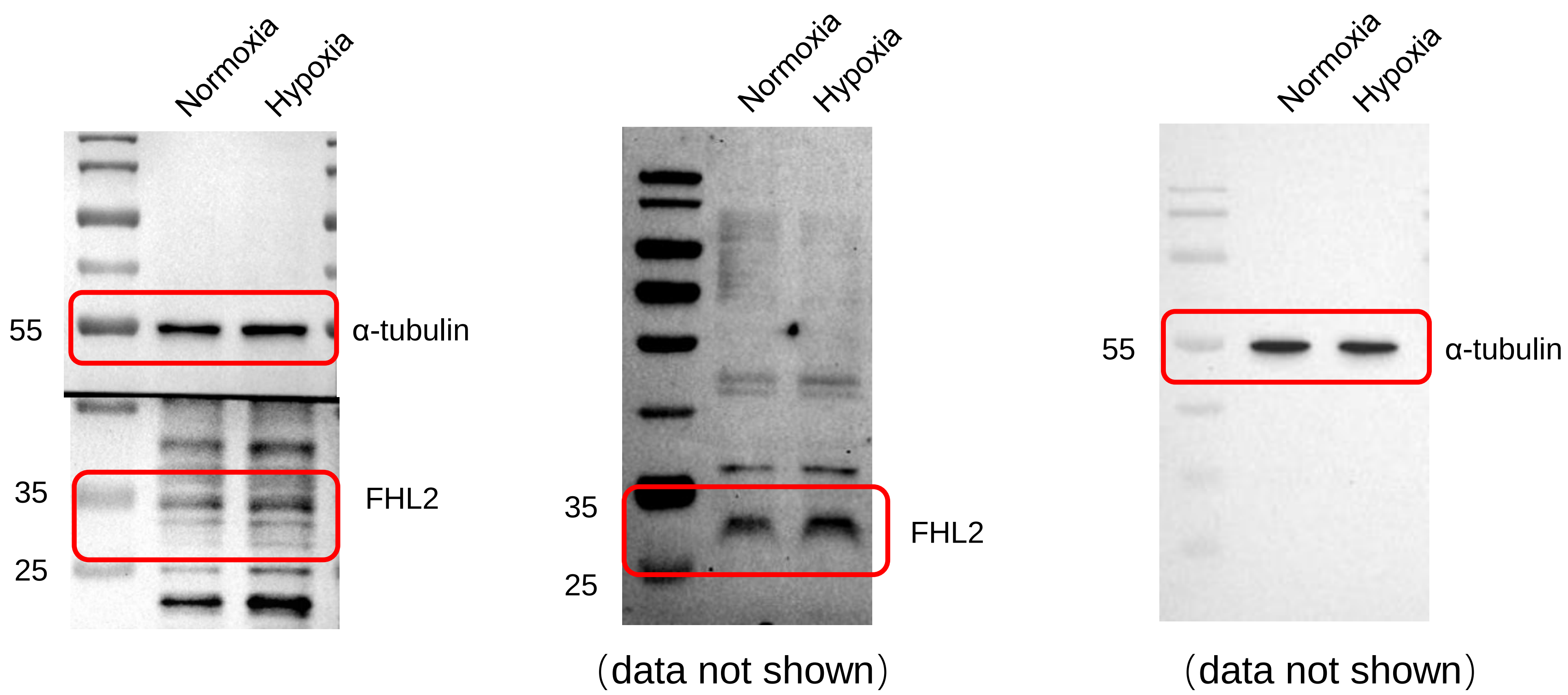

Normoxia
Hypoxia
55
α-tubulin
35
FHL2
25
Normoxia
Hypoxia
35
FHL2
25
(data not shown)
Normoxia
Hypoxia
55
α-tubulin
(data not shown)

Figure 1n

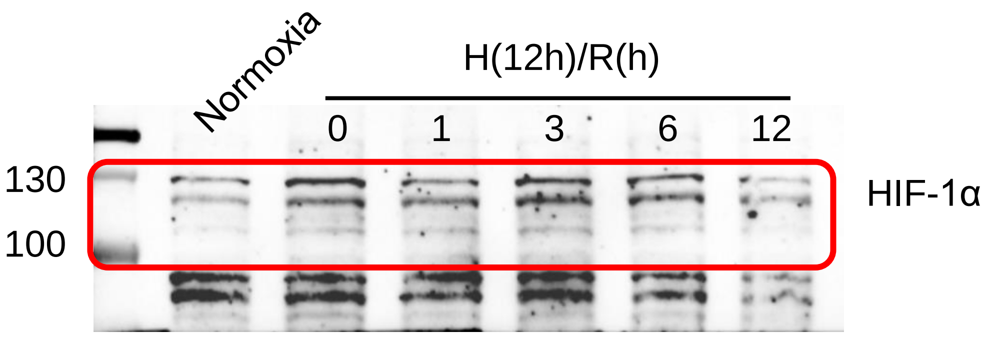
Normoxia
H(12h)/R(h)
0
1
3
6
12
130
100
HIF-1α

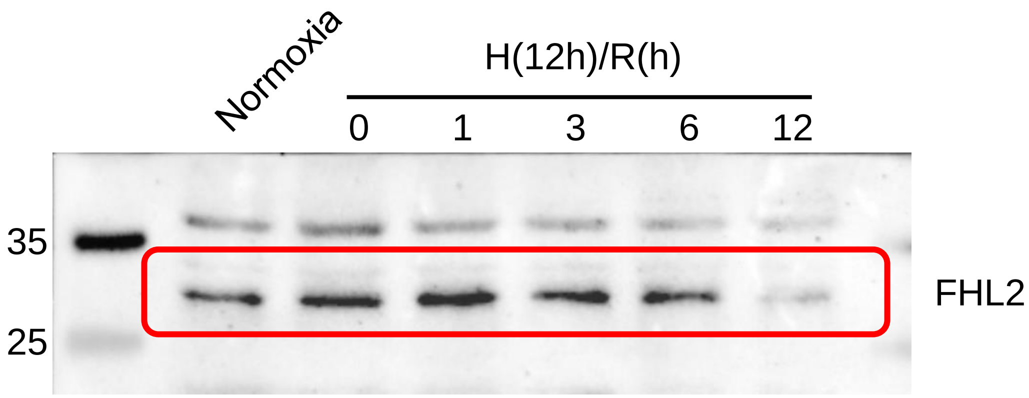
Normoxia
H(12h)/R(h)
0
1
3
6
12
35
25
FHL2

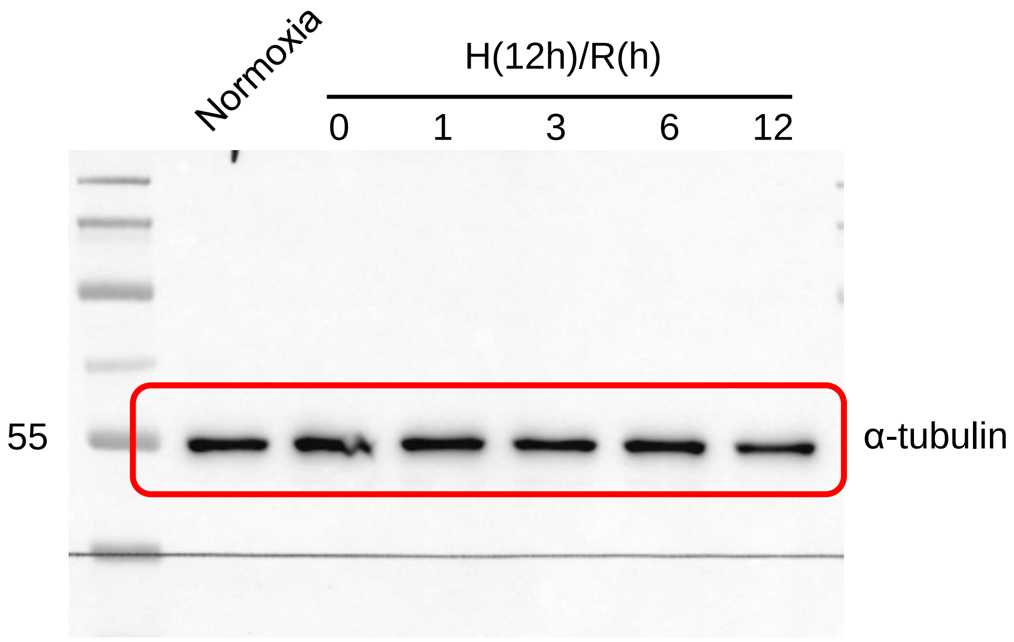
Normoxia
H(12h)/R(h)
0
1
3
6
12
55
α-tubulin

Figure 1n

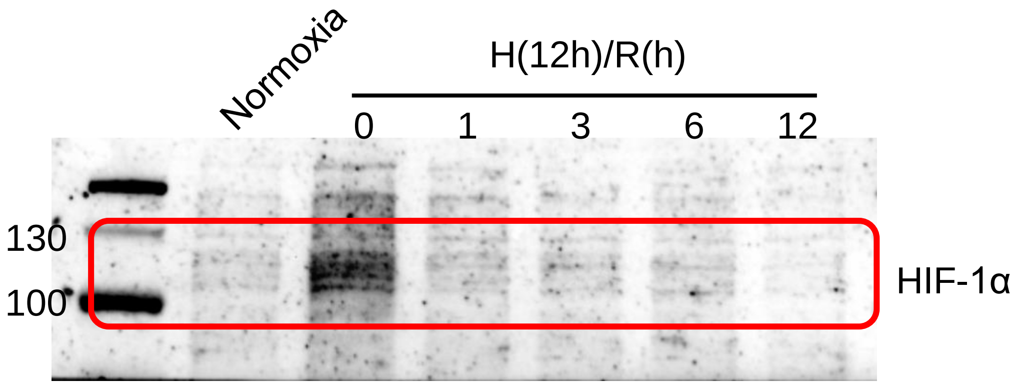

(data not shown)

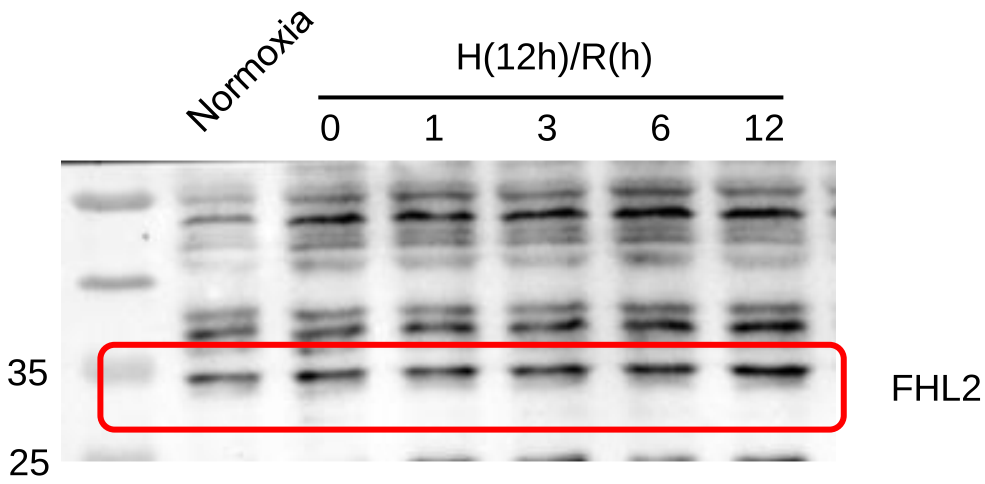

(data not shown)

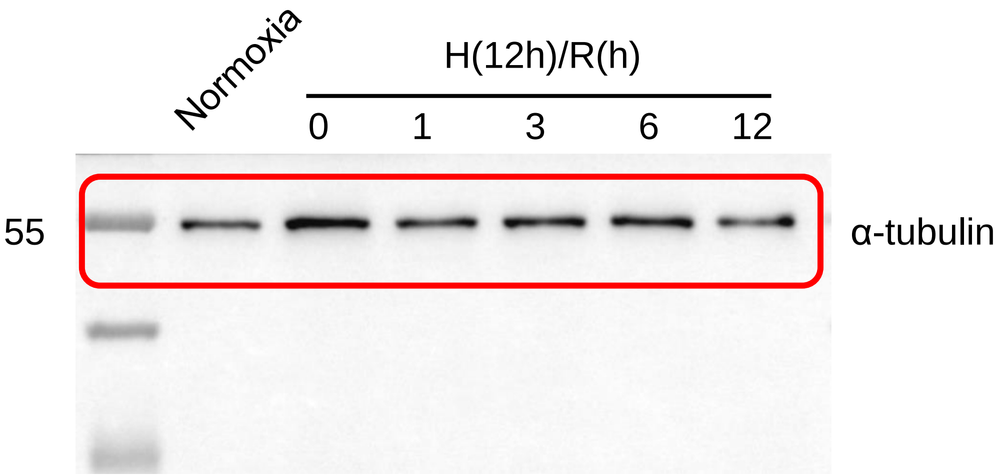

(data not shown)

Figure 1o

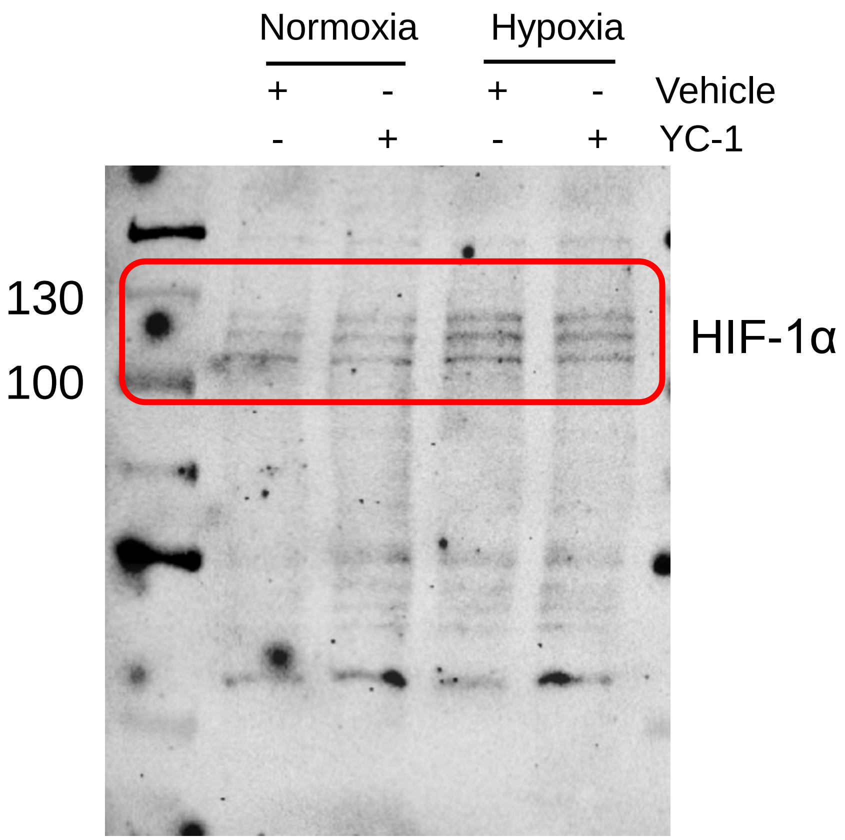

Normoxia
Hypoxia
+ - + - Vehicle
- + - + YC-1
130
100
HIF-1α

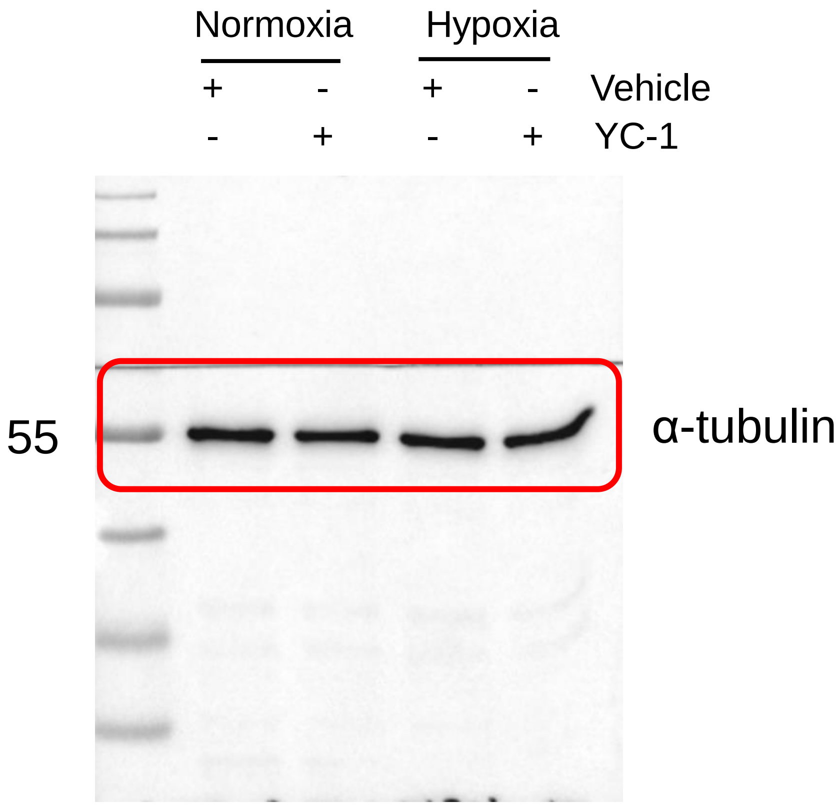

Normoxia
Hypoxia
+ - + - Vehicle
- + - + YC-1
55
α-tubulin

Figure 1o

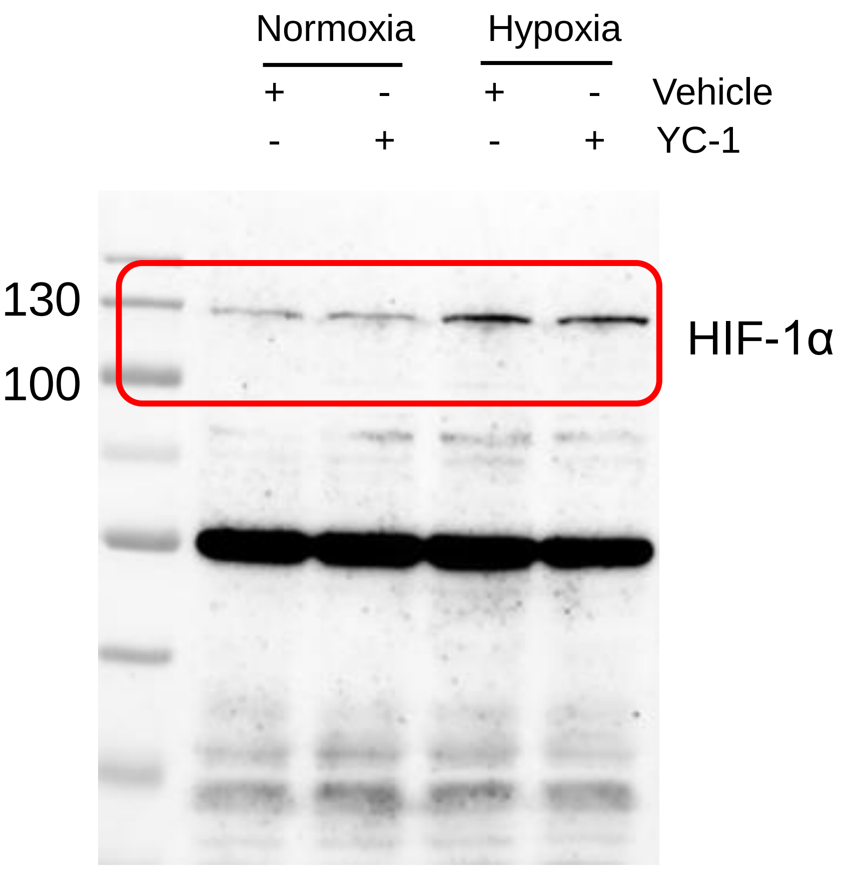

(data not shown)

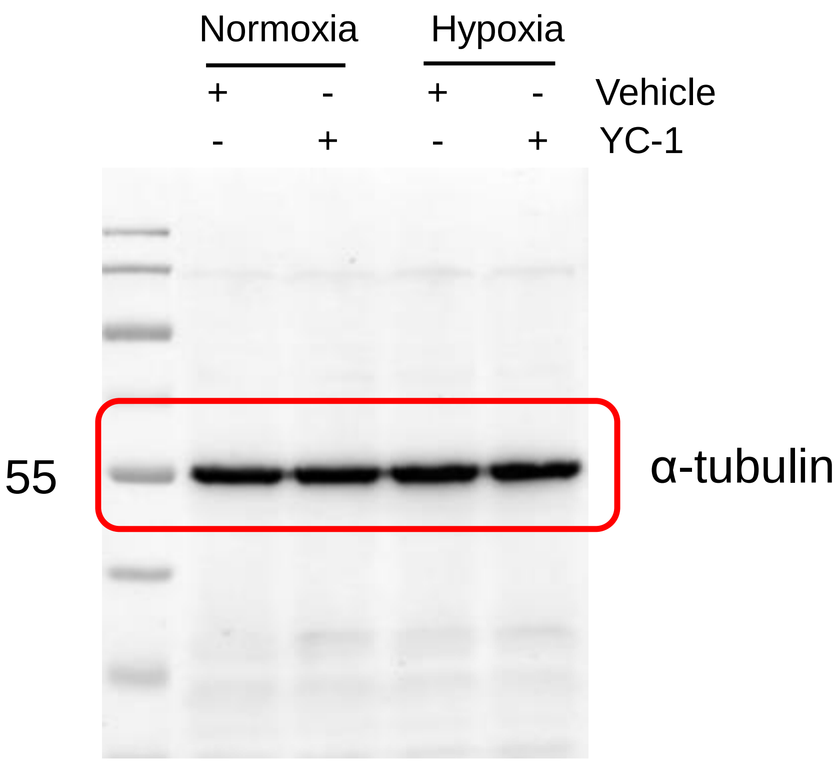

(data not shown)

Figure 1o

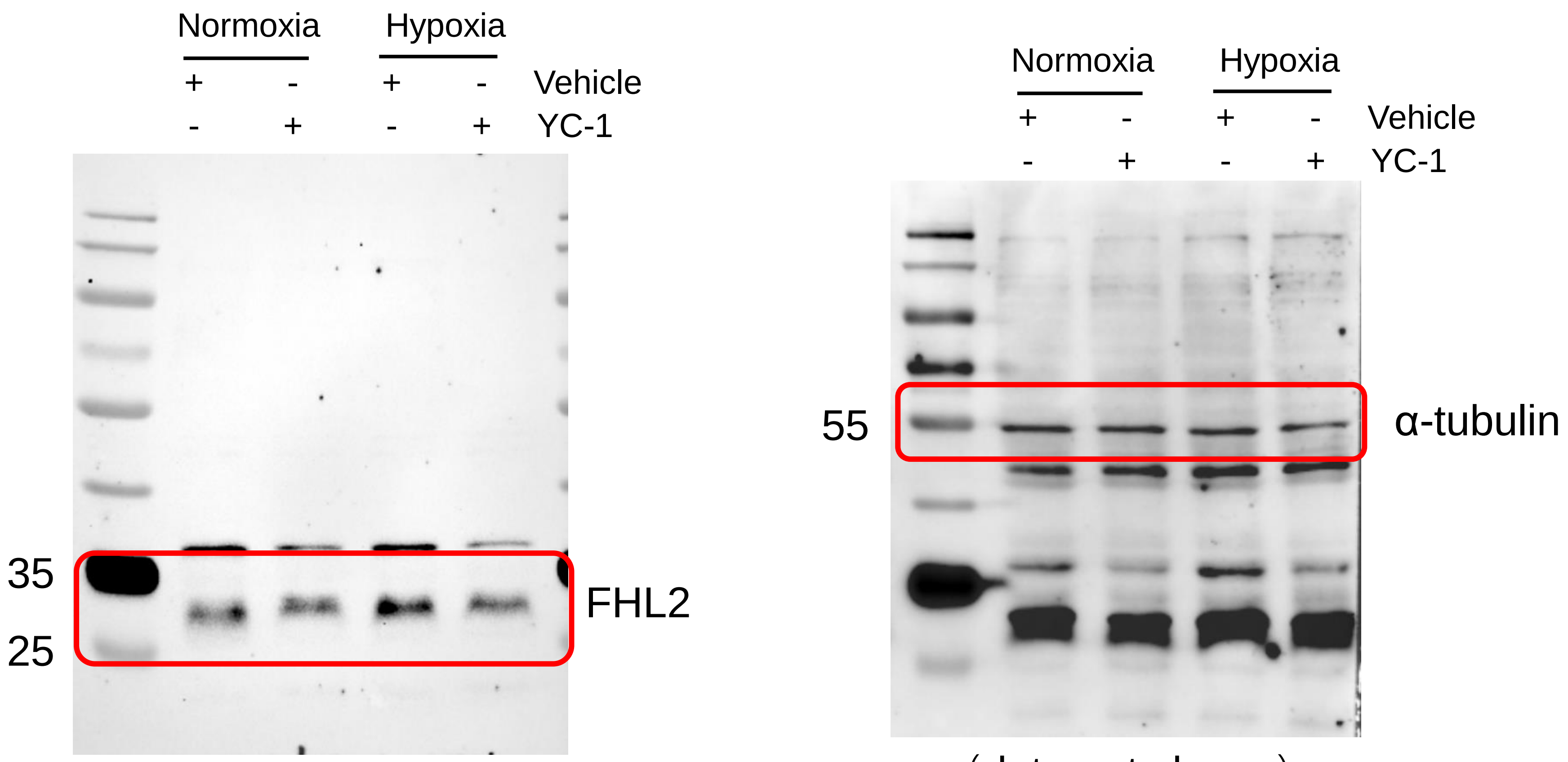

（data not shown）

Figure 1o

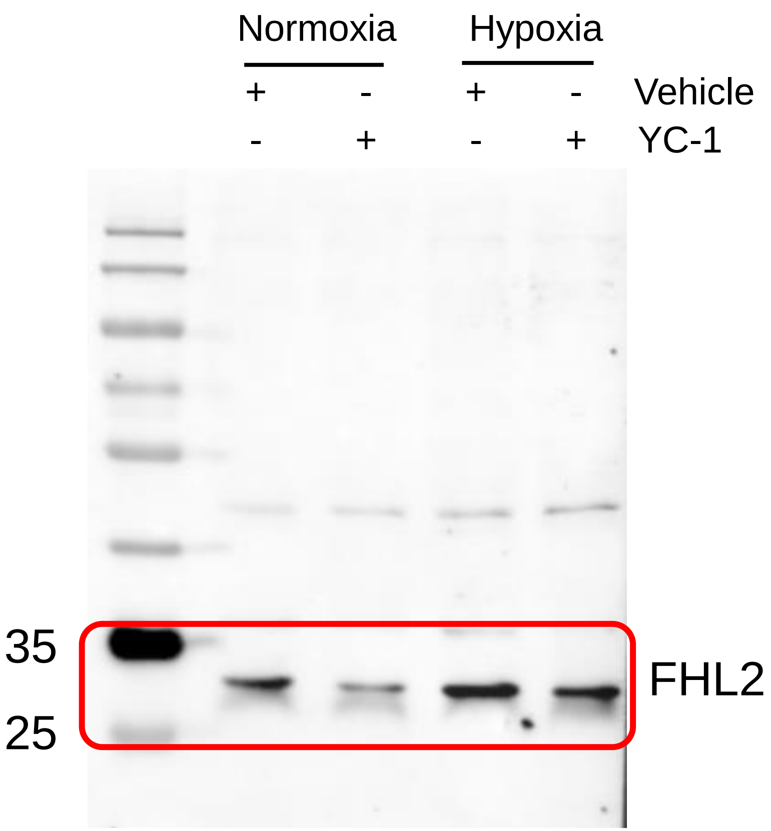

(data not shown)

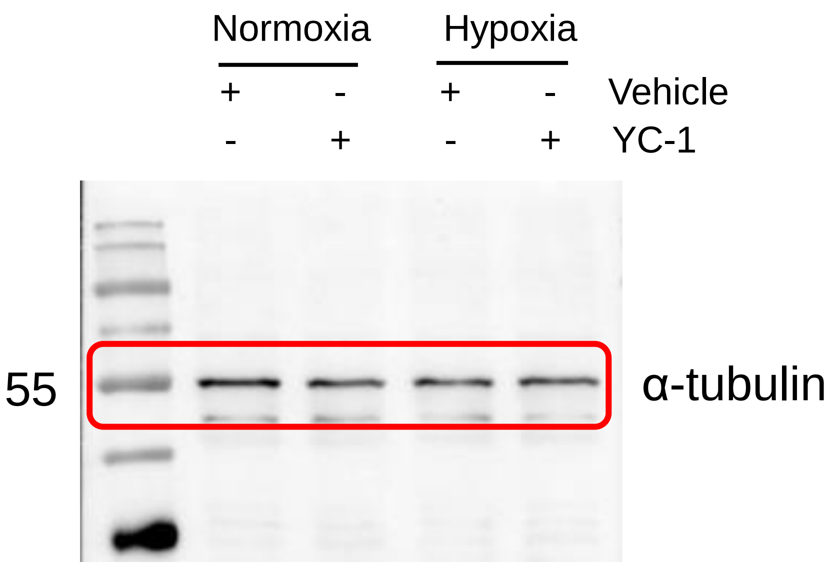

(data not shown)

Figure 2d

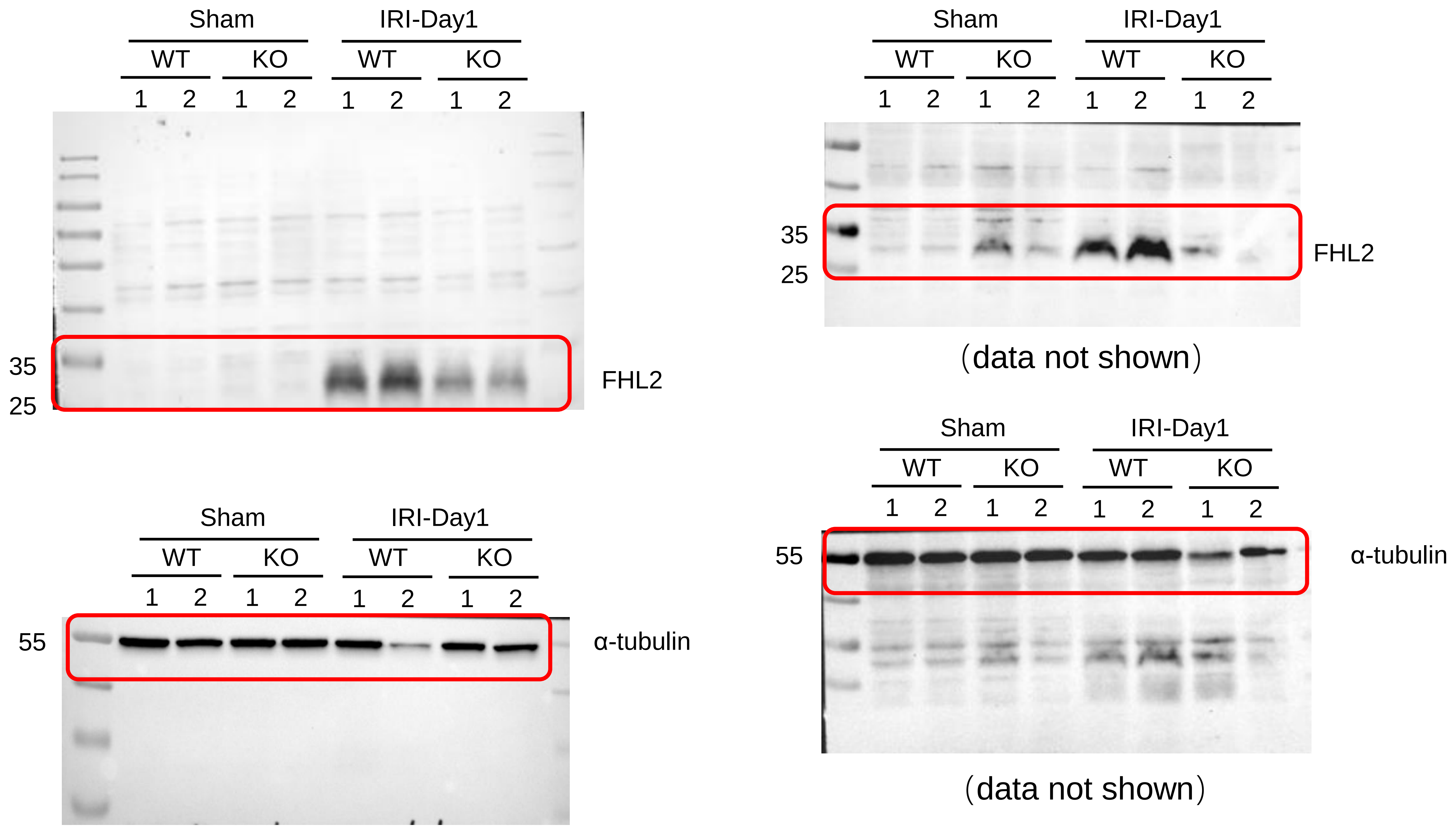
Sham
IRI-Day1
WT
KO
WT
KO
1
2
1
2
1
2
1
2
35
25
FHL2
(data not shown)
55
α-tubulin
(data not shown)

Figure 2d

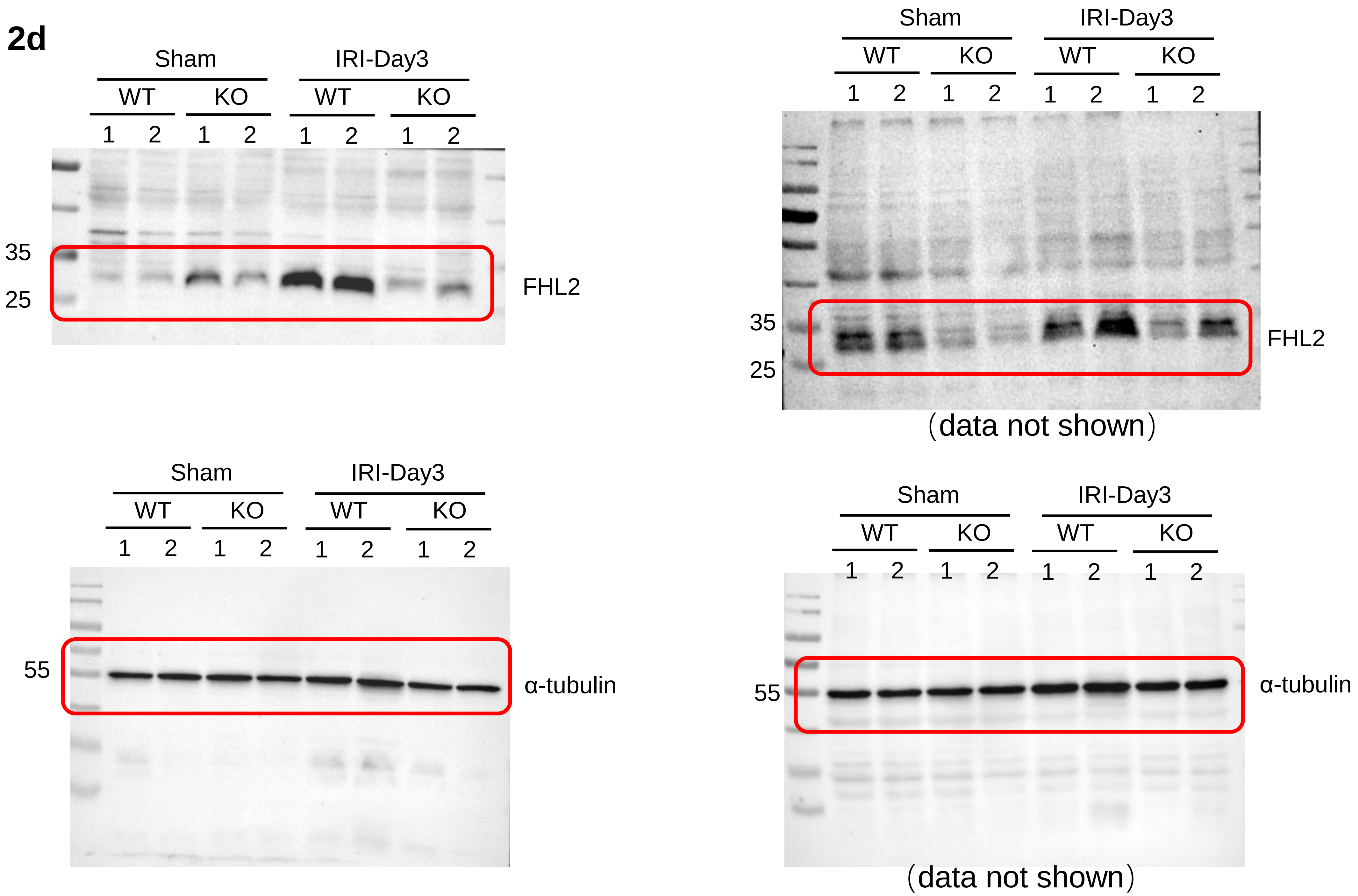

Figure 3d

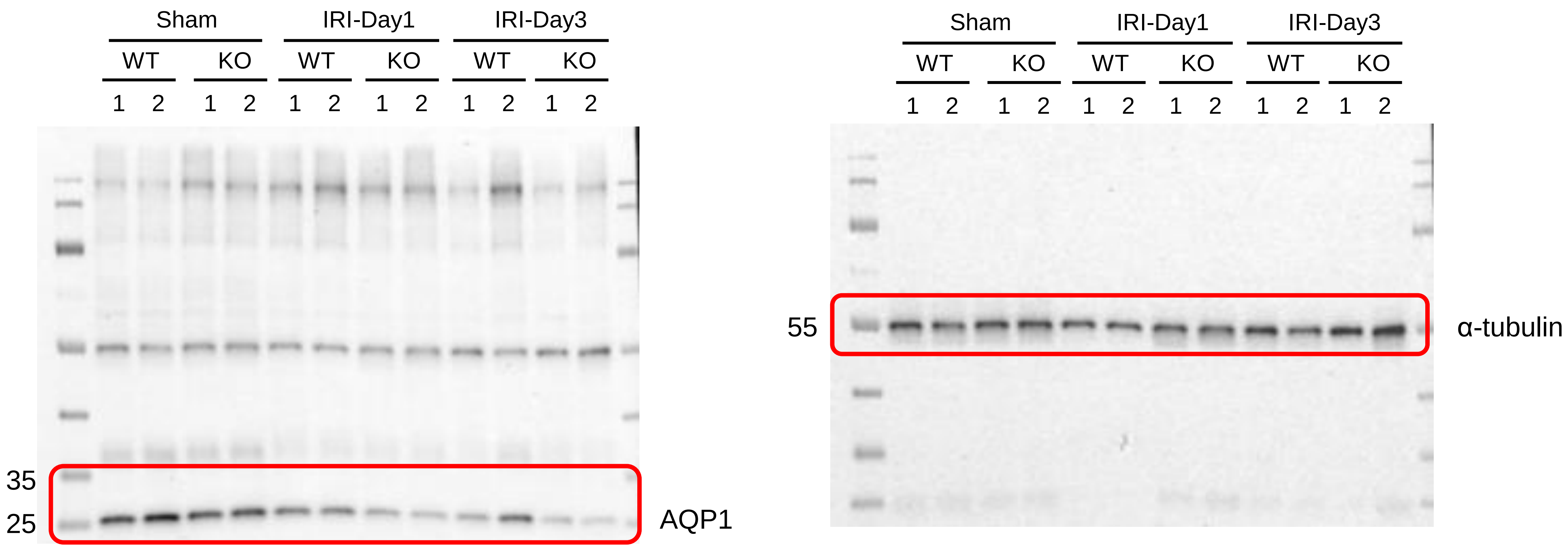
Sham
IRI-Day1
IRI-Day3
WT
KO
WT
KO
WT
KO
1 2 1 2 1 2 1 2 1 2 1 2
35
25
AQP1
Sham
IRI-Day1
IRI-Day3
WT
KO
WT
KO
WT
KO
1 2 1 2 1 2 1 2 1 2 1 2
55
α-tubulin

Figure 3d

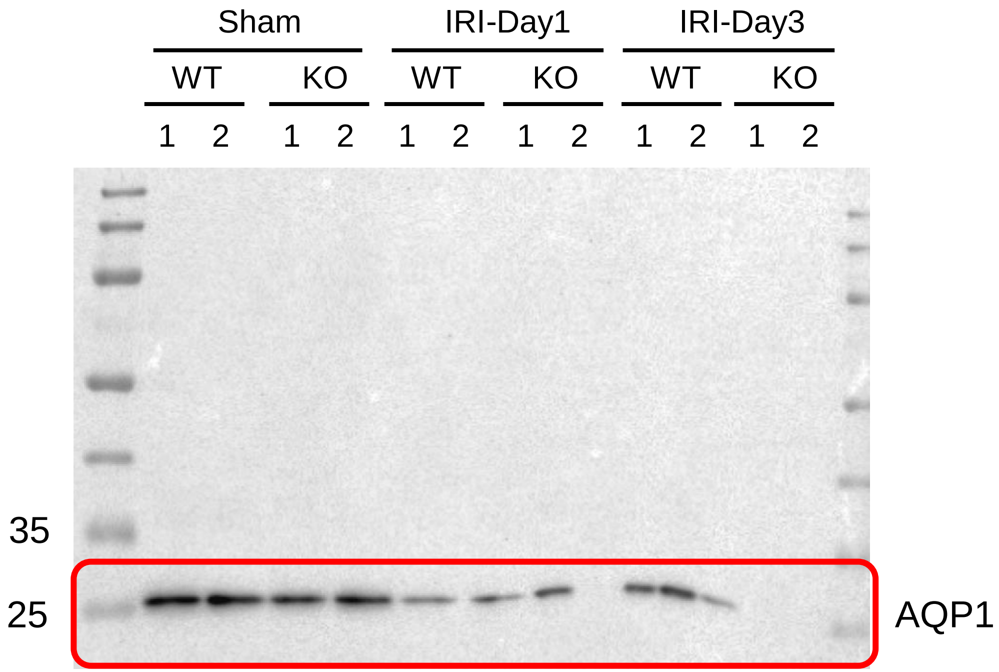

(data not shown)

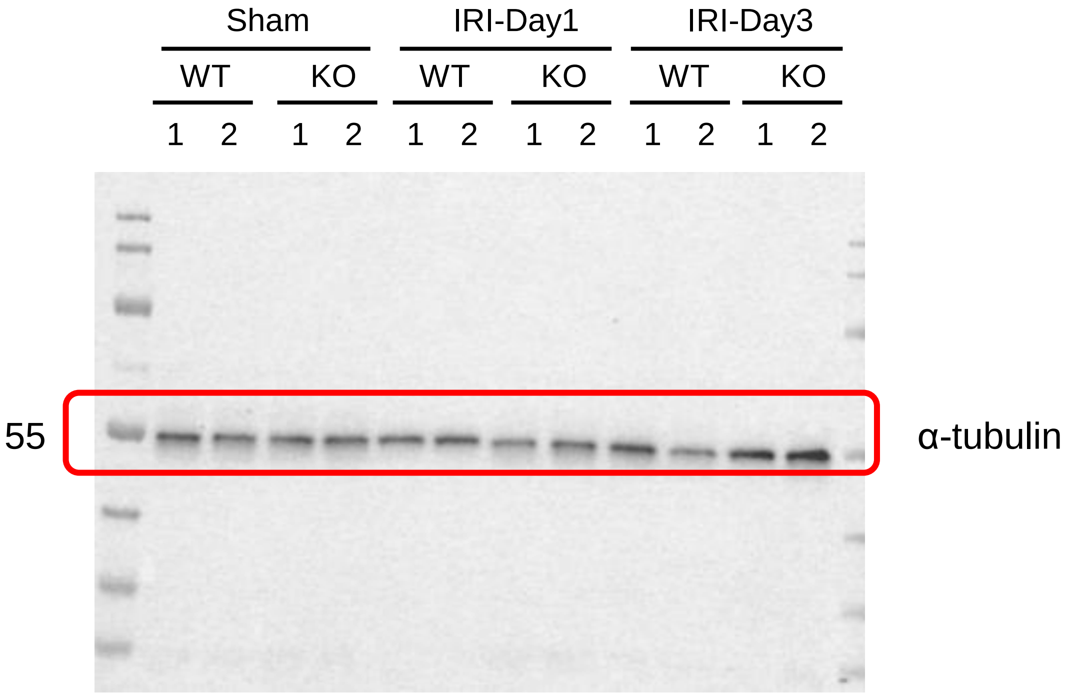

(data not shown)

Figure 3e

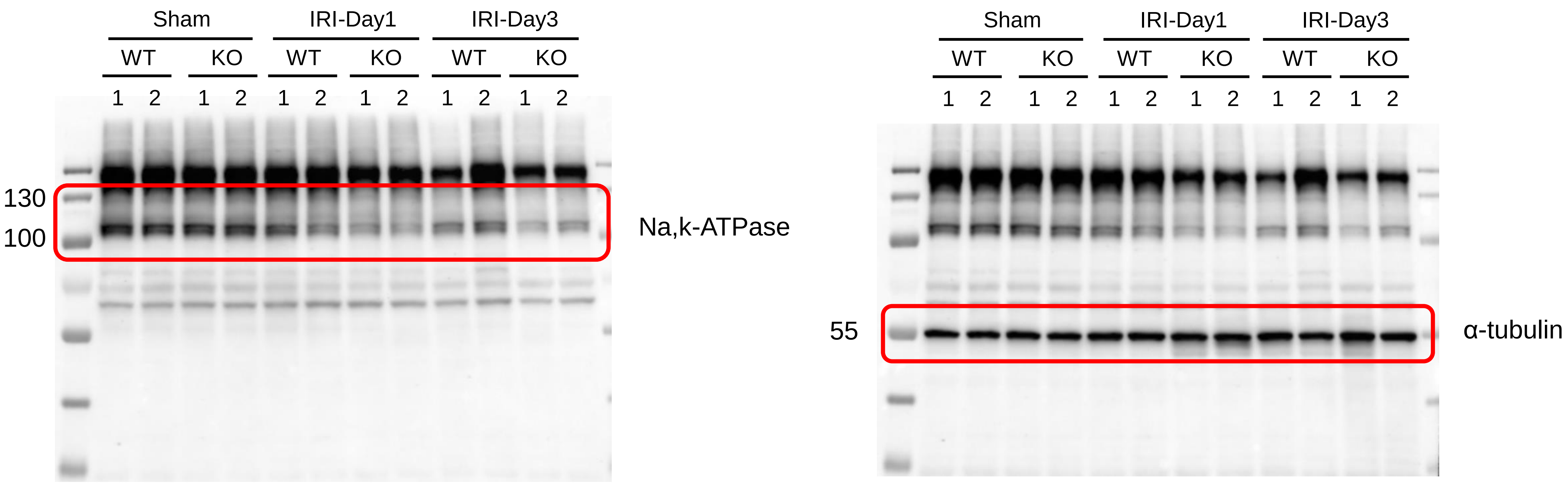

Sham
IRI-Day1
IRI-Day3
WT KO WT KO WT KO
1 2 1 2 1 2 1 2 1 2 1 2
130
100
Na,k-ATPase
Sham
IRI-Day1
IRI-Day3
WT KO WT KO WT KO
1 2 1 2 1 2 1 2 1 2 1 2
55
α-tubulin

Figure 3e

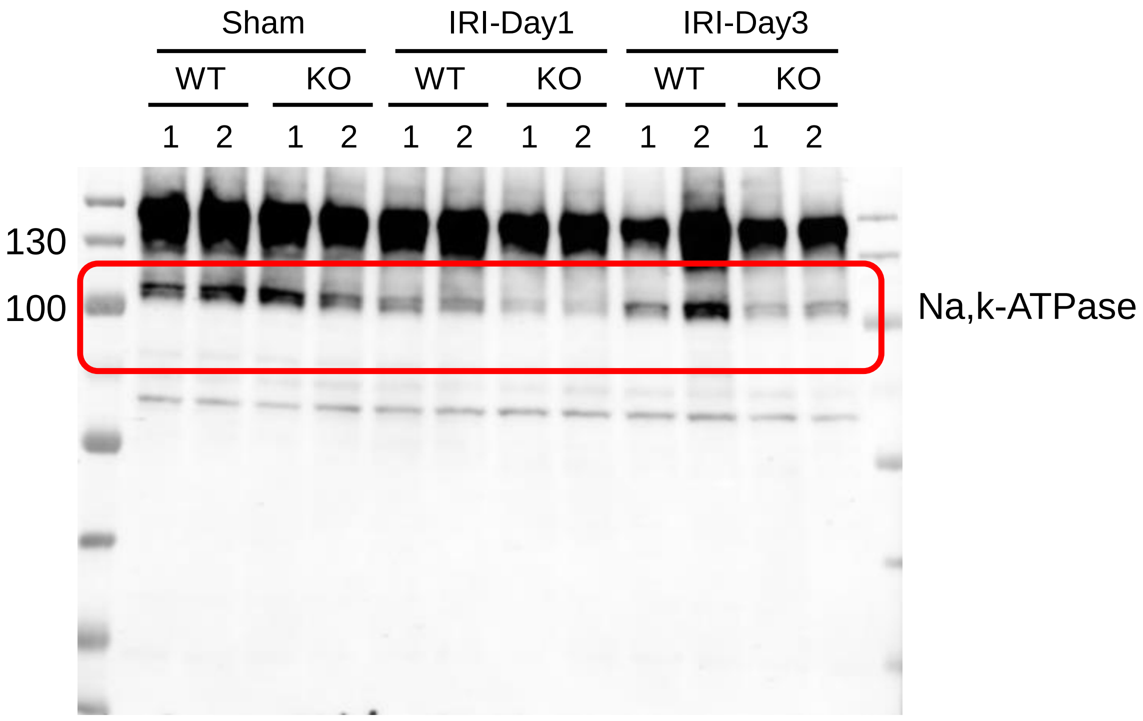

(data not shown)

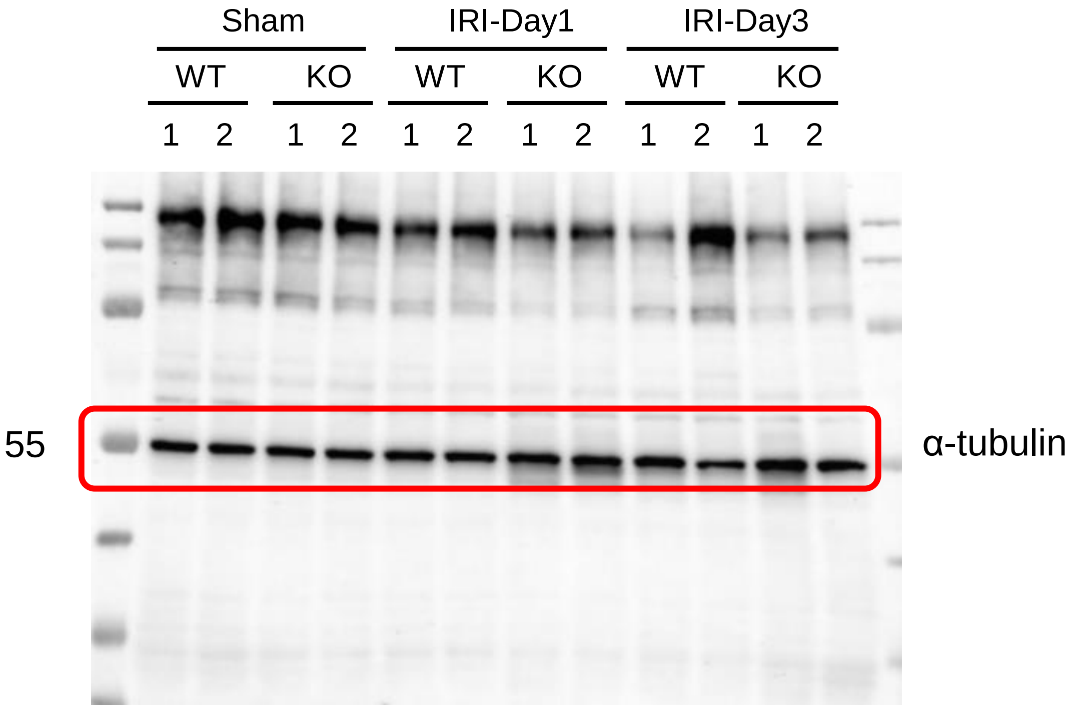

(data not shown)

Figure 4a

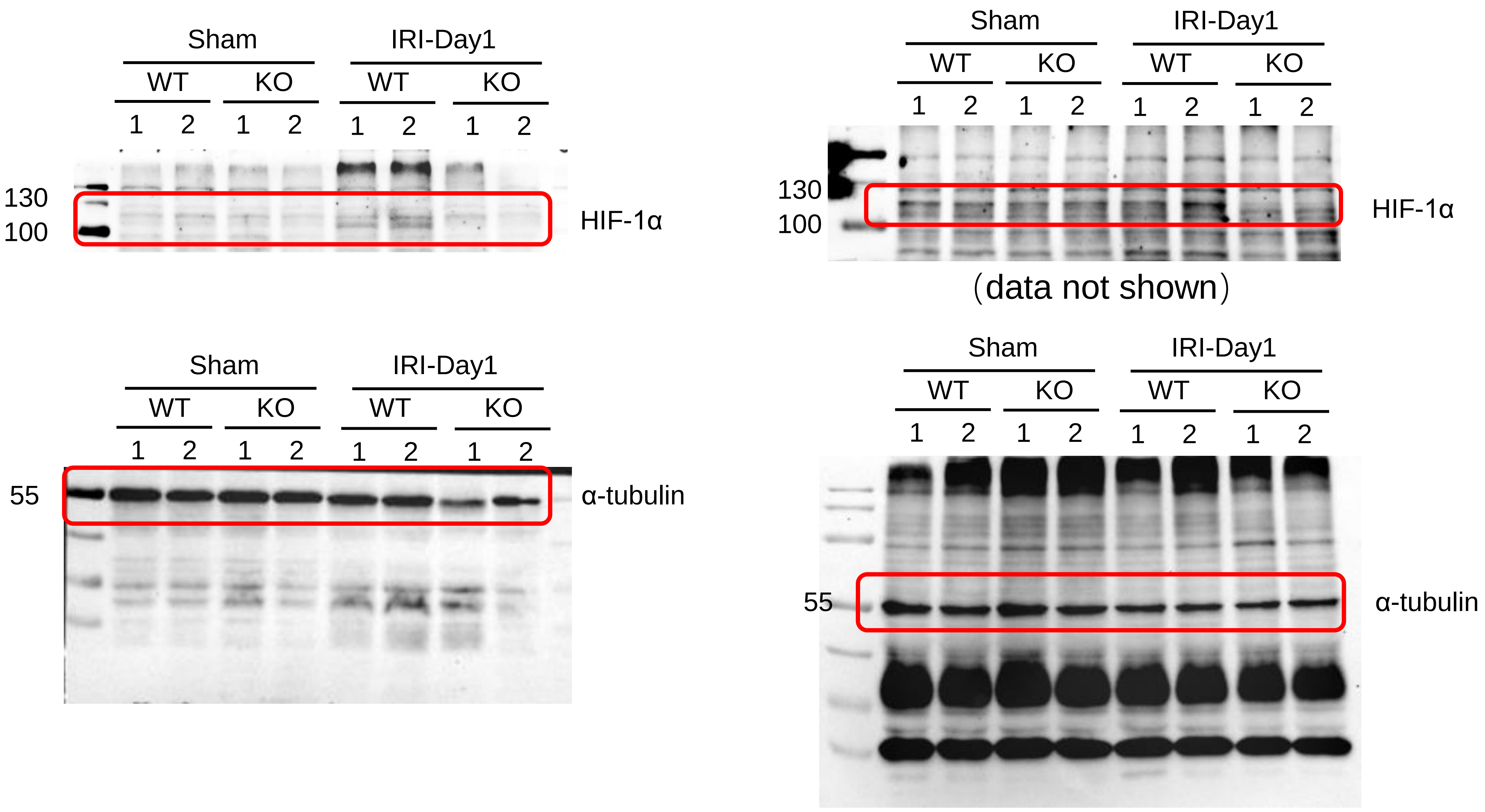

(data not shown)

Figure 4a

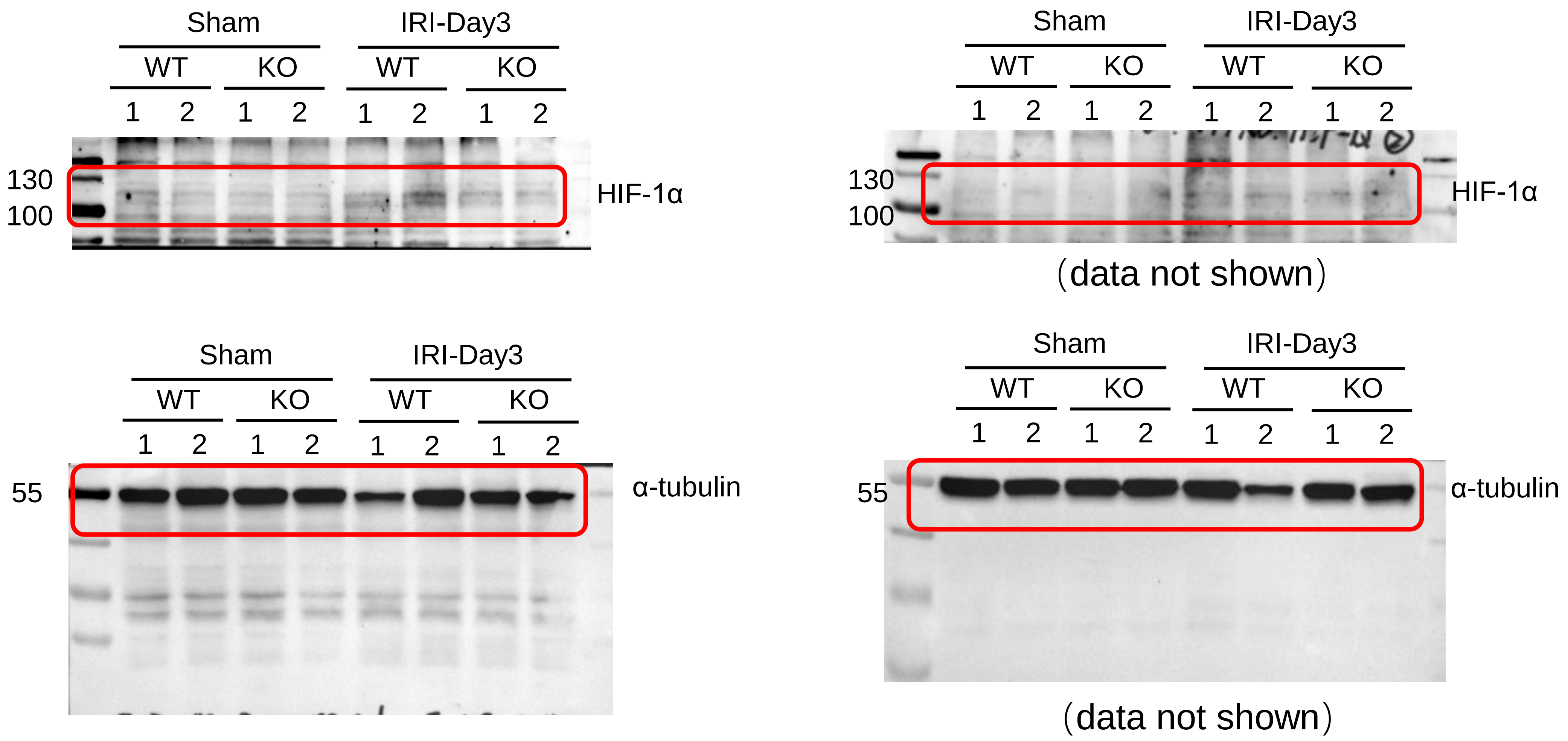

Sham
IRI-Day3
WT
KO
1
2
130
100
HIF-1α
(data not shown)
55
α-tubulin

Figure 4e

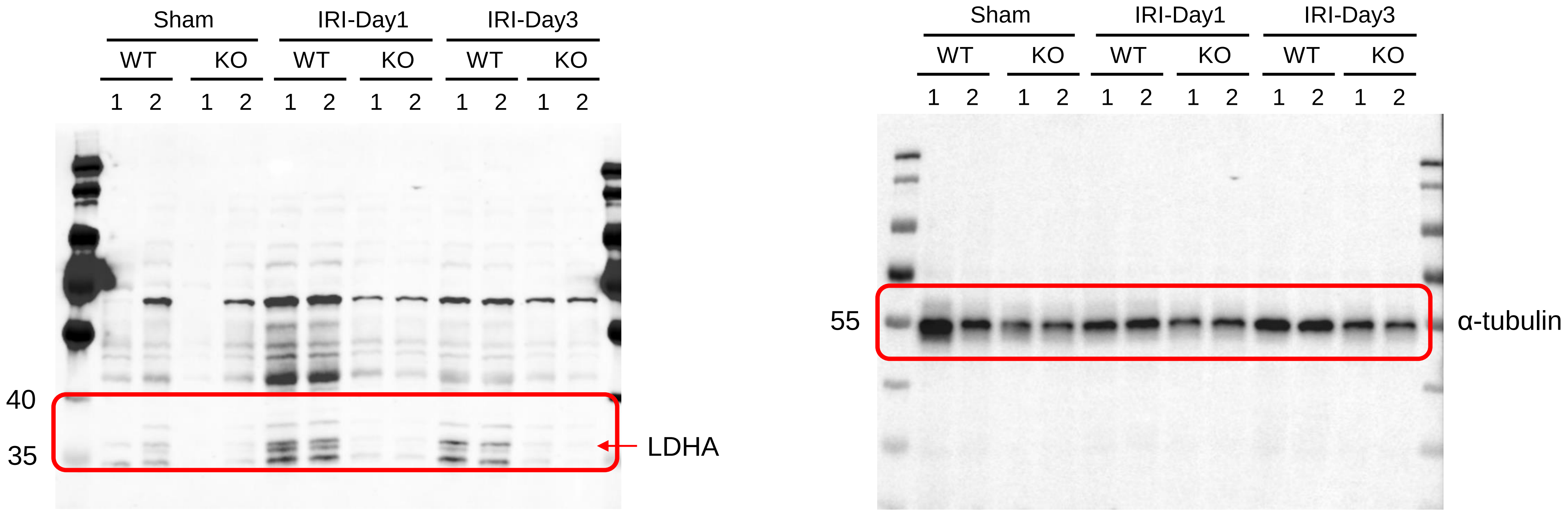

Sham
IRI-Day1
IRI-Day3
WT KO WT KO WT KO
1 2 1 2 1 2 1 2 1 2 1 2
40
35
LDHA
55
α-tubulin

Figure 4e

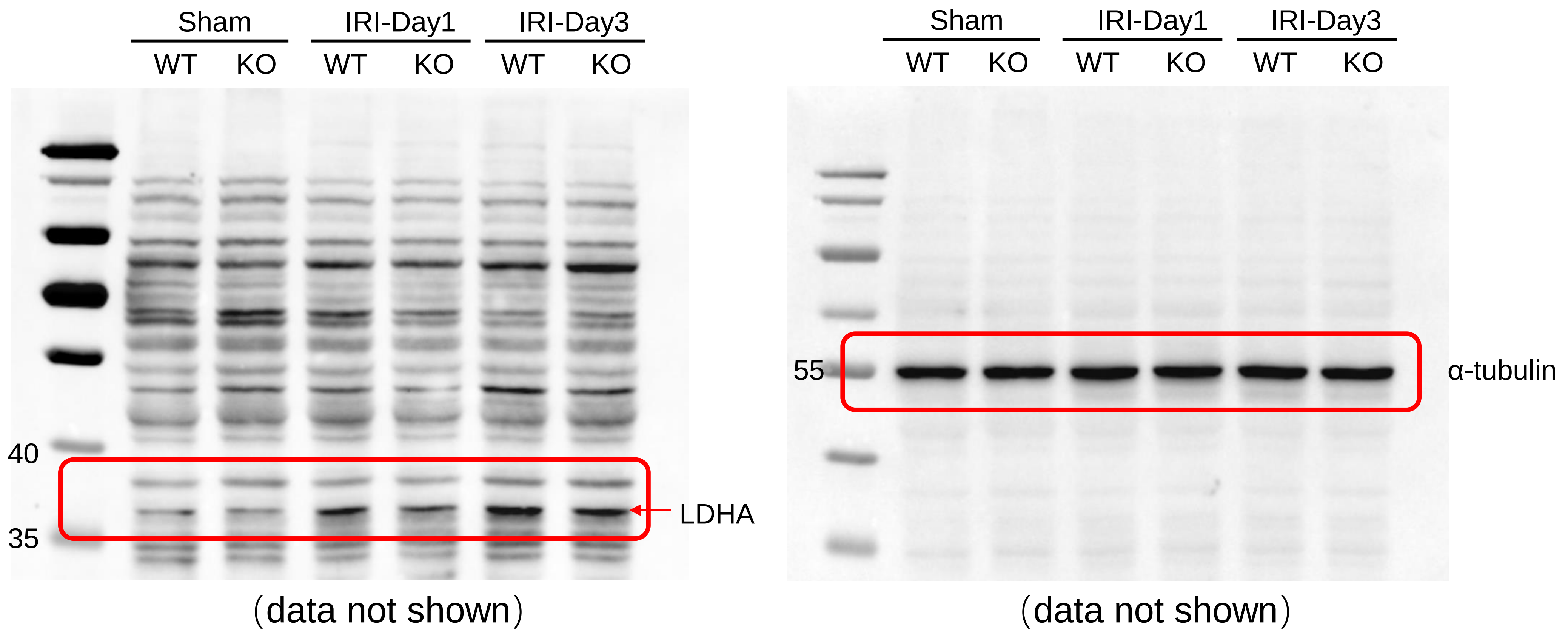
Sham
IRI-Day1
IRI-Day3
WT
KO
WT
KO
WT
KO
40
35
LDHA
Sham
IRI-Day1
IRI-Day3
WT
KO
WT
KO
WT
KO
55
α-tubulin
(data not shown)
(data not shown)

Figure 4h

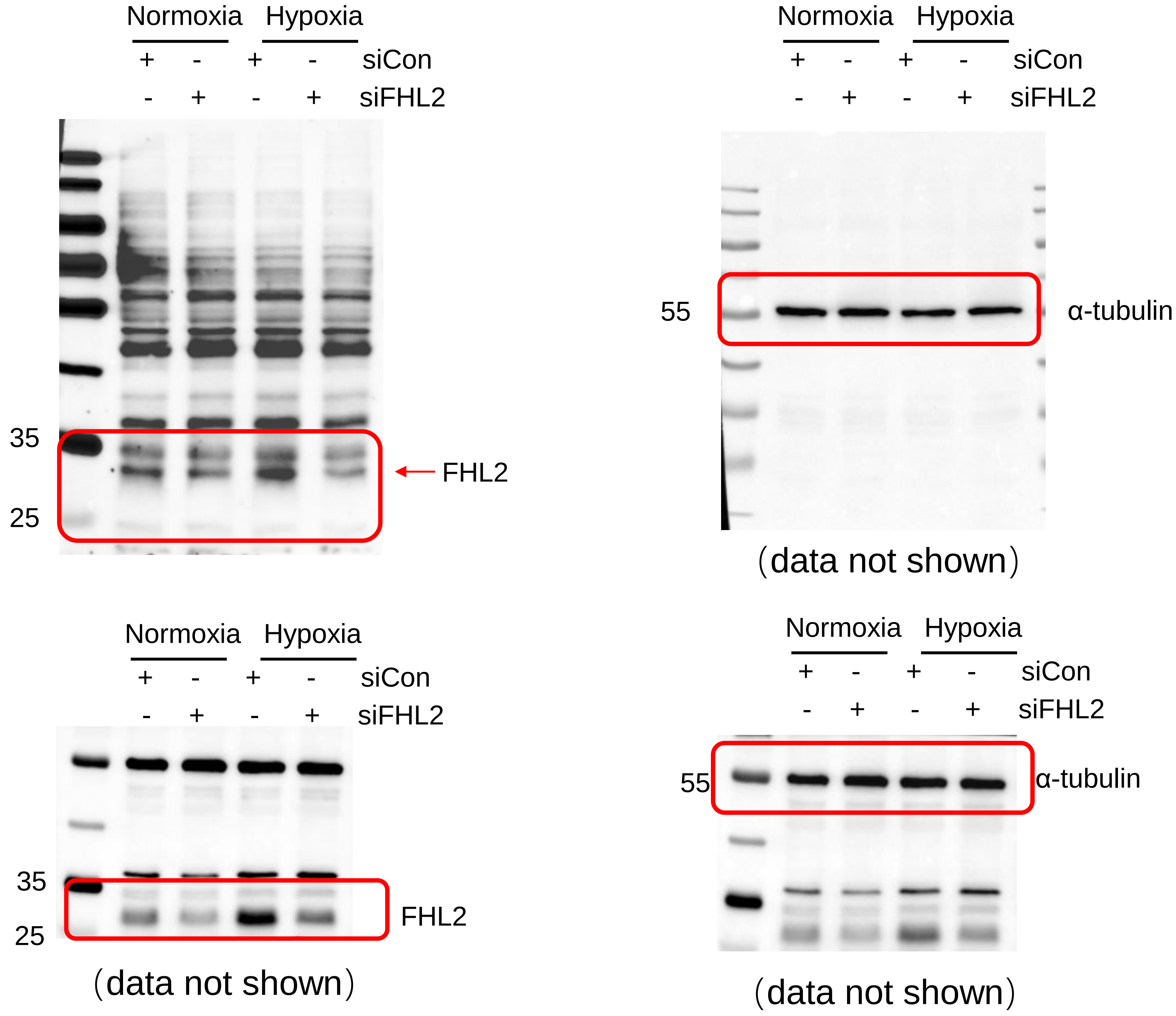

Normoxia
Hypoxia
+ - + - siCon
- + - + siFHL2
35
25
FHL2
55
α-tubulin
(data not shown)
Normoxia
Hypoxia
+ - + - siCon
- + - + siFHL2
35
25
FHL2
(data not shown)
55
α-tubulin
(data not shown)

Figure 4h

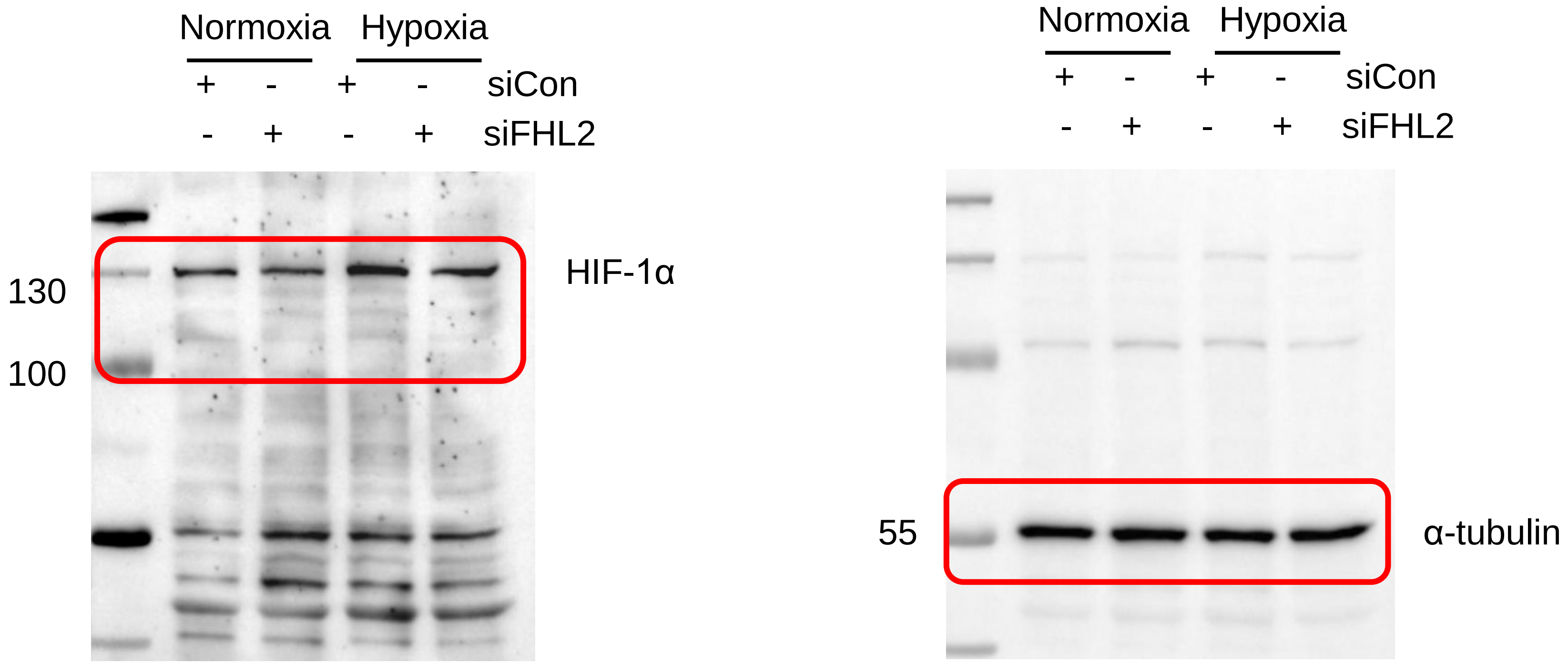
Normoxia
Hypoxia
+ - + - siCon
- + - + siFHL2
130
100
HIF-1α
Normoxia
Hypoxia
+ - + - siCon
- + - + siFHL2
55
α-tubulin

Figure 4h

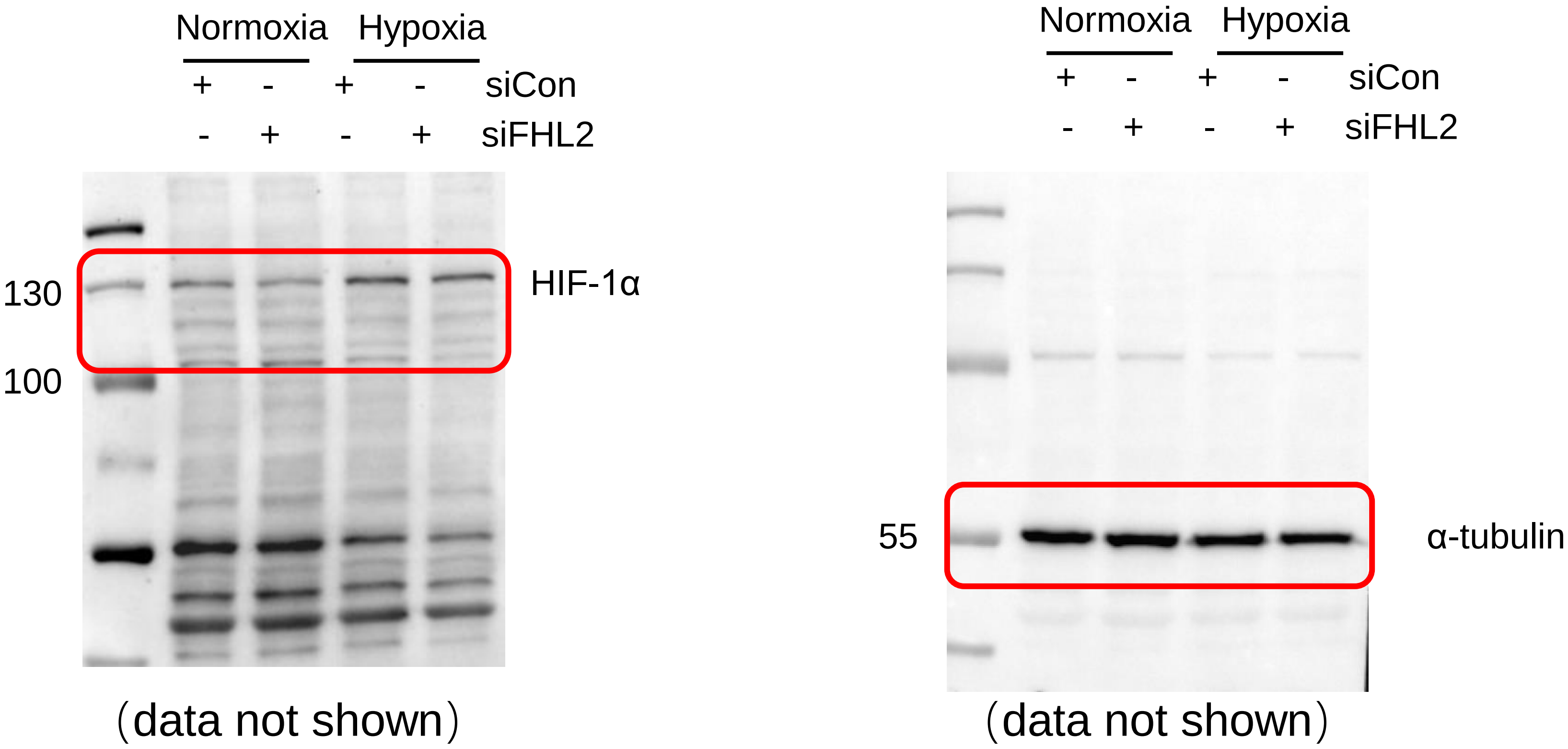

Normoxia
Hypoxia
+ - + - siCon
- + - + siFHL2
130
100
HIF-1α
(data not shown)
Normoxia
Hypoxia
+ - + - siCon
- + - + siFHL2
55
α-tubulin
(data not shown)

Figure 4k

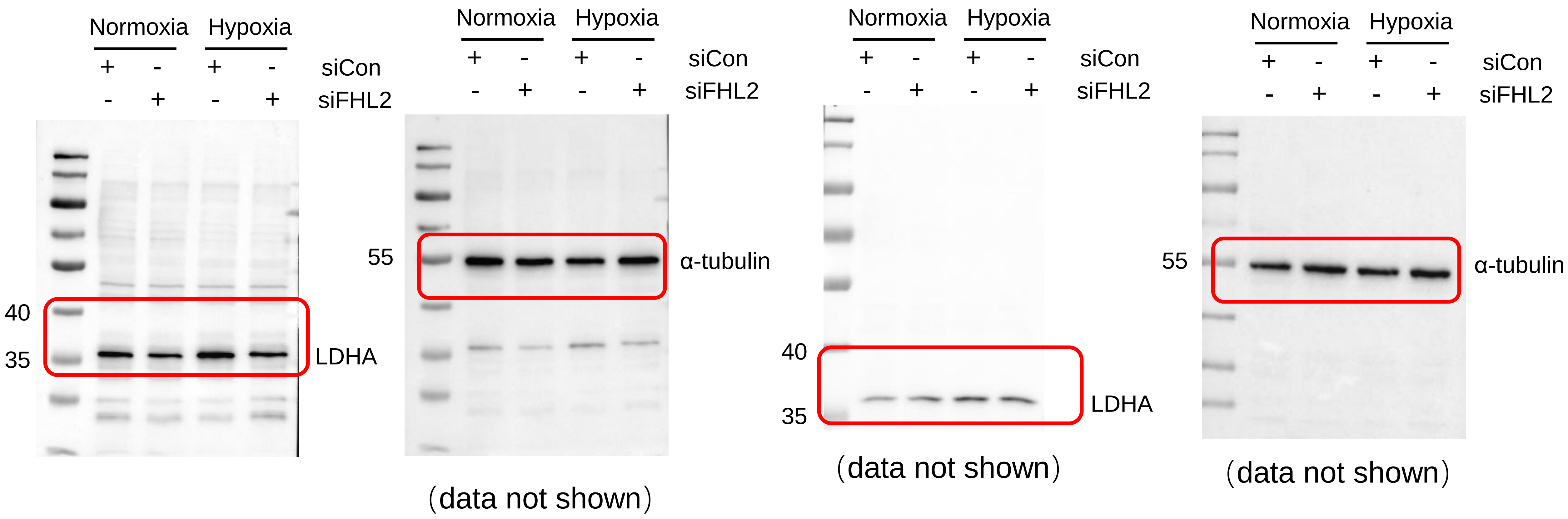

Normoxia
Hypoxia
siCon
siFHL2
+ - + -
- + - +
40
35
LDHA
55
α-tubulin
(data not shown)

Figure 4k

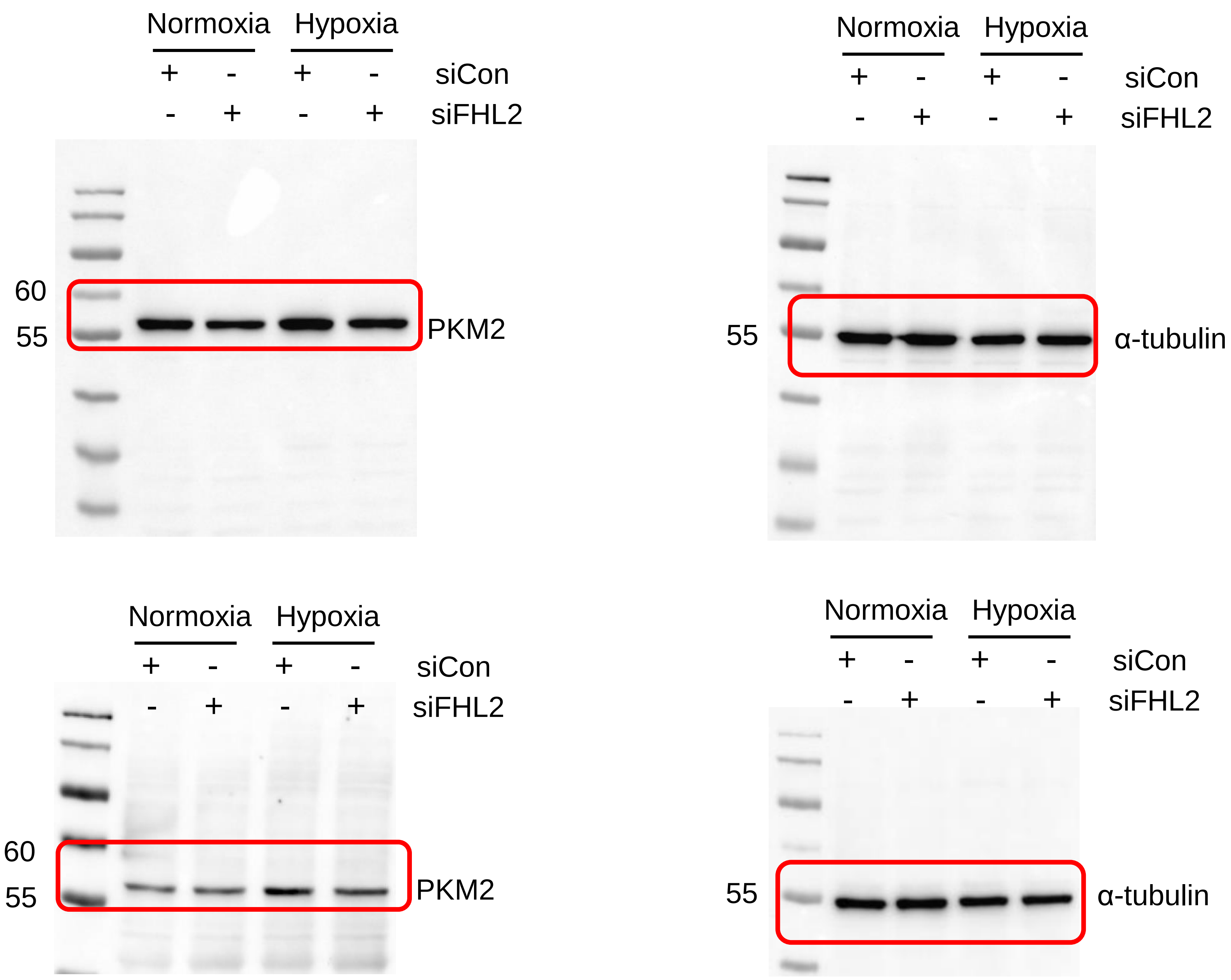

(data not shown)

(data not shown)

Figure 4k

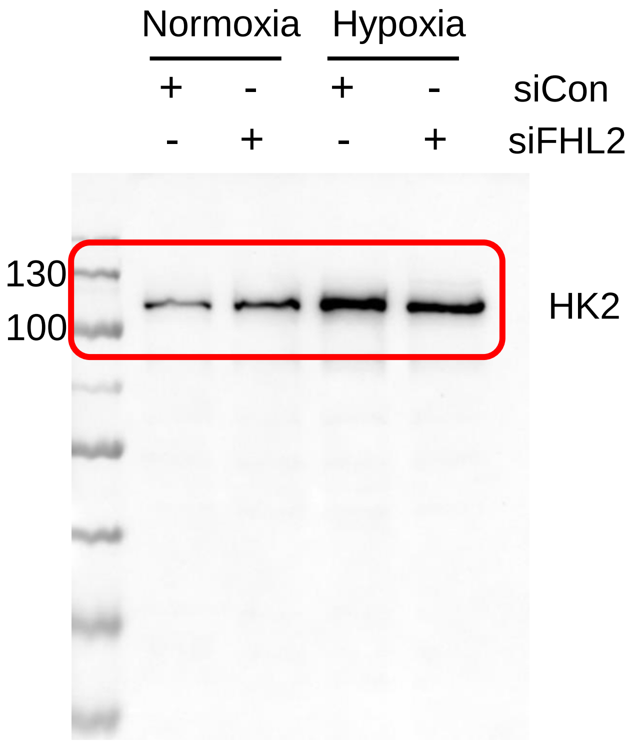

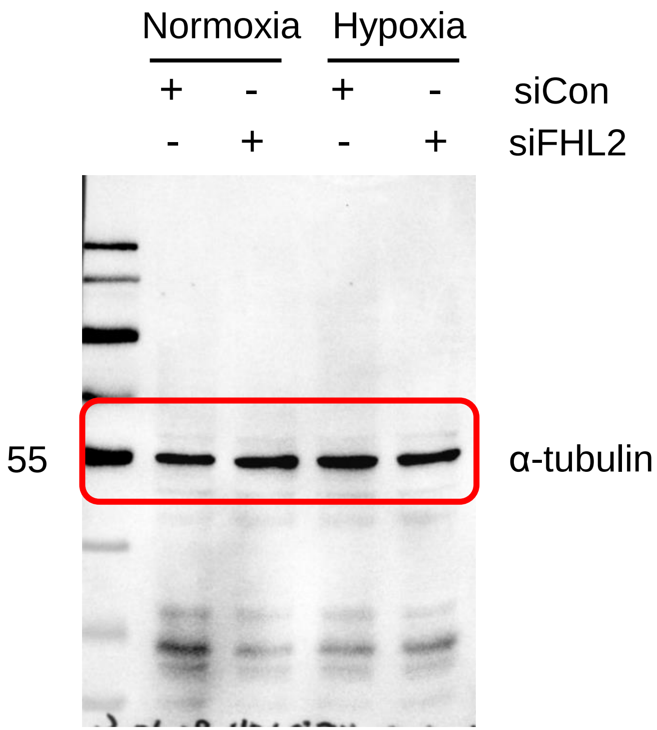

(data not shown)

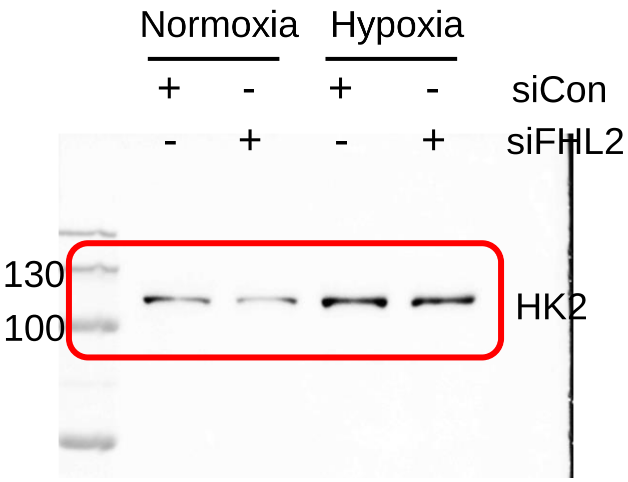

(data not shown)

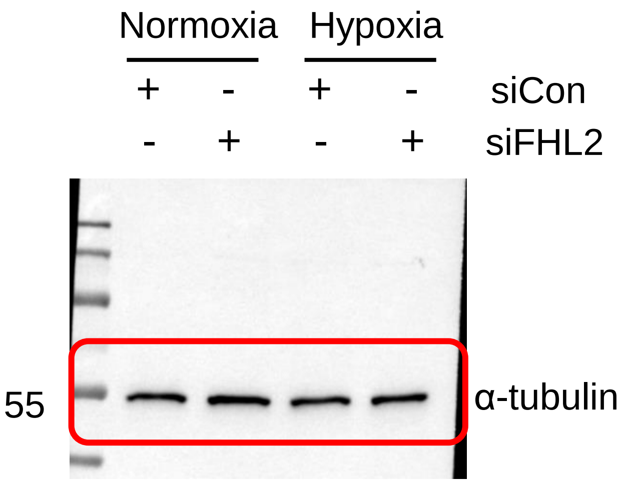

(data not shown)

Figure 5a

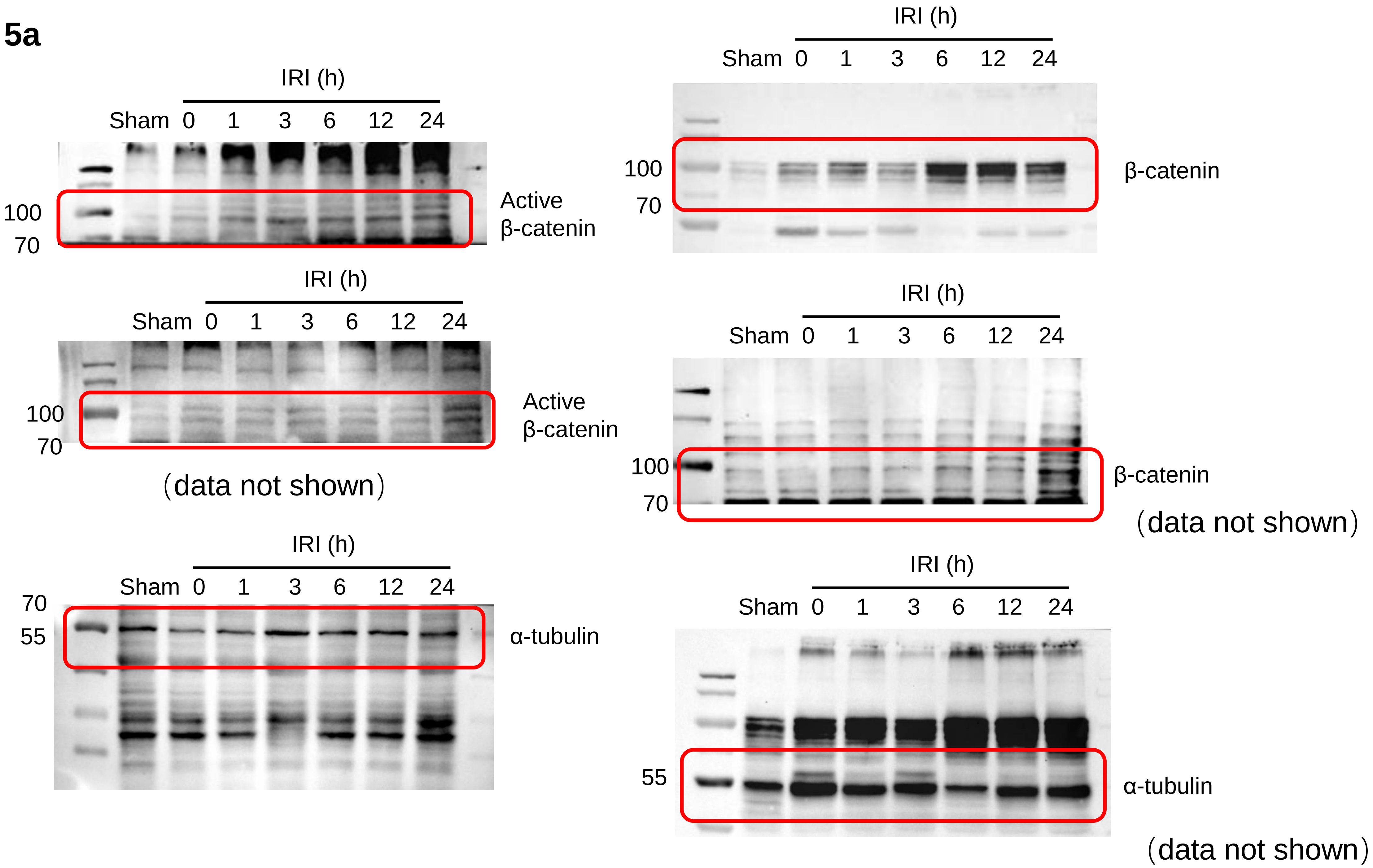
IRI (h)
Sham 0 1 3 6 12 24
100
70
Active β-catenin
IRI (h)
Sham 0 1 3 6 12 24
100
70
β-catenin
IRI (h)
Sham 0 1 3 6 12 24
100
70
Active β-catenin
(data not shown)
IRI (h)
Sham 0 1 3 6 12 24
100
70
β-catenin
(data not shown)
IRI (h)
Sham 0 1 3 6 12 24
70
55
α-tubulin
IRI (h)
Sham 0 1 3 6 12 24
55
α-tubulin
(data not shown)

Figure 5b

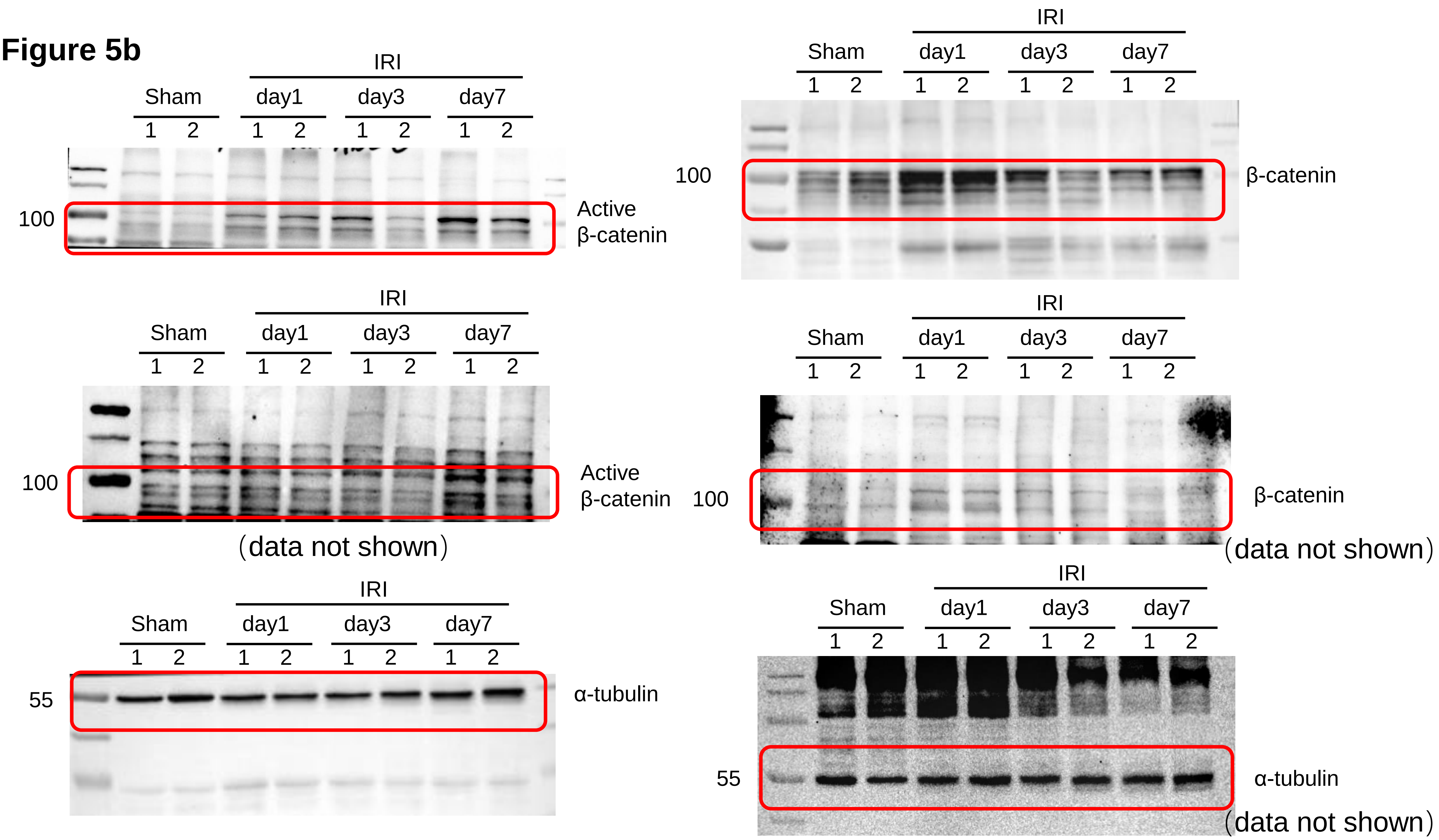
IRI
Sham
day1
day3
day7
1 2 1 2 1 2 1 2
100
Active β-catenin
IRI
Sham
day1
day3
day7
1 2 1 2 1 2 1 2
100
β-catenin
IRI
Sham
day1
day3
day7
1 2 1 2 1 2 1 2
100
Active β-catenin
(data not shown)
IRI
Sham
day1
day3
day7
1 2 1 2 1 2 1 2
100
β-catenin
(data not shown)
IRI
Sham
day1
day3
day7
1 2 1 2 1 2 1 2
55
α-tubulin
IRI
Sham
day1
day3
day7
1 2 1 2 1 2 1 2
55
α-tubulin
(data not shown)

Figure 5c

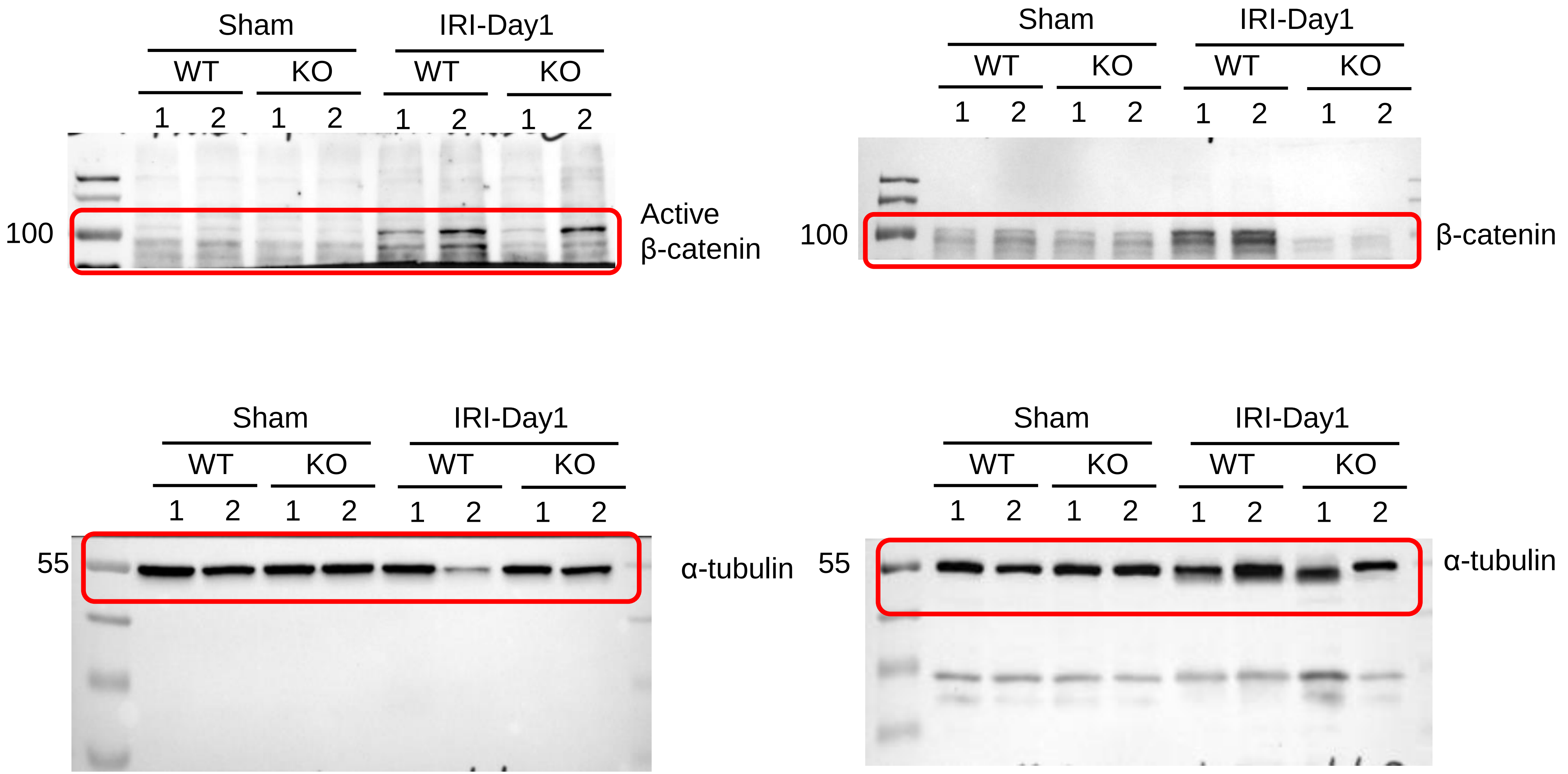

(data not shown)

Figure 5c

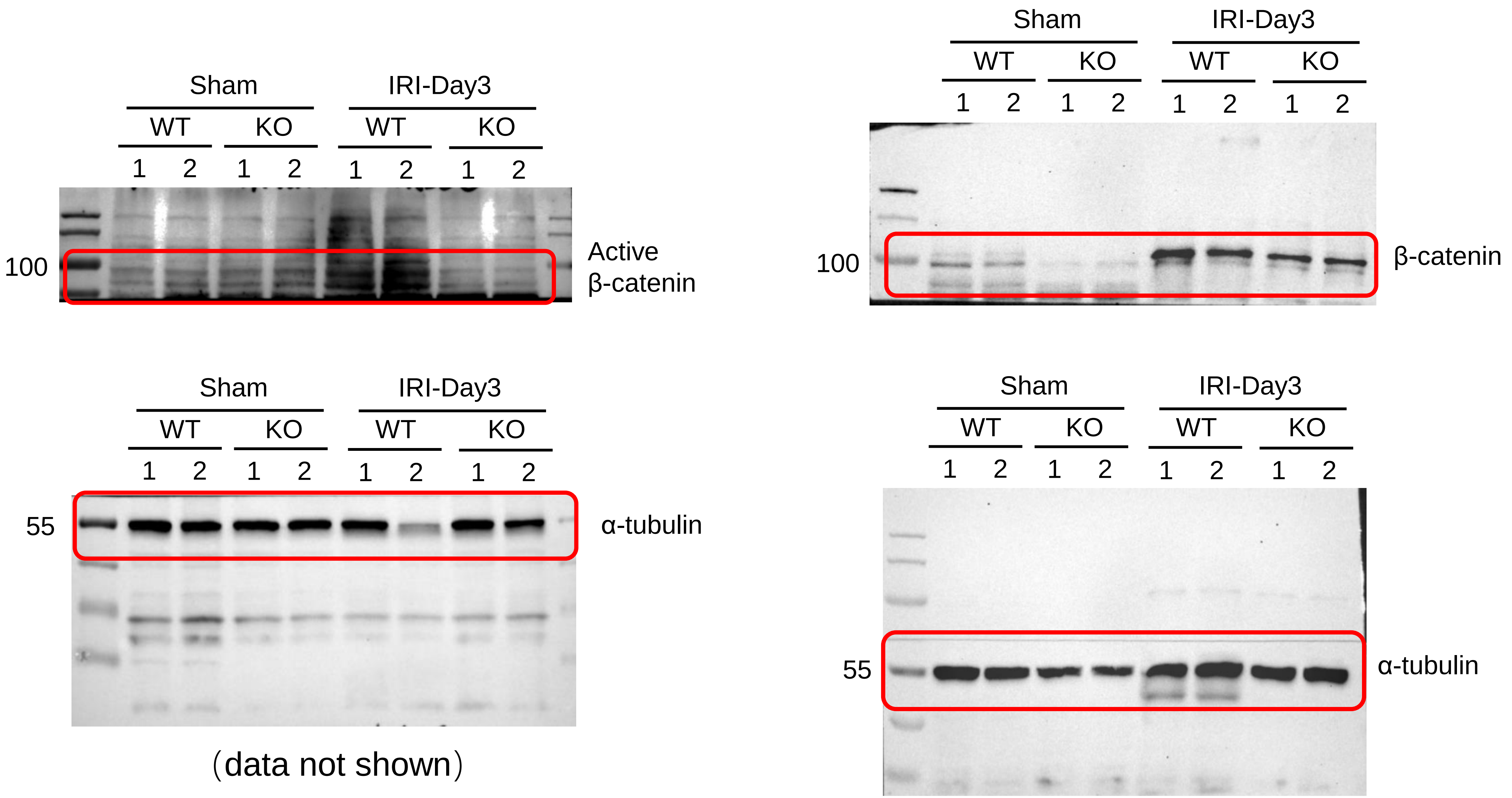
Sham
IRI-Day3
WT
KO
WT
KO
1 2 1 2 1 2 1 2
100
Active β-catenin
Sham
IRI-Day3
WT
KO
WT
KO
1 2 1 2 1 2 1 2
100
β-catenin
Sham
IRI-Day3
WT
KO
WT
KO
1 2 1 2 1 2 1 2
55
α-tubulin
(data not shown)
Sham
IRI-Day3
WT
KO
WT
KO
1 2 1 2 1 2 1 2
55
α-tubulin

Figure 5c

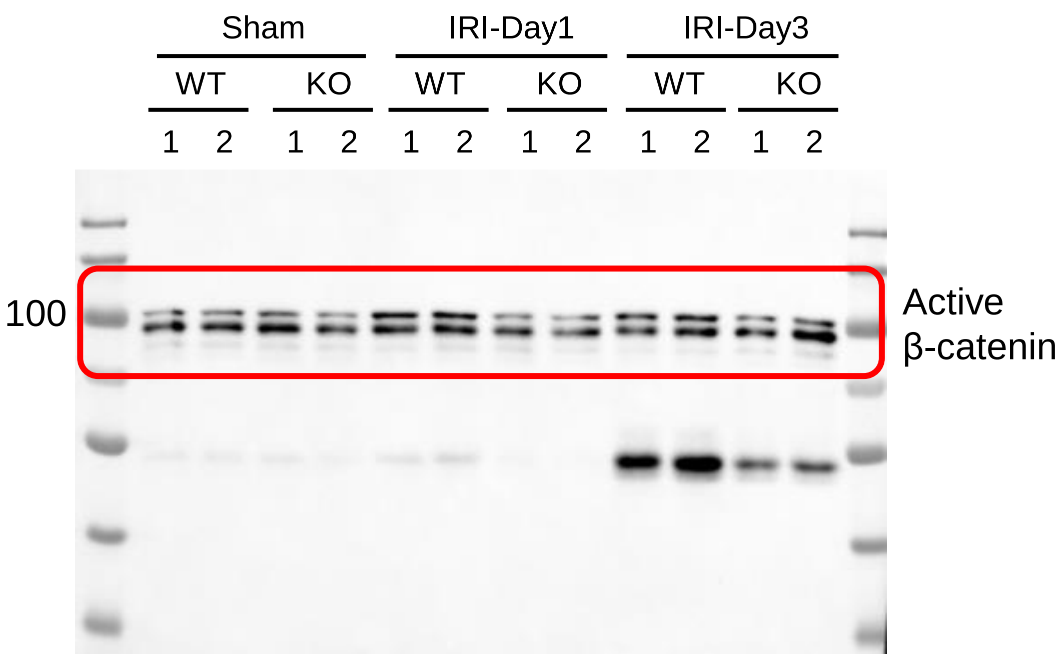

(data not shown)

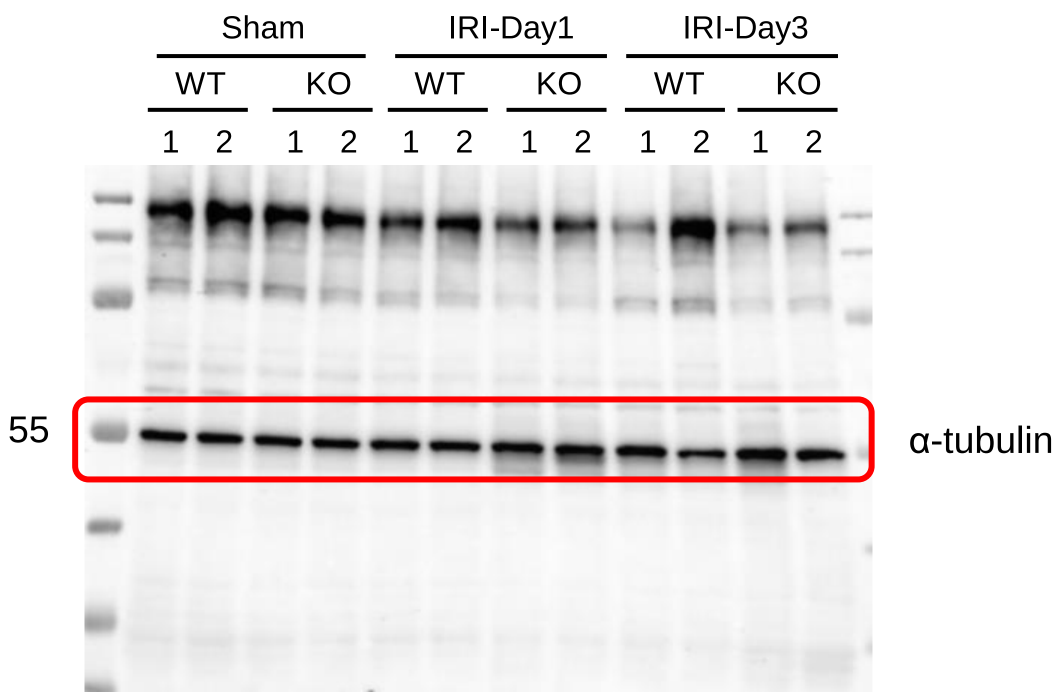

(data not shown)

Figure 5c

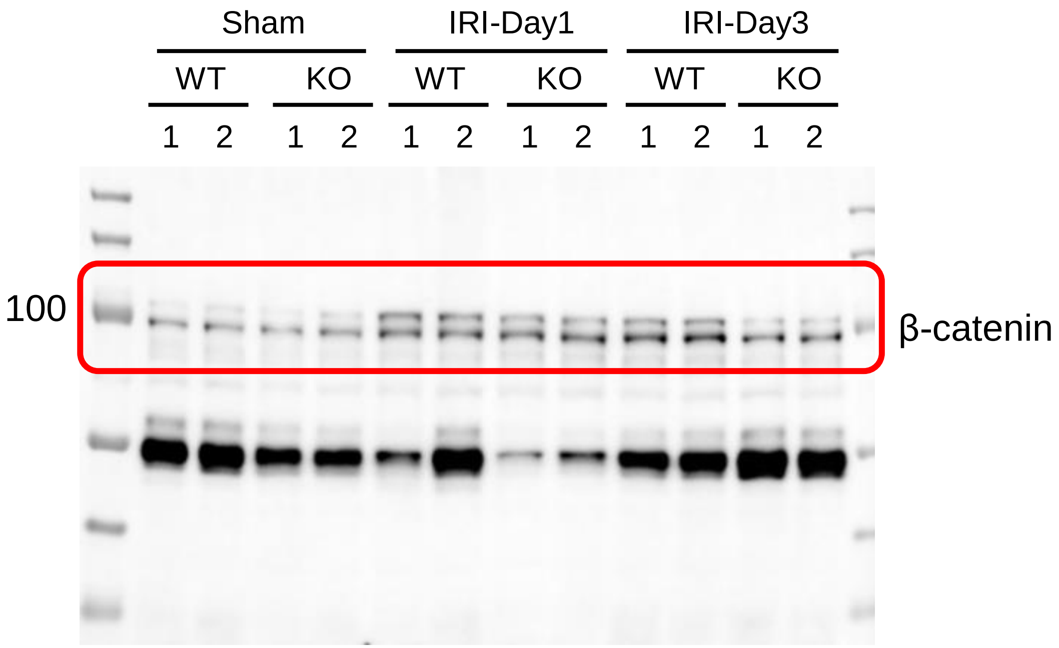

(data not shown)

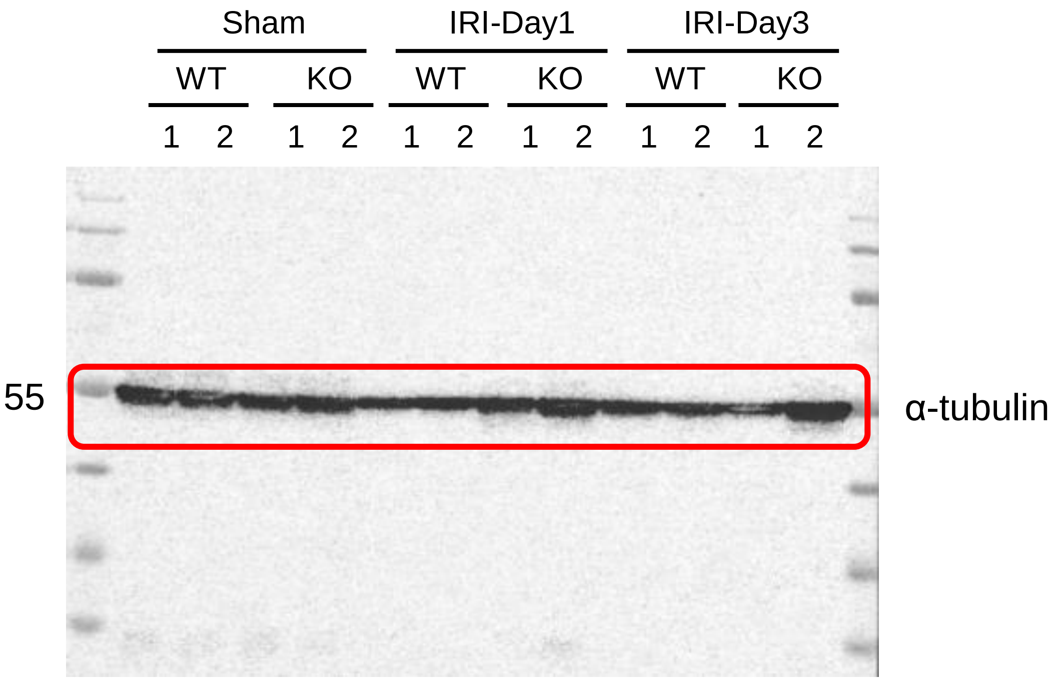

(data not shown)

Figure 5f

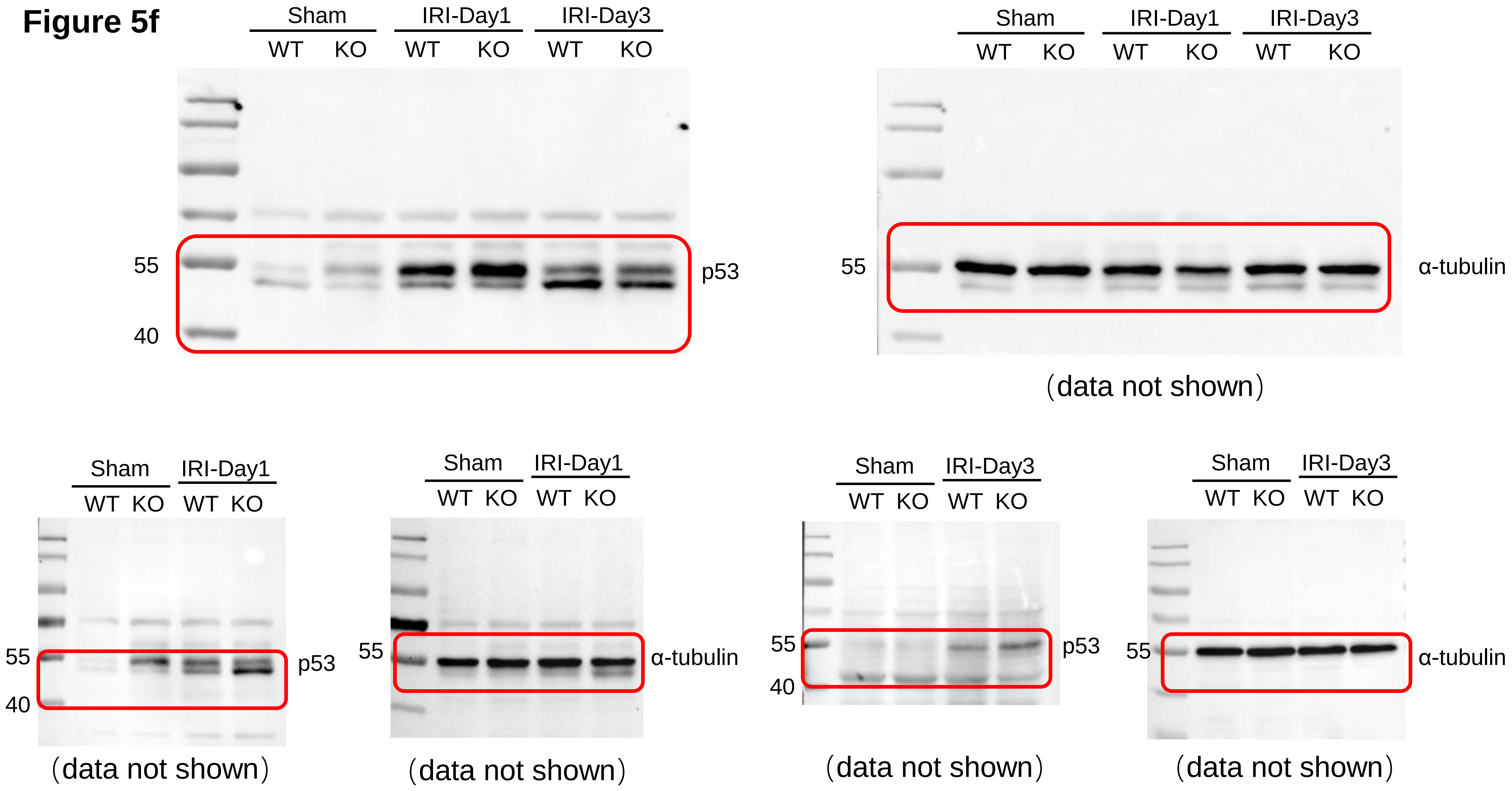
Sham
IRI-Day1
IRI-Day3
WT KO WT KO WT KO
55
40
p53
Sham
IRI-Day1
IRI-Day3
WT KO WT KO WT KO
55
α-tubulin
(data not shown)
Sham
IRI-Day1
WT KO WT KO
55
40
p53
(data not shown)
Sham
IRI-Day1
WT KO WT KO
55
α-tubulin
(data not shown)
Sham
IRI-Day3
WT KO WT KO
55
40
p53
(data not shown)
Sham
IRI-Day3
WT KO WT KO
55
α-tubulin
(data not shown)

Figure 5f

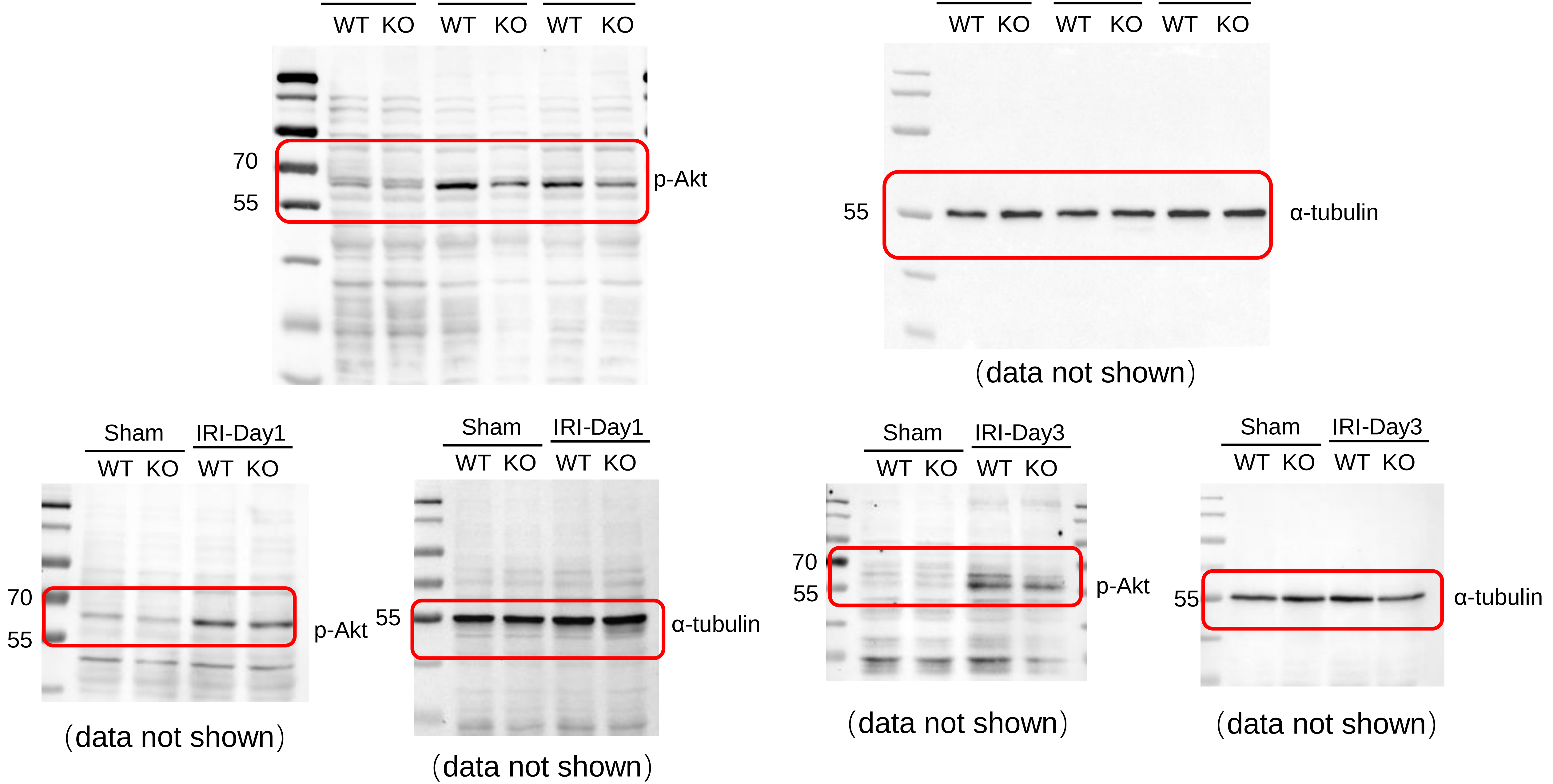

Sham
IRI-Day1
IRI-Day3
WT KO WT KO WT KO
70
55
p-Akt
Sham
IRI-Day1
IRI-Day3
WT KO WT KO WT KO
55
α-tubulin
(data not shown)
Sham
IRI-Day1
WT KO WT KO
70
55
p-Akt
(data not shown)
Sham
IRI-Day1
WT KO WT KO
55
α-tubulin
(data not shown)
Sham
IRI-Day3
WT KO WT KO
70
55
p-Akt
(data not shown)
Sham
IRI-Day3
WT KO WT KO
55
α-tubulin
(data not shown)

Figure 5f

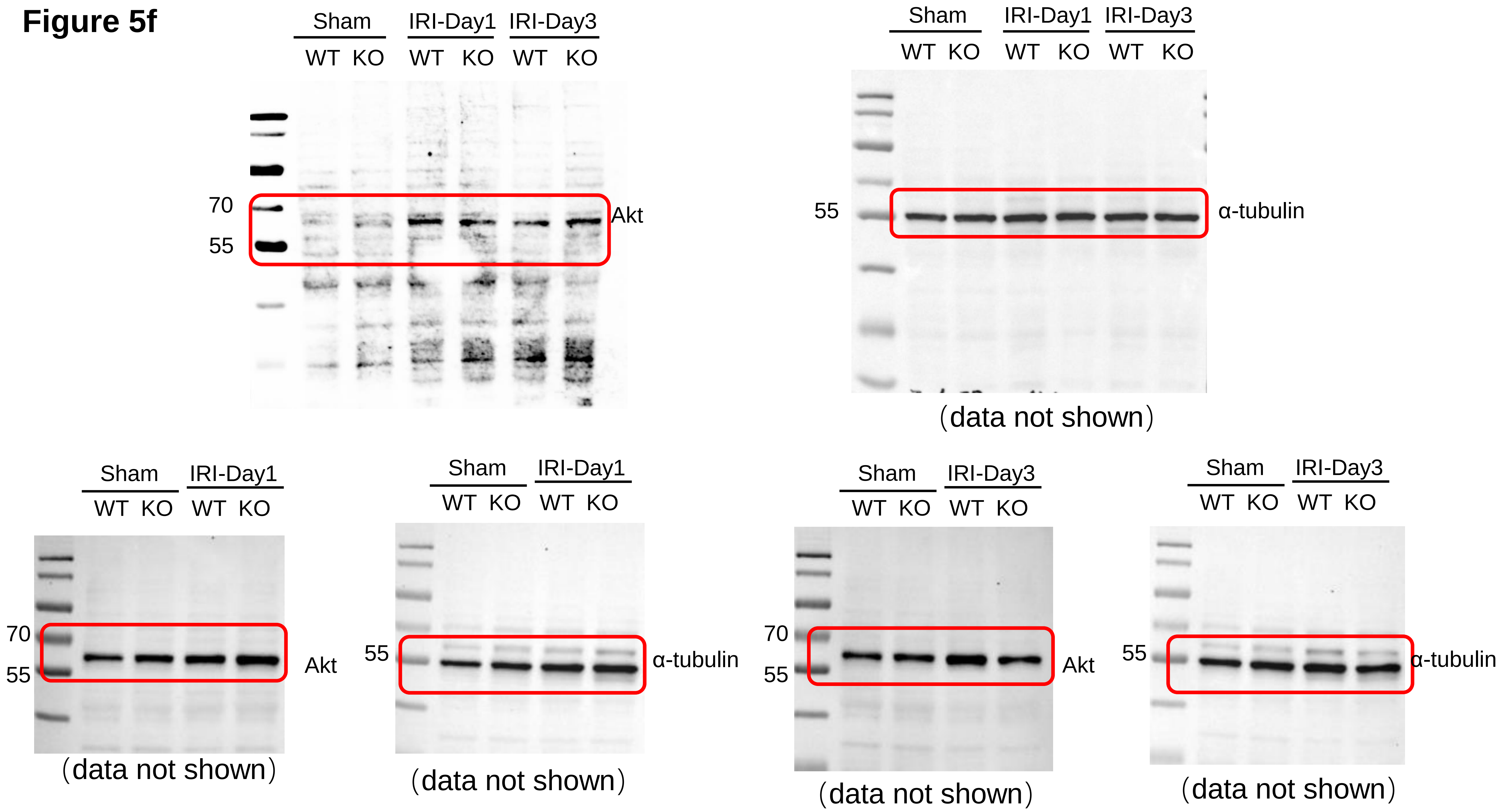
Sham
IRI-Day1
IRI-Day3
WT KO WT KO WT KO
70
55
Akt
Sham
IRI-Day1
IRI-Day3
WT KO WT KO WT KO
55
α-tubulin
(data not shown)
Sham
IRI-Day1
WT KO WT KO
70
55
Akt
(data not shown)
Sham
IRI-Day1
WT KO WT KO
55
α-tubulin
(data not shown)
Sham
IRI-Day3
WT KO WT KO
70
55
Akt
(data not shown)
Sham
IRI-Day3
WT KO WT KO
55
α-tubulin
(data not shown)

Figure 5f

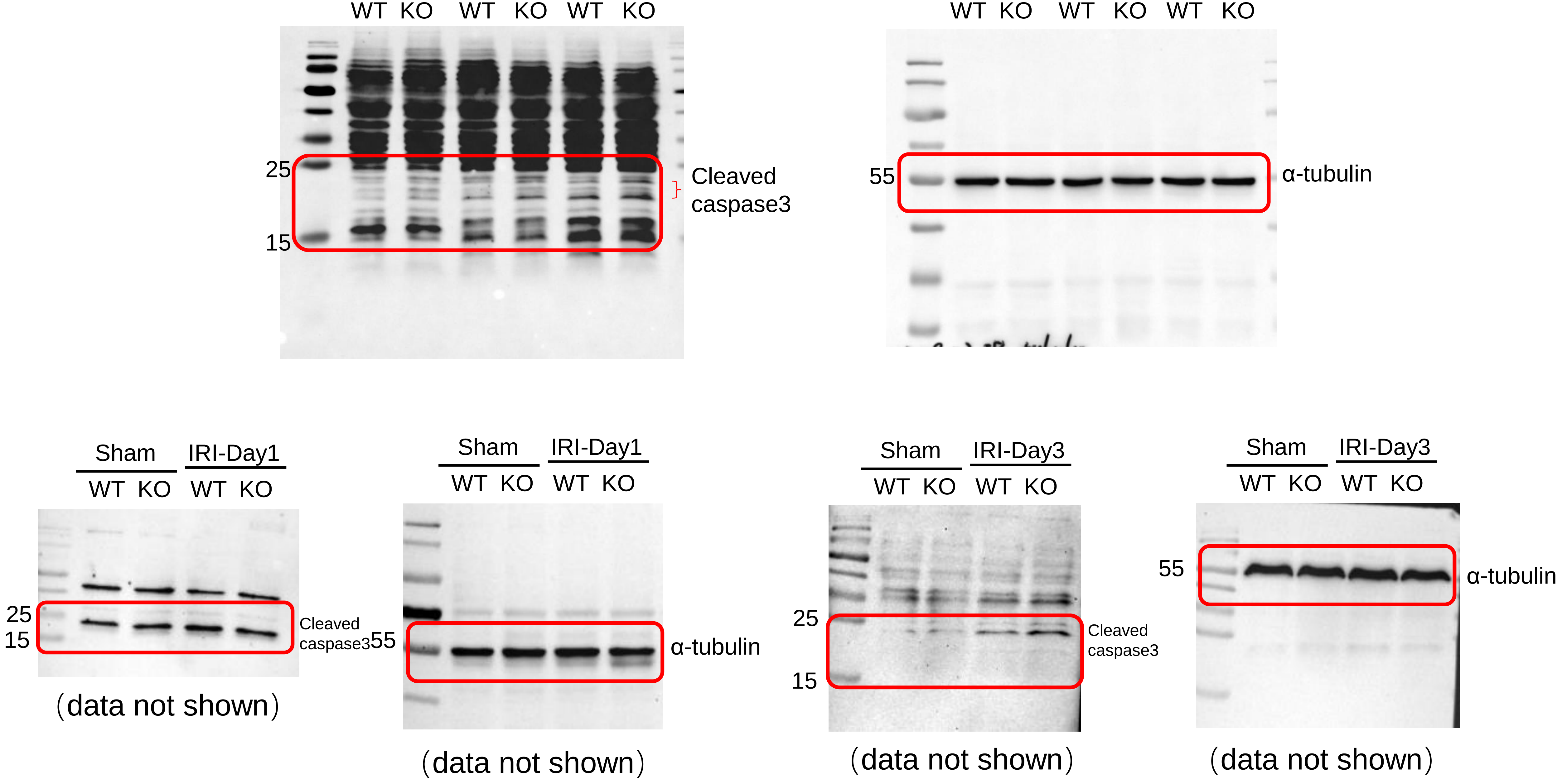
Sham
IRI-Day1
IRI-Day3
WT KO WT KO WT KO
25
15
Cleaved caspase3
Sham
IRI-Day1
IRI-Day3
WT KO WT KO WT KO
55
α-tubulin
Sham
IRI-Day1
WT KO WT KO
25
15
Cleaved caspase3
(data not shown)
Sham
IRI-Day1
WT KO WT KO
55
α-tubulin
(data not shown)
Sham
IRI-Day3
WT KO WT KO
25
15
Cleaved caspase3
(data not shown)
Sham
IRI-Day3
WT KO WT KO
55
α-tubulin
(data not shown)

Figure 5f

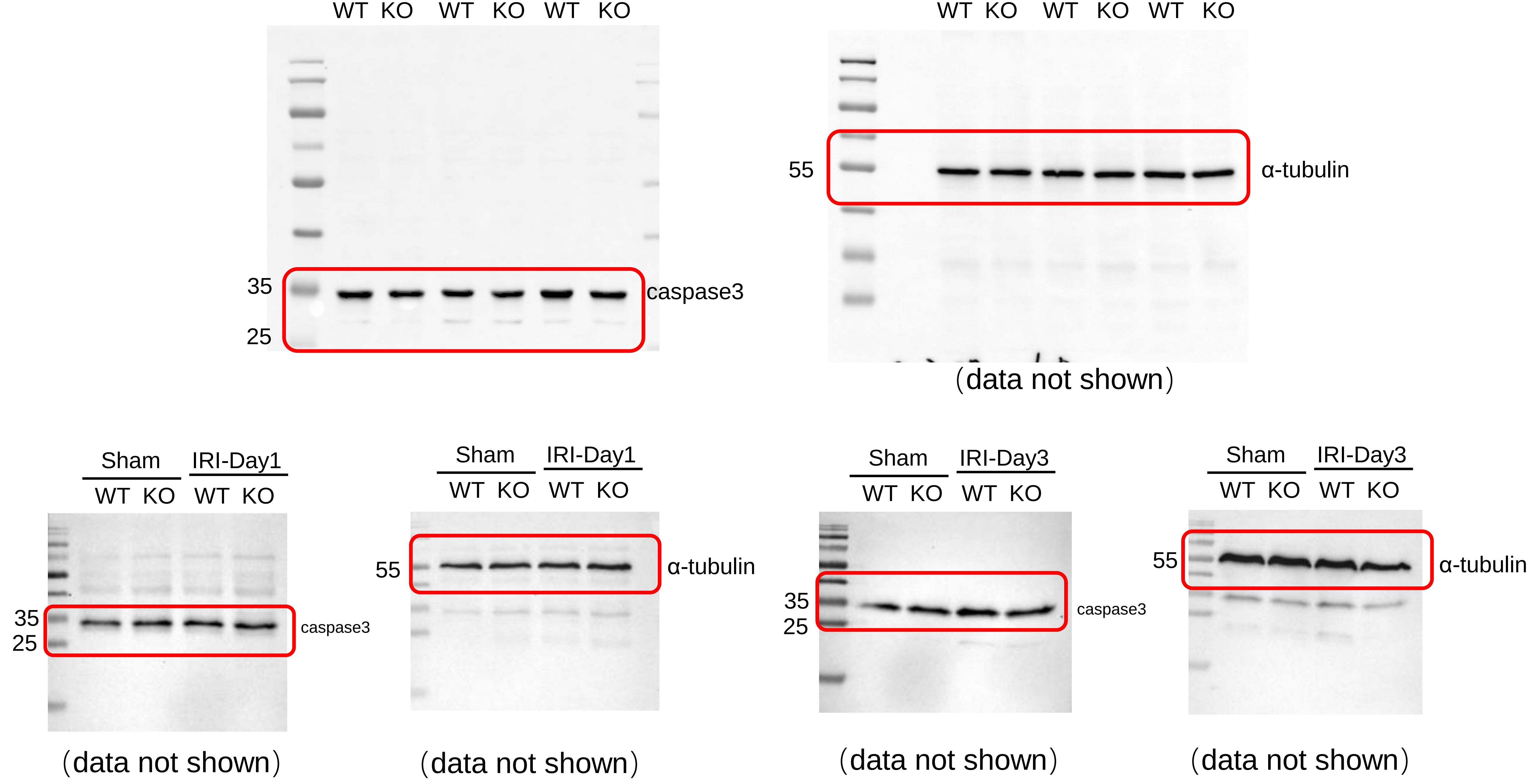
Sham IRI-Day1 IRI-Day3
WT KO WT KO WT KO
35
25
caspase3
Sham IRI-Day1 IRI-Day3
WT KO WT KO WT KO
55
α-tubulin
(data not shown)
Sham IRI-Day1
WT KO WT KO
35
25
caspase3
(data not shown)
Sham IRI-Day1
WT KO WT KO
55
α-tubulin
(data not shown)
Sham IRI-Day3
WT KO WT KO
35
25
caspase3
(data not shown)
Sham IRI-Day3
WT KO WT KO
55
α-tubulin
(data not shown)

Figure 5f

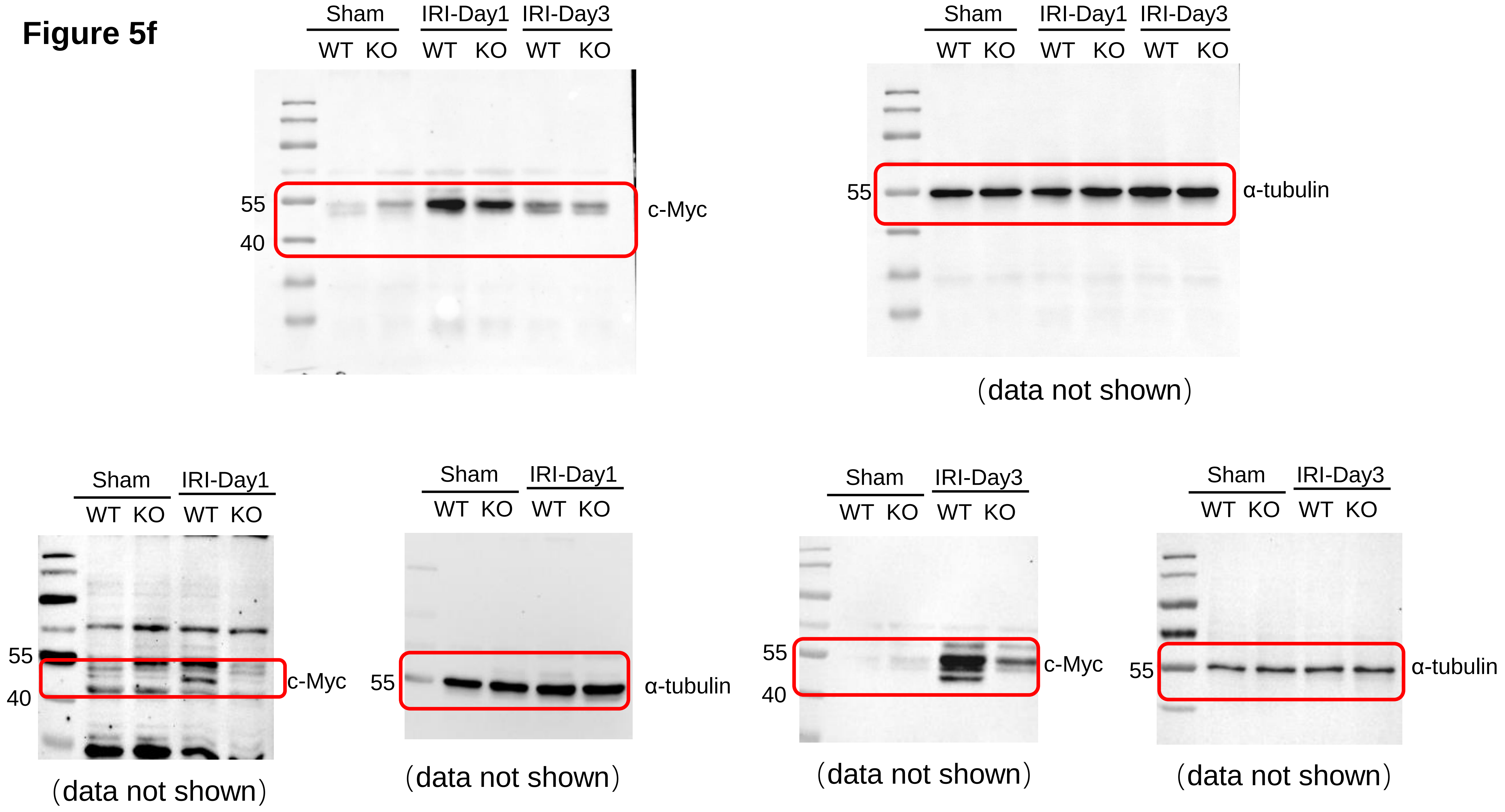

Sham
IRI-Day1
IRI-Day3
WT KO WT KO WT KO
55
40
c-Myc
Sham
IRI-Day1
IRI-Day3
WT KO WT KO WT KO
55
α-tubulin
(data not shown)
Sham
IRI-Day1
WT KO WT KO
55
40
c-Myc
(data not shown)
Sham
IRI-Day1
WT KO WT KO
55
α-tubulin
(data not shown)
Sham
IRI-Day3
WT KO WT KO
55
40
c-Myc
(data not shown)
Sham
IRI-Day3
WT KO WT KO
55
α-tubulin
(data not shown)

Figure 5f

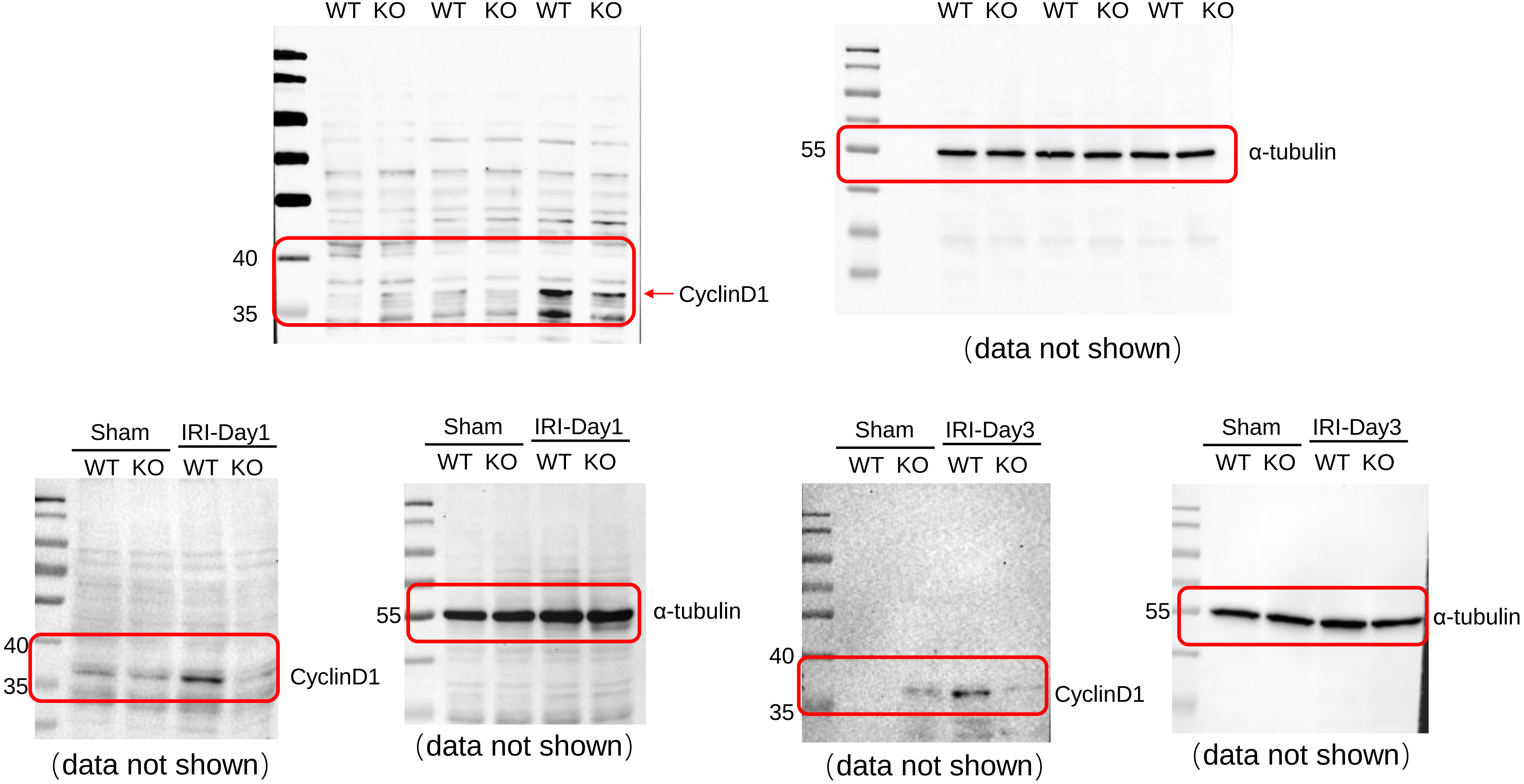

Sham
IRI-Day1
IRI-Day3
WT KO WT KO WT KO
40
35
CyclinD1
55
α-tubulin
(data not shown)
Sham
IRI-Day1
WT KO WT KO
40
35
CyclinD1
(data not shown)
55
α-tubulin
(data not shown)
Sham
IRI-Day3
WT KO WT KO
40
35
CyclinD1
(data not shown)
55
α-tubulin
(data not shown)

Figure 5g

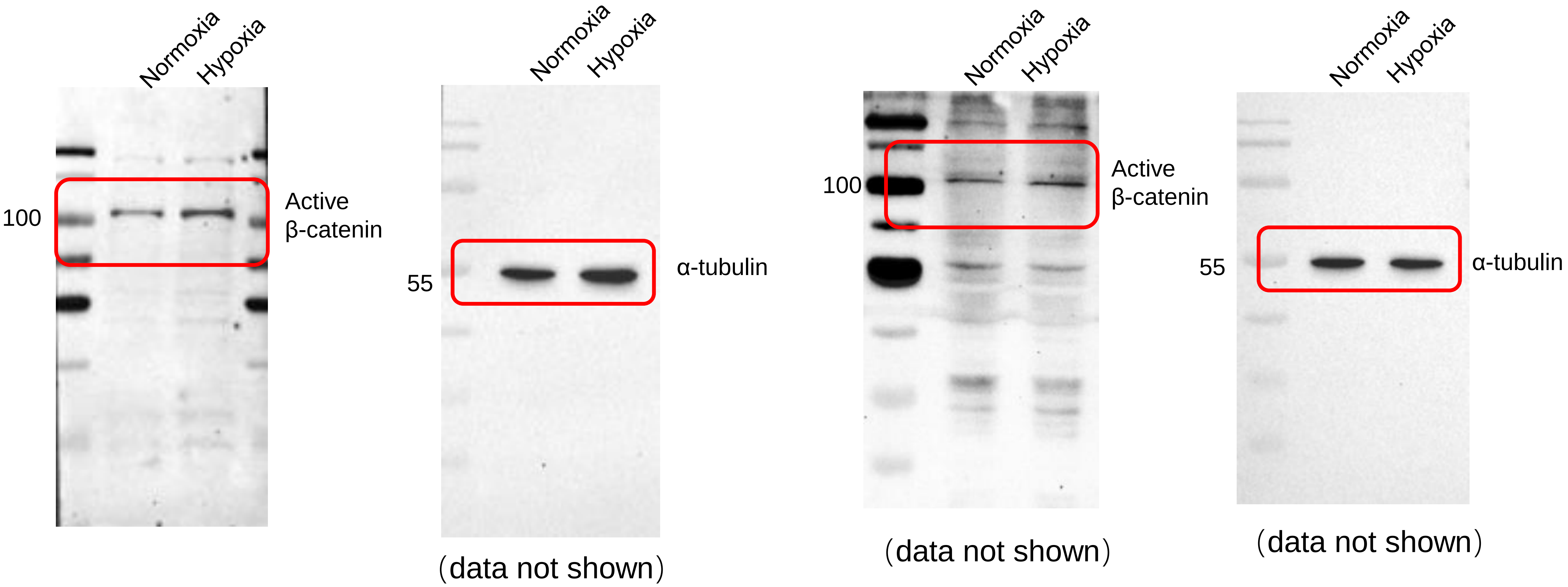
Normoxia
Hypoxia
100
Active
β-catenin
Normoxia
Hypoxia
55
α-tubulin
(data not shown)
Normoxia
Hypoxia
100
Active
β-catenin
(data not shown)
Normoxia
Hypoxia
55
α-tubulin
(data not shown)

Figure 5g

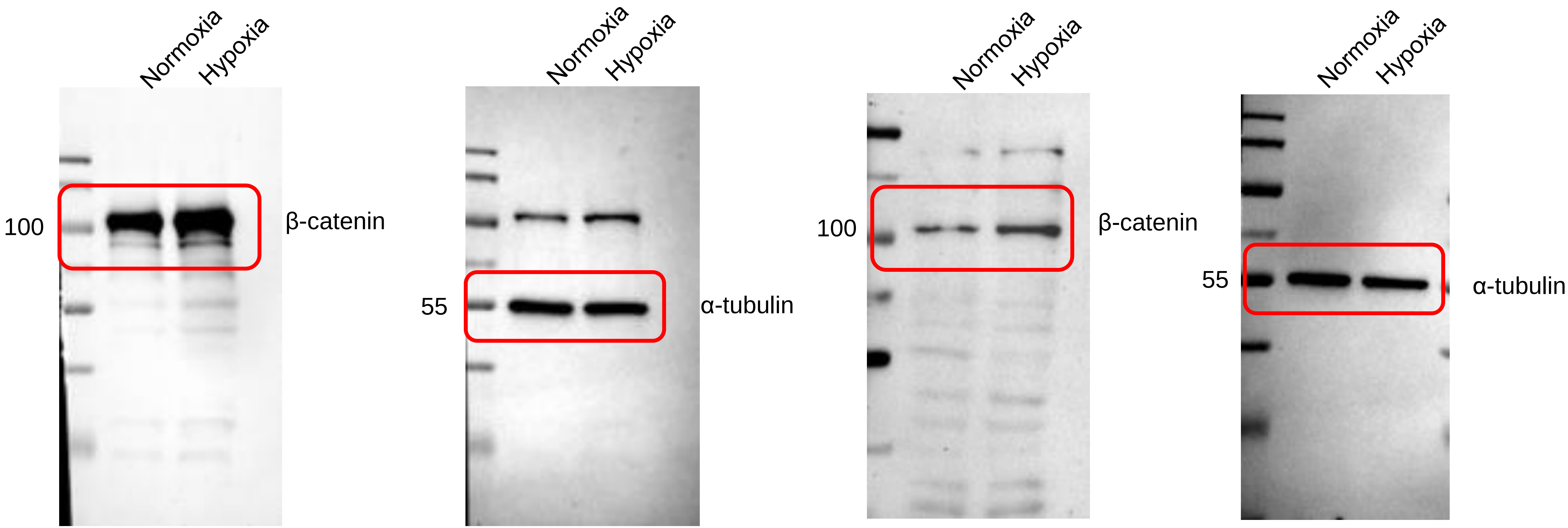

(data not shown)　(data not shown)

Figure 5i

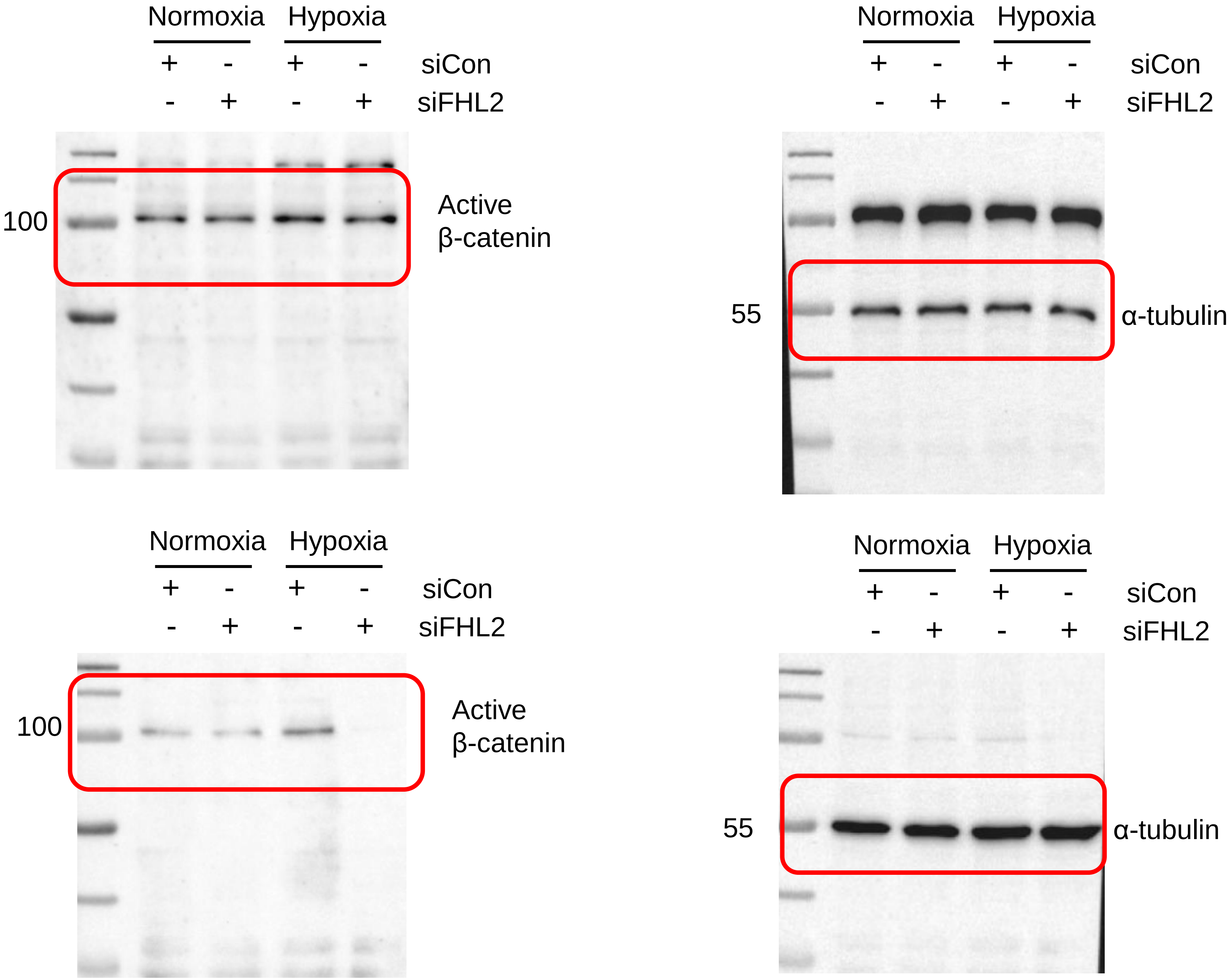

(data not shown)

(data not shown)

Figure 5k

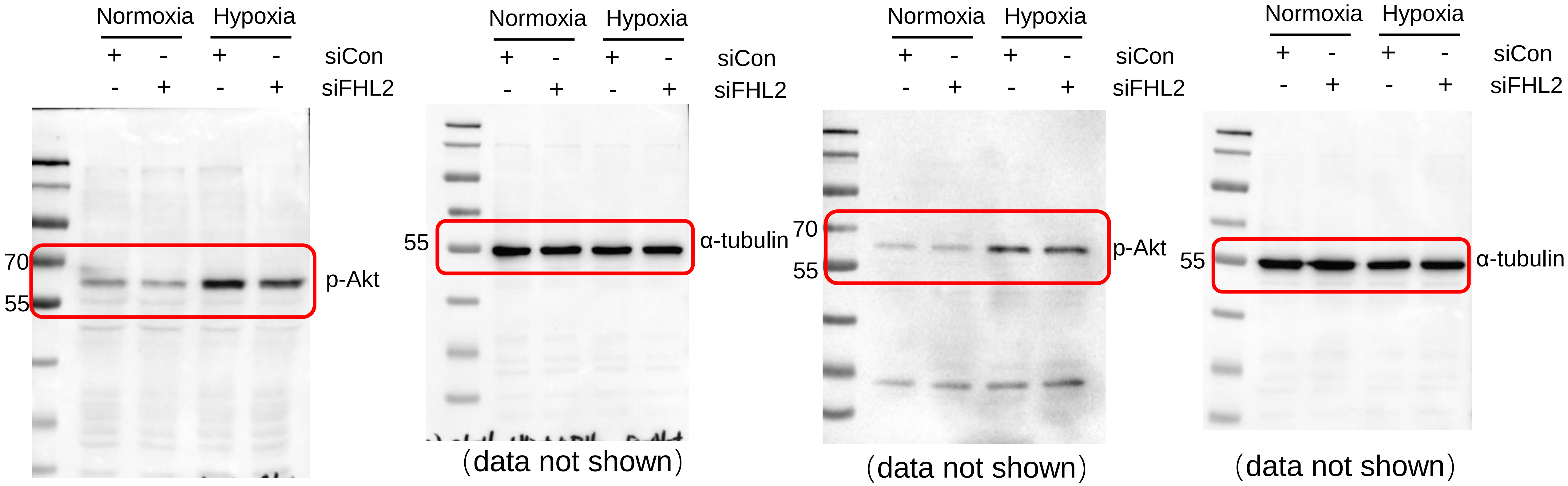
Normoxia
Hypoxia
+ - + - siCon
- + - + siFHL2
70
55
p-Akt
Normoxia
Hypoxia
+ - + - siCon
- + - + siFHL2
55
α-tubulin
(data not shown)
Normoxia
Hypoxia
+ - + - siCon
- + - + siFHL2
70
55
p-Akt
(data not shown)
Normoxia
Hypoxia
+ - + - siCon
- + - + siFHL2
55
α-tubulin
(data not shown)

Figure 5k

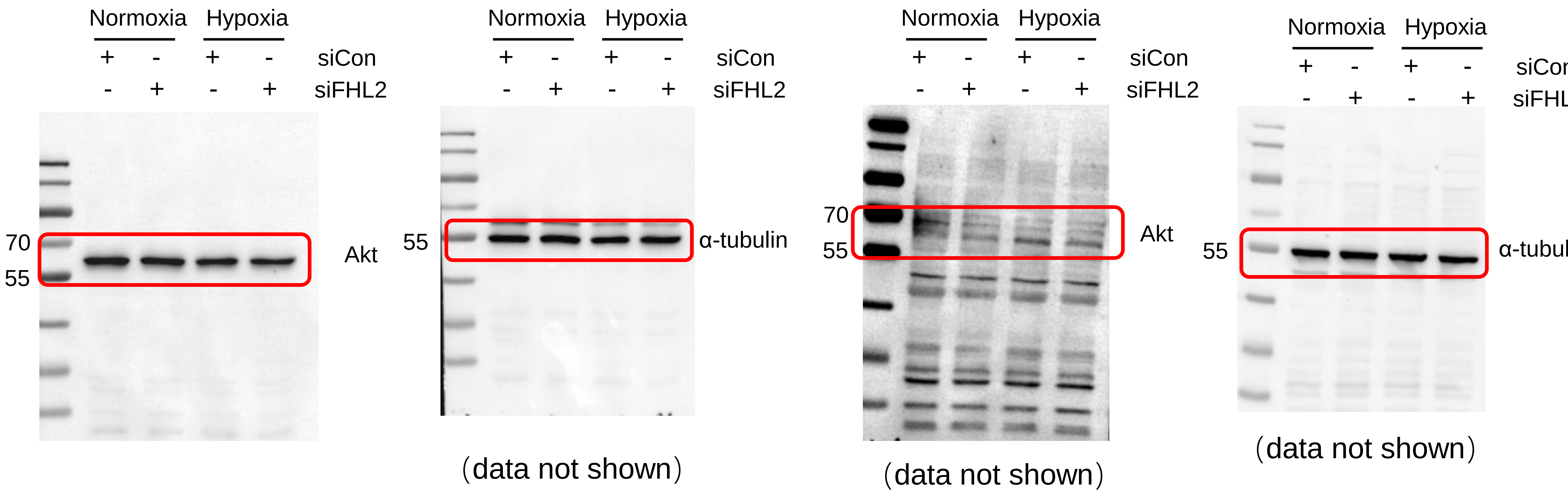

Normoxia
Hypoxia
+ - + - siCon
- + - + siFHL2
70
55
Akt
Normoxia
Hypoxia
+ - + - siCon
- + - + siFHL2
55
α-tubulin
(data not shown)
Normoxia
Hypoxia
+ - + - siCon
- + - + siFHL2
70
55
Akt
(data not shown)
Normoxia
Hypoxia
+ - + -
- + - +
55
(data not shown)

Figure 5k

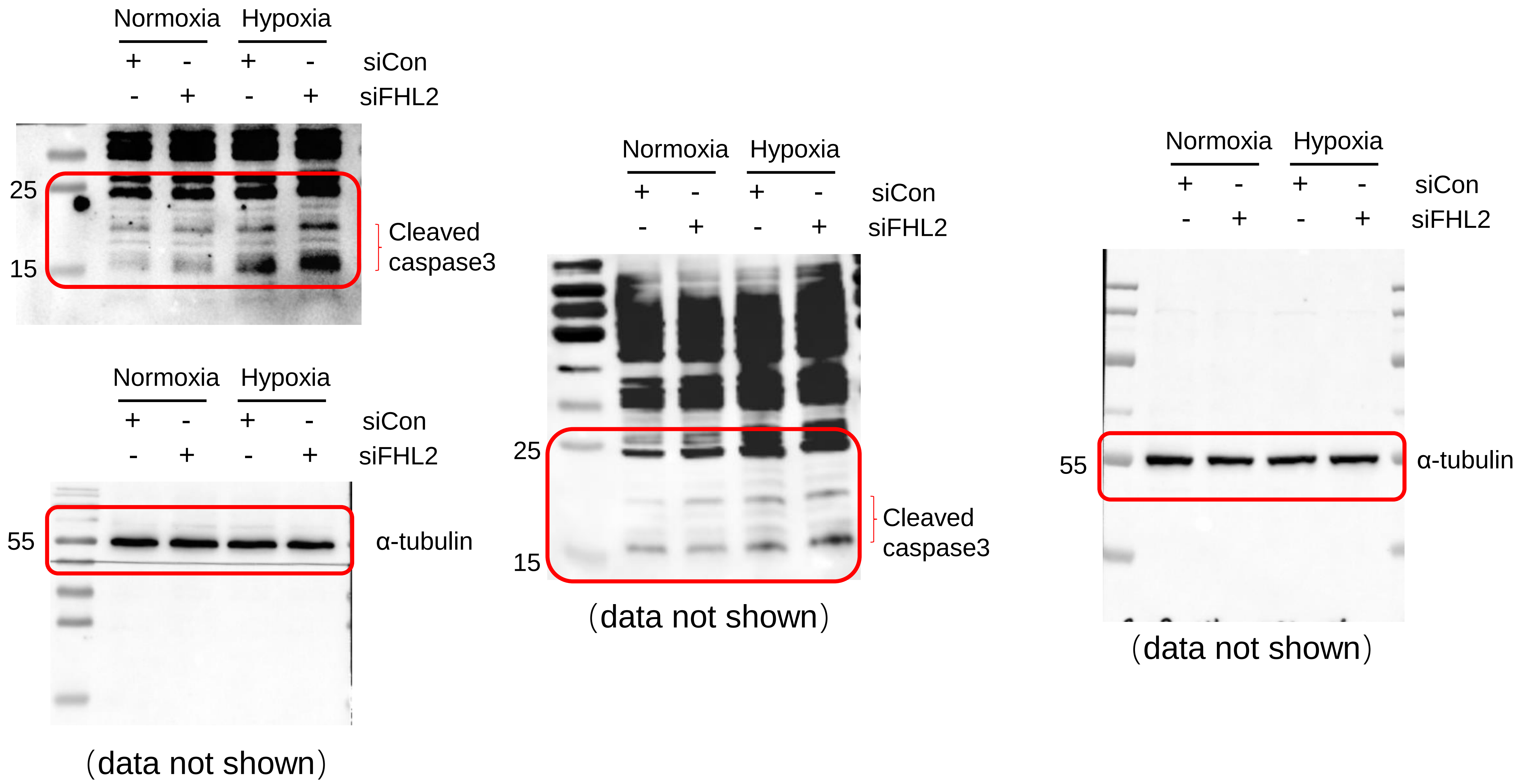
Normoxia
Hypoxia
+ - + - siCon
- + - + siFHL2
25
15
Cleaved caspase3
Normoxia
Hypoxia
+ - + - siCon
- + - + siFHL2
55
α-tubulin
(data not shown)
Normoxia
Hypoxia
+ - + - siCon
- + - + siFHL2
25
15
Cleaved caspase3
(data not shown)
Normoxia
Hypoxia
+ - + - siCon
- + - + siFHL2
55
α-tubulin
(data not shown)

Figure 5k

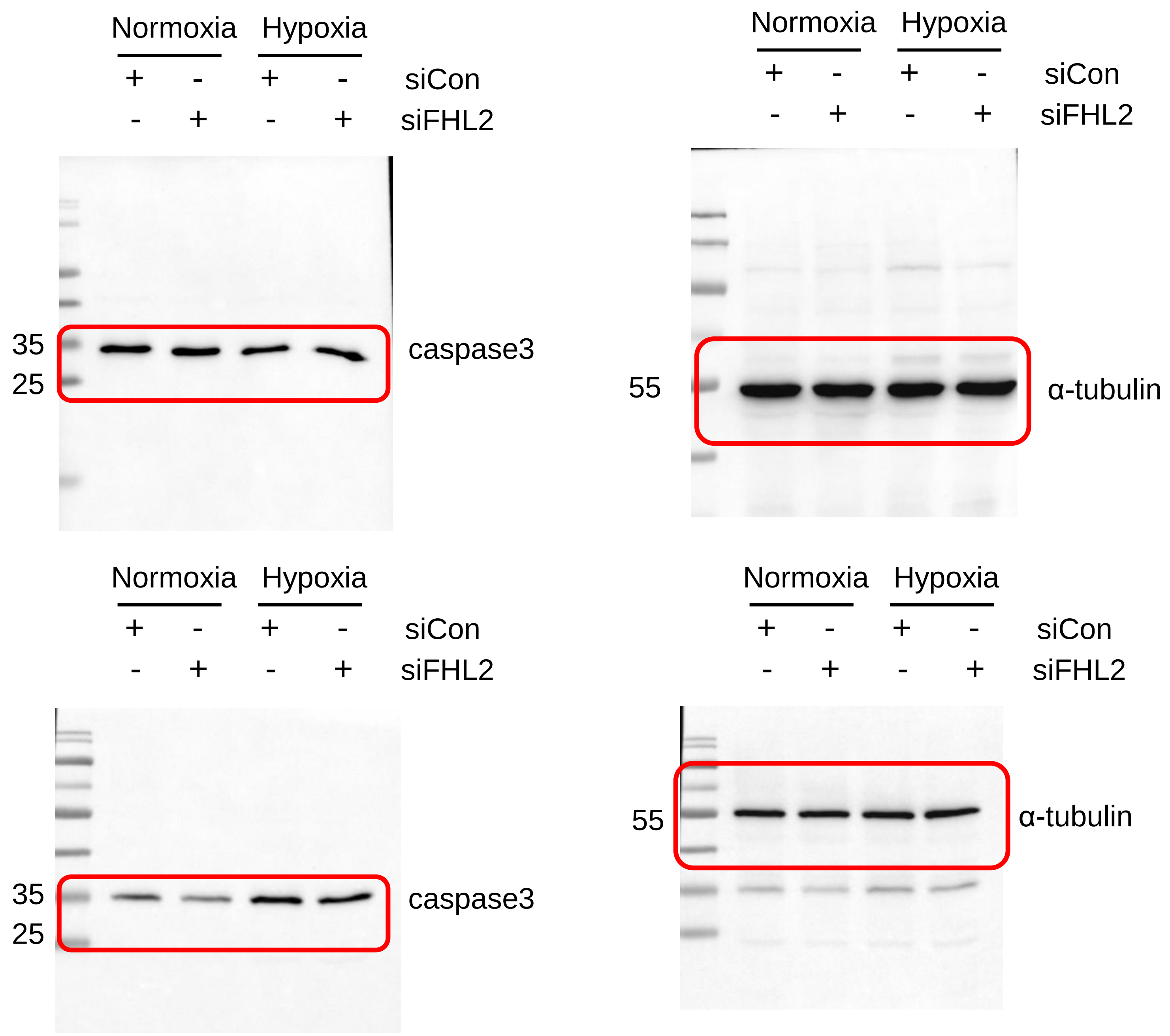

(data not shown)

(data not shown)

Figure 5k

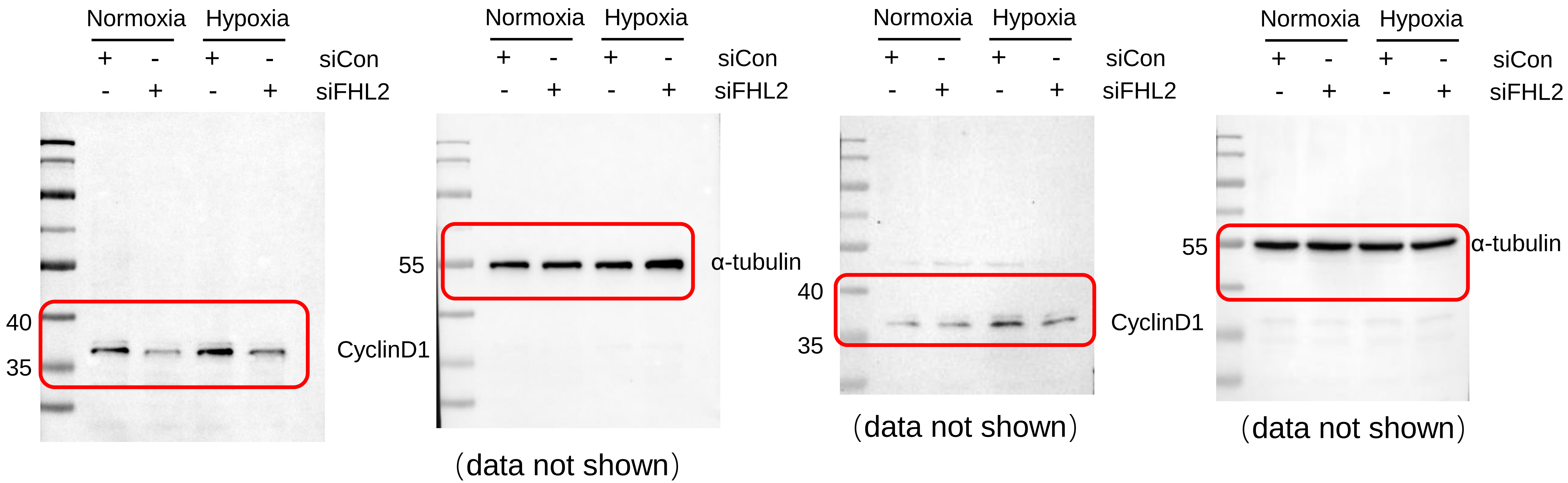
Normoxia
Hypoxia
+ - + - siCon
- + - + siFHL2
40
35
CyclinD1
55
α-tubulin
(data not shown)
Normoxia
Hypoxia
+ - + - siCon
- + - + siFHL2
40
35
CyclinD1
(data not shown)
Normoxia
Hypoxia
+ - + - siCon
- + - + siFHL2
55
α-tubulin
(data not shown)

Figure 6c

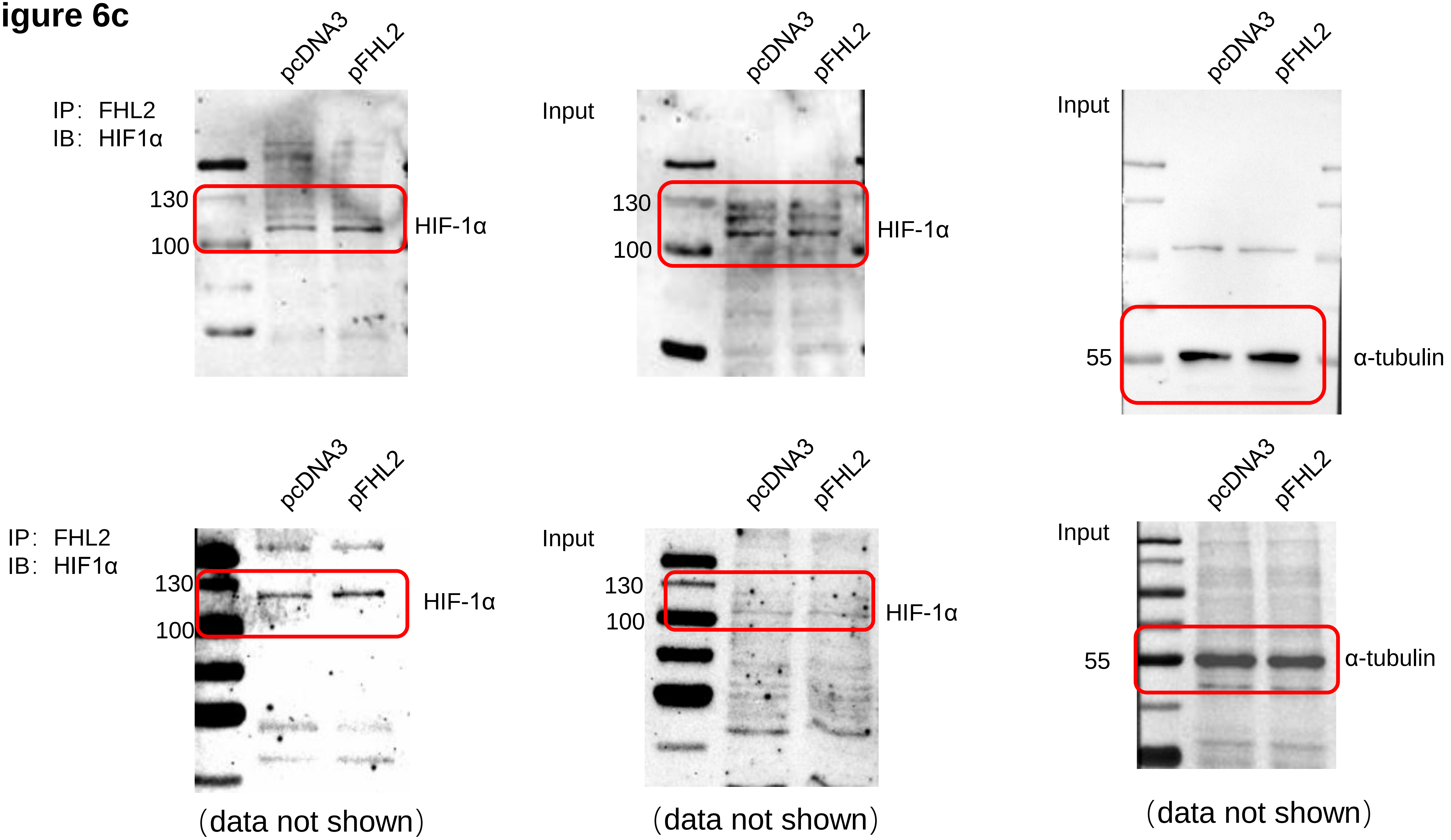

pcDNA3
pFHL2
IP: FHL2
IB: HIF1α
130
100
HIF-1α
Input
pcDNA3
pFHL2
130
100
HIF-1α
Input
pcDNA3
pFHL2
55
α-tubulin
pcDNA3
pFHL2
IP: FHL2
IB: HIF1α
130
100
HIF-1α
Input
pcDNA3
pFHL2
130
100
HIF-1α
Input
pcDNA3
pFHL2
55
α-tubulin
(data not shown)
(data not shown)
(data not shown)

Figure 6d

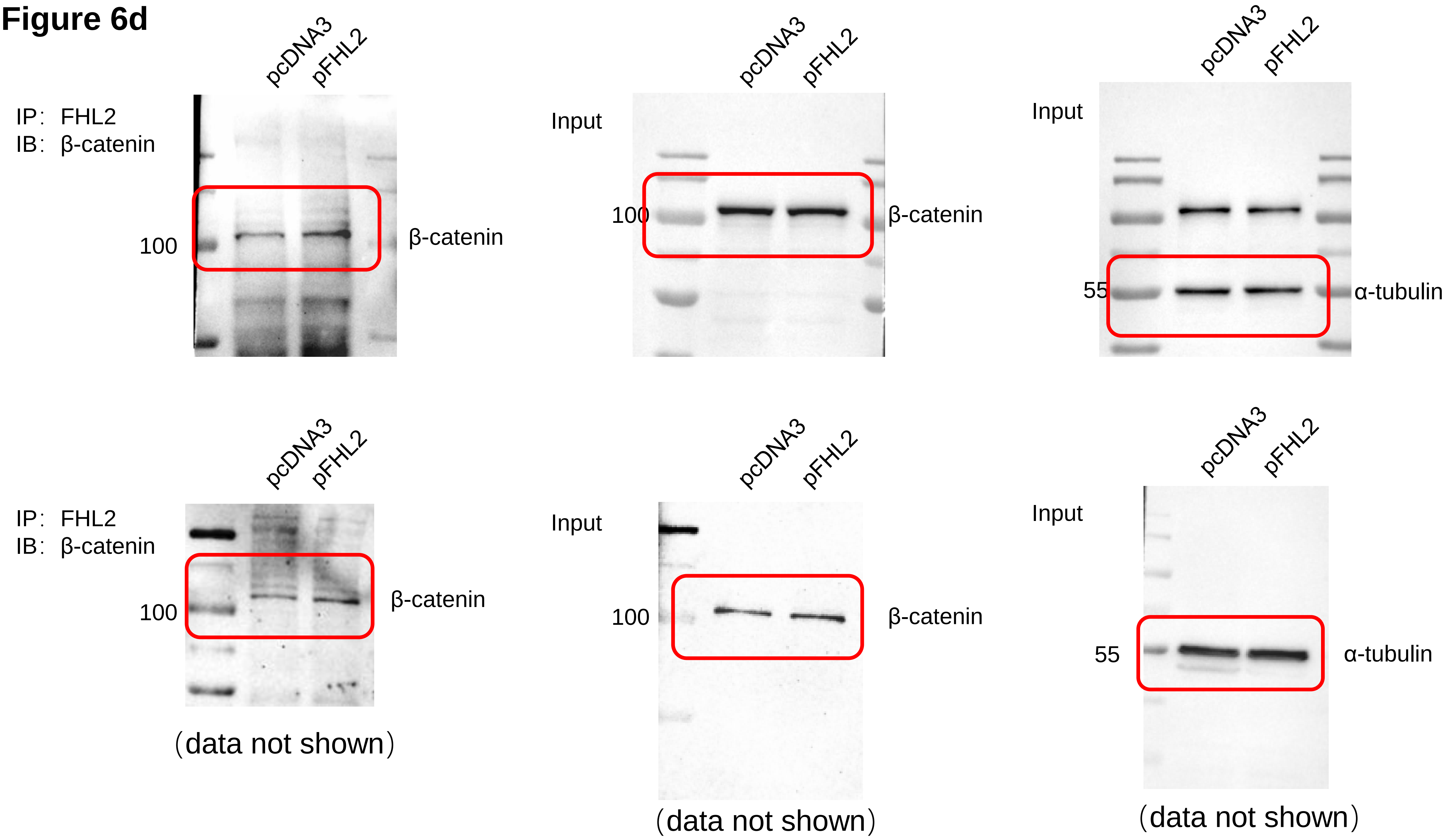

pcDNA3
pFHL2
IP: FHL2
IB: β-catenin
100
β-catenin
Input
100
β-catenin
Input
55
α-tubulin
(data not shown)
(data not shown)
(data not shown)

Figure 6e

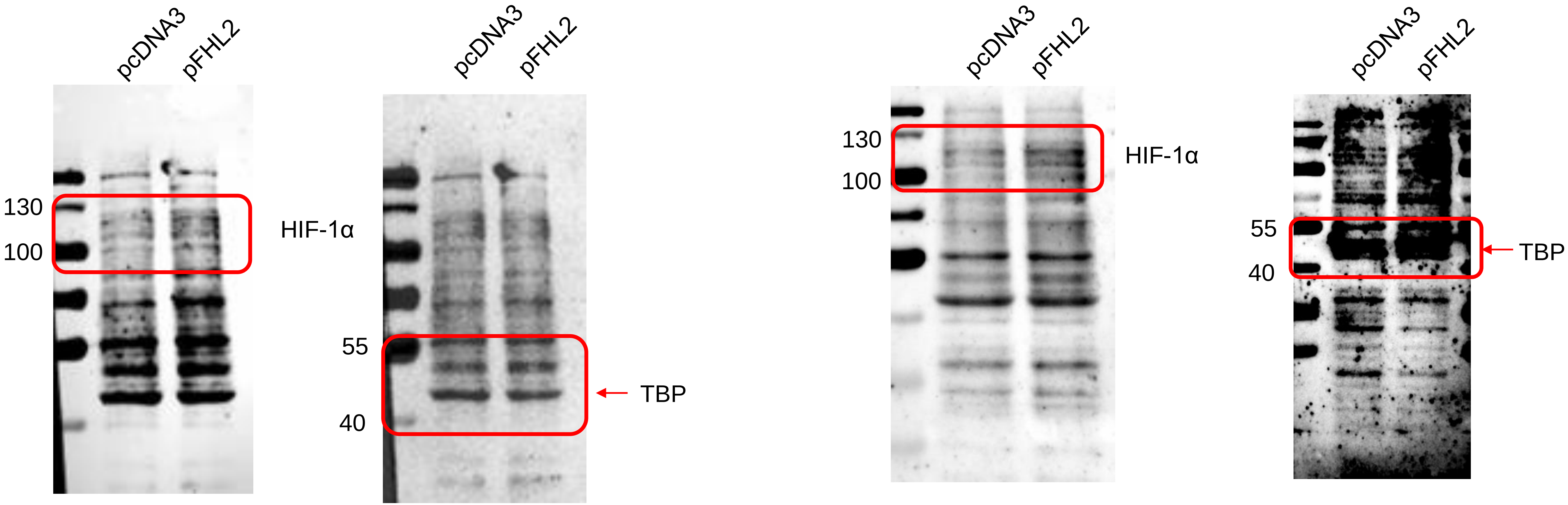

(data not shown)　(data not shown)

Figure 6f

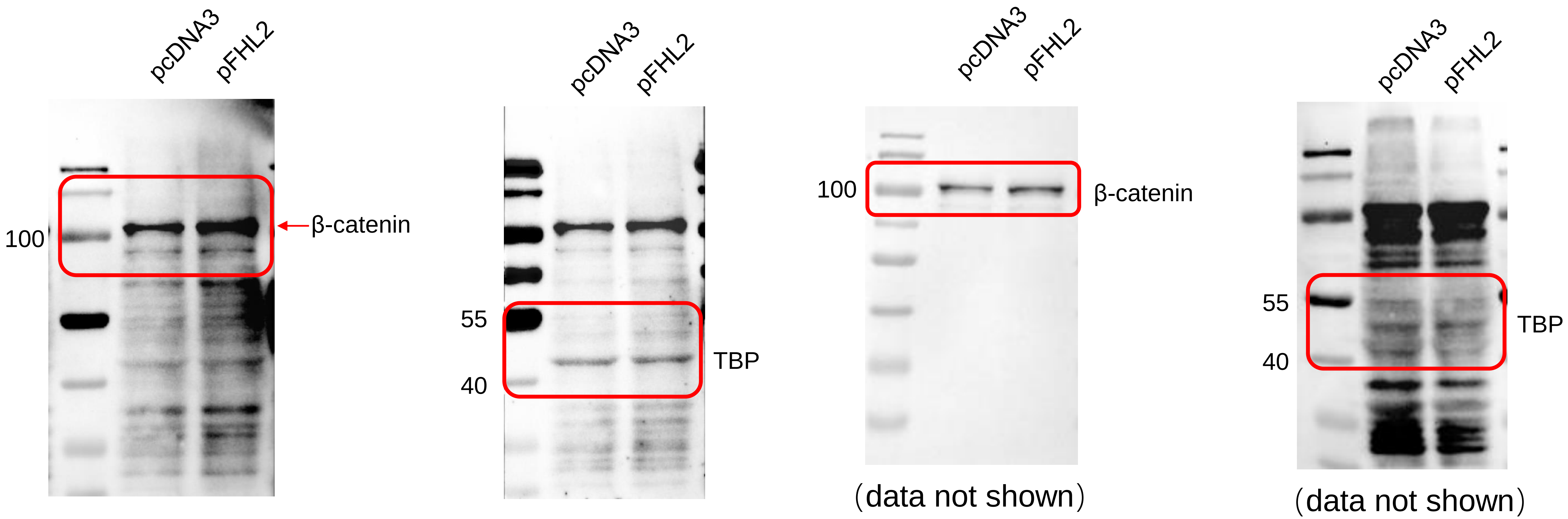
pcDNA3
pFHL2
100
β-catenin
pcDNA3
pFHL2
55
40
TBP
pcDNA3
pFHL2
100
β-catenin
(data not shown)
pcDNA3
pFHL2
55
40
TBP
(data not shown)

Figure 6g

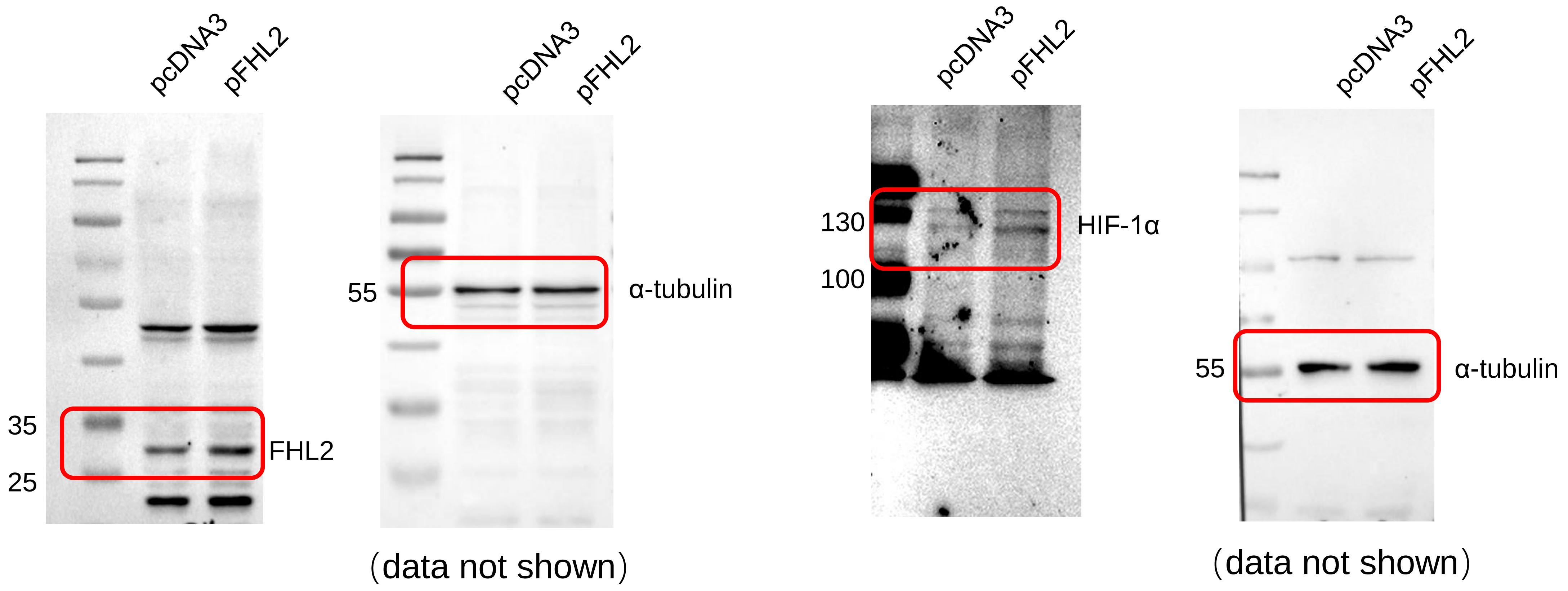

pcDNA3
pFHL2
35
25
FHL2
pcDNA3
pFHL2
55
α-tubulin
(data not shown)
pcDNA3
pFHL2
130
100
HIF-1α
pcDNA3
pFHL2
55
α-tubulin
(data not shown)

Figure 6g

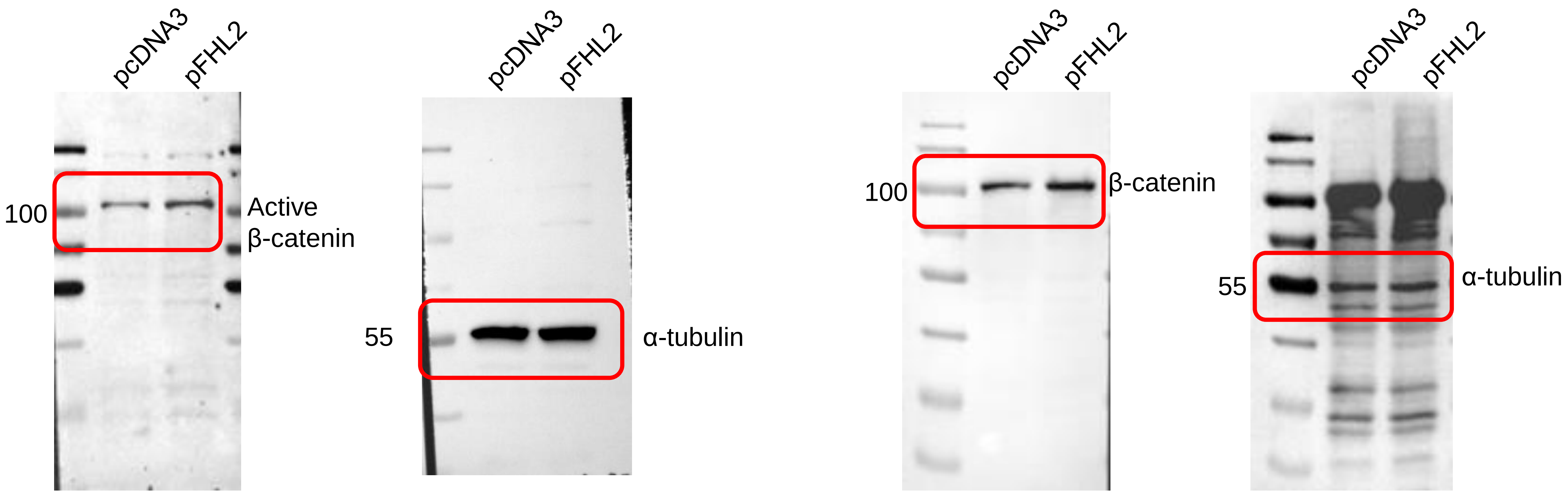

(data not shown)

Figure 6j

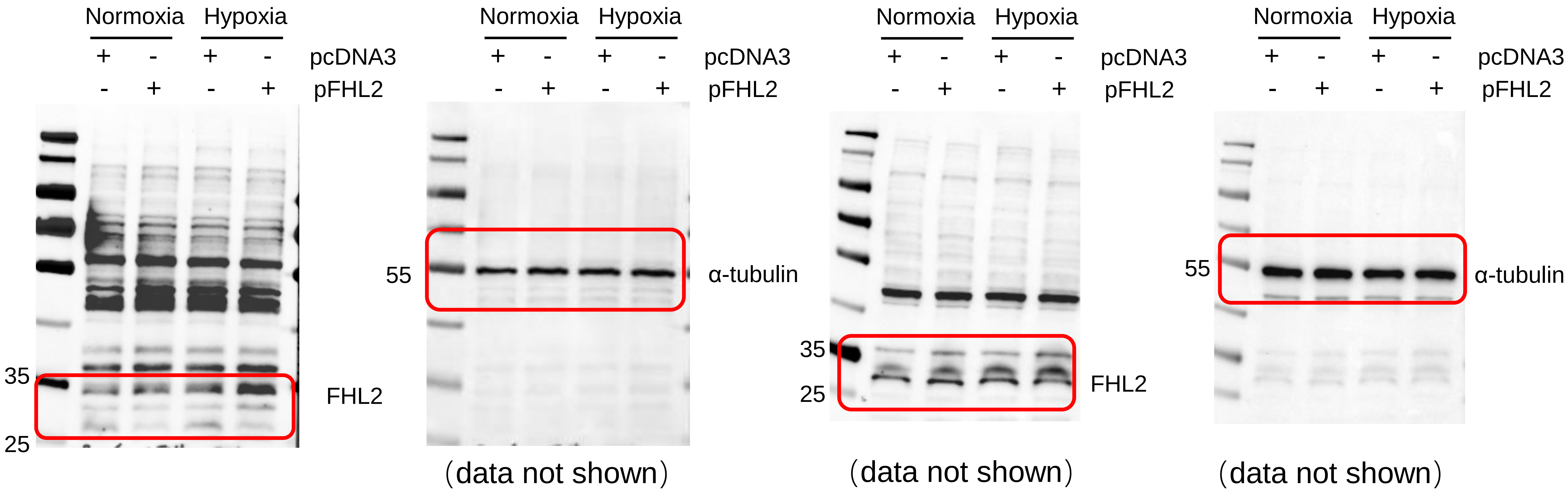
Normoxia Hypoxia
+ - + - pcDNA3
- + - + pFHL2
35
25
FHL2
55
α-tubulin
(data not shown)
Normoxia Hypoxia
+ - + - pcDNA3
- + - + pFHL2
35
25
FHL2
(data not shown)
Normoxia Hypoxia
+ - + - pcDNA3
- + - + pFHL2
55
α-tubulin
(data not shown)
Normoxia Hypoxia
+ - + - pcDNA3
- + - + pFHL2

Figure 6j

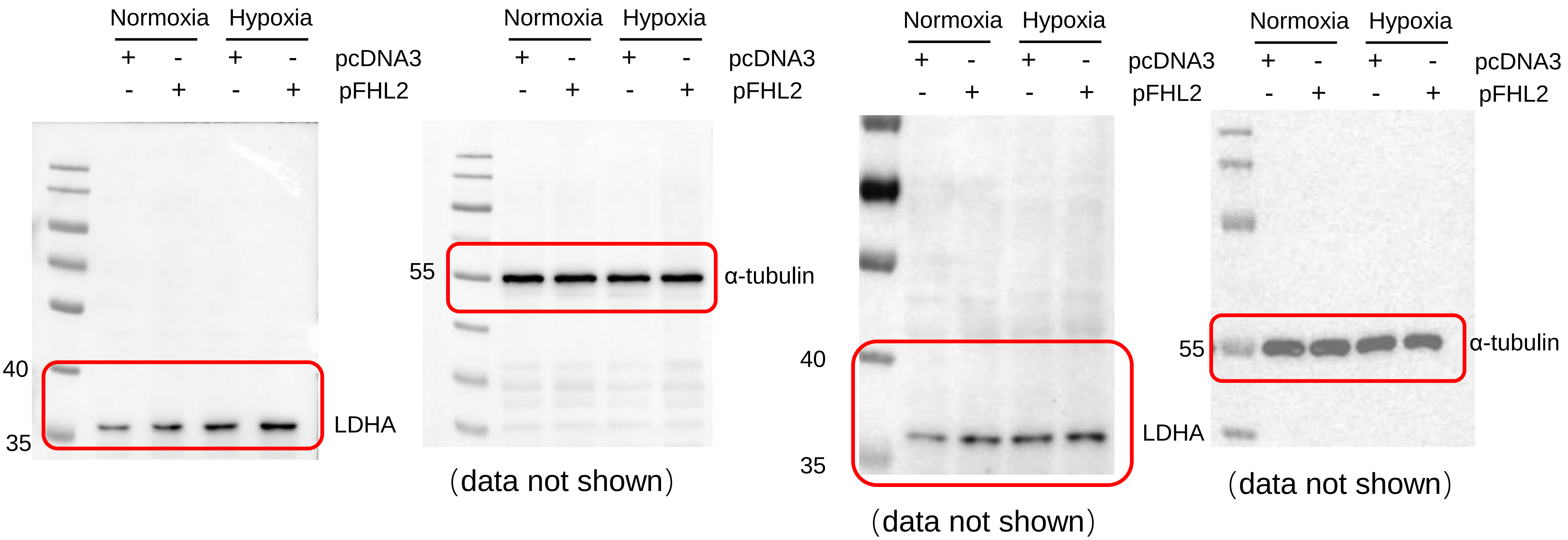
Normoxia
Hypoxia
+ - + - pcDNA3
- + - + pFHL2
40
35
LDHA
Normoxia
Hypoxia
+ - + - pcDNA3
- + - + pFHL2
55
α-tubulin
(data not shown)
Normoxia
Hypoxia
+ - + - pcDNA3
- + - + pFHL2
40
35
LDHA
(data not shown)
Normoxia
Hypoxia
+ - + - pcDNA3
- + - + pFHL2
55
α-tubulin
(data not shown)

Figure 6j

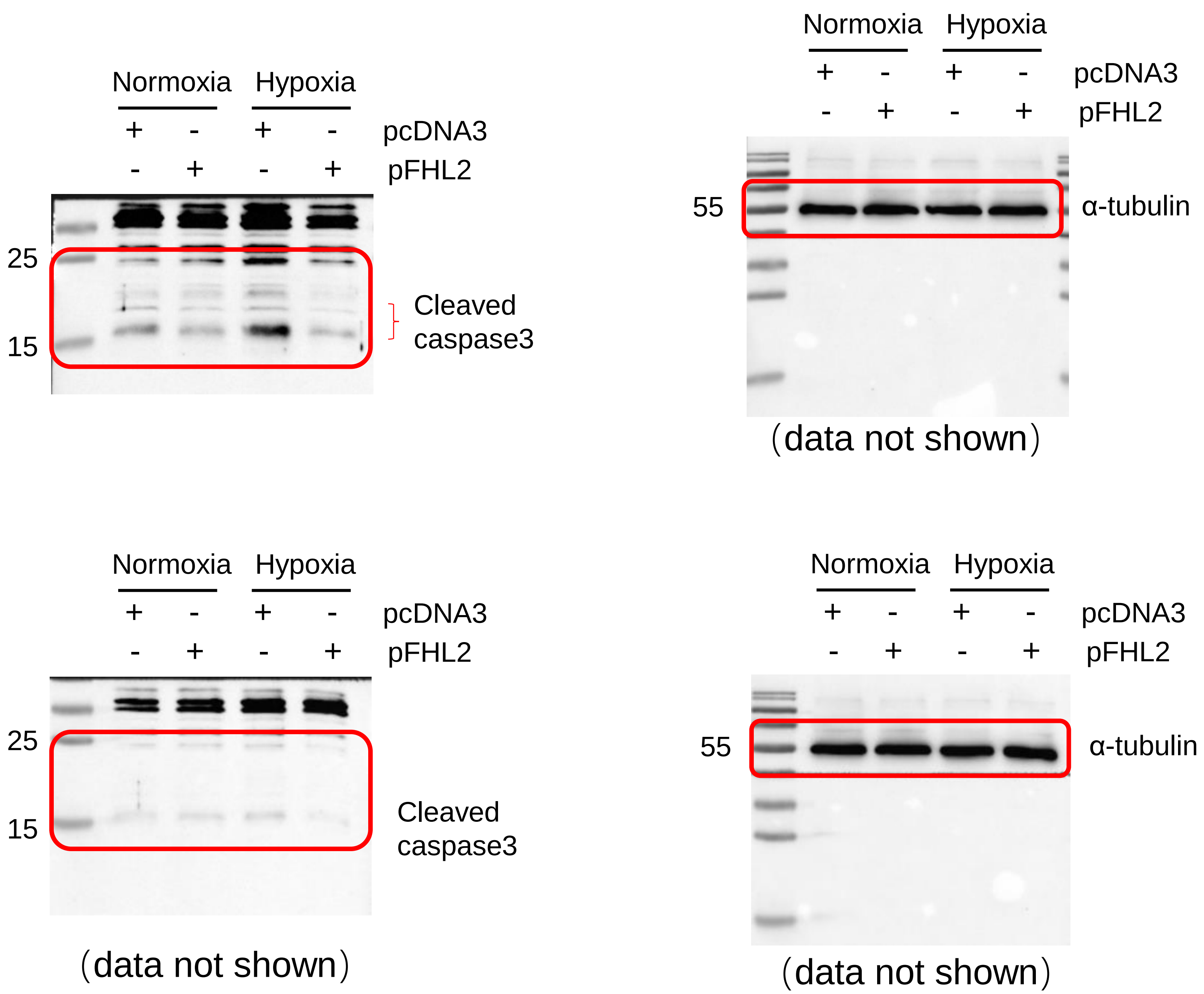
Normoxia
Hypoxia
pcDNA3
pFHL2
25
15
Cleaved caspase3
Normoxia
Hypoxia
pcDNA3
pFHL2
55
α-tubulin
(data not shown)
Normoxia
Hypoxia
pcDNA3
pFHL2
25
15
Cleaved caspase3
(data not shown)
Normoxia
Hypoxia
pcDNA3
pFHL2
55
α-tubulin
(data not shown)

Figure 6j

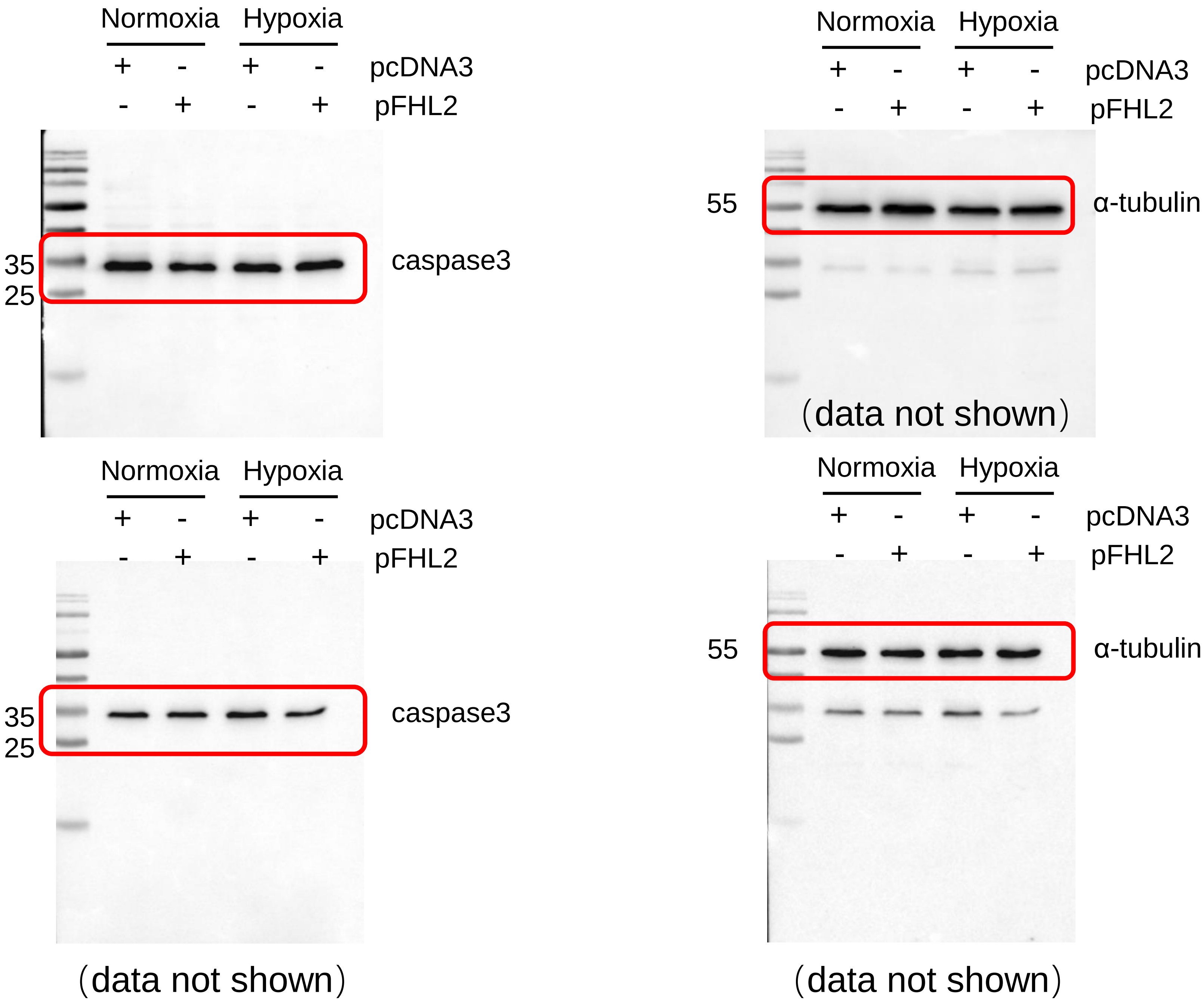
Normoxia
Hypoxia
+ - + - pcDNA3
- + - + pFHL2
35
25
caspase3
55
α-tubulin
(data not shown)
Normoxia
Hypoxia
+ - + - pcDNA3
- + - + pFHL2
35
25
caspase3
(data not shown)
Normoxia
Hypoxia
+ - + - pcDNA3
- + - + pFHL2
55
α-tubulin
(data not shown)

Figure 6j

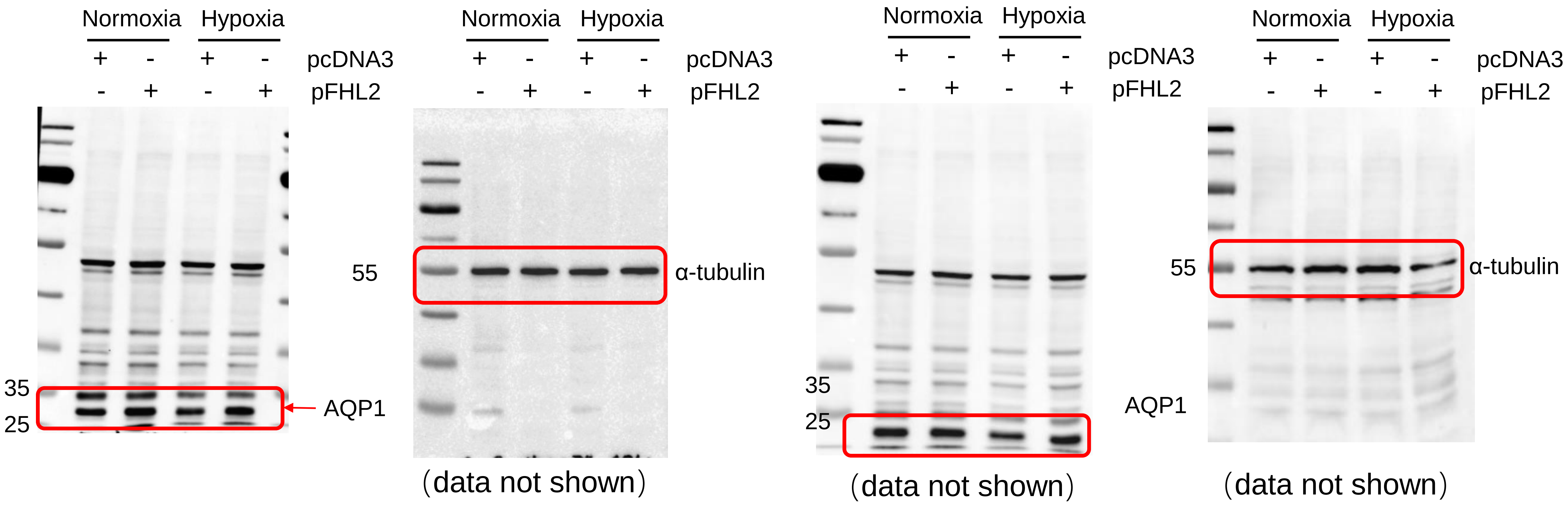

Normoxia
Hypoxia
+ - + - pcDNA3
- + - + pFHL2
35
25
AQP1
Normoxia
Hypoxia
+ - + - pcDNA3
- + - + pFHL2
55
α-tubulin
(data not shown)
Normoxia
Hypoxia
+ - + - pcDNA3
- + - + pFHL2
35
25
AQP1
(data not shown)
Normoxia
Hypoxia
+ - + - pcDNA3
- + - + pFHL2
55
α-tubulin
(data not shown)

Figure 6j

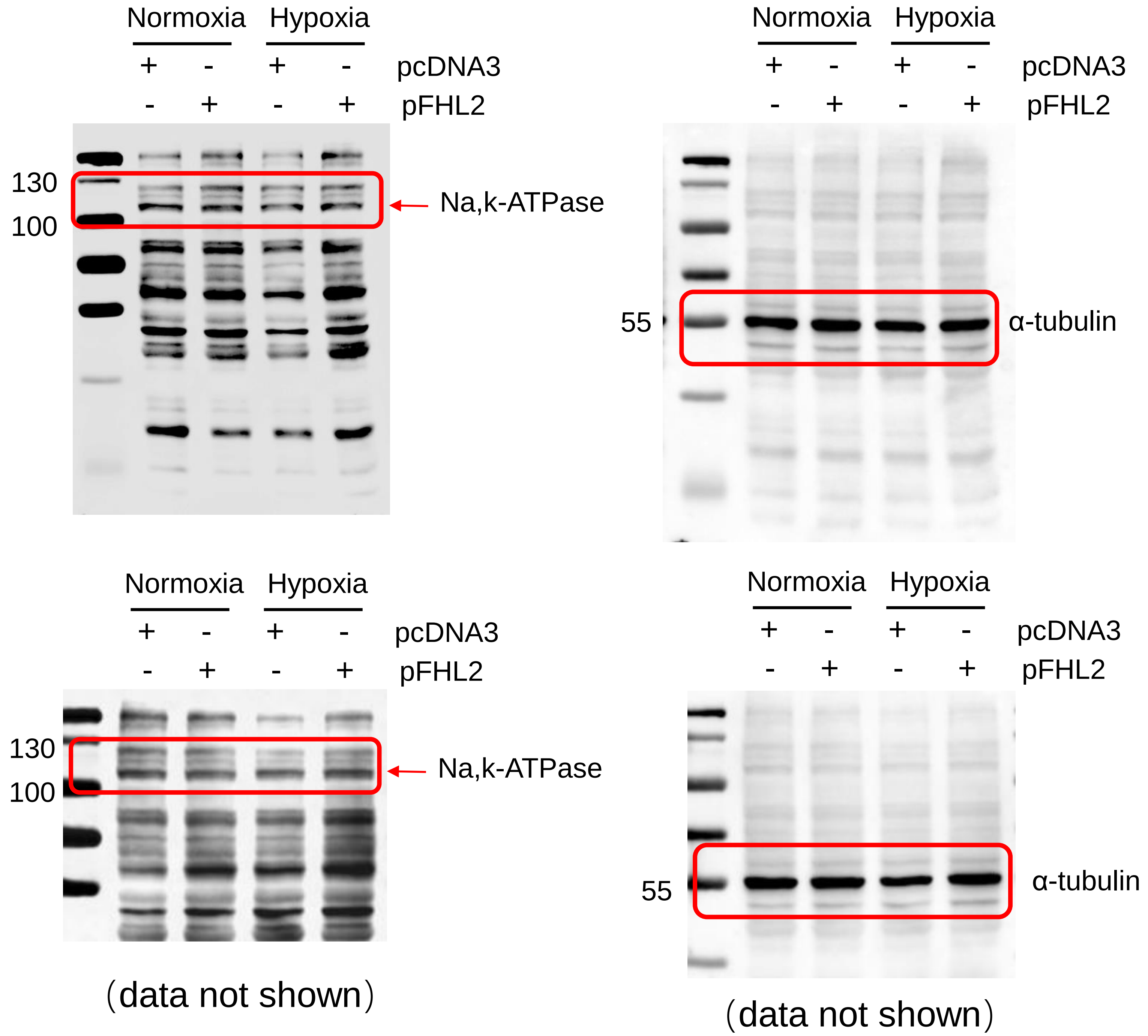

Normoxia
Hypoxia
+ - + - pcDNA3
- + - + pFHL2
130
100
Na,k-ATPase
Normoxia
Hypoxia
+ - + - pcDNA3
- + - + pFHL2
55
α-tubulin
Normoxia
Hypoxia
+ - + - pcDNA3
- + - + pFHL2
130
100
Na,k-ATPase
(data not shown)
Normoxia
Hypoxia
+ - + - pcDNA3
- + - + pFHL2
55
α-tubulin
(data not shown)

Figure 6k

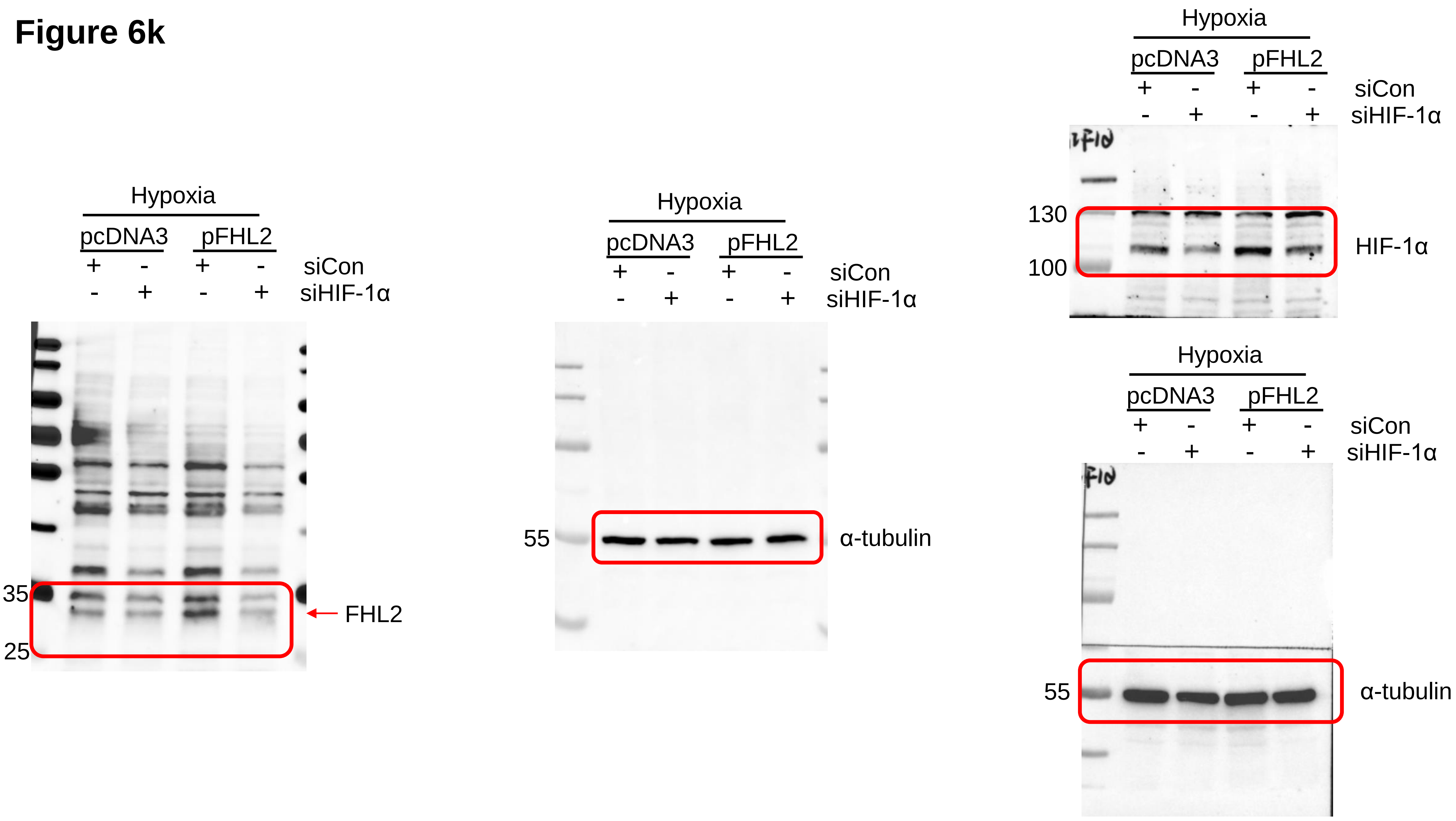

(data not shown)

Figure 6k

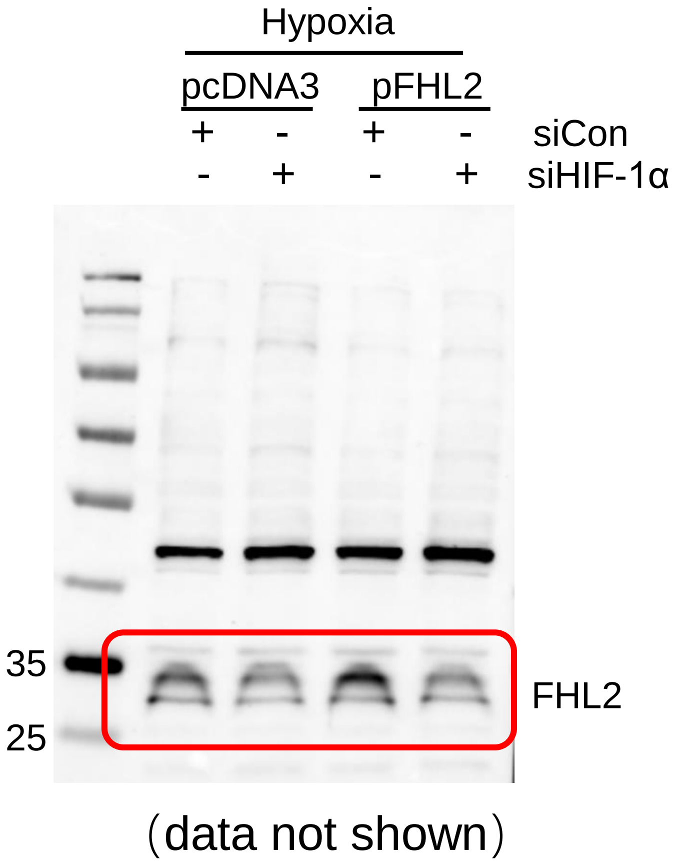
Hypoxia
pcDNA3
pFHL2
+ - + - siCon
- + - + siHIF-1α
35
25
FHL2
(data not shown)

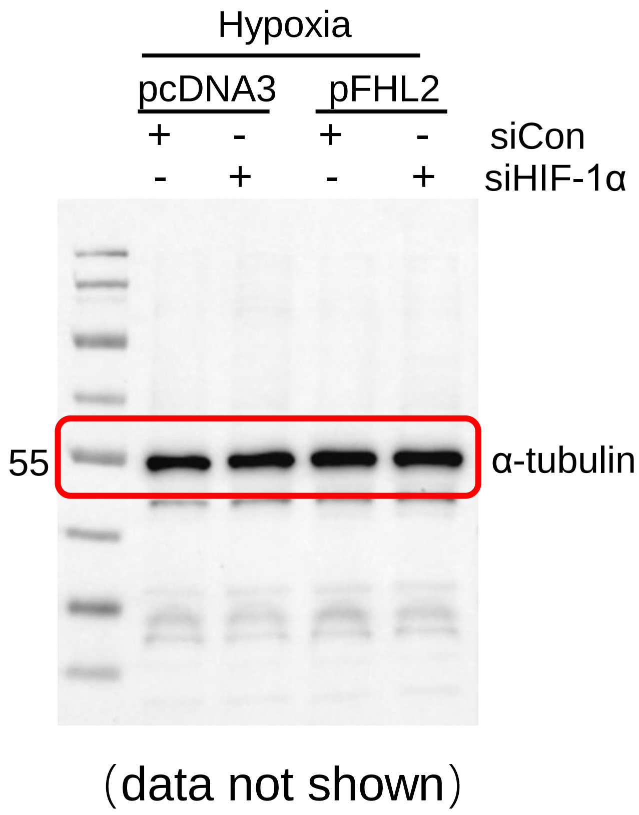
Hypoxia
pcDNA3
pFHL2
+ - + - siCon
- + - + siHIF-1α
55
α-tubulin
(data not shown)

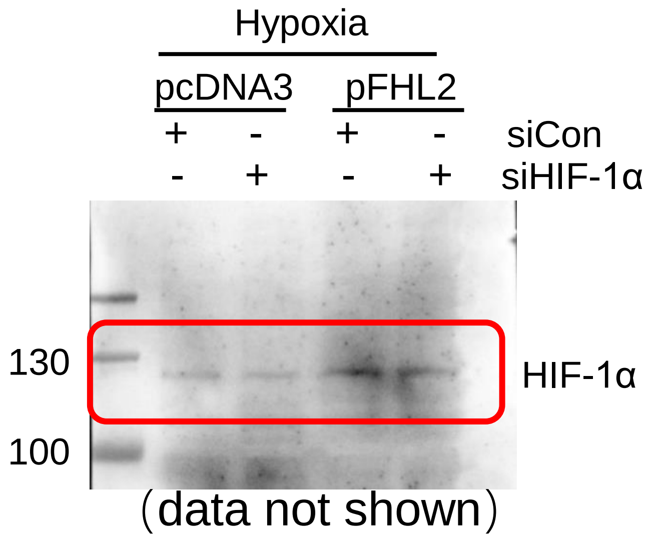
Hypoxia
pcDNA3
pFHL2
+ - + - siCon
- + - + siHIF-1α
130
100
HIF-1α
(data not shown)

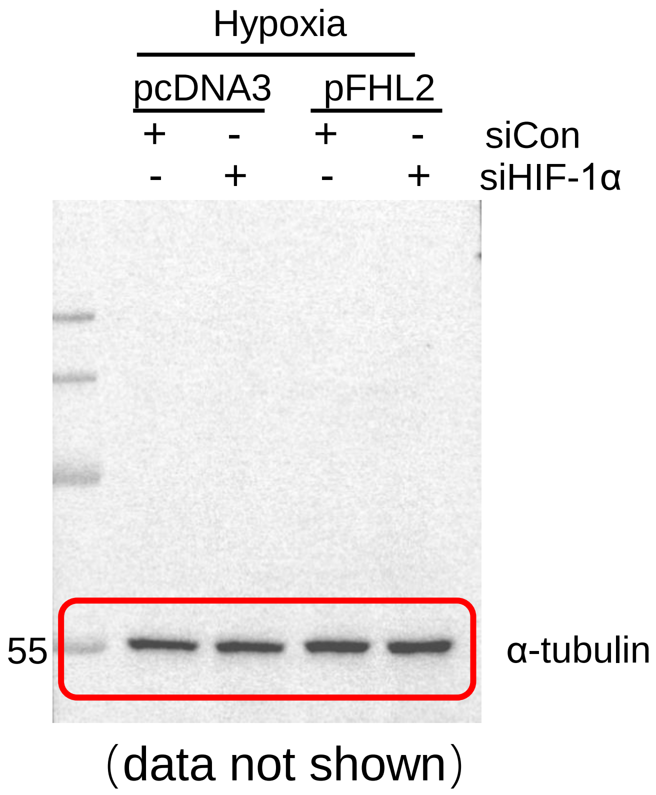
Hypoxia
pcDNA3
pFHL2
+ - + - siCon
- + - + siHIF-1α
55
α-tubulin
(data not shown)

Figure 6k

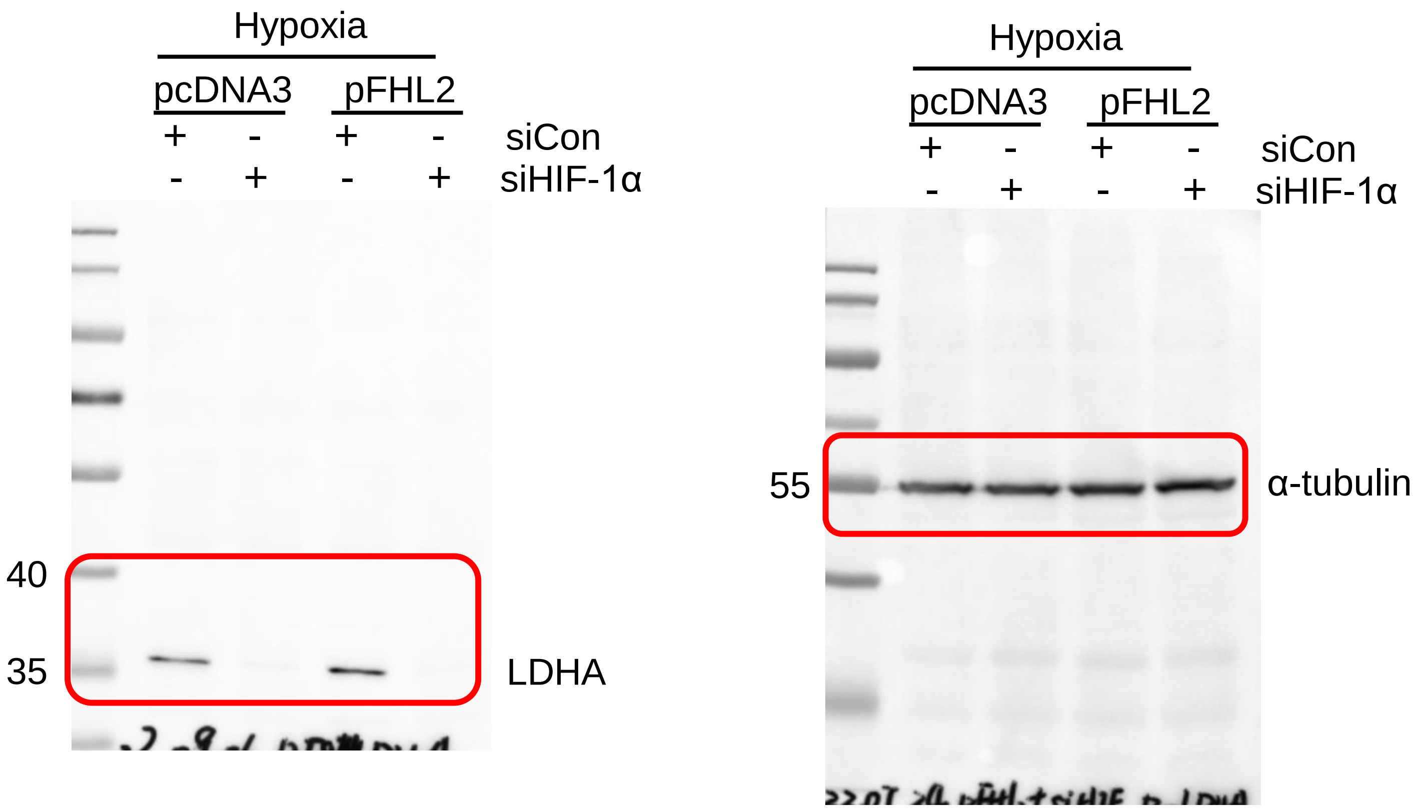

(data not shown)

Figure 6k

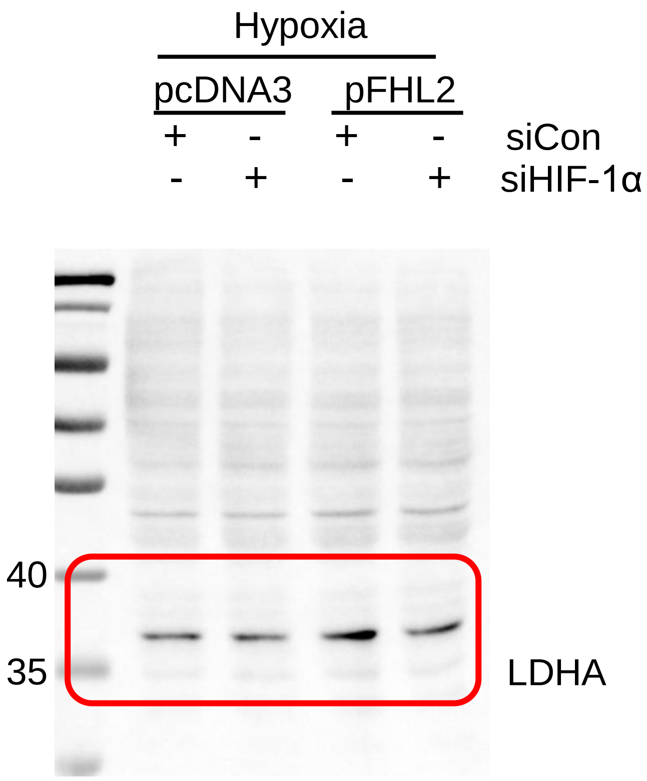

(data not shown)

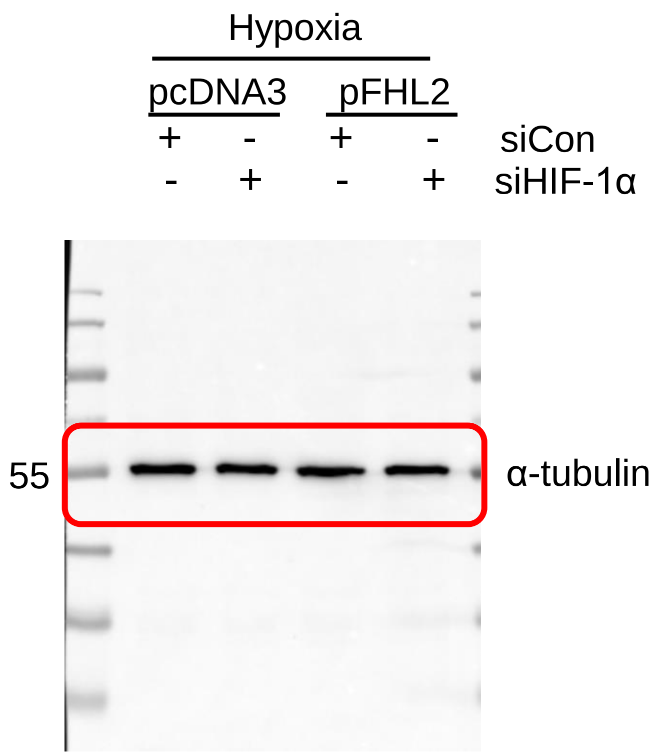

(data not shown)

Figure 6l

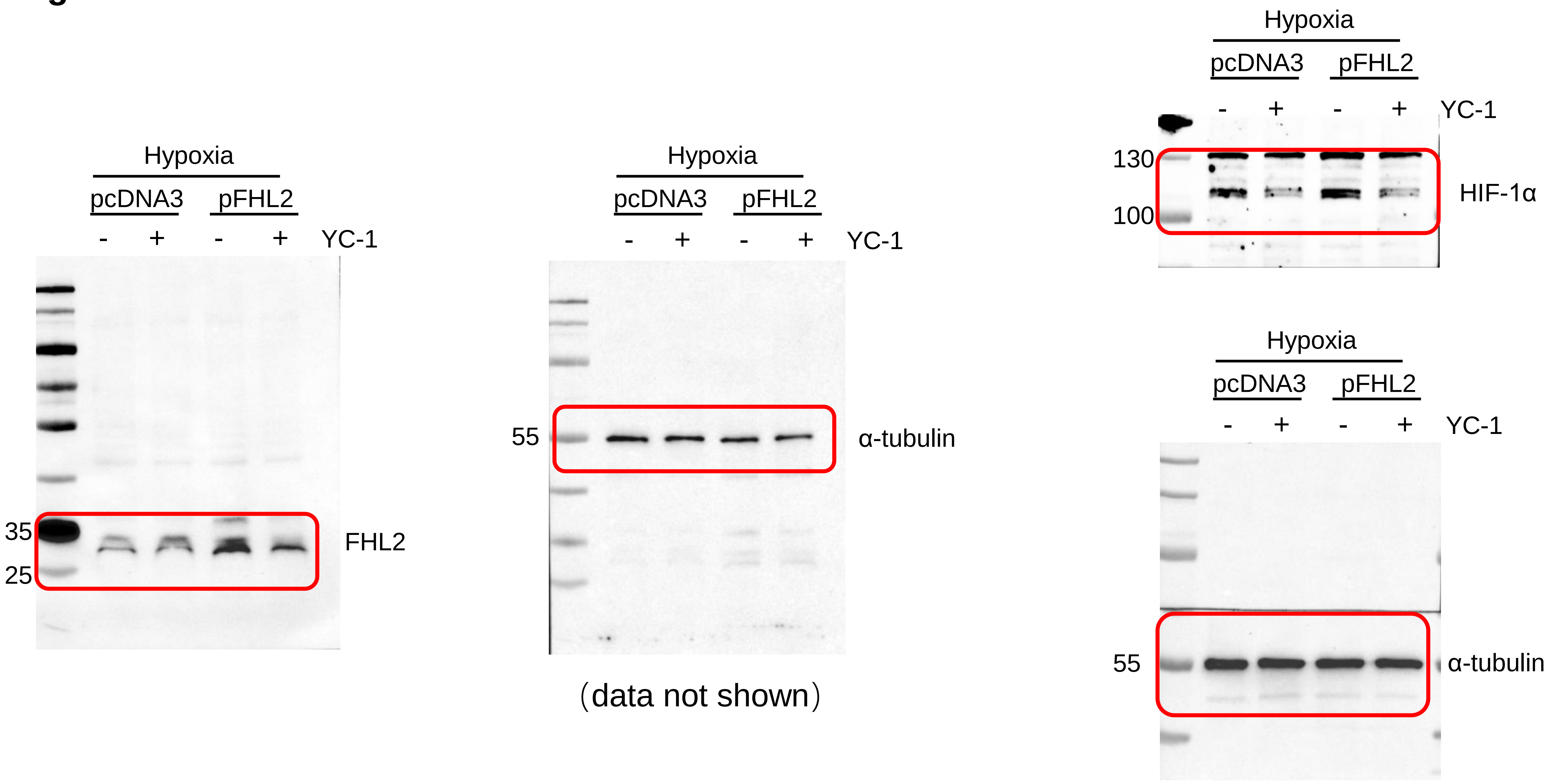
Hypoxia
pcDNA3
pFHL2
- + - + YC-1
35
25
FHL2
Hypoxia
pcDNA3
pFHL2
- + - + YC-1
55
α-tubulin
(data not shown)
Hypoxia
pcDNA3
pFHL2
- + - + YC-1
130
100
HIF-1α
Hypoxia
pcDNA3
pFHL2
- + - + YC-1
55
α-tubulin

Figure 6l

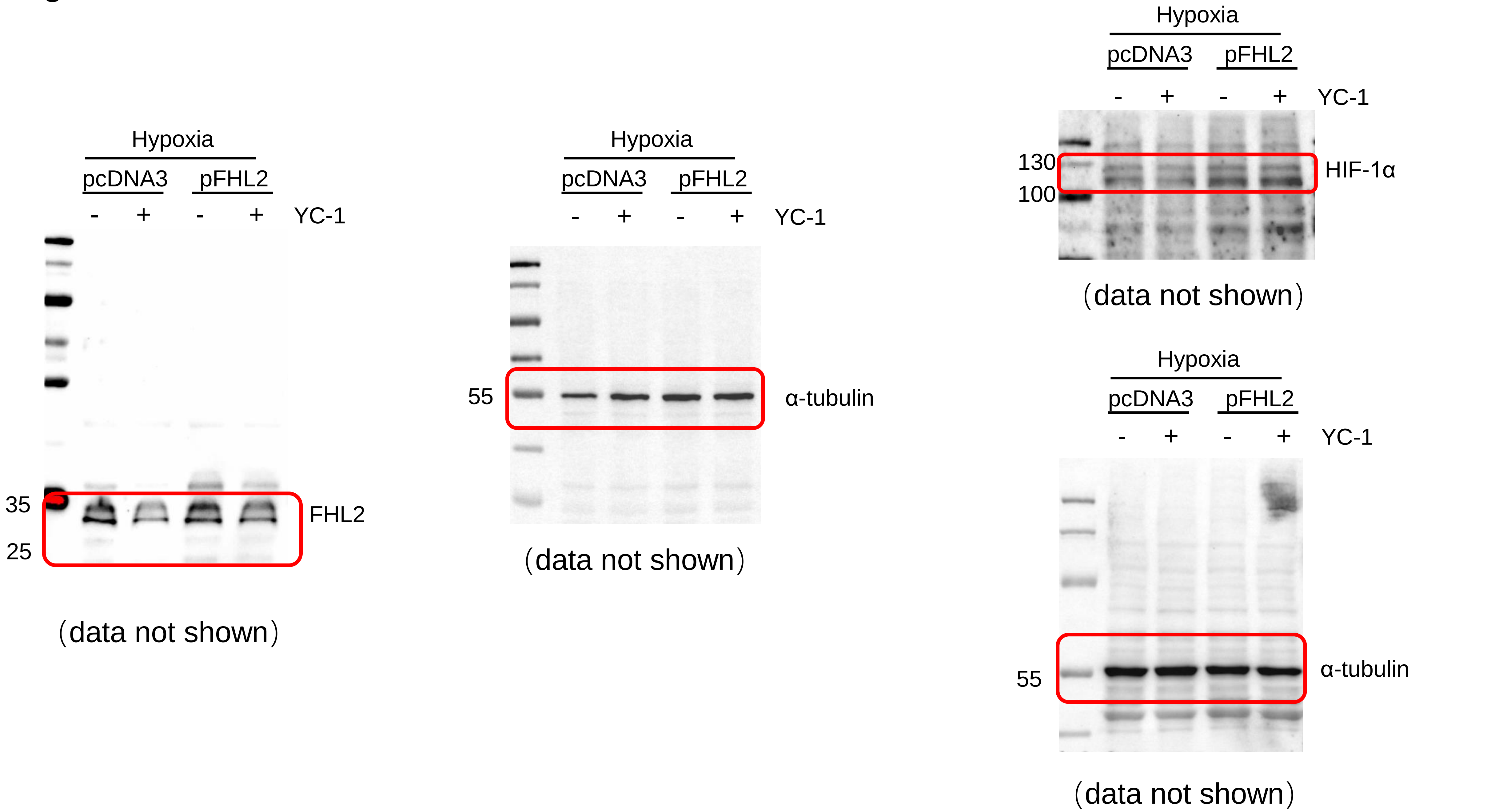
Hypoxia
pcDNA3
pFHL2
- + - + YC-1
35
25
FHL2
(data not shown)
Hypoxia
pcDNA3
pFHL2
- + - + YC-1
55
α-tubulin
(data not shown)
Hypoxia
pcDNA3
pFHL2
- + - + YC-1
130
100
HIF-1α
(data not shown)
Hypoxia
pcDNA3
pFHL2
- + - + YC-1
55
α-tubulin
(data not shown)

Figure 6I

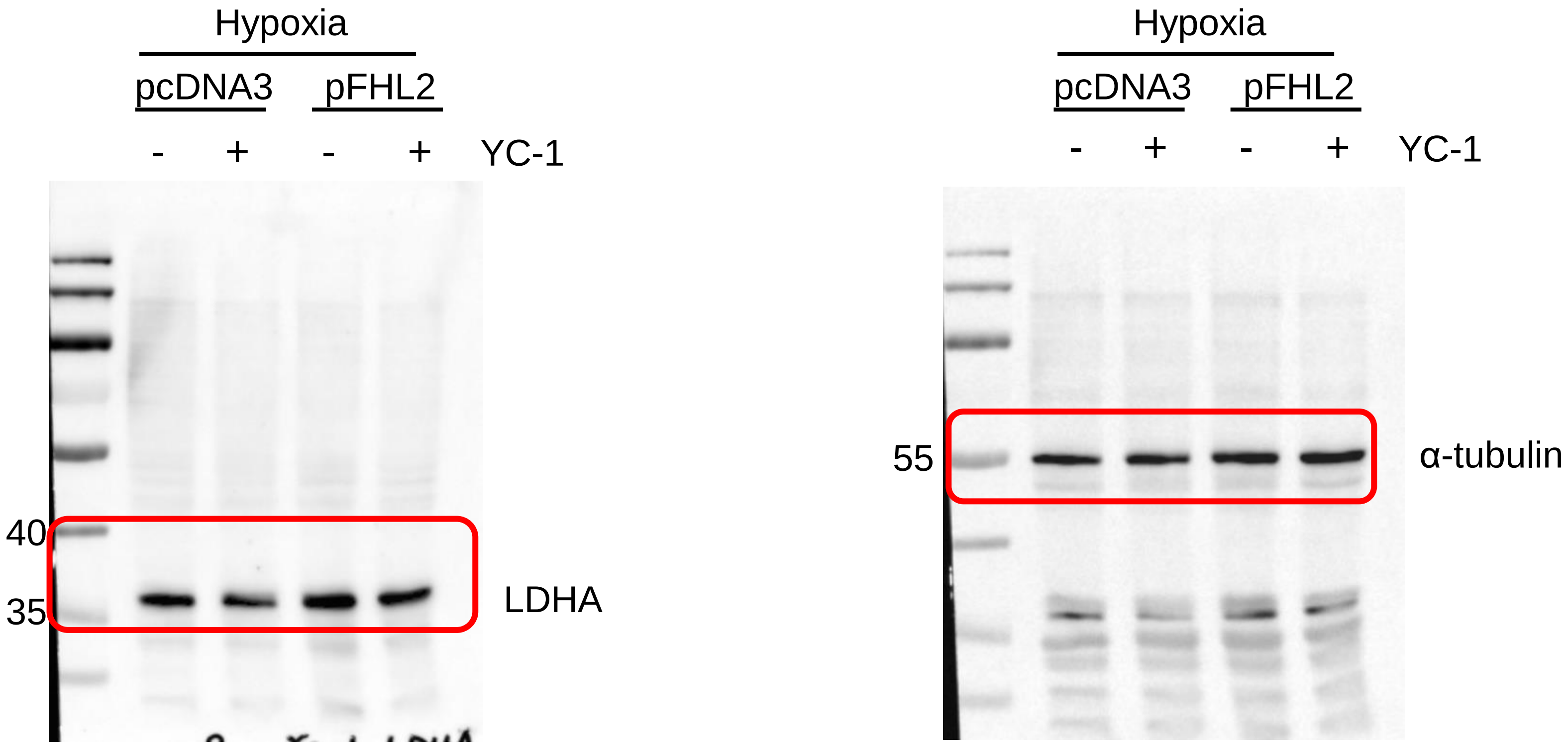

(data not shown)

Figure 6I

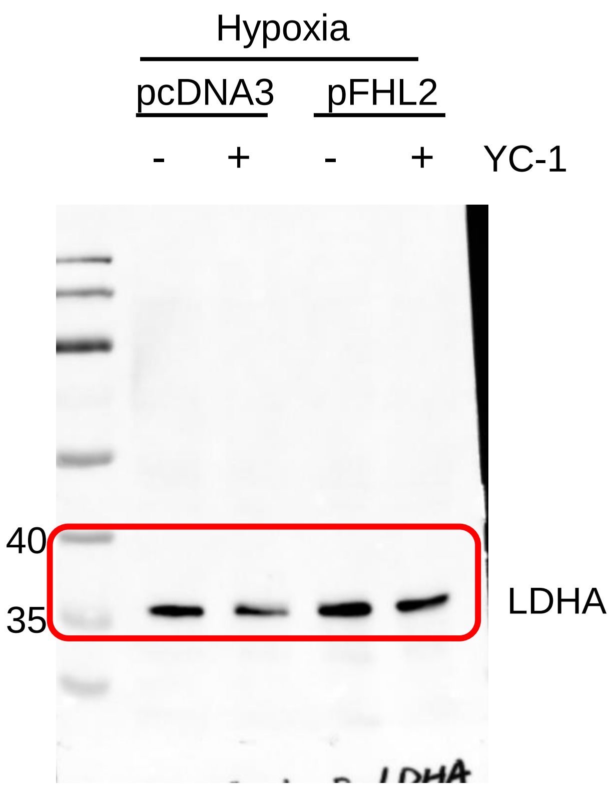

(data not shown)

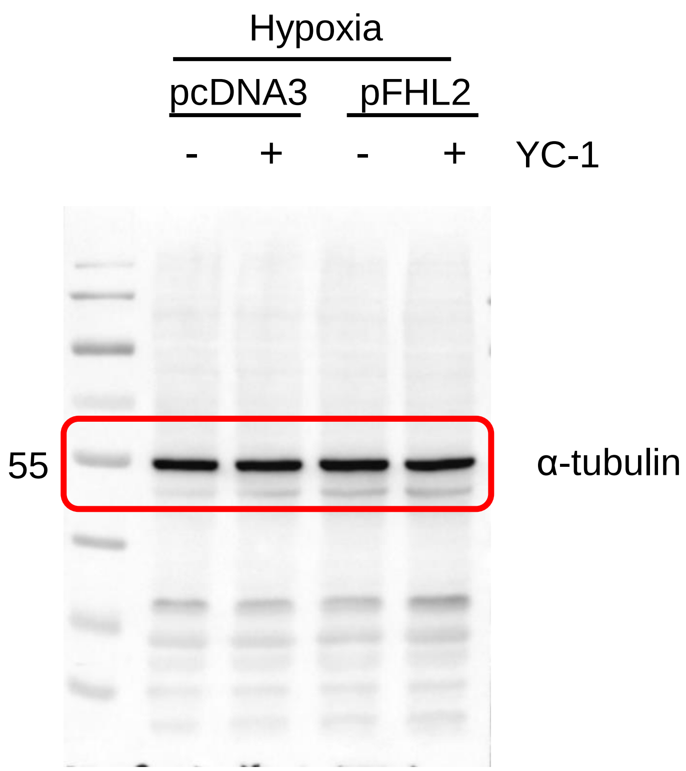

(data not shown)

Figure 6m

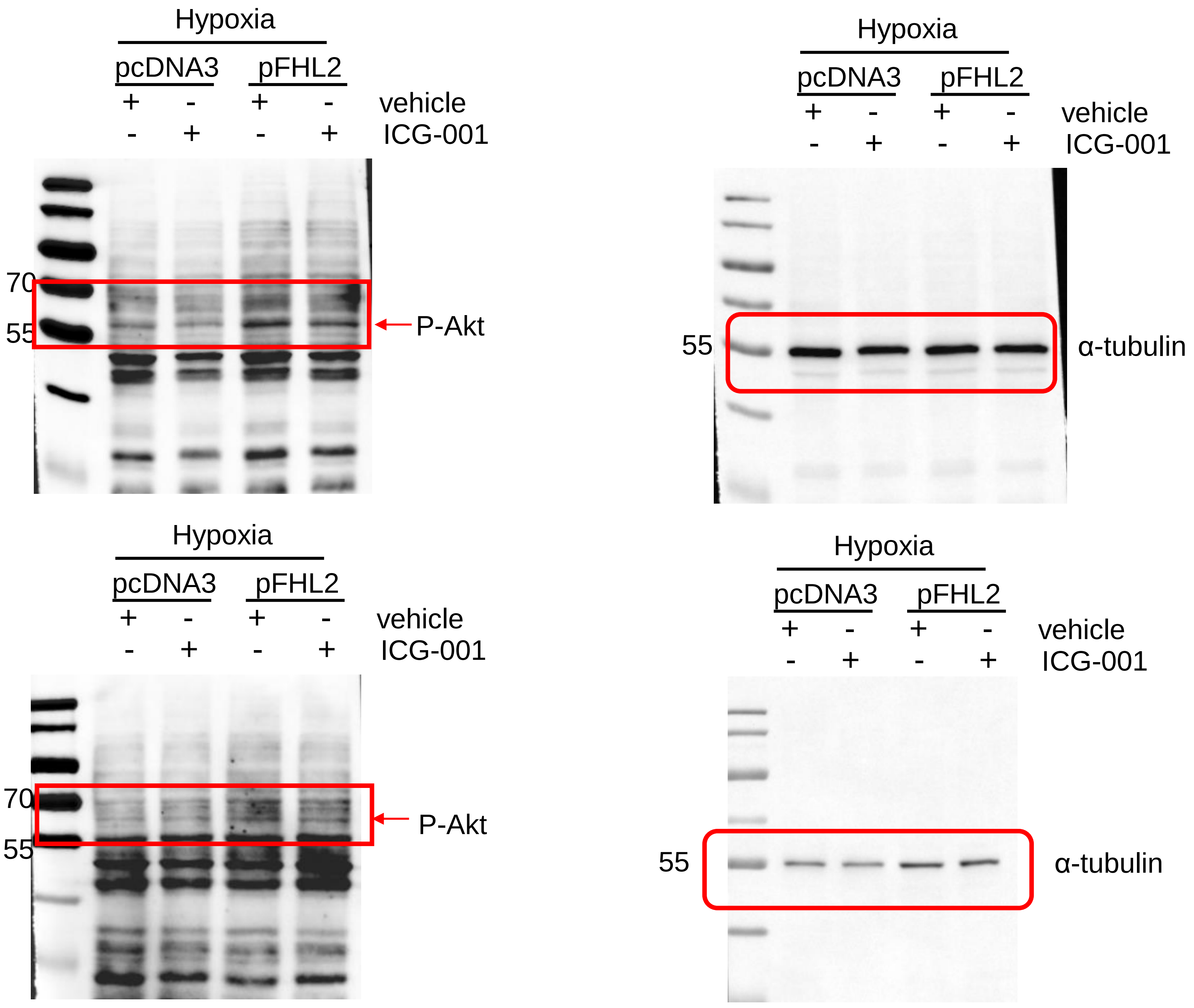

(data not shown)

(data not shown)

Figure 6m

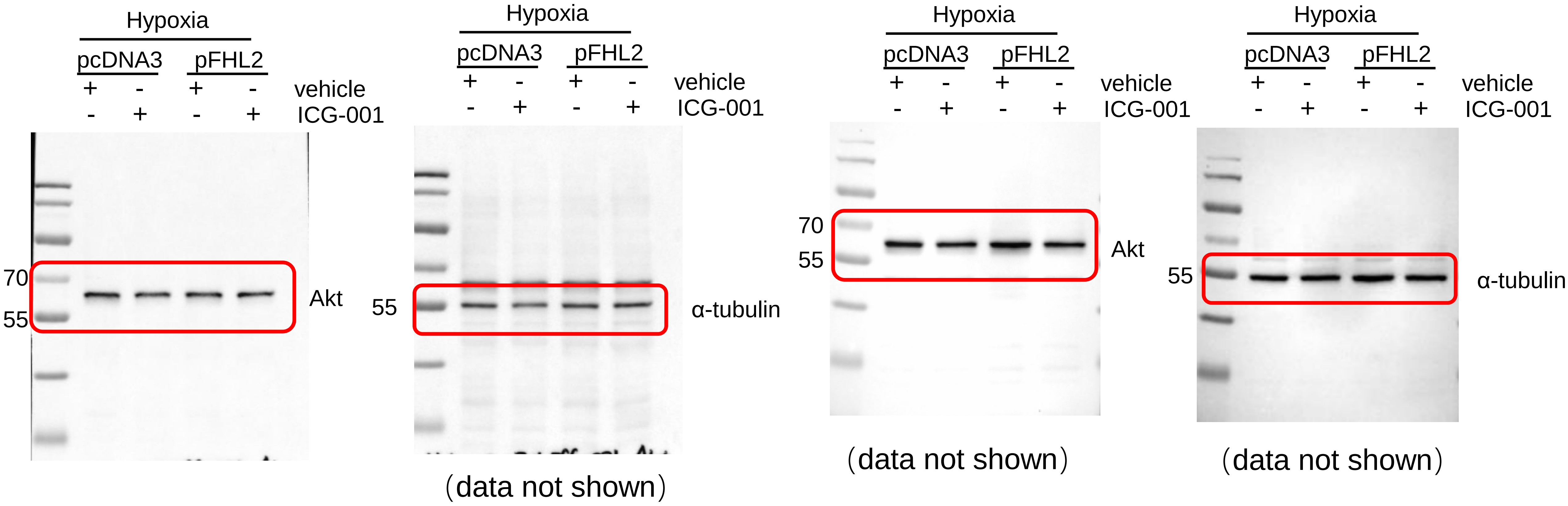
Hypoxia
pcDNA3 pFHL2
+ - + - vehicle
- + - + ICG-001
70
55
Akt
Hypoxia
pcDNA3 pFHL2
+ - + - vehicle
- + - + ICG-001
55
α-tubulin
(data not shown)
Hypoxia
pcDNA3 pFHL2
+ - + - vehicle
- + - + ICG-001
70
55
Akt
(data not shown)
Hypoxia
pcDNA3 pFHL2
+ - + - vehicle
- + - + ICG-001
55
α-tubulin
(data not shown)

Figure 6m

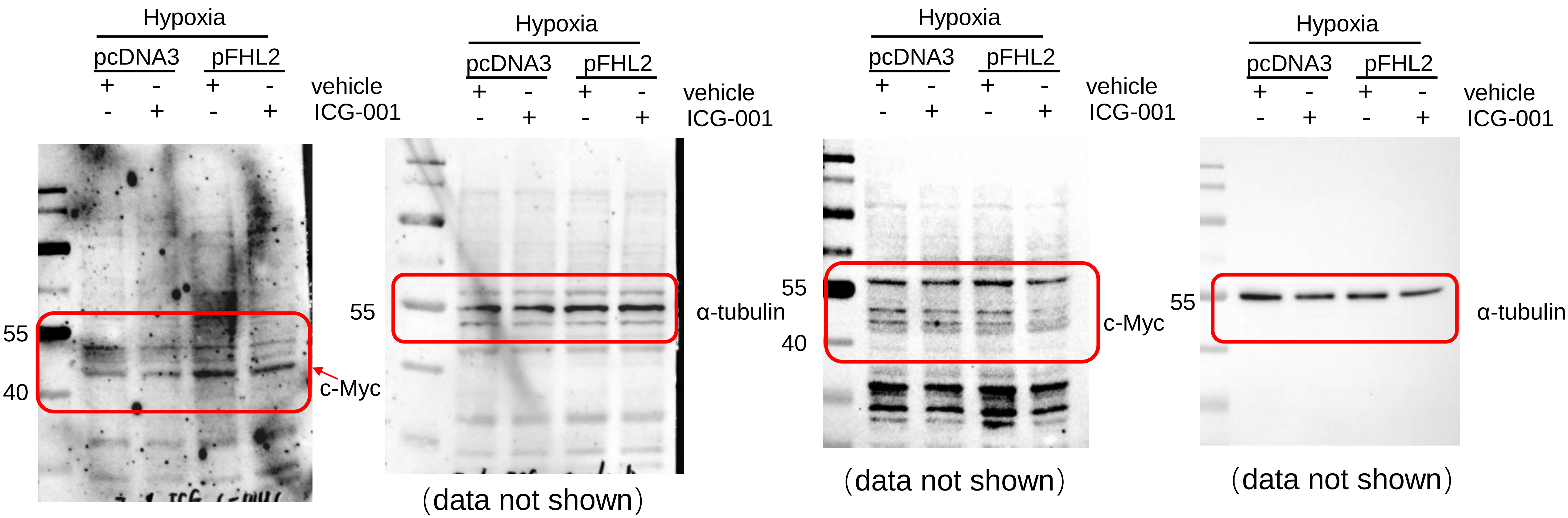
Hypoxia
pcDNA3
pFHL2
+ - + - vehicle
- + - + ICG-001
55
40
c-Myc
Hypoxia
pcDNA3
pFHL2
+ - + - vehicle
- + - + ICG-001
55
α-tubulin
(data not shown)
Hypoxia
pcDNA3
pFHL2
+ - + - vehicle
- + - + ICG-001
55
40
c-Myc
(data not shown)
Hypoxia
pcDNA3
pFHL2
+ - + - vehicle
- + - + ICG-001
55
α-tubulin
(data not shown)

Figure 6m

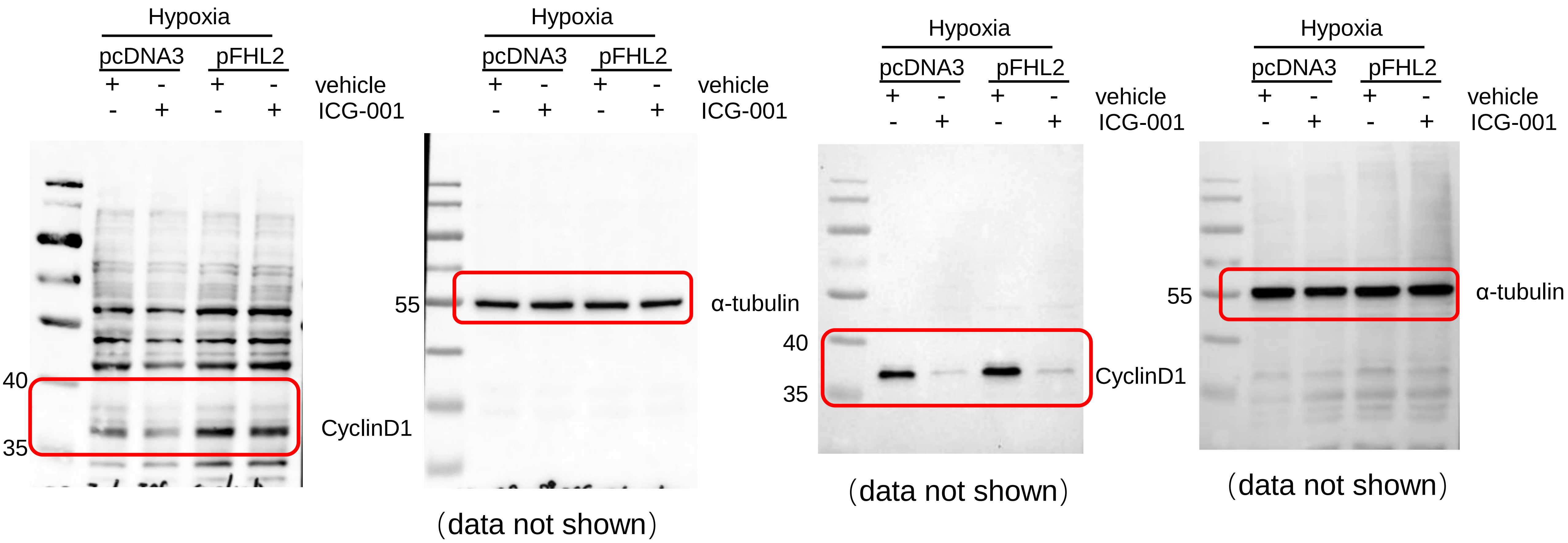

Hypoxia
pcDNA3
pFHL2
+ - + - vehicle
- + - + ICG-001
40
35
CyclinD1
Hypoxia
pcDNA3
pFHL2
+ - + - vehicle
- + - + ICG-001
55
α-tubulin
(data not shown)
Hypoxia
pcDNA3
pFHL2
+ - + - vehicle
- + - + ICG-001
40
35
CyclinD1
(data not shown)
Hypoxia
pcDNA3
pFHL2
+ - + - vehicle
- + - + ICG-001
55
α-tubulin
(data not shown)

Figure 6m

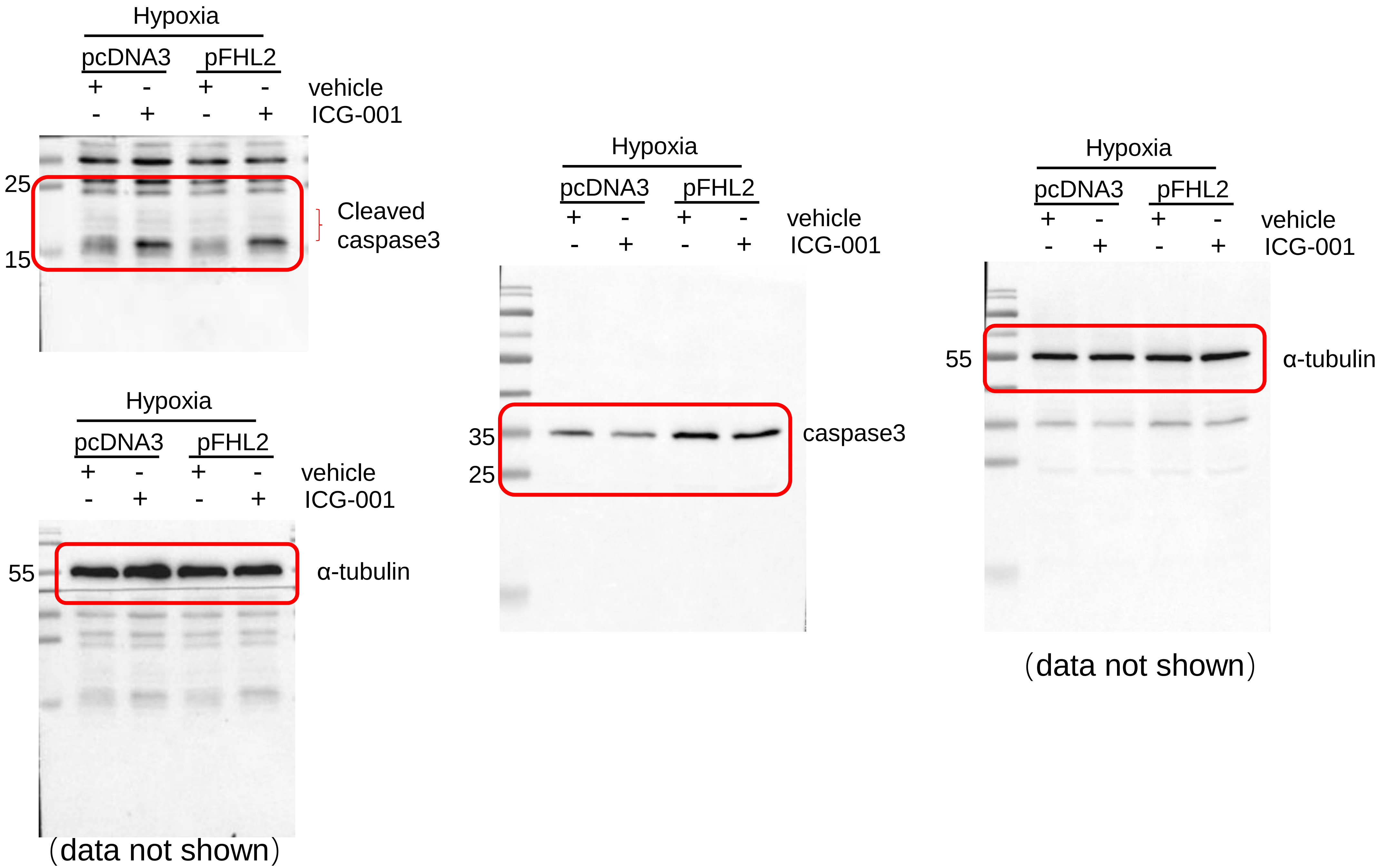
Hypoxia
pcDNA3
pFHL2
+ - + - vehicle
- + - + ICG-001
25
15
Cleaved caspase3
Hypoxia
pcDNA3
pFHL2
+ - + - vehicle
- + - + ICG-001
55
α-tubulin
(data not shown)
Hypoxia
pcDNA3
pFHL2
+ - + - vehicle
- + - + ICG-001
35
25
caspase3
Hypoxia
pcDNA3
pFHL2
+ - + - vehicle
- + - + ICG-001
55
α-tubulin
(data not shown)

Figure 6m

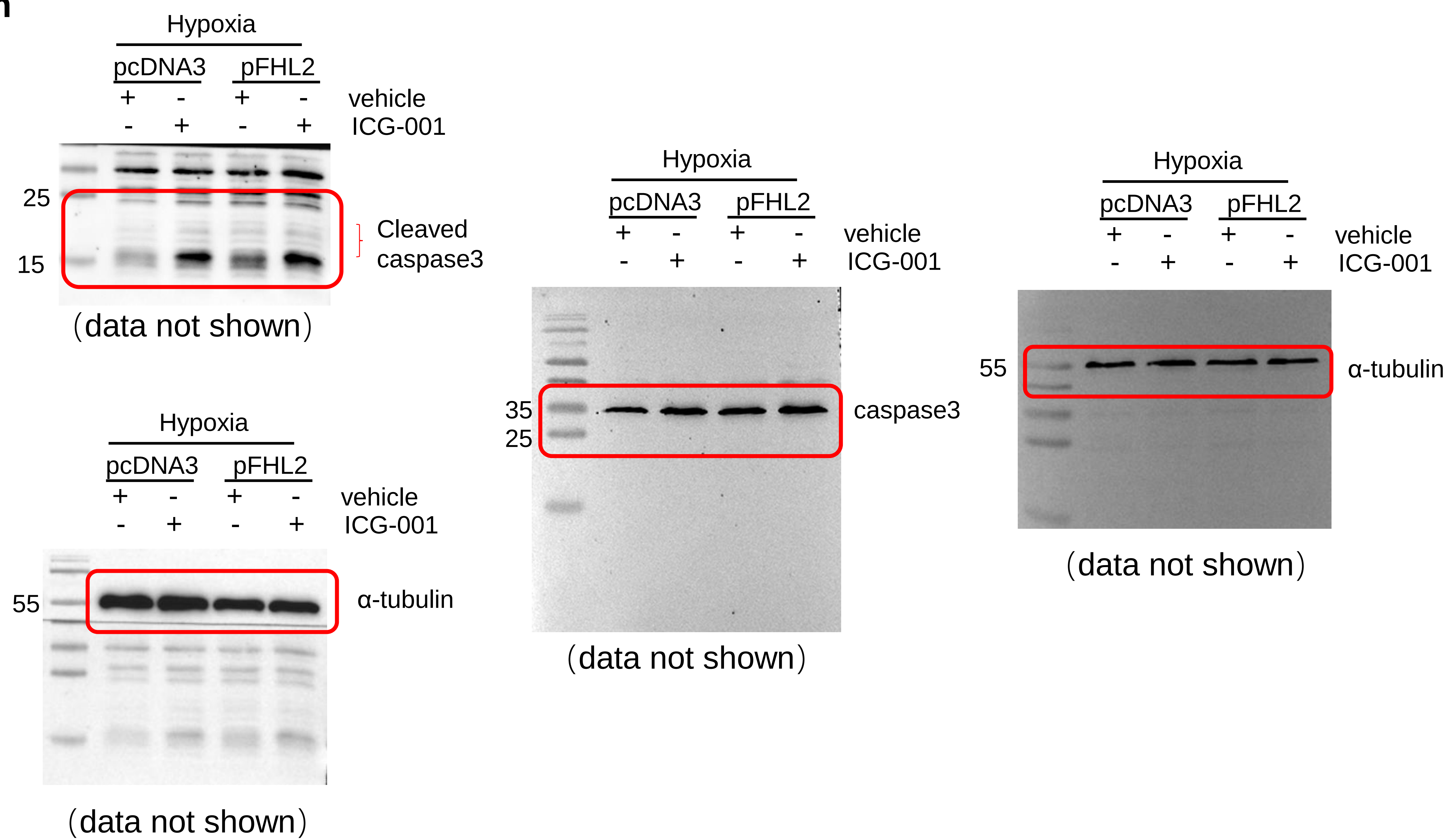
Hypoxia
pcDNA3
pFHL2
vehicle
ICG-001
+ - + -
- + - +
25
15
Cleaved caspase3
(data not shown)
Hypoxia
pcDNA3
pFHL2
vehicle
ICG-001
55
α-tubulin
(data not shown)
Hypoxia
pcDNA3
pFHL2
vehicle
ICG-001
35
25
caspase3
(data not shown)
Hypoxia
pcDNA3
pFHL2
vehicle
ICG-001
55
α-tubulin
(data not shown)

Figure 6n

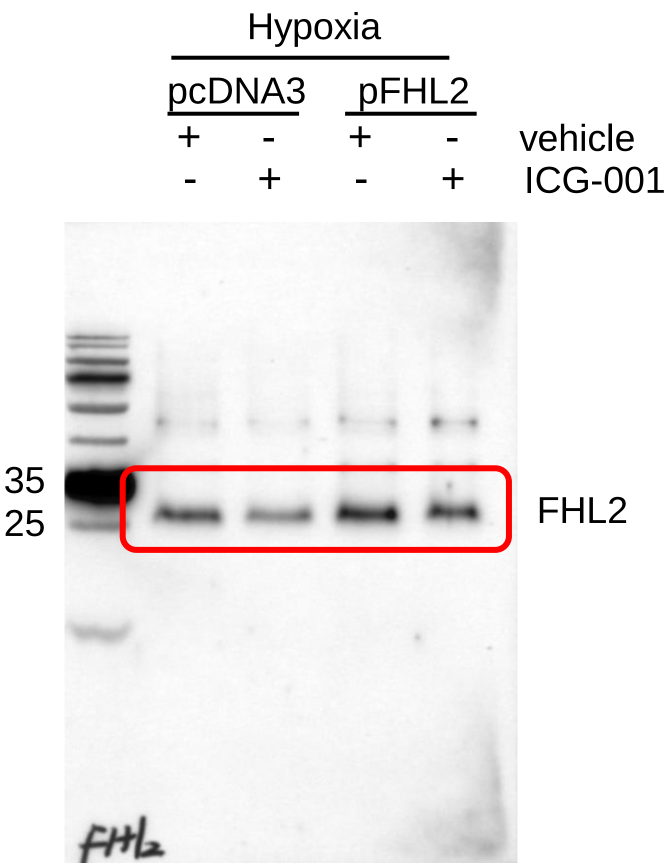

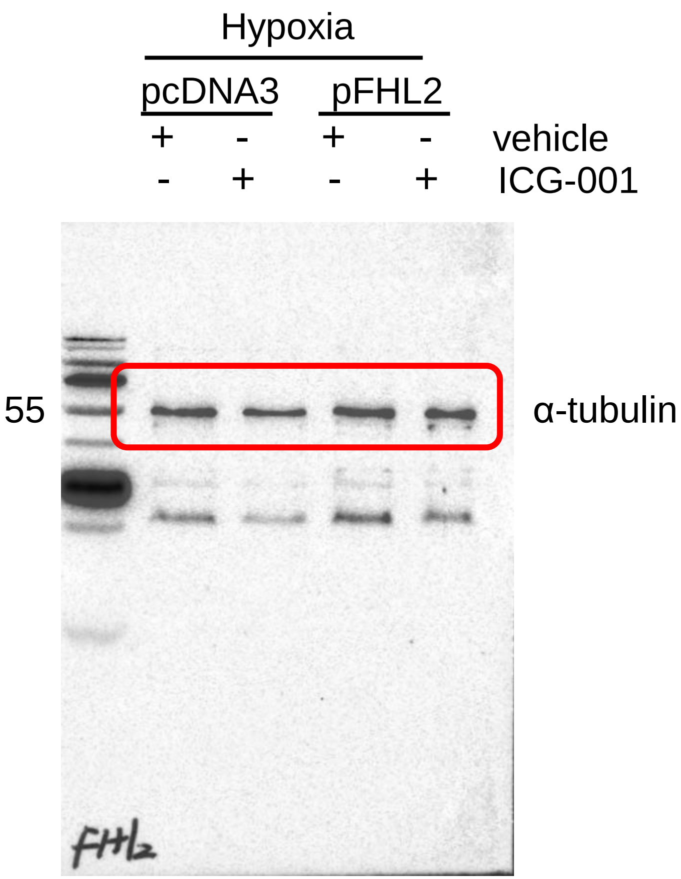

(data not shown)

Figure 6n

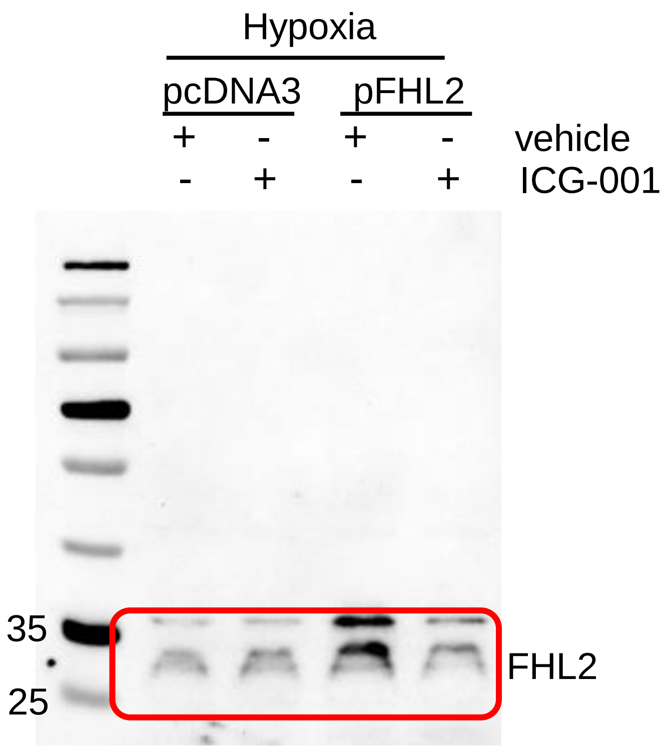

(data not shown)

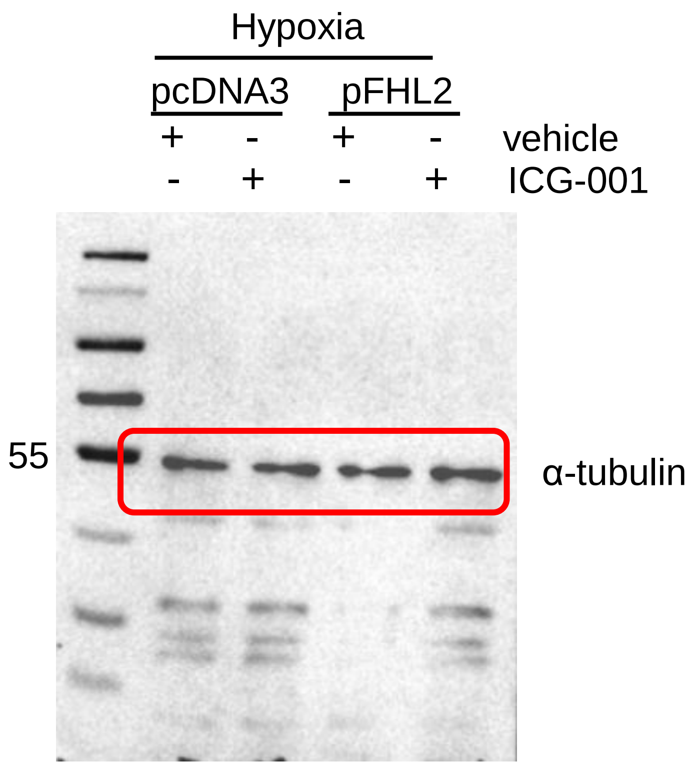

(data not shown)

Figure 6n

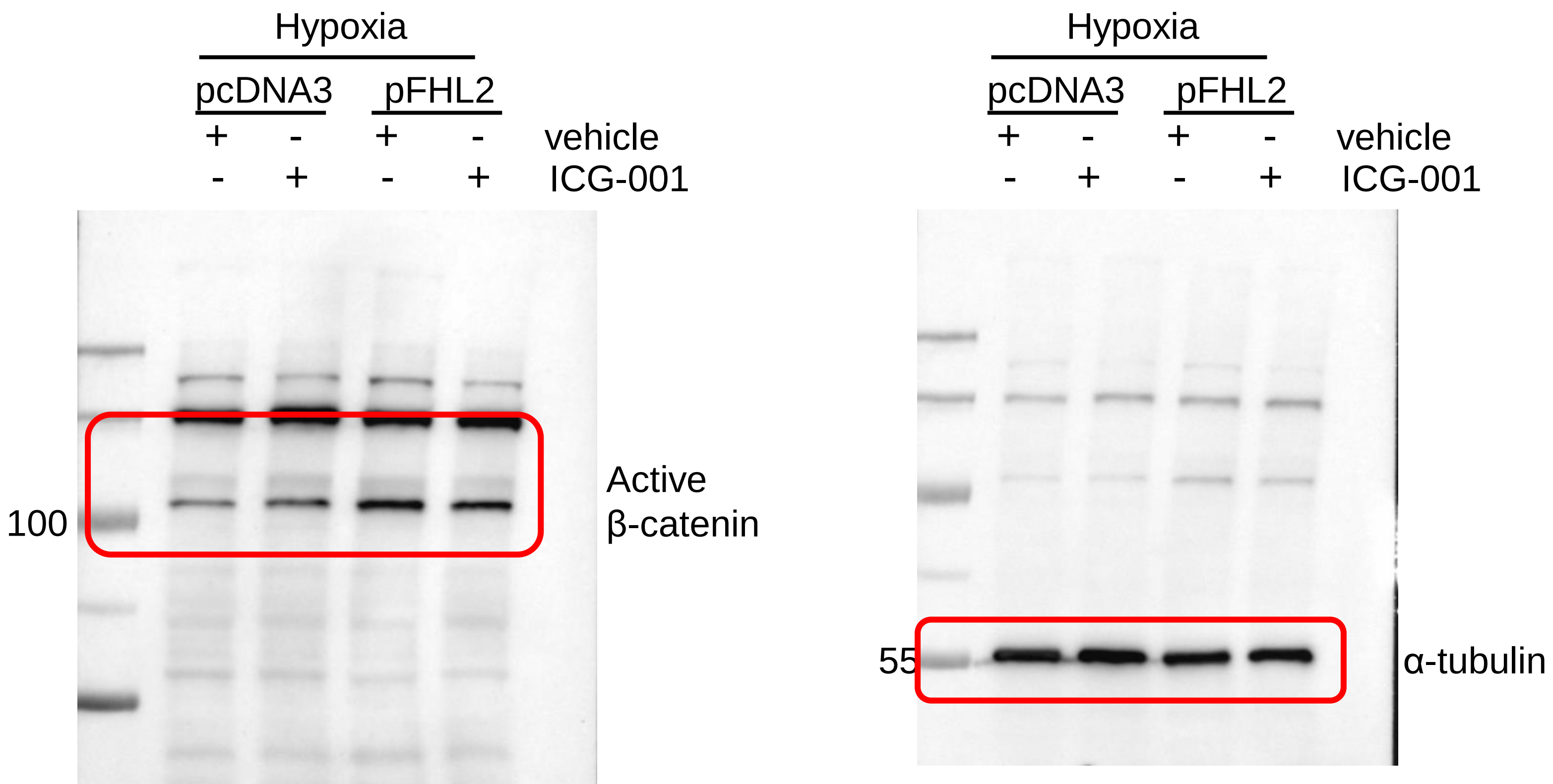

(data not shown)

Figure 6n

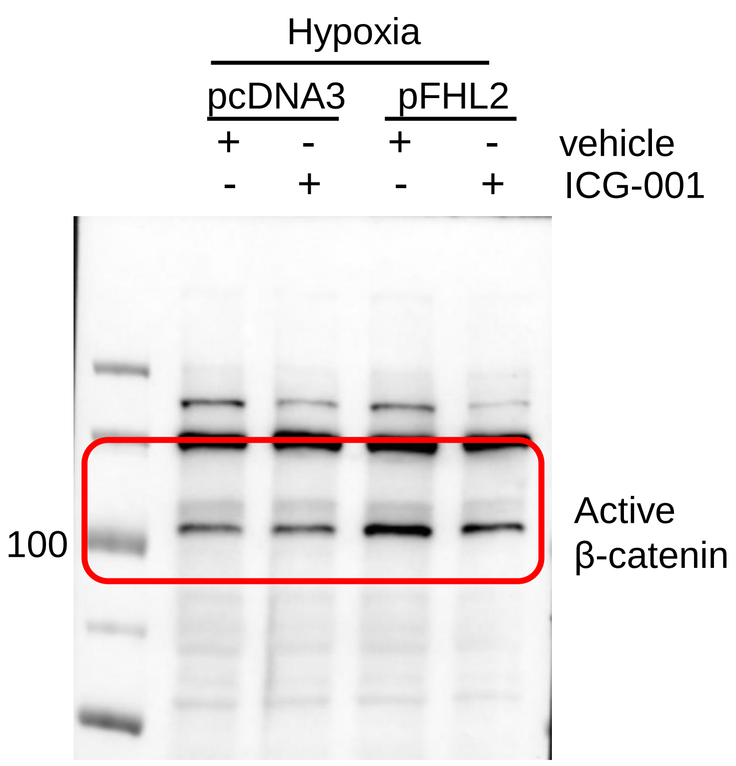

(data not shown)

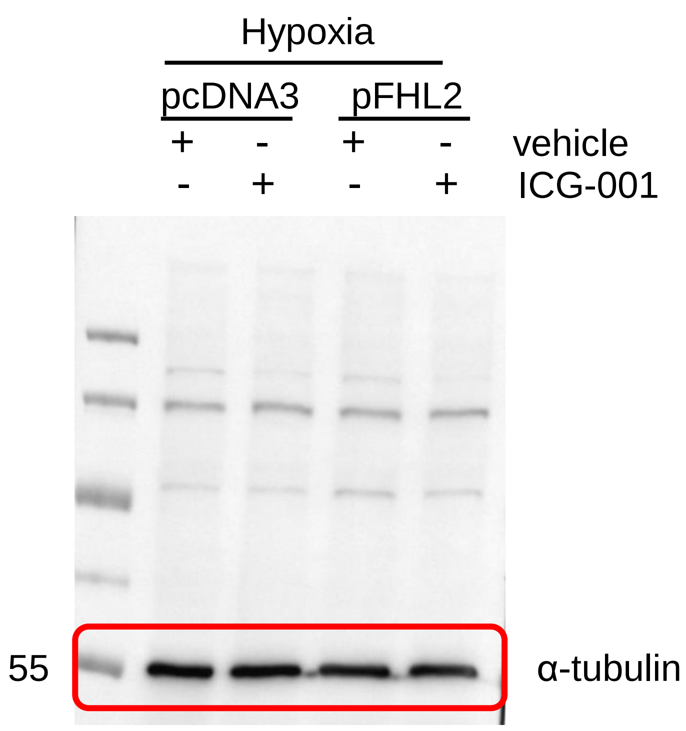

(data not shown)

Figure 6n

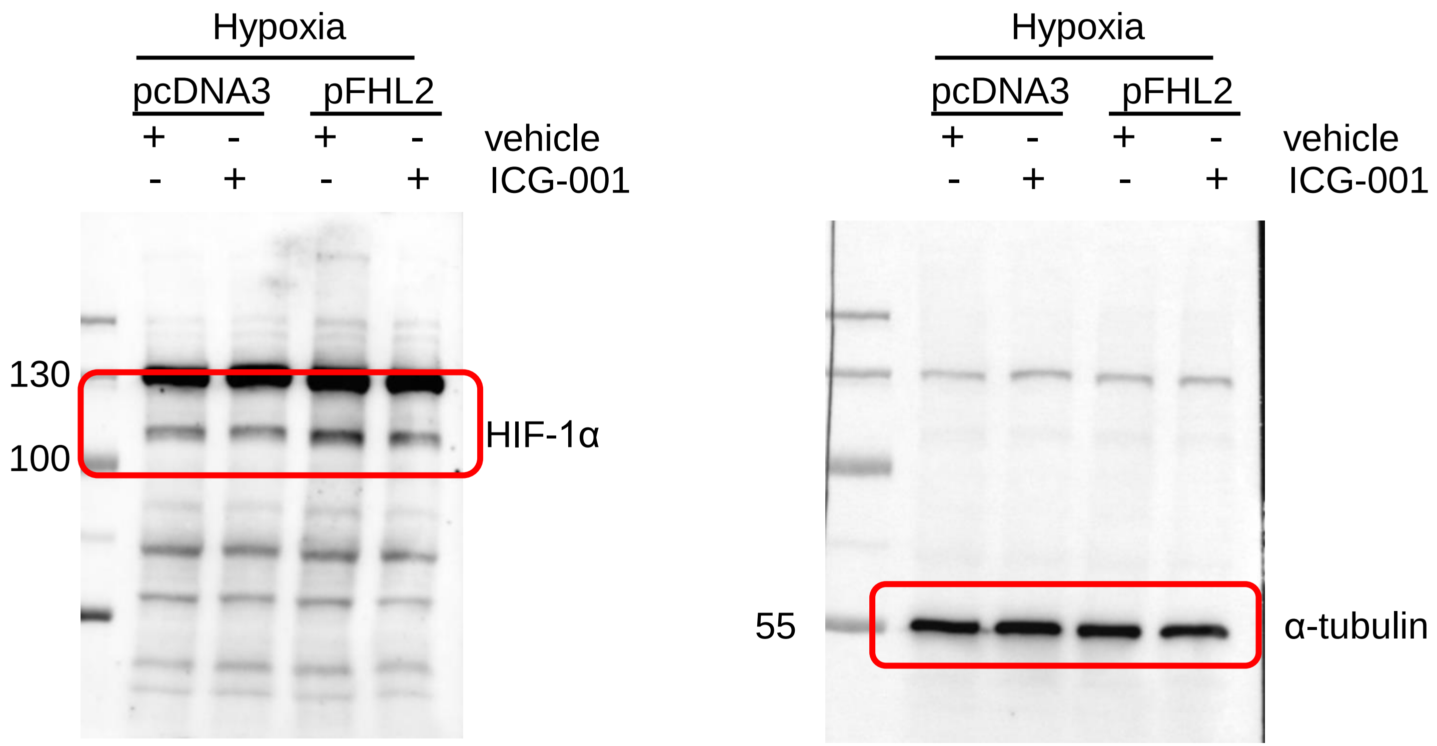

Hypoxia
pcDNA3
pFHL2
+ - + - vehicle
- + - + ICG-001
130
100
HIF-1α
Hypoxia
pcDNA3
pFHL2
+ - + - vehicle
- + - + ICG-001
55
α-tubulin

Figure 6n

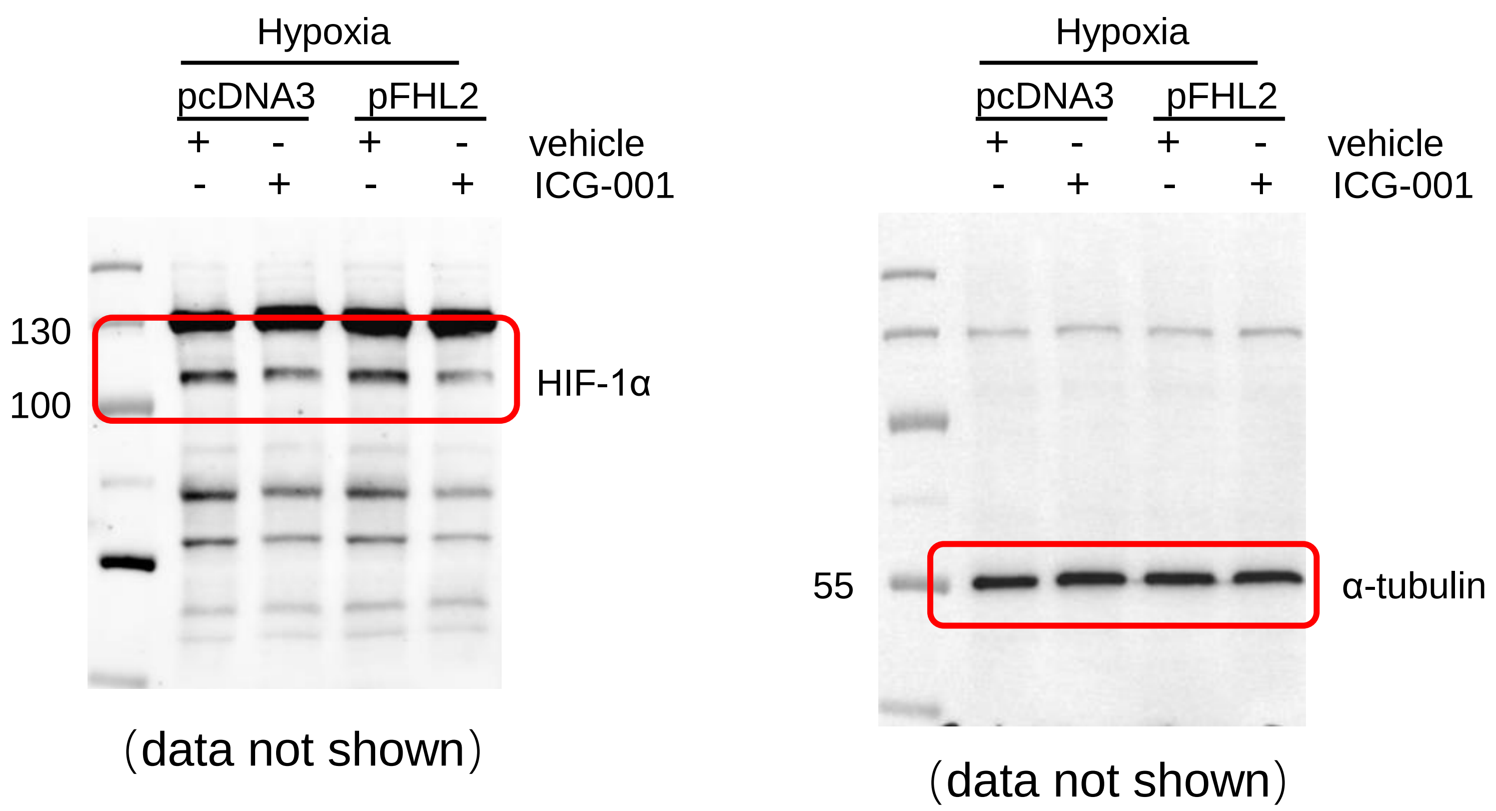

Hypoxia
pcDNA3
pFHL2
+ - + - vehicle
- + - + ICG-001
130
100
HIF-1α
(data not shown)
Hypoxia
pcDNA3
pFHL2
+ - + - vehicle
- + - + ICG-001
55
α-tubulin
(data not shown)

Figure 7a

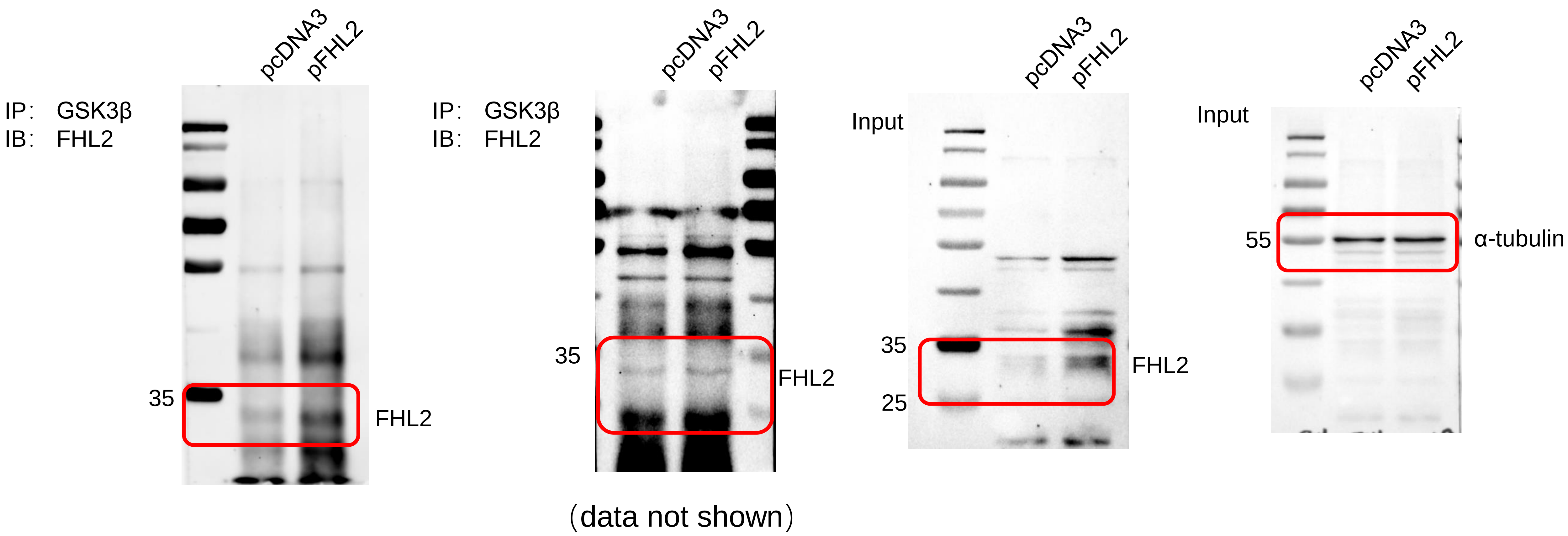

IP: GSK3β
IB: FHL2
pcDNA3
pFHL2
35
FHL2
IP: GSK3β
IB: FHL2
pcDNA3
pFHL2
35
FHL2
(data not shown)
Input
pcDNA3
pFHL2
35
25
FHL2
Input
pcDNA3
pFHL2
55
α-tubulin

Figure 7a

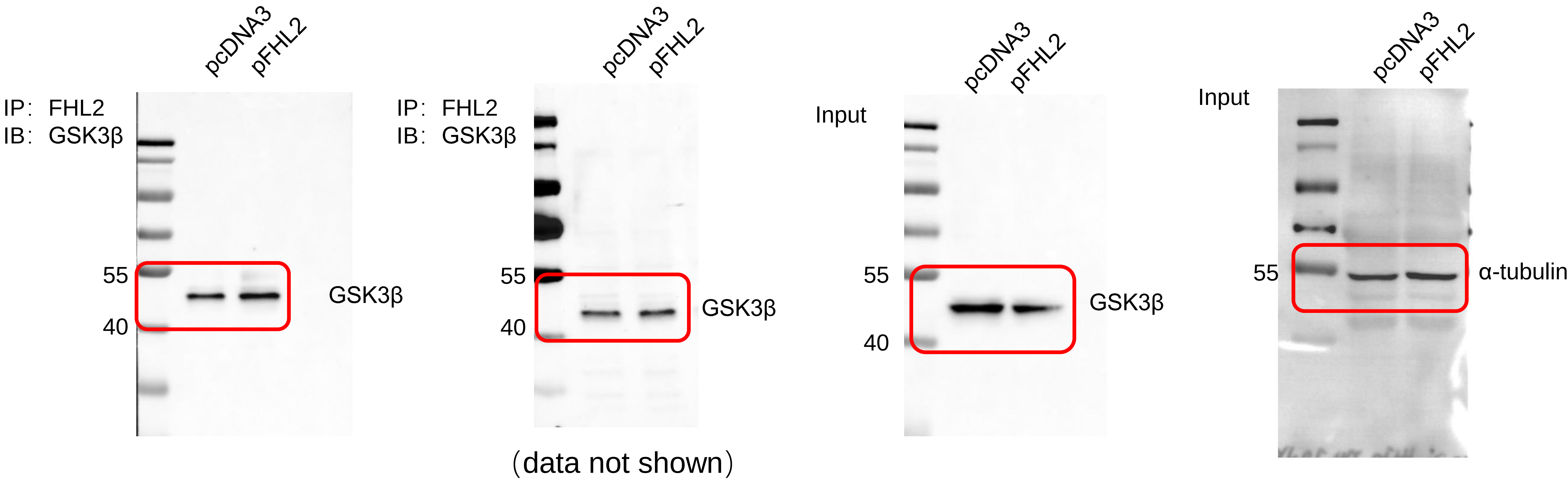
pcDNA3
pFHL2
IP: FHL2
IB: GSK3β
55
40
GSK3β
pcDNA3
pFHL2
IP: FHL2
IB: GSK3β
55
40
GSK3β
(data not shown)
pcDNA3
pFHL2
Input
55
40
GSK3β
pcDNA3
pFHL2
Input
55
α-tubulin

Figure 7d

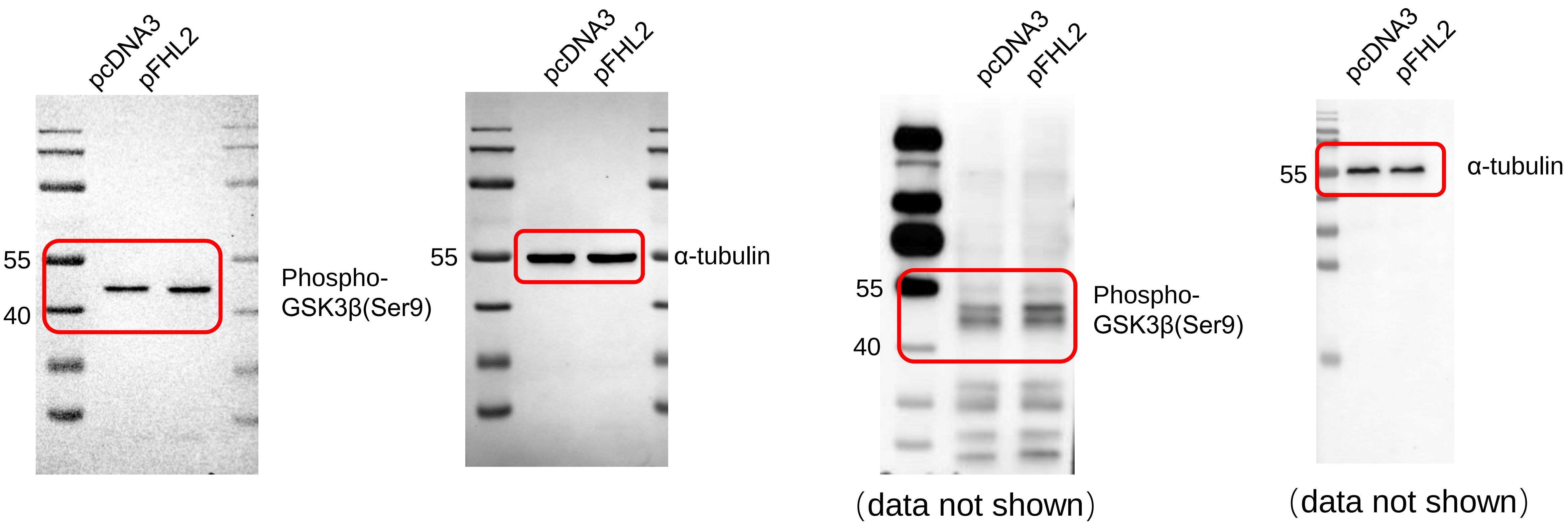
pcDNA3
pFHL2
55
40
Phospho-
GSK3β(Ser9)
pcDNA3
pFHL2
55
α-tubulin
pcDNA3
pFHL2
55
40
Phospho-
GSK3β(Ser9)
(data not shown)
pcDNA3
pFHL2
55
α-tubulin
(data not shown)

Figure 7d

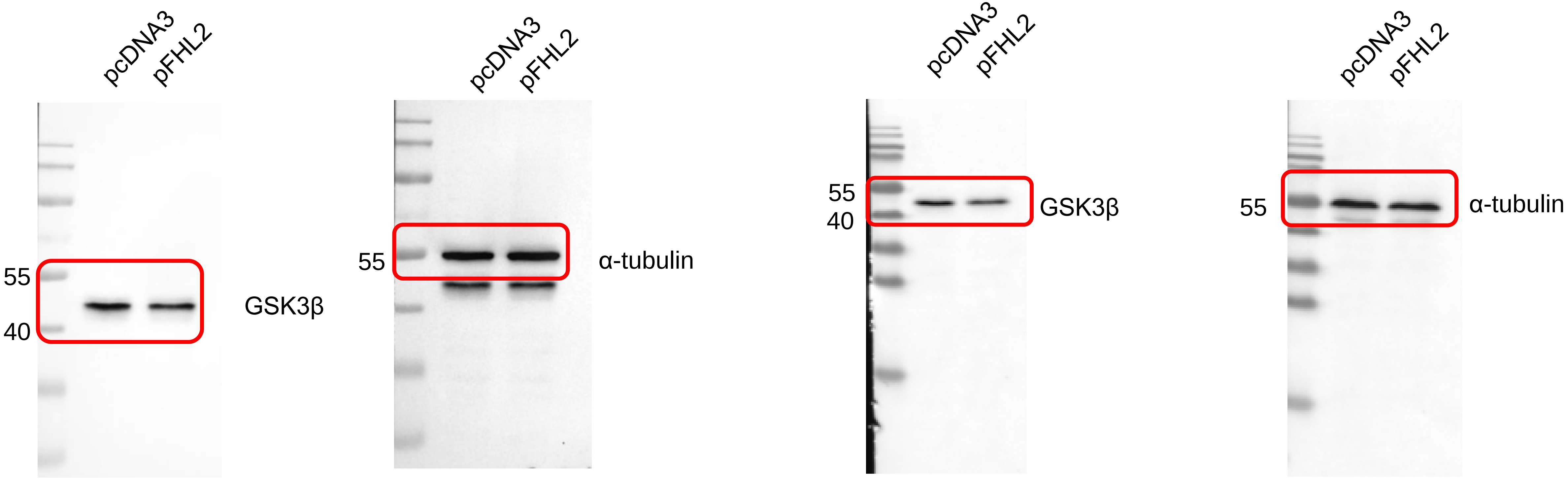

(data not shown) (data not shown) (data not shown)

Figure 7f

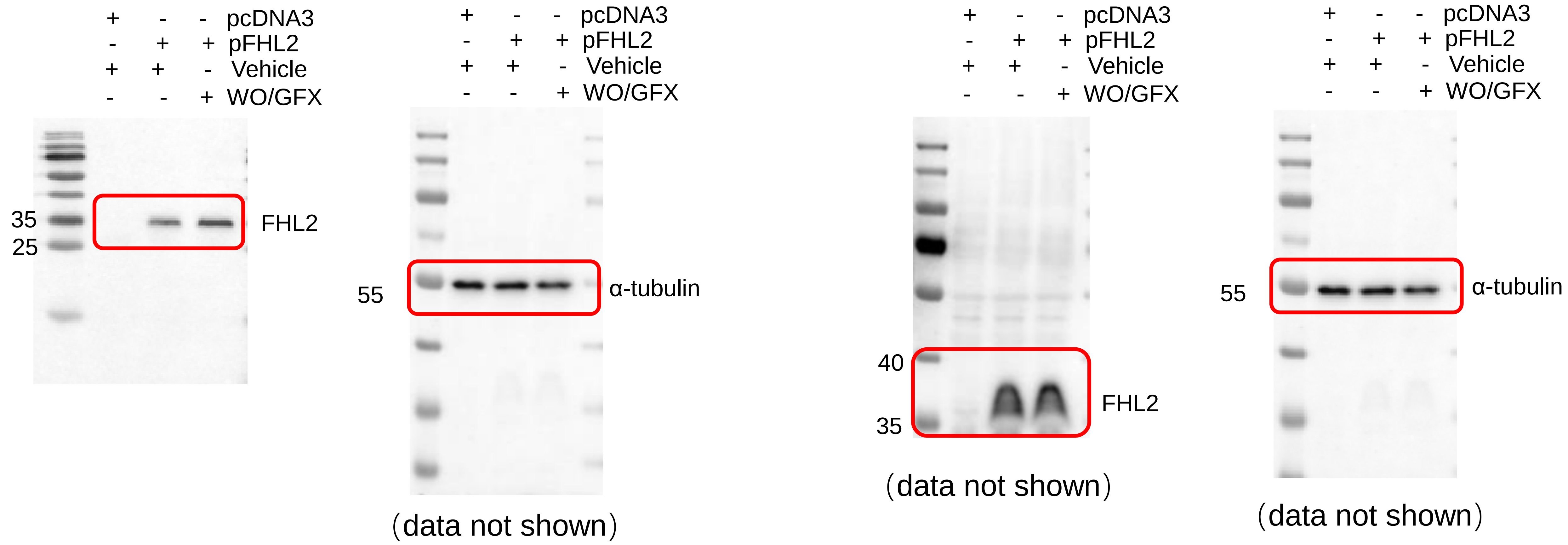

+ - - pcDNA3
- + + pFHL2
+ + - Vehicle
- - + WO/GFX
35
25
FHL2
+ - - pcDNA3
- + + pFHL2
+ + - Vehicle
- - + WO/GFX
55
α-tubulin
(data not shown)
+ - - pcDNA3
- + + pFHL2
+ + - Vehicle
- - + WO/GFX
40
35
FHL2
(data not shown)
+ - - pcDNA3
- + + pFHL2
+ + - Vehicle
- - + WO/GFX
55
α-tubulin
(data not shown)

Figure 7f

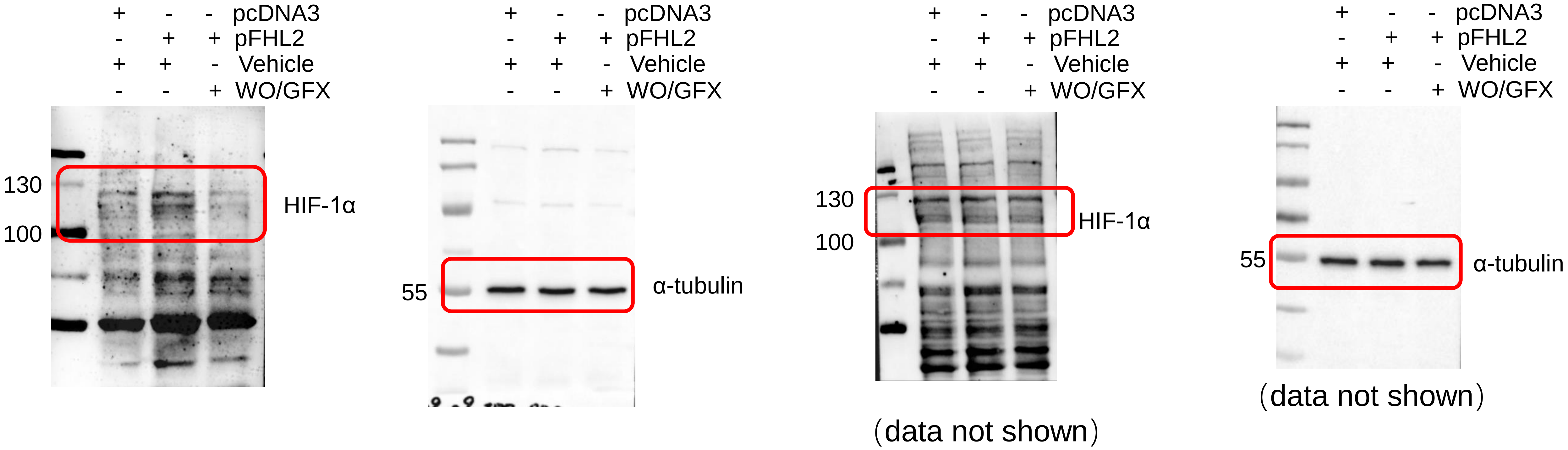
+ - - pcDNA3
- + + pFHL2
+ + - Vehicle
- - + WO/GFX
130
100
HIF-1α
55
α-tubulin
(data not shown)
(data not shown)

Figure 7f

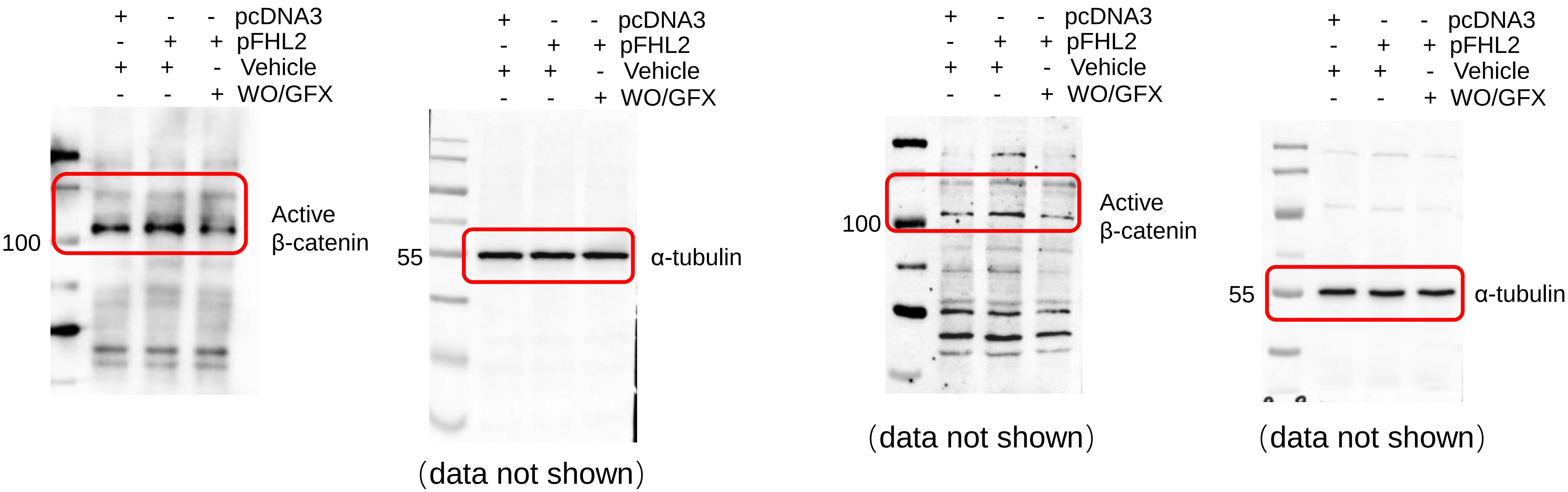

+ - - pcDNA3
- + + pFHL2
+ + - Vehicle
- - + WO/GFX
100
Active β-catenin
+ - - pcDNA3
- + + pFHL2
+ + - Vehicle
- - + WO/GFX
55
α-tubulin
(data not shown)
+ - - pcDNA3
- + + pFHL2
+ + - Vehicle
- - + WO/GFX
100
Active β-catenin
(data not shown)
+ - - pcDNA3
- + + pFHL2
+ + - Vehicle
- - + WO/GFX
55
α-tubulin
(data not shown)

Figure 7f

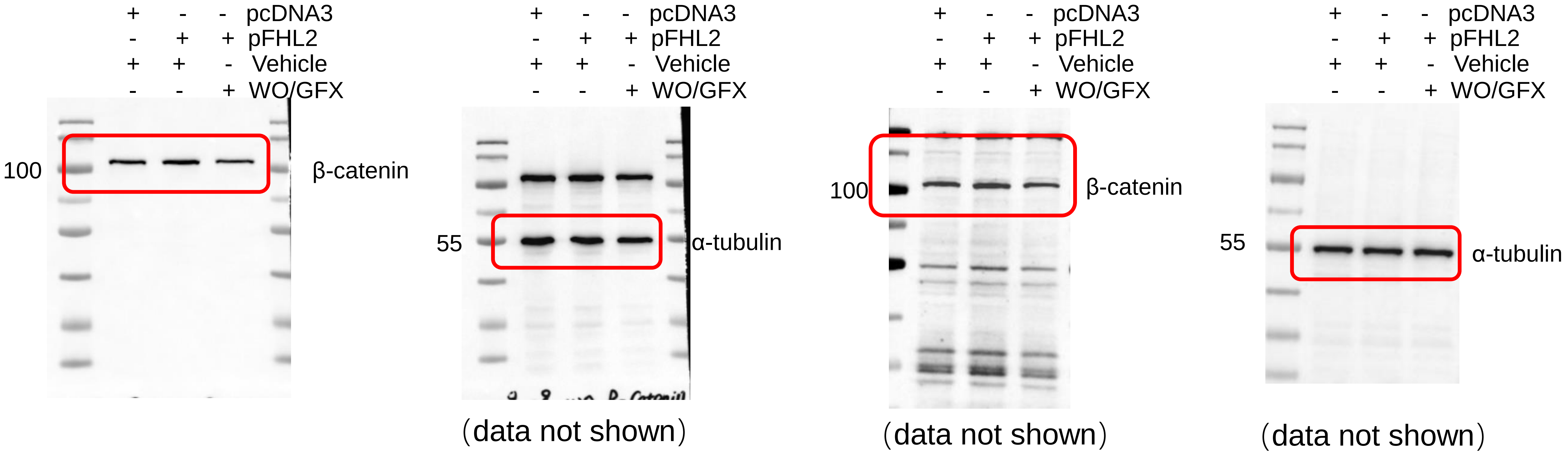
+ - - pcDNA3
- + + pFHL2
+ + - Vehicle
- - + WO/GFX
100
β-catenin
55
α-tubulin
(data not shown)
100
β-catenin
(data not shown)
55
α-tubulin
(data not shown)

Figure 7i

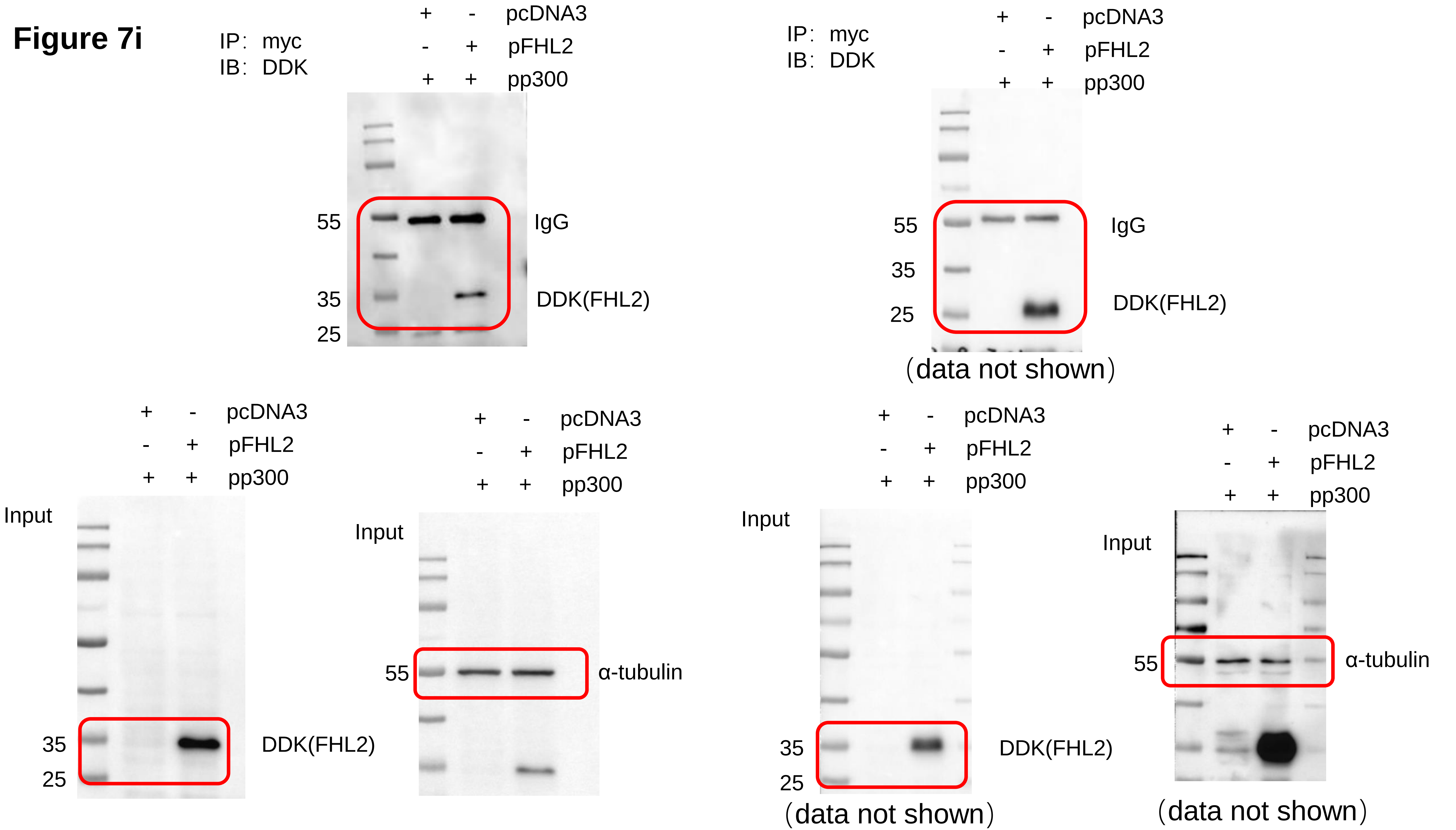

IP: myc
IB: DDK
pcDNA3
pFHL2
pp300
55
35
25
IgG
DDK(FHL2)
(data not shown)
Input
α-tubulin

Figure 7i

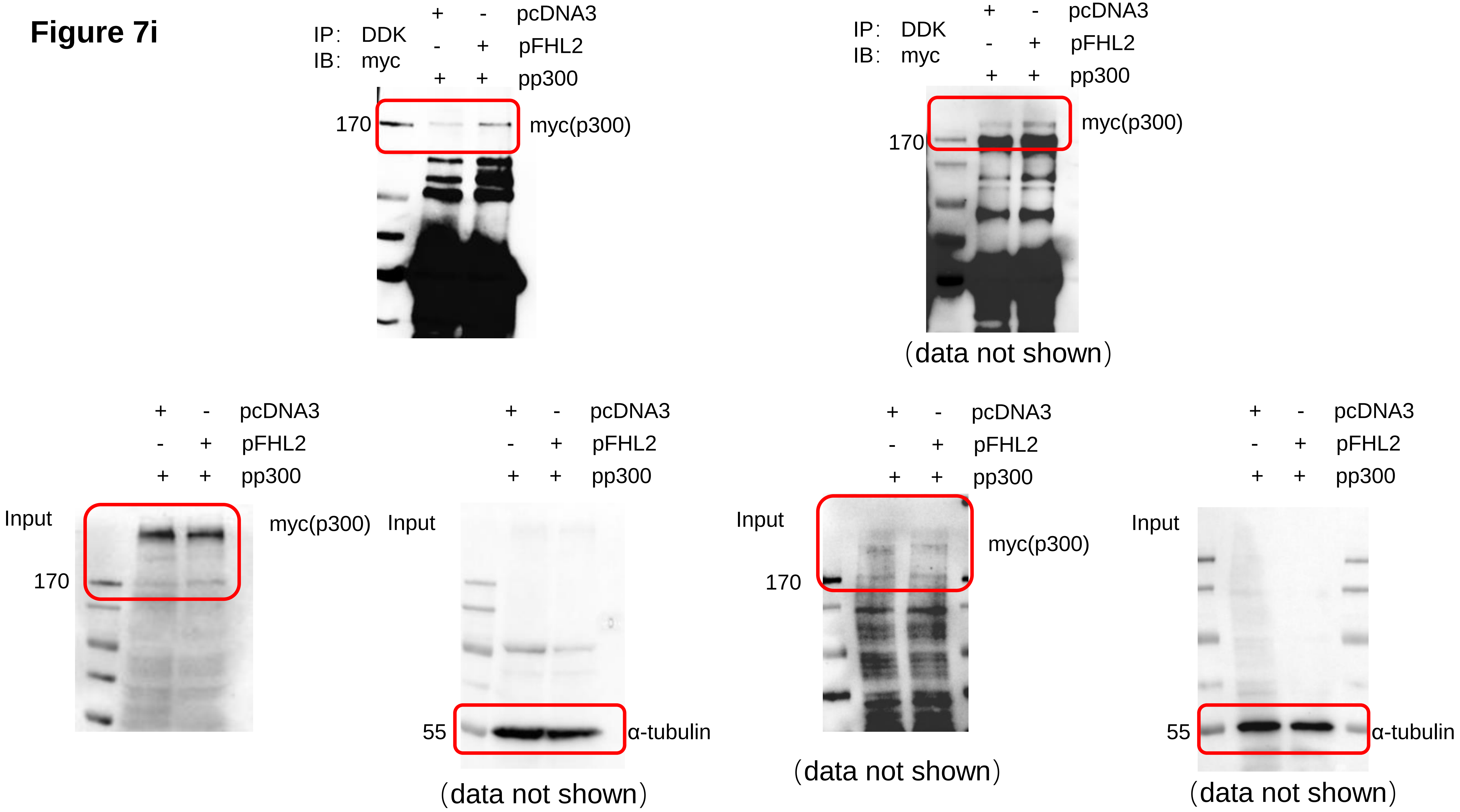
IP: DDK
IB: myc
+ - pcDNA3
- + pFHL2
+ + pp300
170
myc(p300)
(data not shown)
Input
55
α-tubulin

Figure 7j

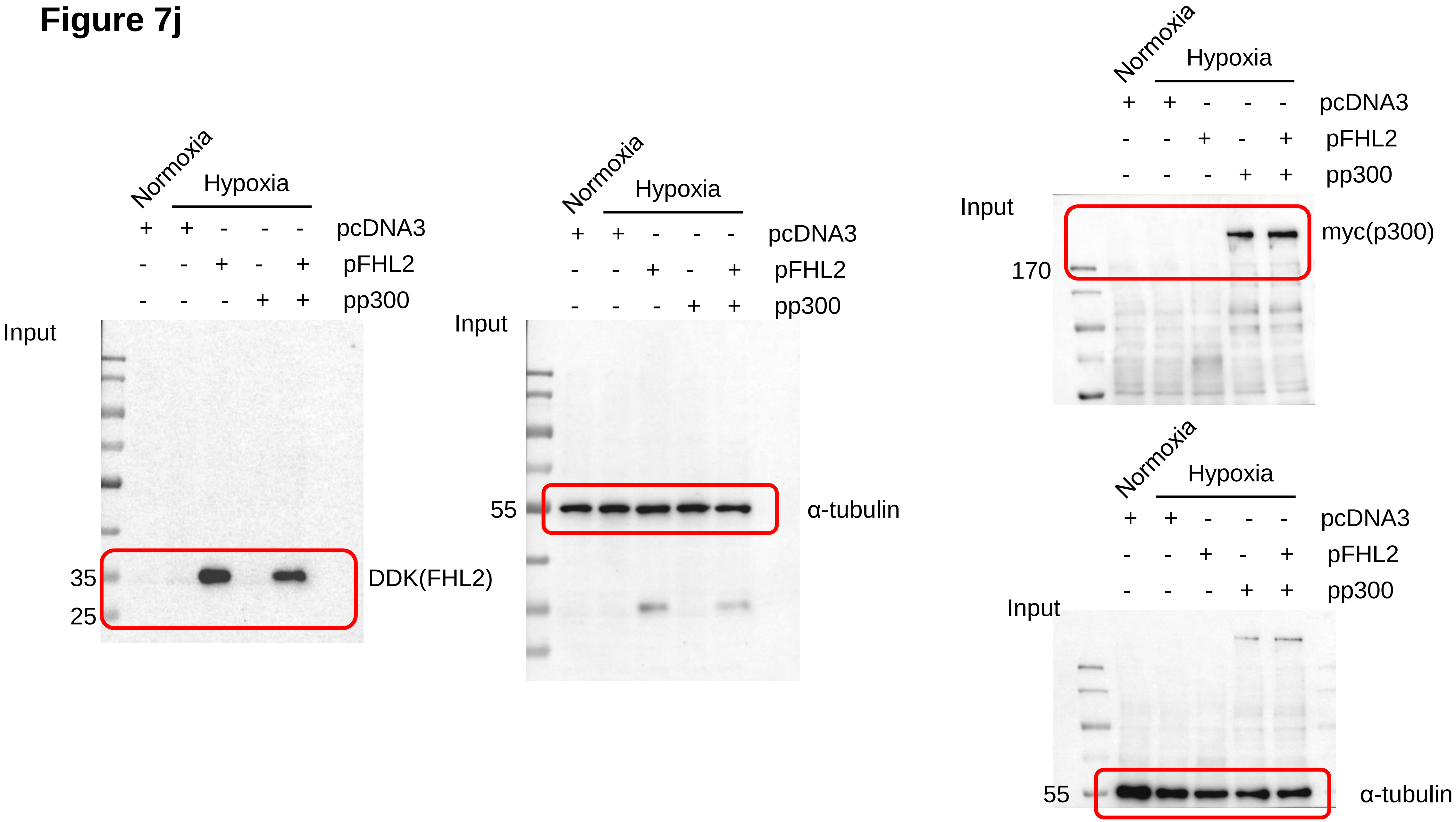

（data not shown）

Figure 7j

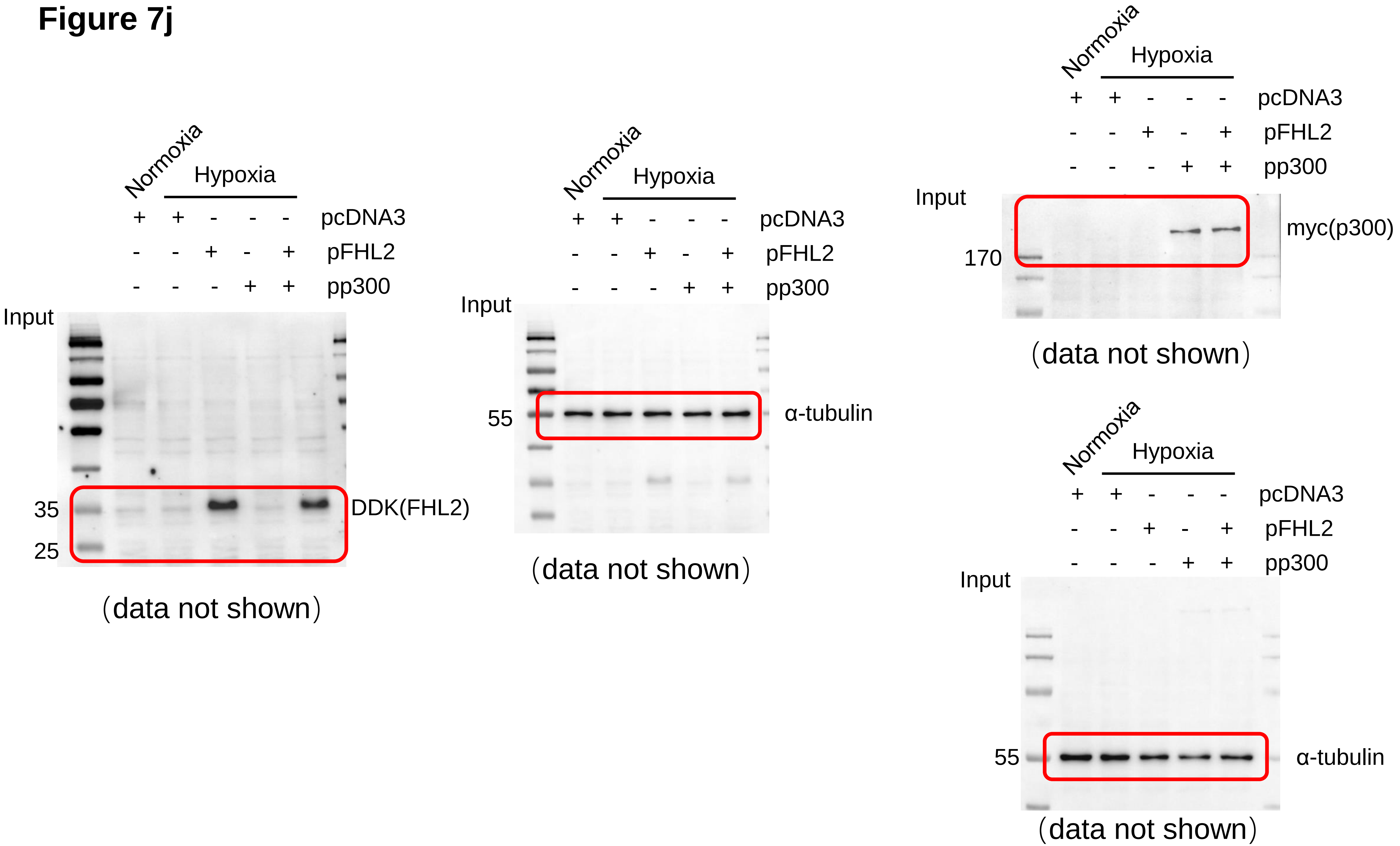
Normoxia
Hypoxia
+ + - - - pcDNA3
- - + - + pFHL2
- - - + + pp300
Input
35
25
DDK(FHL2)
(data not shown)
Normoxia
Hypoxia
+ + - - - pcDNA3
- - + - + pFHL2
- - - + + pp300
Input
55
α-tubulin
(data not shown)
Normoxia
Hypoxia
+ + - - - pcDNA3
- - + - + pFHL2
- - - + + pp300
Input
170
myc(p300)
(data not shown)
Normoxia
Hypoxia
+ + - - - pcDNA3
- - + - + pFHL2
- - - + + pp300
Input
55
α-tubulin
(data not shown)

Figure 7j

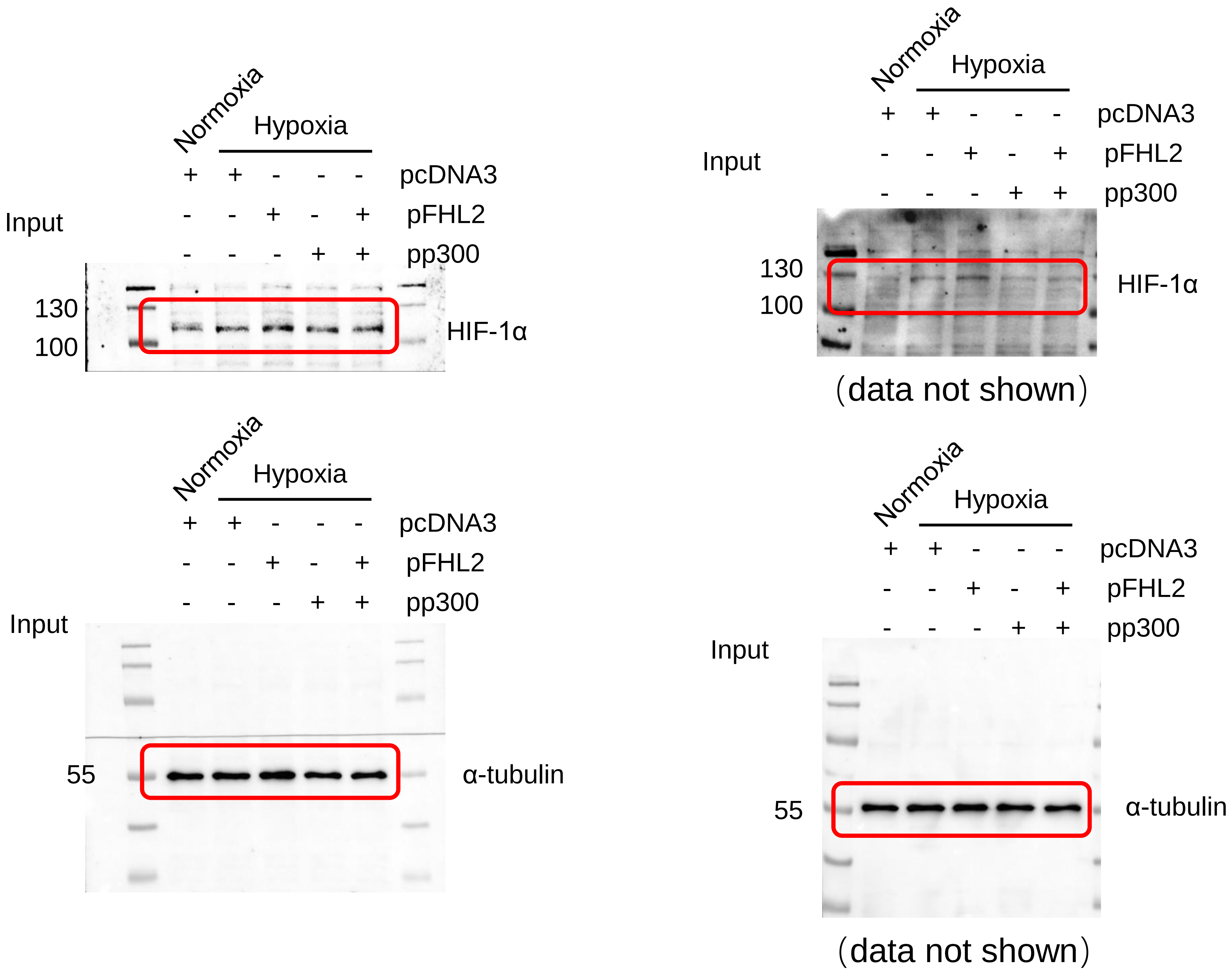
Normoxia
Hypoxia
Input
+ + - - - pcDNA3
- - + - + pFHL2
- - - + + pp300
130
100
HIF-1α
(data not shown)
Normoxia
Hypoxia
Input
+ + - - - pcDNA3
- - + - + pFHL2
- - - + + pp300
55
α-tubulin
(data not shown)

Figure 7j

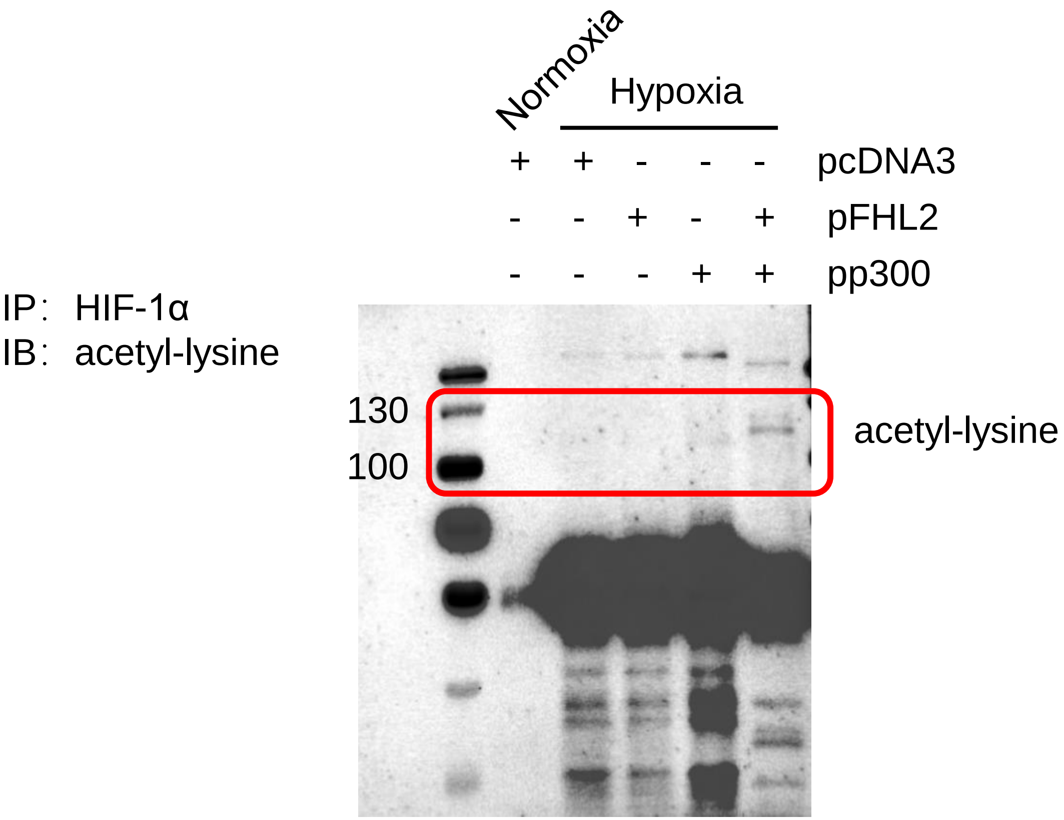

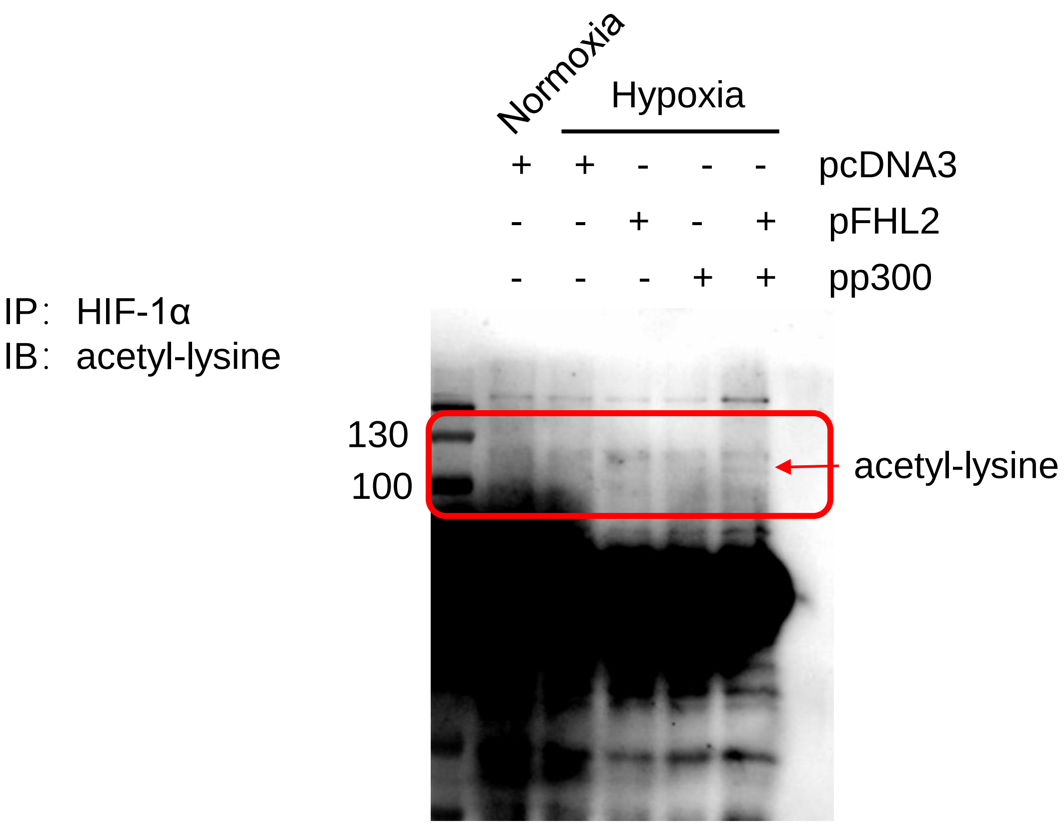

(data not shown)

Figure 7k

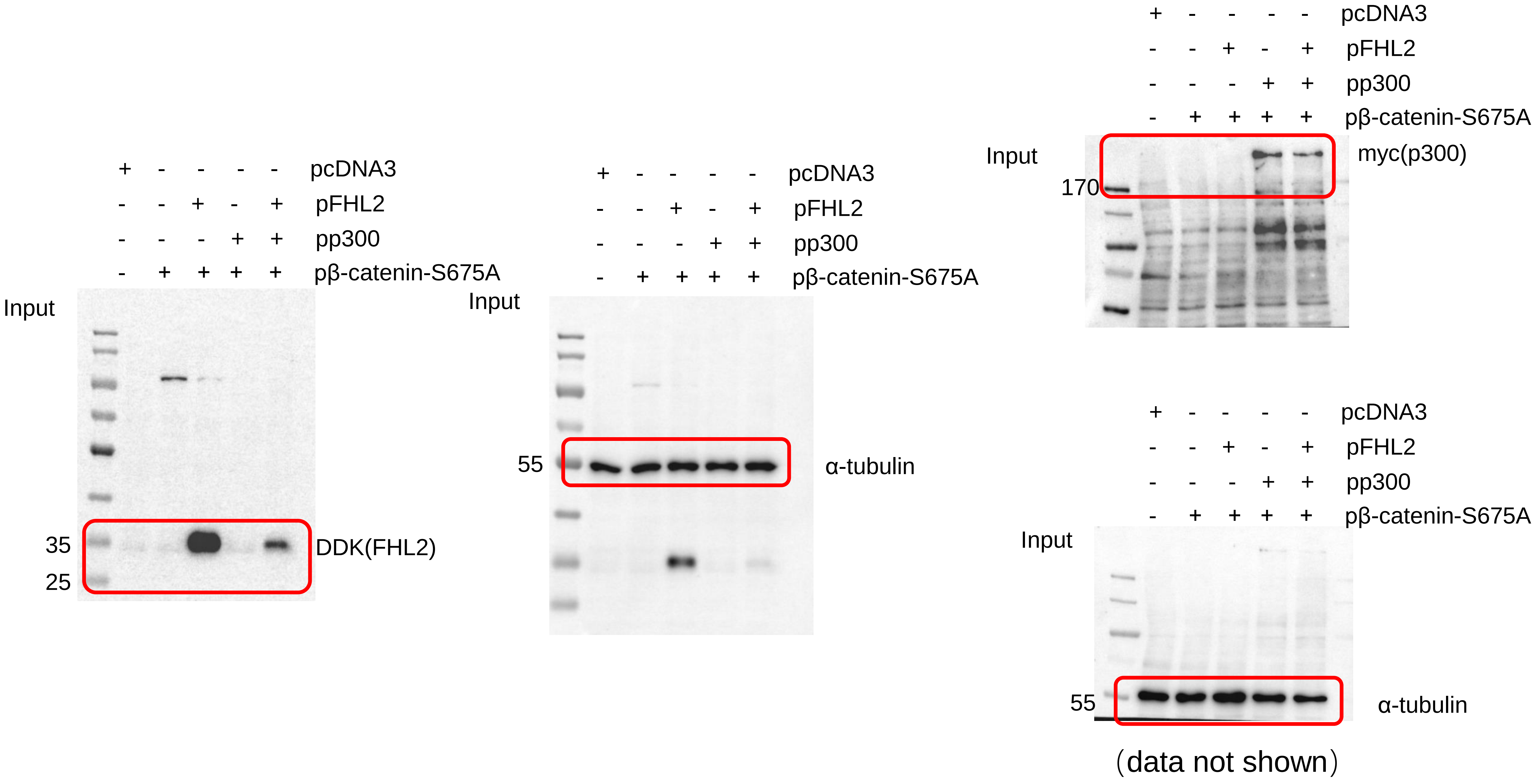
+ - - - - pcDNA3
- - + - + pFHL2
- - - + + pp300
- + + + + pβ-catenin-S675A
Input
35
25
DDK(FHL2)
+ - - - - pcDNA3
- - + - + pFHL2
- - - + + pp300
- + + + + pβ-catenin-S675A
Input
55
α-tubulin
+ - - - - pcDNA3
- - + - + pFHL2
- - - + + pp300
- + + + + pβ-catenin-S675A
Input
170
myc(p300)
+ - - - - pcDNA3
- - + - + pFHL2
- - - + + pp300
- + + + + pβ-catenin-S675A
Input
55
α-tubulin
(data not shown)

Figure 7k

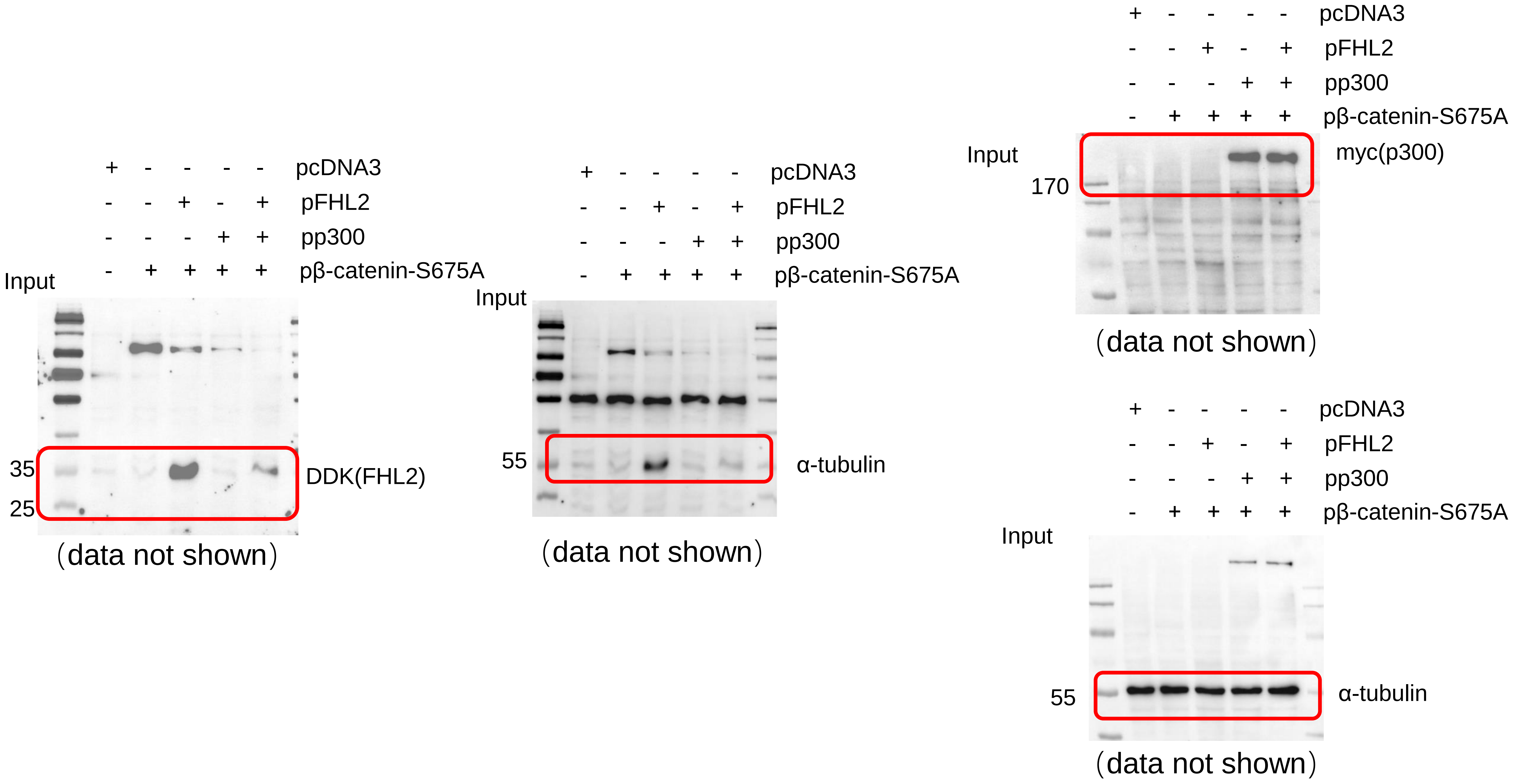
+ - - - - pcDNA3
- - + - + pFHL2
- - - + + pp300
- + + + + pβ-catenin-S675A
Input
35
25
DDK(FHL2)
(data not shown)
+ - - - - pcDNA3
- - + - + pFHL2
- - - + + pp300
- + + + + pβ-catenin-S675A
Input
55
α-tubulin
(data not shown)
+ - - - - pcDNA3
- - + - + pFHL2
- - - + + pp300
- + + + + pβ-catenin-S675A
Input
170
myc(p300)
(data not shown)
+ - - - - pcDNA3
- - + - + pFHL2
- - - + + pp300
- + + + + pβ-catenin-S675A
Input
55
α-tubulin
(data not shown)

Figure 7k

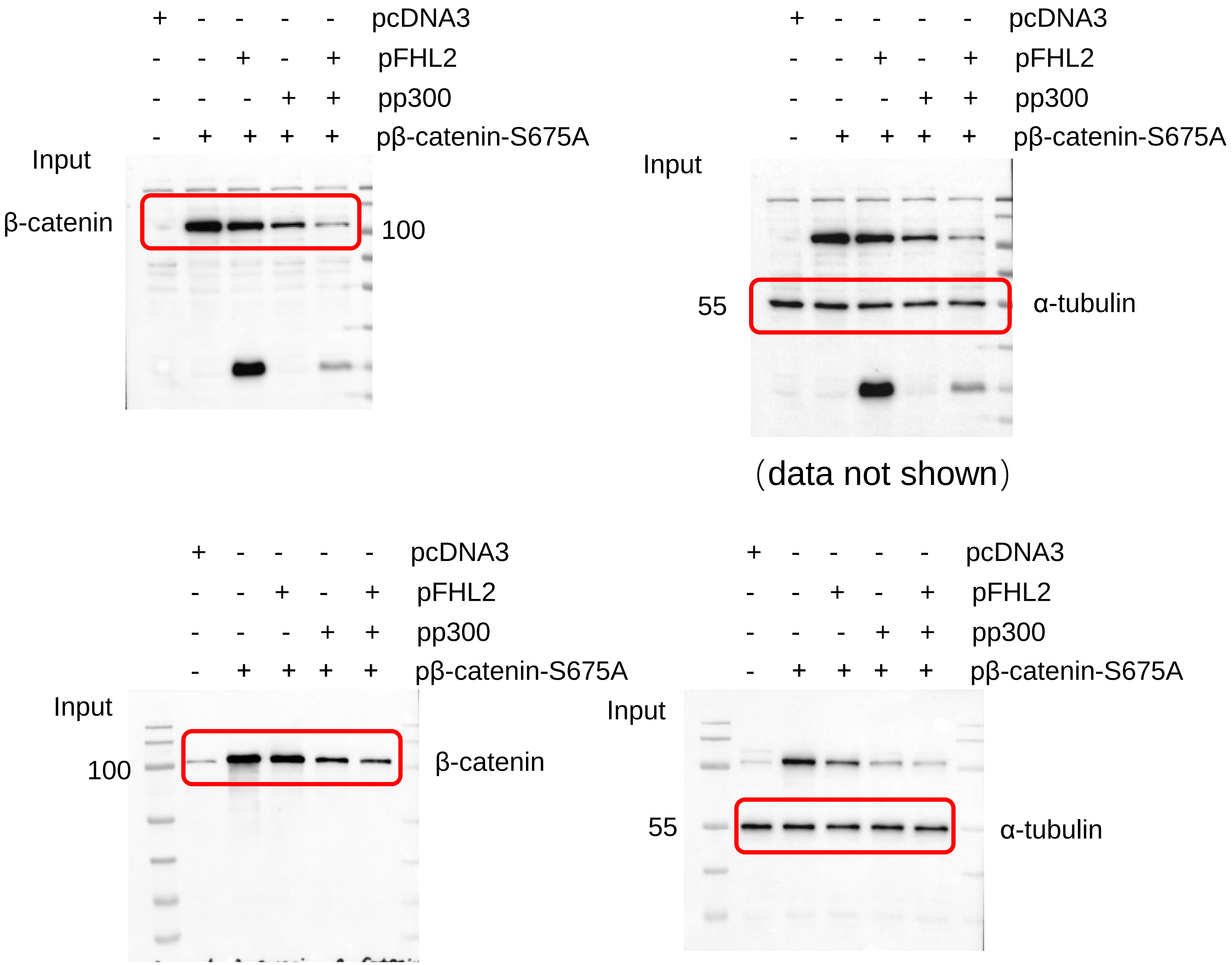

+ - - - - pcDNA3
- - + - + pFHL2
- - - + + pp300
- + + + + pβ-catenin-S675A
Input
β-catenin
100
+ - - - - pcDNA3
- - + - + pFHL2
- - - + + pp300
- + + + + pβ-catenin-S675A
Input
55
α-tubulin
(data not shown)
+ - - - - pcDNA3
- - + - + pFHL2
- - - + + pp300
- + + + + pβ-catenin-S675A
Input
100
β-catenin
+ - - - - pcDNA3
- - + - + pFHL2
- - - + + pp300
- + + + + pβ-catenin-S675A
Input
55
α-tubulin

Figure 7k

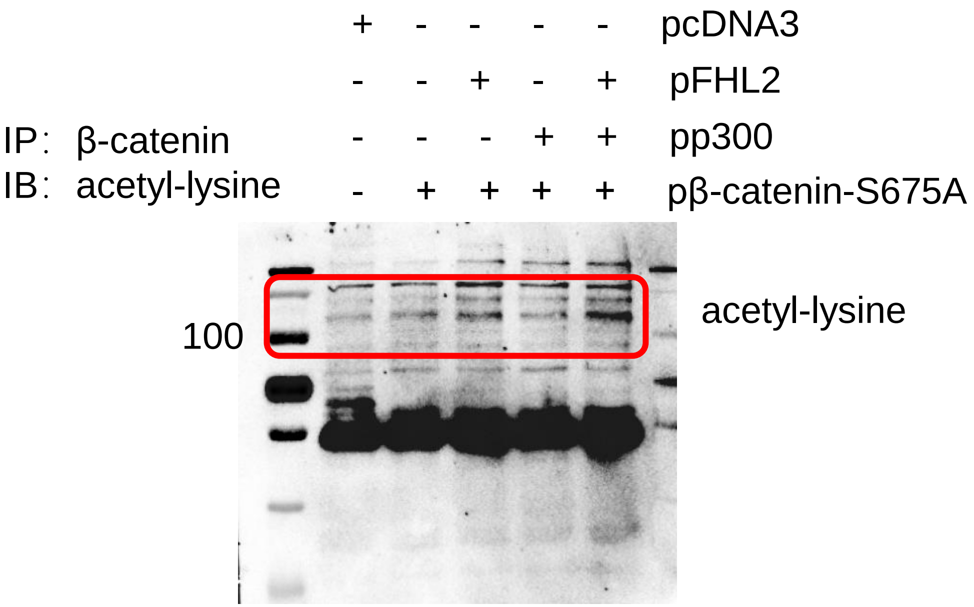

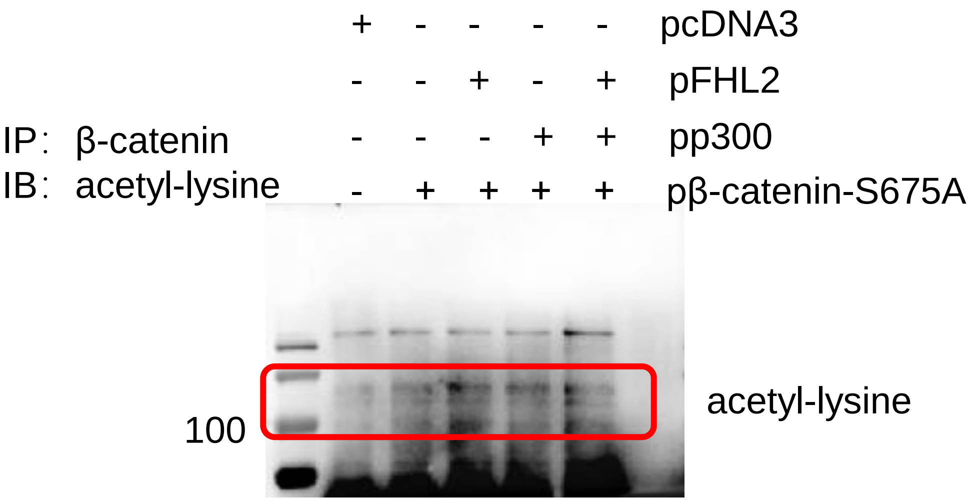

(data not shown)
